# DNA Barcoding the Canadian Arctic Flora: Core Plastid Barcodes (*rbcL* + *matK*) for 490 Vascular Plant Species

**DOI:** 10.1371/journal.pone.0077982

**Published:** 2013-10-22

**Authors:** Jeffery M. Saarela, Paul C. Sokoloff, Lynn J. Gillespie, Laurie L. Consaul, Roger D. Bull

**Affiliations:** Botany Section, Research and Collections Services, Canadian Museum of Nature, Ottawa, Ontario, Canada; University of Milano Bicocca, Italy

## Abstract

Accurate identification of Arctic plant species is critical for understanding potential climate-induced changes in their diversity and distributions. To facilitate rapid identification we generated DNA barcodes for the core plastid barcode loci (*rbcL* and *matK*) for 490 vascular plant species, representing nearly half of the Canadian Arctic flora and 93% of the flora of the Canadian Arctic Archipelago. Sequence recovery was higher for *rbcL* than *matK* (93% and 81%), and *rbcL* was easier to recover than *matK* from herbarium specimens (92% and 77%). Distance-based and sequence-similarity analyses of combined *rbcL* + *matK* data discriminate 97% of genera, 56% of species, and 7% of infraspecific taxa. There is a significant negative correlation between the number of species sampled per genus and the percent species resolution per genus. We characterize barcode variation in detail in the ten largest genera sampled (*Carex*, *Draba*, *Festuca*, *Pedicularis*, *Poa*, *Potentilla*, *Puccinellia*, *Ranunculus, Salix*, and *Saxifraga*) in the context of their phylogenetic relationships and taxonomy. Discrimination with the core barcode loci in these genera ranges from 0% in *Salix* to 85% in *Carex*. Haplotype variation in multiple genera does not correspond to species boundaries, including *Taraxacum*, in which the distribution of plastid haplotypes among Arctic species is consistent with plastid variation documented in non-Arctic species. Introgression of *Poa glauca* plastid DNA into multiple individuals of *P. hartzii* is problematic for identification of these species with DNA barcodes. Of three supplementary barcode loci (*psbA–trnH*, *psbK–psbI*, *atpF–atpH*) collected for a subset of *Poa* and *Puccinellia* species, only *atpF–atpH* improved discrimination in *Puccinellia*, compared with *rbcL* and *matK*. Variation in *matK* in *Vaccinium uliginosum* and *rbcL* in *Saxifraga oppositifolia* corresponds to variation in other loci used to characterize the phylogeographic histories of these Arctic-alpine species.

## Introduction

Arctic regions of the world are changing rapidly in response to climate change. Understanding the diversity and distributions of Arctic plant species in the past and present is critical for documenting change, and accurate identification of plant species underpins all research on Arctic vegetation. Changes in Arctic vegetation linked to climate change include increased plant productivity [1–6], shrub expansion [7–11], changes in community composition (species diversity and abundance) of varying degree [1,4,12–15], and treeline advancement [16].

The Arctic ecozone in Canada extends from northwestern Yukon to northern Newfoundland and Labrador and represents 36% of the global Arctic [17,18]. The Canadian Arctic flora has been studied since explorers searched for the Northwest Passage in the early 1800s (e.g., [19]), but knowledge of its composition, distribution, and evolutionary history remains incomplete. There are several Arctic genera in which species boundaries are not clear and/or species are difficult to identify [20]. Authoritative floras and checklists synthesizing knowledge of Canadian Arctic plants have been published [20–25] and there is an extensive literature reporting floristic discoveries in the Arctic (e.g., [26–30]), but there is no vascular plant flora covering the entire Canadian Arctic ecozone. The number of vascular plant species in this large region is not known with precision. The Panarctic Flora divides the global Arctic—defined fairly broadly in North America to include regions with numerous borderline Arctic taxa in the Brooks Range, along the Mackenzie River Delta, near Great Bear Lake, and along Hudson Bay—into biogeographical zones, and provides data on the distribution and frequency of taxa in each zone [20]. The North American Arctic is divided into six zones: Western Alaska, Northern Alaska–Yukon Territory, Central Canada, Hudson Bay–Labrador, Ellesmere Land–Northern Greenland, and Western Greenland. There are some 1100 vascular plant species in the four zones that include Canada [20], but the number of species restricted to Canada is probably lower, as there are species in the trans-national zones that do not occur in Canada. In the Canadian Arctic Archipelago, a more easily defined geographical region, there are 341 vascular plant species (349 taxa) [25].

### DNA Barcoding

DNA barcoding is the use of short regions of DNA—the DNA barcode—to identify species by assigning individuals to known taxa through comparison of their barcodes with a reference library. The search for a plant DNA barcode has been challenging. The mitochondrial locus cytochrome oxidase I (COI) widely used as a DNA barcode for animal taxa (e.g., [31–33]) is not suitable as a plant barcode due to its (usually) low variability—a function of the (usually) low substitution rate of the mitochondrial genome in plants compared with animals [34–37] and substantial variation in plant mitochondrial genome structure [38]. Multiple coding and non-coding plastid loci, alone or combined, have been proposed and tested as potential plant DNA barcodes (e.g., [34,37,39–50], reviewed in Hollingsworth et al. [51]). A comparison of the performances of eight of these regions demonstrated that (1) multi-locus plant barcodes perform better than single-locus barcodes, (2) various combinations of plastid regions performed similarly, and (3) a maximum of 71% of species could be distinguished with plastid barcodes [34]. The Consortium for the Barcode of Life (CBOL) Plant Working Group [52] conducted a broader study of the performance of candidate barcode regions (*atpF–atpH*, *matK*, *rbcL*, *rpoB*, *rpoC1*, *psbK–psbI*, *psbA–trnH*), and recommended *rbcL* + *matK* as the standard or core plant barcode. Recognizing that these two loci discriminate only some 70% of plant species, they acknowledged that supplementary loci will be required to improve identification success in many plant groups. The Executive Committee of the Consortium for the Barcode of Life (2009) approved *matK* and *rbcL* as the required core barcode regions for land plants, and also encouraged researchers to collect data from supplementary non-coding loci [53]. 

The nuclear ribosomal internal transcribed spacer regions (ITS1 and ITS2) were also initially considered as possible plant barcode regions (e.g., [37,39,54]), but dismissed due to complicating problems, including fungal contamination, paralogous copies, and amplification difficulties [51,55]. Despite these limitations, recent studies have demonstrated substantially higher discrimination rates for one or both ITS regions compared with plastid barcodes [56–58]. The largest of these was conducted by the China Plant BOL Group [58], who tested the performance of four barcoding regions (*rbcL*, *matK*, *psbA–trnH*, ITS) in a large data set (6,286 individuals, 1,757 species), and found ITS alone had the highest discriminatory power. There is renewed interest in ITS as an additional core plant barcode region [55,58], and many plant barcoding studies are now including ITS along with the core and/or other plastid loci.

There has been an explosion of plant DNA barcoding studies in recent years that test the performance of candidate DNA barcode regions and recommend markers best suited to particular taxa. Many have focused on individual or closely related genera [59–73], families [44,74–83], and higher taxa [84–87]. Studies that sample multiple closely related species generally have the lowest species resolution among barcoding studies, whereas studies that do not sample multiple close relatives generally have the highest species discrimination [51]. The latter includes studies of non-taxonomic groups such as medicinal plants [57,88,89], crop species [90,91], and invasive species [92,93]. Another subset of non-taxonomic studies has focused on barcoding the floras or subsets of the floras of restricted geographical areas, in which diversity is lower and fewer closely related species are expected compared with floras of broader geographical areas. Species identification with DNA barcodes in some of these studies has been high: >98% for forest plots on Barro Colorado Island in Panama [94]; >90% for orchids of Mesoamerica and trees, shrubs and achlorophyllous parasites from Kruger National Park, South Africa [43]; >93% for the Koffler Scientific Reserve, Ontario, Canada [95]; ca. 85% for the Japanese pteridophyte flora [96]; and high (no percentage given) for NW-European ferns [87]. In other geographically restricted barcoding studies, species resolution was considerably lower: up to 70% for tropical tree species in French Guiana [97], and 69.4–74.9% for the flora of Wales [98], for example.

Despite their inability to unambiguously identify all plant species, plant DNA barcodes will be useful in many situations [51]. Already the application of plant DNA barcoding has been demonstrated in a number of studies, including an analysis of spatial variation in root diversity [99], accurate identification of a horticultural fern [100], authenticity of natural health products [101], and diet analyses of herbivorous animals [102] and leaf beetles [103]. Analyses of barcode data have also aided the discovery of new plant and algae species [104,105].

### Barcoding Arctic Plants

Barcode data have been produced for some Arctic plant species, but coverage is incomplete. The *trnL*(UAA) intron and its P6 loop have been used as a DNA barcode for Norwegian Arctic plants [50], and these data have been used to study plant–herbivore interactions [106] and composition of past Arctic plant communities based on ancient DNA [107]. Barcode data for the core plastid loci are available for 23 species of Carex L. and *Kobresia* Willd. (Cyperaceae) from the Canadian Arctic Archipelago [108], and data for the core plastid regions plus ITS2 were recently published for 312 of 354 vascular plant species known from Churchill, Manitoba, at the southern edge of the Arctic in central Canada [109]. In this study, the three barcode regions combined identified 69% of species, while *rbcL* + *matK* identified 54% of species. 

Advancement of a DNA barcode library for Arctic plants will facilitate plant identification for taxonomic research, vegetation monitoring, as well as floristic and ecological studies, and will contribute to knowledge of genetic diversity in Arctic plants. As barcode data accumulate for arctic-alpine plant species from throughout their global ranges, the growing barcode library may also contribute to our understanding of the origins and distribution of Arctic flora. 

Here we report new DNA barcode data for the core plastid regions (*rbcL* + *matK*) for 490 Arctic and northern vascular plant species (over 500 taxa) from Canada, and data for three supplementary plastid loci (*atpF–atpH*, *psbA–trnH*, *psbK–psbI*) for a subset of *Puccinellia* Parl. and *Poa* L. species. We characterize the ability of the barcode loci to discriminate genera, species, and infraspecific taxa, and in a subset of genera we explore patterns of genetic variation from taxonomic, phylogenetic, and phylogeographic perspectives. 

## Methods

### Taxon Sampling

**Figure 1:**
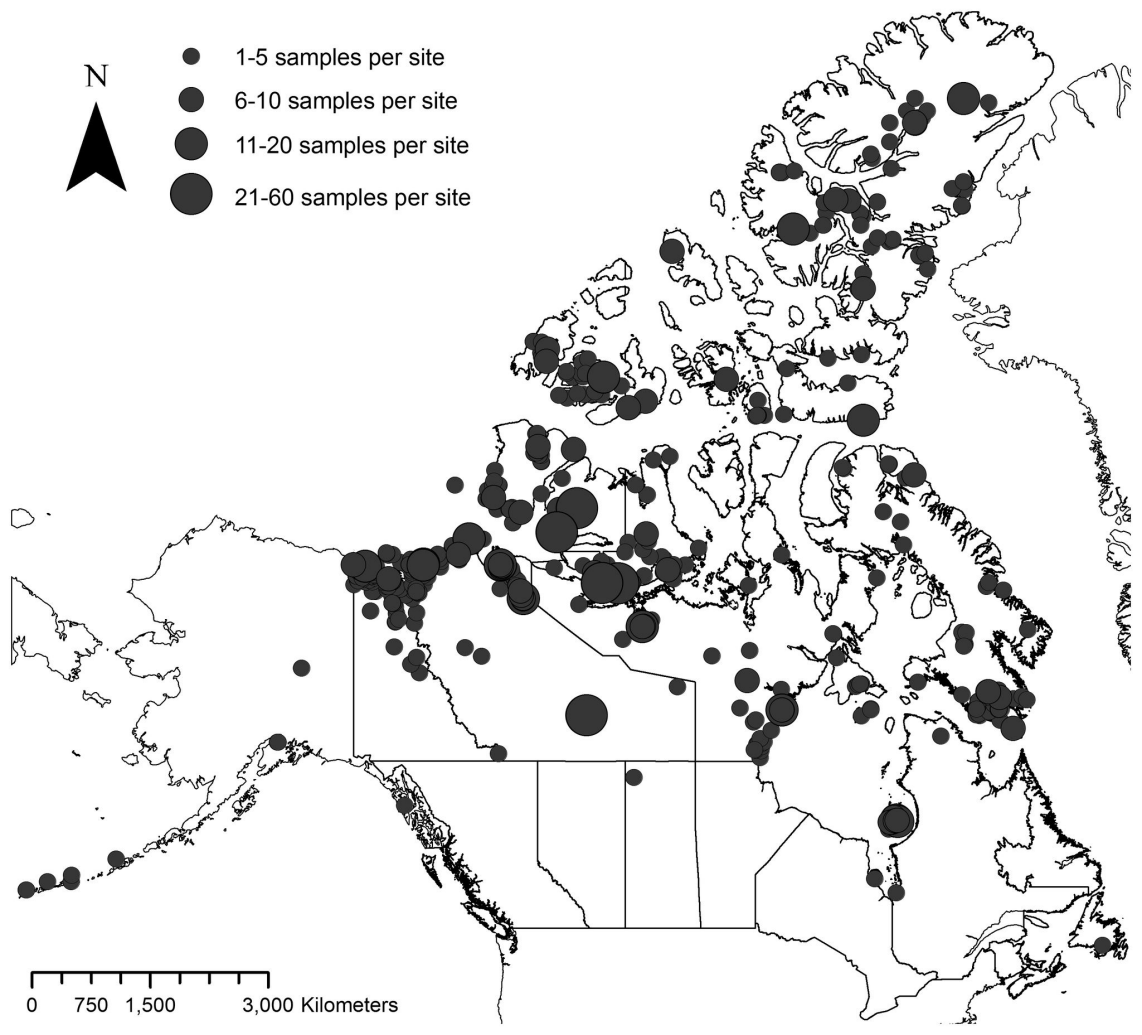
Map of sample locations in Canada and Alaska, U.S.A.

The study includes 2644 individuals representing 490 vascular plant species plus 30 additional infraspecific taxa from 50 plant families. Plant material used in the study comes from throughout the Canadian Arctic, with a few specimens from the adjacent boreal region (e.g., Yellowknife, Northwest Territories) and Alaska, U.S.A. (Figure 1). The number of individuals sampled per taxon ranged from 1–27 (mean = 5.0 ± 4.2) (Figure S1). All data for the project were managed in the Barcode of Life Systems (BOLD) database [110] in a project called "Flora of the Canadian Arctic" (project code FCA). Detailed voucher information, including the scientific names of taxa sampled, locality information, collection dates, collectors and collection numbers, herbarium accession numbers, and GenBank accession numbers for all sequences are given in Data Set S1. All voucher specimens were annotated with a label recording their corresponding BOLD Sample ID. Voucher specimens were scanned with Epson flatbed scanners at a resolution of 600 dots per inch, and images were uploaded to BOLD. 

A large proportion of the barcode data was generated from silica-gel dried leaf tissue samples collected in the western Arctic as part of floristic studies undertaken by the authors in 2008, 2009, and 2010 (J.M. Saarela and L.J. Gillespie, unpublished data, [29,30]), and field trips throughout the Canadian Arctic Archipelago by Gillespie and L.L. Consaul in the 1990s and 2000s. Fieldwork on Victoria Island, Nunavut, in 2008 was conducted under Nunavut Wildlife Research Permit No. WL 2008-1039 and Nunavut Water Board Licence No. 3BC-AFP0813. Fieldwork on mainland Northwest Territories in 2009 was conducted under Aurora Research Institute Licence No.14524, Inuvialuit Land Administration Licence No. ILA09PN009, and with permission from Parks Canada to conduct fieldwork in Tuktut Nogait National Park of Canada. Fieldwork on Victoria Island, Northwest Territories in 2010 was conducted under Aurora Research Institute Licence No. 14733 and Inuvialuit Land Administration Licence No. ILA10HN004. Voucher specimens for these collections are housed in the National Herbarium of Canada (CAN), Canadian Museum of Nature, Natural Heritage Campus, Gatineau (Data Set S1).

In addition to field-collected material, we also sampled species from CAN herbarium specimens, to expand our taxonomic coverage and geographic sampling. Where possible, we sampled the most recently collected herbarium specimens available, and in most cases we only sampled herbarium material that was green, indicative of fast drying. Sampled herbarium specimens were collected between 1950 and 2010. A small subset of samples was amplified and sequenced from existing DNA extracts. 

### Taxonomy

Higher-level taxonomy follows recent family-level classifications for lycophytes [111], gymnosperms [112], monilophytes [113], and angiosperms [114]. For nomenclature at the genus, species, and infraspecific levels, we considered available taxonomic information in combination with our experiences with the Arctic flora, as there are many conflicting treatments of species and/or species complexes, even among recent literature [20]. Many Arctic taxa have been variously recognized as species, varieties, or subspecies with little consensus on an appropriate rank, and barcode data may provide new insights that could be taxonomically informative. We thus made a concerted effort to identify taxa to infraspecific rank, even when only one infraspecific taxon was present in our data set, expecting that future growth of the barcode reference library will add infraspecific taxa not sampled here. In most cases infraspecific taxa are represented by the nominal subspecies or variety and one additional infraspecific taxon, and for a few species we sampled more than two additional infraspecific taxa. 

We identified our recent field collections and confirmed or re-determined all sampled herbarium specimens through extensive consultation with older [22–24] and recent Arctic floras [25,115], available volumes of the *Flora of North America* series [116], and the *Annotated Checklist of the Panarctic Flora* (*PAF*)*: Vascular Plants* [20]. For many genera, particularly those that are taxonomically difficult, we also consulted the primary taxonomic literature (e.g., *Arctagrostis* Griseb. [117], *Braya* Sternb. & Hoppe [118], *Chrysosplenium* L. [119], *Draba* L [120]., *Eriophorum* L. [121], *Festuca* L. [122–124], *Juncus* L [125]., *Luzula* DC [126]., *Papaver* L. [127], *Pedicularis* L. [128], *Petasites* Mill. [129,130], *Poa* [131–133], *Puccinellia* [134–143]). All willows (*Salix* L.) were determined by G.L. Argus (Canadian Museum of Nature). A subset of the *Draba*, *Papaver*, *Potentilla* L., and *Hippuris* L. material was identified by R. Elven (Natural History Museum, University of Oslo). Nomenclatural information was also obtained from the online databases VASCAN [144,145], Tropicos [146], the International Plant Names Index [147], and the Taxonomic Names Resolution service (http://tnrs.iplantcollaborative.org; version 3.0; accessed December 2012).

### DNA Sequencing and Alignment

DNA extraction, amplification, and sequencing of *matK* and *rbcL* followed the protocols of the Canadian Centre for DNA Barcoding (CCDB) [148–153], as summarized in Kuzmina et al. [109]. *rbcL* was amplified and sequenced using the primers *rbcL*a–F (Levin et al. [154], modified from Soltis et al. [155]) and *rbcL*a–R (Kress et al. [94], modified from Fofana et al. [156]). *matK* was amplified in two successive rounds, as necessary. First, all samples were amplified with *matK*–1RKIM–f and *matK*–3FKIM–r [157]. Failed samples from the first PCR round were then amplified with *matK*–390f and *matK*–1326r [158]. All primer sequences can also be found on the CCDB Protocols website [157]. For a subset of *Poa* and *Puccinellia* individuals (Data Set S1) we also sequenced *psbA–trnH* (20 taxa, 57 individuals; primers [159]:), *psbK–psbI* (20 taxa, 57 individuals), and *atpF–atpH* (19 taxa, 56 individuals). Unpublished primers for *psbK–psbI* and *atpF–atpH* were designed by K.J. Kim (School of Life Sciences and Biotechnology, Korea University, Seoul, Korea; see Fazekas et al. [34]).

Sequence chromatograms were edited and assembled using CodonCode Aligner 3.7 (CodonCode Co. USA), Sequencher 4.7 (Gene Codes Corporation, Ann Arbor, MI, USA) and Geneious version 6.0.3 (created by Biomatters, available from ​http://www.geneious.com/​​). All traces were assembled into contigs and edited manually. Consensus sequences were generated and aligned using MUSCLE [160] as implemented in CodonCode Aligner, Geneious and BOLD. These alignments were examined by eye to detect potential base calling errors, particularly at the beginning and ends of traces. Potential errors were checked in the trace files and corrected as necessary. This process was undertaken multiple times as sequence data were generated and with the final complete data set. 

### Barcode Validation

Barcode data were validated (quality control) iteratively throughout data collection to identify potential contamination, misidentification, and alignment error. As sequence data were generated, neighbour joining trees (phenograms) using a K2P distance model were generated in BOLD for each plastid region, including all taxa in the data set. In these trees we looked for individuals that were grossly misplaced with respect to their correct families or genera. We also generated trees for single families, in which branch lengths were readily visible compared with the compressed branch lengths in multi-family trees, allowing us to more easily identify potential problems. Individuals that did not cluster with other individuals of their species or species-groups were flagged for follow-up. Voucher specimens of all problematic samples were re-examined, misidentifications were corrected, and clear instances of contamination were removed from the project. In genera with low or no variation among species and/or species-groups, identifying potential identification errors based on the neighbour joining trees was difficult or impossible, as there was little or no informative clustering of taxa to guide this process.

When misidentifications were corrected on specimens, taxon names and the name of the most recent identifier were also updated in BOLD. BOLD does not track identification changes, thus in most cases we included brief comments in the "Taxonomy Notes" field on the BOLD specimen page recording that the determination of the specimen had changed; we also noted the previous and current identifications, the date, and the identifier. This information is the only indication to a BOLD user that a determination of a specimen has changed, as we did not re-image the re-determined voucher specimens.

### Genetic Diversity

Infraspecific and interspecific (within-genus) uncorrected *p*-distances were calculated for all pairwise combinations for *rbcL* and *matK* based on unambiguous family-level alignments for each marker using the "Pairwise Summary" function in the program TaxonDNA [161]. The distributions of genetic distances are summarized in a single histogram. 

### Sequence Recoverability

We calculated the number of *rbcL* and *matK* sequences in the entire data set, from samples obtained from herbarium specimens, and from samples obtained from silica-gel dried leaf samples. To determine if herbarium specimen age and sequence recovery are correlated, we divided the herbarium specimens into seven decade-long age classes (and one age class of one year with a single specimen representing the current decade) and counted the number of *rbcL* and *matK* sequences recovered from specimens in each age class. We then used a Spearman rank correlation to test for a relationship between these variables. Spearman rank correlation was calculated in PAST [162]. The *matK* analysis excluded Dryopteridaceae, Equisetaceae, Juncaceae, Polypodiaceae, and Lycopodiaceae, as all samples from these families failed for this marker due to primer mismatch. 

### Barcode Success

We use the terms 'resolved', 'species resolution', 'discriminated', and 'discrimination success' in reference to taxa with unique DNA barcodes in the current data set. To examine species discrimination for the plastid barcodes, we conducted distance-based and sequence-similarity analyses. Distance-based analyses, which are useful for visualizing patterns of genetic variation, were based on neighbour joining trees generated from uncorrected *p*-distances [163,164]. Global matrices were initially aligned using BOLD and the Geneious MUSCLE plugin, and examined in Geneious showing codons to ensure the matrices were in frame; these alignments were adjusted by eye as necessary. The global *rbcL* alignment was straightforward and unambiguous, whereas the *matK* alignment had several problematic regions, primarily due to insertion/deletion events. To eliminate the effects of possible alignment errors in our analyses, we generated separate *rbcL* and *matK* alignments for each family and conducted neighbour joining analyses with these. At the family level, *matK* was straightforward and unambiguous to align. Single-family neighbour joining trees were much easier to score than multi-family trees. Short branch lengths in the latter, particularly within genera and species complexes, were difficult or impossible to see because of high genetic variation among families. 

To determine the discrimination success of a two-locus barcode, the *rbcL* and *matK* matrices for each family were concatenated in Geneious into a single matrix. Individuals lacking data for one of the markers were deleted, resulting in a matrix in which all individuals have data for both loci. Neighbour joining trees were generated independently for *psbA–trnH, psbK–psbI*, and *atpF–atpH*. Six base pair microinversions were present in *psbA–trnH* in 14 *Puccinellia* sequences (BOLD Sample IDs: FCA1084-10, FCA1068-10, FCA1069-10, FCA1070-10, FCA1084-10, FCA1089-10, FCA1090-10, FCA1099-10, FCA1102-10, FCA1105-10, FCA1106-10, FCA1111-10, FCA1118-10, FCA1119-10) and nine *Poa* sequences (FCA1134-10, FCA1122-10, FCA1123-10, FCA1124-10, FCA1125-10, FCA1126-10, FCA1127-10, FCA1146-10, FCA1148-10) at positions 527 to 532 in the alignment. These inversions were reoriented in the matrix so that all sequences had the same inversion state, and these corrected sequences were uploaded to BOLD. 

We scored discrimination in neighbour joining trees by determining the number of genera, species, and infraspecific taxa that could be unambiguously identified in the trees based on their clustering patterns and branch length variation. We did not consider topological monophyly a necessary criterion for taxon discrimination. A genus was scored as discriminated if it shared no identical sequences with other genera. A species was scored as discriminated if its individuals shared no identical sequences with other species. Typically, conspecific individuals clustered together, although in several instances this was not the case. Sometimes, conspecific individuals formed two or more clusters distinct from each other and from all other taxa. Discrimination of infraspecific taxa was scored in the same manner as species when more than one infraspecific taxon was represented in the data sets.

To test species discrimination based on sequence similarity, we conducted BLAST searches following the methods of Burgess et al. [95]. We assembled separate databases of all *matK* and *rbcL* sequences. We did not conduct BLAST searches for combined loci, as multilocus searches cannot be conducted in GenBank or BOLD. For each database we conducted all-to-all BLAST searches, querying each sample to the database using the BLASTN plugin in Geneious. For each BLAST search we scored match success at the genus, species, and infraspecific levels. At the genus level, an individual was scored as discriminated when there were no 100% similarity matches with an individual of another genus, regardless of whether the query sequence was more similar to sequences from another genus than to congeneric sequences, as was sometimes the case (e.g., in non-monophyletic genera such as *Minuartia*). A genus for which all sampled species were correctly assigned was considered discriminated. A species was scored as discriminated if the sequences of all its individuals were distinct from those of all other species. A species was scored as not discriminated if one or more individuals had barcodes identical to those from another species. Species represented by only one individual were scored as discriminated if the individual’s sequence was unique in the database. For species with more than one infraspecific taxon in the database, discrimination success at this level was also scored. An infraspecific taxon was scored as discriminated if all sequences for this taxon differed from those of conspecific infraspecific taxa.

## Results

### Sequence Recoverability

**Figure 2:**
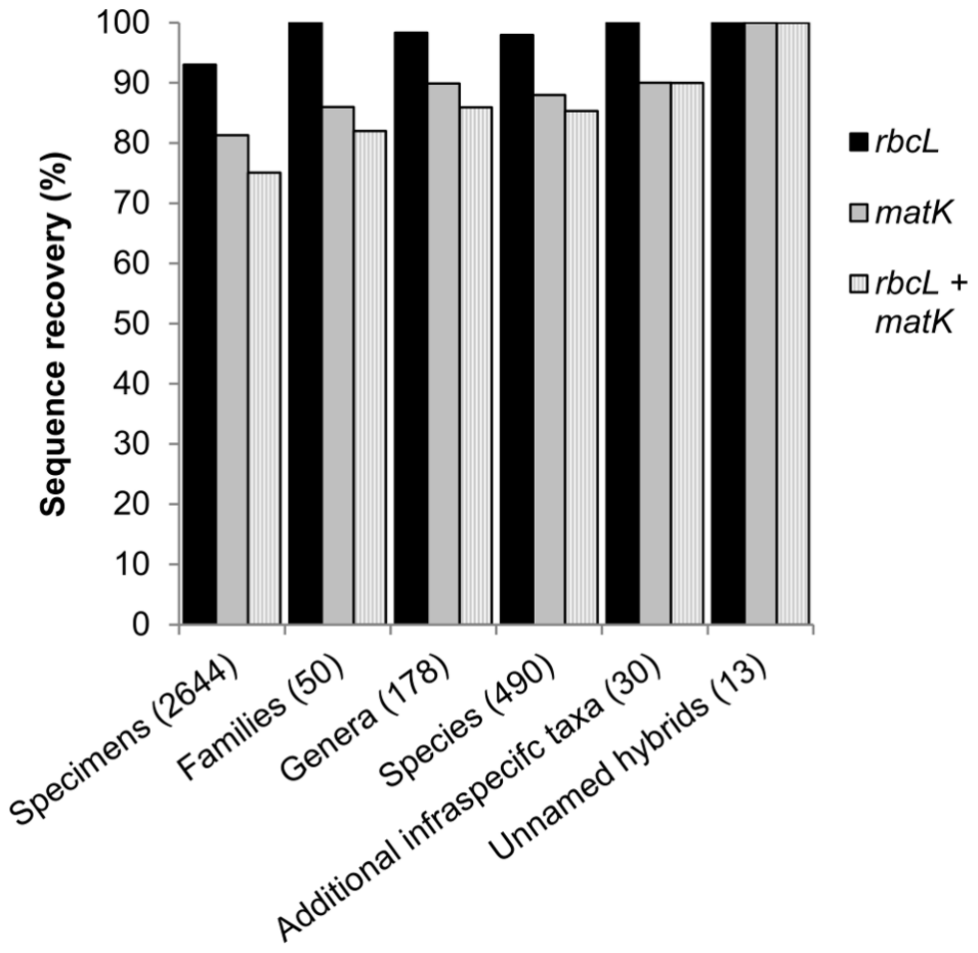
Percentage of specimens, families, genera, species, additional infraspecific taxa, and unnamed hybrids in the data set from which *rbcL* and *matK* barcodes were recovered. Numbers in parentheses are the total number of individuals (specimens, unnamed hybrids) and taxa (families, genera, species, additional infraspecific taxa) in each category in the data set.

For the core barcode loci we obtained 4610 new sequences (2150 and 2460 for *matK* and *rbcL*, respectively) from 2644 specimens representing 50 plant families, 178 genera, 490 species, 30 additional infraspecific taxa, and 13 unnamed putative hybrids. The number of *rbcL* and *matK* sequences recovered per plant family ranged from 1–498 (mean = 49.2 ± 88.0) and 0-486 (mean = 43 ± 84.6), respectively (Figure S2). Both markers were recovered from more than 100 individuals in Poaceae, Cyperaceae, Brassicaceae, Asteraceae, Caryophyllaceae, Saxifragaceae, Salicaceae, and Rosaceae (listed in descending order by number of individuals sampled; Figure S2). Sequence recoverability was significantly higher for *rbcL* than *matK* (93% and 81.3% of specimens, respectively; two sample Z-test, Z = 12.7, p = 0; Figure 2). Combined *rbcL* + *matK* sequence data were obtained from 75% of the specimens sampled (Figure 2). *rbcL* sequences were recovered from more families, genera, and species than were *matK* sequences. We obtained *rbcL* data for 100% of the 50 families sampled, *matK* for 86%, and *rbcL* + *matK* for 82%; we obtained *rbcL* data for 98% of the 178 genera sampled, *matK* for 90%, and *rbcL* + *matK* for 86%; we obtained *rbcL* data for 98% of the 490 species sampled, *matK* for 88%, and *rbcL* + *matK* sequences for 8% (Figure 2). We did not recover any *matK* sequences in Pinales (Cupressaceae, Pinaceae), monilophytes (Dryopteridaceae, Equisetaceae, Lycopodiaceae, Ophioglossaceae, Polypodiaceae), or one monocot family (Juncaceae) (Figure S2). 

**Figure 3:**
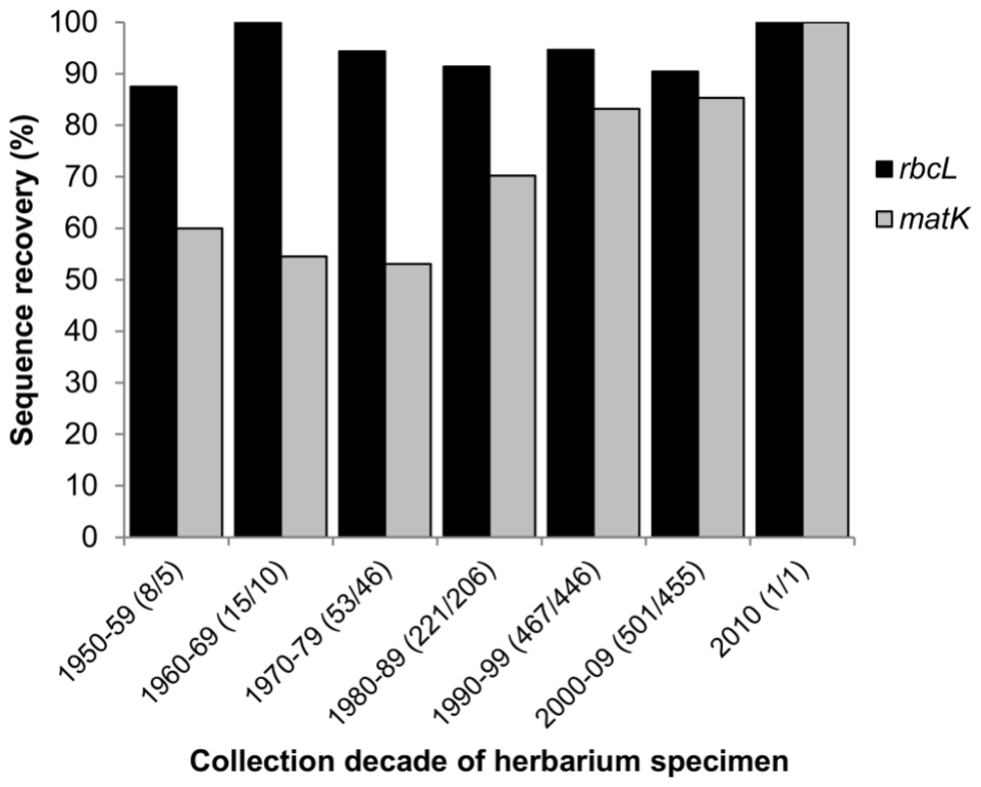
Relationship between herbarium specimen age and sequence recovery (%) for *rbcL* and *matK*. Material was sampled from 1169 herbarium specimens collected between 1950–2010. These were divided into seven age classes and a Spearman rank correlation was used to test for a relationship between age class and percent recovery of *rbcL* (Spearman's rho 0.306, p = 0.50079) and *matK* (Spearman's rho 0.893, p = 0.012302). The *matK* analysis excluded Dryopteridaceae, Equisetaceae, Juncaceae, Polypodiaceae, and Lycopodiaceae, which failed for all samples for this marker due to primer mismatch. Numbers in parentheses are the total number of herbarium specimens sampled from each age class for *rbcL* and *matK*, respectively.

There was no difference in *rbcL* recovery from herbarium specimens or silica-gel dried material (92.3% for herbarium specimens and 93.1% for silica-gel dried samples; two sample Z-test, Z = -0.841, p = 0.20045). In contrast, *matK* recovery differed significantly for source materials (76.5% for herbarium specimens and 85.1% for silica-gel dried samples; two sample Z-test, Z = -5.11, p = 0). Excluding monilophyte, lycophyte, and conifer taxa (all of which failed for *matK* regardless of sample source due to primer mismatch), *matK* was recovered from 80.2% of herbarium specimens sampled and 84.9% of silica-gel dried samples (two sample Z-test, Z = -5.678, p = 0). Age of herbarium specimen and recovery of *matK* were significantly correlated (Spearman's rank correlation, p < 0.05, Figure 3), whereas there was no relationship between these variables for *rbcL* (Spearman's rank correlation, p > 0.05, Figure 3). 

### Genetic Diversity

**Figure 4:**
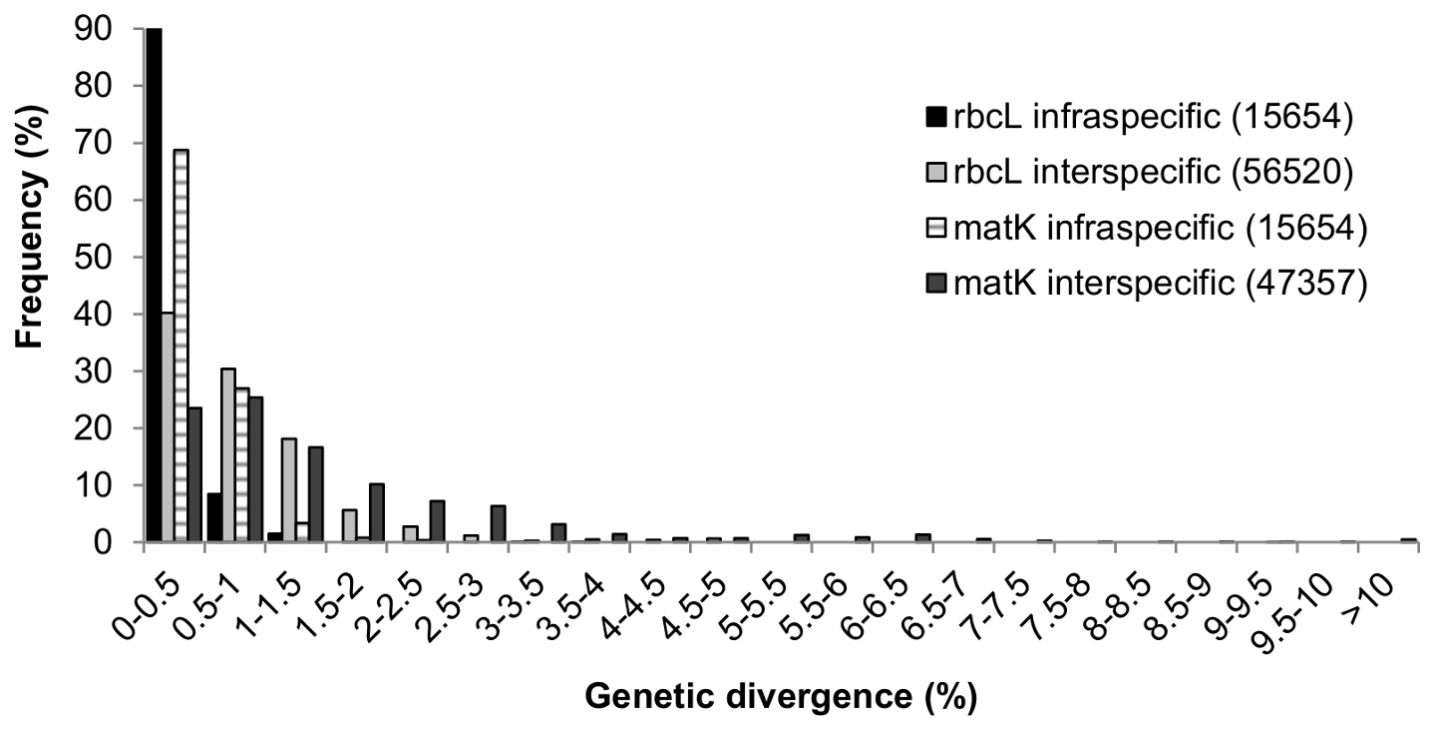
Frequency distribution of infraspecific and congeneric interspecific genetic divergences of *rbcL* and *matK*. Numbers in parentheses are the total number of comparisons for each category. Divergences were calculated using uncorrected *p*-distances.

Based on genetic divergence within and among congeneric species, *matK* is ca. three times more divergent than *rbcL* at the infraspecific level (mean pairwise divergence: matK = 0.000905 ± 0.002226, rbcL = 0.000299 ± 0.001327) and ca. 2.6 times more divergent than *rbcL* at the interspecific level (mean pairwise divergence: *matK* = 0.010686 0.01579, rbcL = 0.00404 ± 0.006439). A few *matK* interspecific pairwise comparisons differ by more than 10% (Figure 4). Frequency distribution of intra- and interspecific divergences showed that ranges of genetic distances within species and among congeneric species are considerably greater for *matK* than for *rbcL* (Figure 4). For both markers there was considerable overlap between infraspecific and interspecific genetic distances.

### Barcode Success

**Figure 5:**
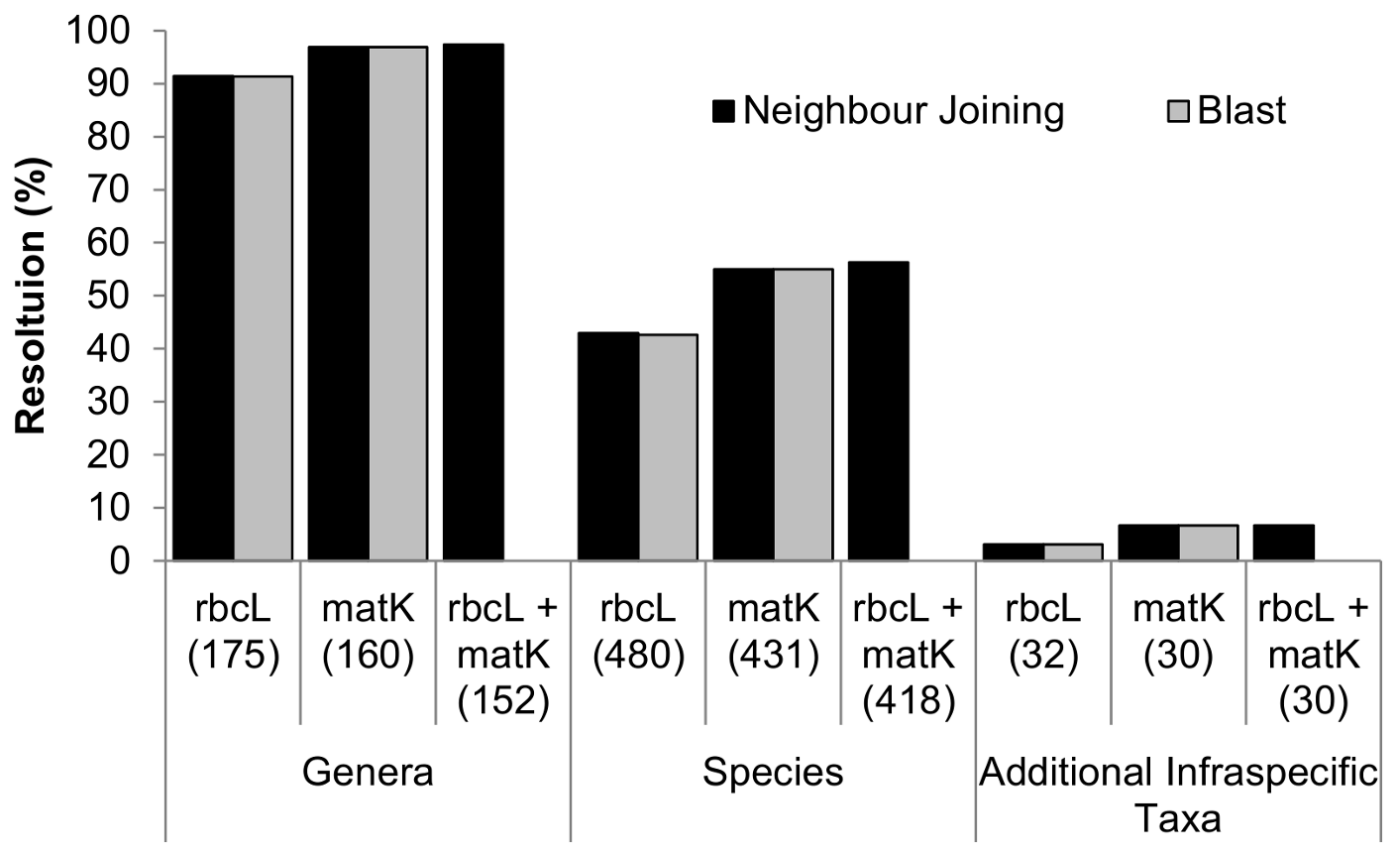
Resolution (%) of genera, species, and additional infraspecific taxa for *rbcL*, *matK*, and *rbcL* + *matK* in neighbour joining trees and BLAST searches. Neighbour joining trees were generated from single-family alignments using uncorrected *p*-distances. Numbers in parentheses are the numbers of genera or species sampled in each data set. BLAST searches were not conducted for the combined *rbcL* + *matK* data.

We determined percent species resolution based on BLAST searches and neighbour joining trees generated from uncorrected *p*-distances calculated from single-family alignments. Neighbour joining trees for *rbcL*, *matK*, and *rbcL* + *matK* for each family are presented in Figures S3–S47; trees are not presented for Araceae, Ophioglossaceae, and Santalaceae, as only one or two individuals in these families were sampled. For both BLAST and neighbour joining analyses, a species was considered resolved if all members had barcodes that differed from all individuals of other species. Levels of discrimination were identical or nearly identical for BLAST searches and neighbour joining trees. In both BLAST and neighbour joining analyses, over 90% of sampled species could be assigned to their genus by *rbcL* (91.4%), *matK* (96.9%), or *rbcL* + *matK* (97.4%) (Figure 5). Discrimination of species was 42.6–42.9% based on *rbcL*, 55% based on *matK*, and 56.3% based on *rbcL* + *matK* (Figure 5). Discrimination of infraspecific taxa was low for all markers, ranging from 3.1% for *rbcL* to 6.7% for *matK* and *rbcL* + *matK* (Figure 5).

**Figure 6:**
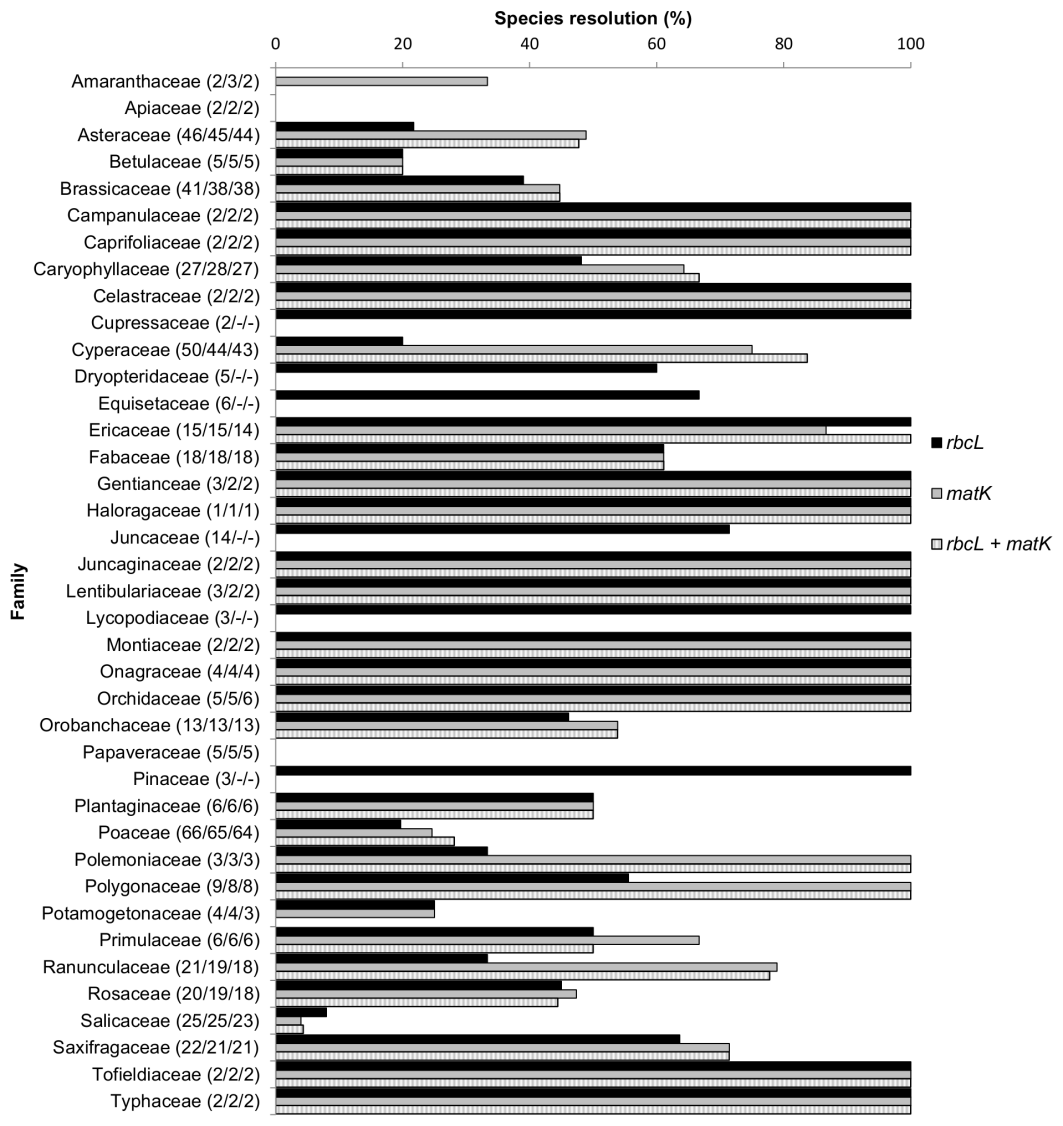
Species resolution (%) per family for *rbcL*, *matK*, and *rbcL* + *matK*. Numbers in parentheses refer to the numbers of species for which barcode data were recovered for *rbcL*, *matK*, and *rbcL* + *matK*, respectively. Families with a single genus and species sampled are excluded. Dashes (-) indicate that no sequences were recovered.

**Figure 7:**
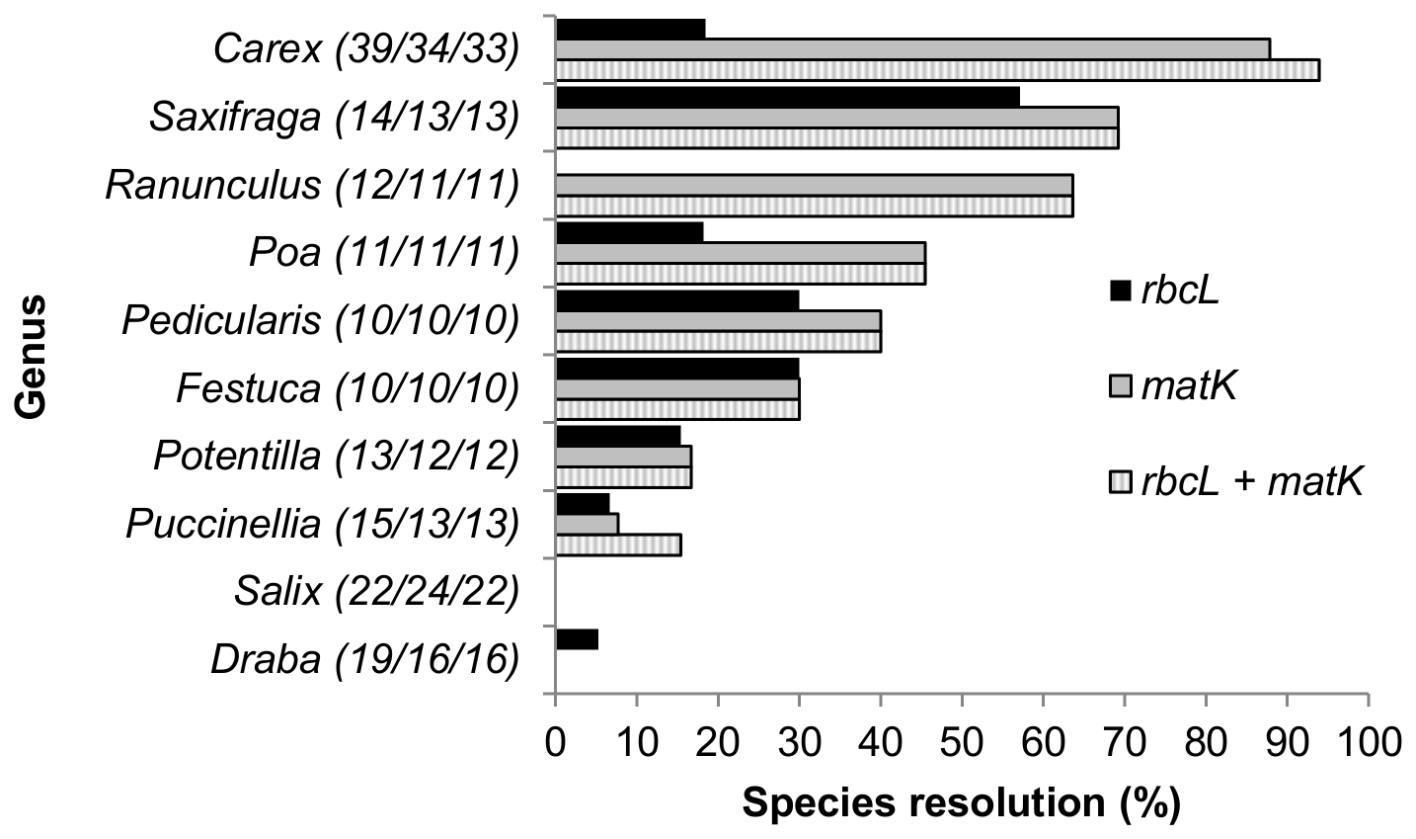
Species resolution (%) in ten genera with the greatest number of species sampled. Numbers in parentheses refer to the number of species sampled for *rbcL*, *matK*, and *rbcL* + *matK*, respectively.

**Figure 8:**
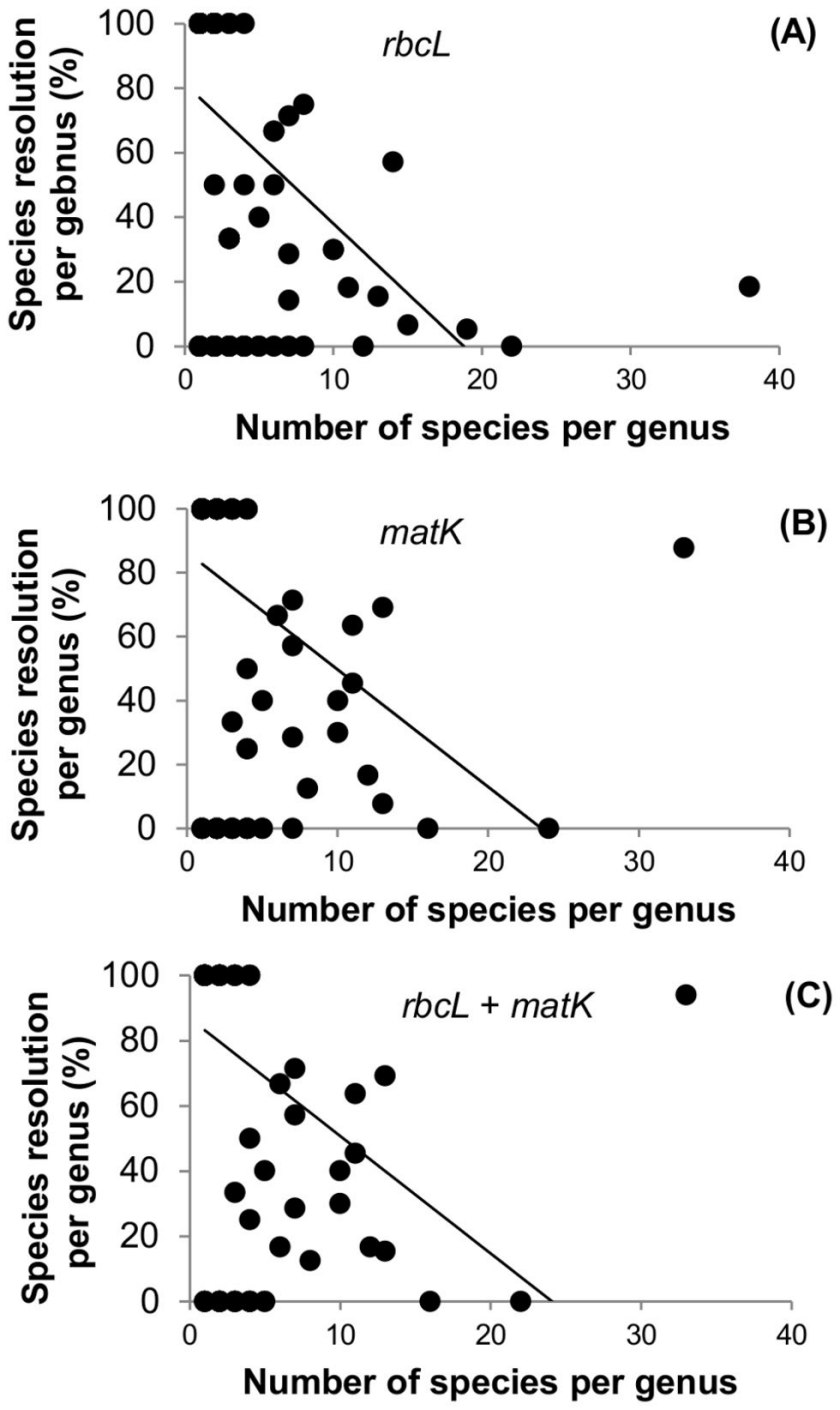
Scatterplots of the number of species sampled in each genus against the percentage of species resolved in each genus with *rbcL*, *matK*, and *rbcL* + *matK*. A. rbcL-175 genera (Pearson correlation coefficient r = 0.4180, n = 175, P < 0.0001), R^2^ = 0.1747. B. matK-159 genera (Pearson correlation coefficient r = 0.3685, n = 159, P < 0.0001), R^2^ = 0.1358. C. rbcL + matK-153 genera (Pearson correlation coefficient r = 0.3636, n = 153, P < 0.0001), R^2^ = 0.1322. Species resolution was scored in neighbour joining trees generated from uncorrected *p*-distances calculated from single-family alignments.

At the family level, species resolution ranged from 0% to 100% (Figure 6). Resolution of species with *rbcL* + *matK* in nine families from which 20 or more species were sampled (Poaceae, Cyperaceae, Asteraceae, Brassicaceae, Caryophyllaceae, Salicaceae, Saxifragaceae, Rosaceae, Ranunculaceae; listed in descending order by number of species sampled) ranged from 4% (Salicaceae) to 84% (Cyperaceae) (Figure 6). In 29 genera the *rbcL* + *matK* barcode provided 100% species resolution; in these genera two to four species were sampled (Table 1). By contrast, in 21 genera with two or more species sampled for the two-locus barcode there was 0% species resolution (Table 2). In four genera, one species per genus was sampled and none were resolved (Table 2); these taxa are not distinct at the genus level. Species resolution in the ten genera with ten or more sampled species (*Carex*, *Draba*, *Festuca*, *Pedicularis*, *Poa*, *Potentilla*, *Puccinellia*, *Ranunculus* L.*, Salix, Saxifraga* L.) ranged from 0% to 94%; in seven of these less than 50% of species were resolved (Figure 7). For both individual and combined data sets, there was a significant negative correlation between the number of species sampled per genus and the percent species resolution per genus (Figure 8). 

**Table 1:** Genera in which all species are resolved by *rbcL*, *matK*, and/or *rbcL* + *matK*.

**Family**	**Genus**	**Number of species sampled/resolved**		
		***rbcL***	***matK***	***rbcL* + *matK***
Asteraceae	*Arnica* Rupp. ex L.	2/0	2/2	2/2
	*Artemisia* L.	3/1	3/3	3/3
Brassicaceae	*Arabidopsis* (DC.) Heynh.	2/2	2/2	2/2
	*Cardamine* L.	4/4	4/4	4/4
	*Parrya* R. Br.	2/2	2/2	2/2
Campanulaceae	*Campanula* L.	2/2	2/2	2/2
Caryophyllaceae	*Arenaria* L.	2/0	2/2	2/2
	*Sagina* L.	2/2	2/2	2/2
	*Stellaria* L.	3/1	3/3	3/3
Cyperaceae	*Parnassia* L.	2/2	2/2	2/2
	*Kobresia* Willd.	3/2	3/3	3/3
Ericaceae	*Arctous* (A. Gray) Nied.	2/2	2/0	–
	*Rhododendron* L.	2/2	2/2	2/2
	*Vaccinium* L.	2/2	2/2	2/2
Fabaceae	*Hedysarum* L.	2/2	2/2	2/2
Juncaginaceae	*Triglochin* L.	2/2	2/2	2/2
Utriculariaceae	*Pinguicula* L.	2/2	2/2	2/2
Onagraceae	*Chamerion* (Raf.) Raf. ex Holub	2/2	2/2	2/2
	*Epilobium* L.	2/2	2/2	2/2
Orchidaceae	*Platanthera* Rich.	3/1	3/3	3/3
	*Plantago* L.	2/2	2/2	2/2
Polemoniaceae	*Phlox* L.	2/0	2/2	2/2
Polygonaceae	*Bistorta* (L.) Scop.	2/2	2/2	2/2
	*Rumex* L.	4/0	4/4	4/4
Primulaceae	*Androsace* L.	2/2	2/2	2/2
Ranunculaceae	*Anemone* L.	3/3	3/3	3/3
Rosaceae	*Rubus* L.	2/2	2/2	2/2
Salicaceae	*Populus* L.	2/2	–	–
Saxifragaceae	*Chrysosplenium* L.	3/3	3/1	3/3
Tofieldiaceae	*Tofieldia* Huds.	2/2	2/2	2/2
Typhaceae	*Sparganium* L.	2/2	2/2	2/2

A dash (-) indicates that only one species was sampled for a locus or the combined loci.

**Table 2:** Genera in which no sampled species are resolved by *rbcL*, *matK*, and/or *rbcL* + *matK*.

**Family**	**Genus**	**Number of species sampled/resolved**		
		***rbcL***	***matK***	***rbcL* + *matK***
Amaranthaceae	*Suaeda* Forssk. ex J.F. Gmel.	2/0	2/0	2/0
Apiaceae	*Bupleurum* L.	2/0	2/0	2/0
Asteraceae	*Antennaria* Gaertn.	6/0	4/0	4/0
	*Arctanthemum* (Tzvelev) Tzvelev	1/0	1/1	1/1
	*Arnica* Rupp. ex L.	2/0	2/2	2/2
	*Eurybia* (Cass.) Gray	1/0	1/1	1/1
	*Hulteniella* Tzvelev	1/0	1/1	1/1
	*Petasites* Mill.	2/2	2/0	2/0
	*Saussurea* DC.	2/0	2/0	2/0
	*Solidago* L.	2/0	2/0	2/0
	*Symphyotrichum* Nees	1/0	1/1	1/1
	*Taraxacum* F.H. Wigg.	8/0	8/1	8/1
	*Tephroseris* (Rchb.) Rchb.	3/0	3/0	3/0
Betulaceae	*Betula* L.	4/0	4/0	4/0
Brassicaceae	*Braya* Sternb. & Hoppe	4/0	4/1	4/1
	*Draba* L.	18/1	16/0	16/0
	*Erysimum* L.	3/0	2/0	2/0
	*Transberingia* Al-Shehbaz & O'Kane	1/0	1/1	1/1
Caryophyllaceae	*Arenaria* L.	2/0	2/2	2/2
	*Cerastium* L.	4/0	4/0	4/0
Cupressaceae	*Juniperus* L.	2/0	2/2	2/2
Cyperaceae	*Eriophorum* L.	7/0	7/0	7/1
Papaveraceae	*Papaver* L.	5/0	5/0	5/0
Plantaginaceae	*Hippuris* L.	3/0	3/0	3/0
Poaceae	*Agrostis* L.	2/0	2/0	2/0
	*Anthoxanthum* L.	3/0	4/1	4/1
	*Arctagrostis* Griseb.	1/1	1/0	1/1
	*Arctophila* (Rupr.) Andersson	1/0	1/0	1/0
	*Calamagrostis* Adans.	5/0	5/0	5/0
	*Deschampsia* P. Beauv.	3/0	3/0	3/0
	*Dupontia* R. Br.	1/0	1/0	1/0
	*Elymus* L.	4/0	4/0	4/0
	*Koeleria* Pers.	1/0	1/0	1/0
	*Phippsia* (Trin.) R. Br.	2/0	2/0	2/0
	*Trisetum* Pers.	1/0	1/0	1/0
Polemoniaceae	*Phlox* L.	2/0	2/2	2/2
Polygonaceae	*Rumex* L.	4/0	4/4	4/4
Potamogetonaceae	*Stuckenia* Börner	3/0	3/1	3/0
Primulaceae	*Primula* L.	3/0	3/0	3/0
Ranunculaceae	*Coptidium* (Nyman) Tzvelev	2/0	1/1	–
	*Ranunculus* L.	12/0	11/7	11/7
Salicaceae	*Salix* L.	22/0	24/0	22/0

A dash (-) indicates no species were sampled for the combined loci.

Thirty species from 11 families include more than one infraspecific taxon (51 infraspecific taxa in total; Table S1, Data Set S1). Five infraspecific taxa were distinguished with the barcode data: *Oxytropis borealis* DC. var. *Borealis*, *O. Borealis* var. *viscida* (Nutt.) S.L. Welsh, *Eriophorum scheuchzeri* Hoppe subsp. *scheuchzeri*, *E. scheuchzeri* subsp. *arcticum* M.S. Novos., and *Stuckenia filiformis* (Pers.) Börner subsp. *filiformis* (Table S1). 

The supplementary plastid loci *psbA–trnH, atpF–atpH*, and *psbK–psbI* were sequenced for a subset of *Poa* species and additional infraspecific taxa and *Puccinellia* species (Data Set S1, Figures S49, S50, S51). *psbA–trnH* discriminated 50% of *Poa* species, 38% of *Poa* taxa (i.e., species and additional infraspecific taxa), and no *Puccinellia* species; *psbK–psbI* discriminated 17% of *Poa* species, 10% of *Poa* taxa, and no *Puccinellia* species; and *atpF–atpH* discriminated 33% of *Poa* species, 20% of *Poa* taxa, and 44% of *Puccinellia* species (Table S3). 

## Discussion

### Sequence Recoverability

A key criterion for a standard land plant barcode is universality, meaning that the DNA barcode should be easily recovered from all land plants, ideally with a single primer pair [52]. Our amplification and sequencing success was greater for *rbcL* than *matK*, consistent with the results of numerous other studies that sampled broadly across land plants (e.g., [52,58,94,95,109]). Recovery of *rbcL* was high (93.1%), similar to the results of other studies focused primarily on angiosperms in which *rbcL* recovery ranged from 90–100% [34,52,58,79,95,109,165]. The single pair of *rbcL* primers we used worked in gymnosperms, lycophytes, monilophytes, and angiosperms, as other studies have also found (e.g., [34]). Although other primers have been used to recover *rbcL* from ferns [87,96] and gymnosperms in DNA barcoding studies (e.g., [166]), a single set of primers that will amplify *rbcL* across land plants is of great practical use, facilitating recovery of the region from any unknown sample. In angiosperms, *rbcL* was recovered from an equal or greater number of samples per family than *matK*, except in Boraginaceae, in which *rbcL* was recovered from just one of 11 samples, which all worked for *matK* (Figure S2). The reasons for this high *rbcL* failure in Boraginaceae are unclear. 

In contrast to *rbcL*, recoverability of *matK* differed substantially among the major land plant lineages. No *matK* sequences were recovered from gymnosperm, lycophyte, and monilophyte taxa (Figure S2). These results are similar to previous studies, which have found that *matK* primers used in angiosperms generally work poorly in gymnosperms [45,52,98,167–169]. *matK* has been successfully recovered in gymnosperms using taxon-specific primers [34,61], and a new set of *matK* primers with high PCR universality, high sequence quality, and high coverage across gymnosperms has been recently recommended for barcoding gymnosperms [167]. 

Previous studies have similarly not been successful in recovering *matK* from ferns with the universal primers widely used in DNA barcoding studies [37,96,109]. These *matK* primers do not work because of the loss of *trnK* and its intron in most leptosporangiate ferns, including the *trnK* exons in which the priming sites for the angiosperm-designed primers are located [170-172]. Among the fern taxa sampled here, *Botrychium* Sw. (Ophioglossaceae) and *Equisetum* L. (Equisetaceae) are non-leptosporangiate genera [113] and have *trnK* genes, yet the universal primers still did not amplify *matK* for these taxa, likely reflecting variation in the priming sites. Acknowledging the difficulty of amplifying *matK* in ferns, DNA barcode data have been generated for regional fern floras using combinations of *rbcL* and *trnH*–*psbA* [96] and *rbcL* and *trnL*–*F* [87], excluding *matK* from consideration. Nevertheless, *matK* has recently been demonstrated to be variable and useful as a DNA barcode for ferns in studies using primers specific to particular genera or lineages [172,173]. These should be tested for Arctic ferns. 

Among angiosperms we recovered *matK* sequences from all families except Juncaceae (Figure S2). Results of other studies confirm that sequencing *matK* is problematic in this family with the primers we used. For example, *matK* failed for nearly all Juncaceae taxa sampled from Churchill, being successful in just two of 33 samples [109], and Burgess et al. [95] obtained sequence data for only two of five sampled Juncaceae taxa from southern Canada. Alternate *matK* primers have provided better results: de Vere et al. [98] amplified and sequenced some 40 of 76 sampled Juncaceae taxa using primers they designed for Poales, though still had a nearly 50% failure rate, whereas Schaefer et al. [174] sequenced nine sampled *Juncus* taxa using *matK* primers they newly designed. These latter two sets of primers should be tested across the family.

Recovery of *matK* ranged considerably in the other angiosperm families. Considering only specimens sequenced from silica-gel dried leaf material (from which sequence recovery is not expected to be affected by sample age) and 14 families with more than 15 individuals sampled (not including Juncaceae), *matK* failure was less than 10% in ten families, but considerably higher in the remainder (Table S2). The greatest failure to recover *matK* occurred in Saxifragaceae (46.8% failure), Ranunculaceae (26.1%), Polygonaceae (22.7%), and Cyperaceae (22.6%) (Table S2). In all of these families there was no clear pattern to the failures: in most cases, some individuals of a species failed while other individuals of the same species were successful; an exception to this is *Saxifraga oppositifolia* L., from which we recovered *matK* from just one of ten samples. These results are generally similar to those of de Vere et al. [98], who found recovery of *matK* from fresh material to be low to moderate in Saxifragales, Ranunculales, and Poales, the orders in which Saxifragaceae, Ranunculaceae, and Cyperaceae are classified. Increased *matK* failure in these groups may be a primer design issue. In contrast to our inability to recover *matK* barcodes from 1/5 of our Cyperaceae samples, two studies that generated large amounts of barcode data for Cyperaceae did not report any major problems recovering *matK* [108,175]. These studies used different primers for this gene (*matK* 2.1af, *matK*–2.1f, *matK*–5r; Royal Botanic Gardens, Kew, www.kew.org/barcoding), which may be better than the primers we used to recover *matK* in Cyperaceae, particularly in *Carex* and *Kobresia*. Given the variability in *matK* across land plants—the property that makes it useful as a DNA barcode—it is now recognized that a single universal primer pair that will amplify *matK* in all taxa is likely not realistic [37,176]. Accordingly, new order-specific primers have been designed for *matK* across angiosperms that increase its recovery significantly [176]. *matK* primers that have been used in phylogenetic studies are available for Saxifragaceae, as well (e.g., [177–179]). 

Over half of the barcodes in our study were recovered from silica-gel dried material collected fresh in the field, and recovery of *rbcL* and *matK* sequences from this material was generally quite high (93.1% and 85.1%, respectively). Such recently-collected material, when available, is desirable to work with as it generally performs well in the laboratory, and does not require destructive sampling of existing herbarium collections. Unfortunately, obtaining fresh plant collections from most Arctic areas requires substantial logistical planning and financial resources. Sampling existing herbarium material allowed us to nearly double the number of specimens sampled in our study, expanding considerably our sampling both taxonomically and geographically. Forty-four percent of the DNA extracts we used were obtained from herbarium specimens, collected from 1950 to the present. We found no differences in recoverability of *rbcL* when sampled from silica-gel dried (93.1%) or herbarium material (92.7%), and the ages of the herbarium specimens we sampled did not affect *rbcL* recoverability, as in a related study of Arctic plants [109]. This result is not unexpected, as our selection of herbarium material was not random. We chose herbarium specimens collected recently and with material that appeared to have dried rapidly, which likely contributed to the high success of *rbcL* recovery. By contrast, recoverability of *rbcL* from herbarium specimens documenting the Welsh flora was 14% lower than from fresh material [98]. Recovery of *rbcL* from herbarium material in that study may have been lower than observed here because they included material up to nearly a century older (as old as 1868) than did we (as old as 1950). 

In contrast to our results for *rbcL*, we found recovery of *matK* to be significantly lower (76.5%) from herbarium specimens than from silica-gel dried material (85.1%), and the age of herbarium specimens did have a significant effect on *matK* recoverability. As the age of the herbarium specimens increased, we failed to recover *matK* from an increasingly greater proportion of the sampled specimens (Figure 3), as in de Vere et al. [98]. Over 85% of the specimens collected in the last two decades yielded *matK* sequences, similar to the barcoding study of the Welsh flora, in which *matK* was recovered from 70% and 80% of specimens collected in each of the last two decades, respectively. Lower recovery of *matK* from herbarium specimens has been attributed to the longer length of this region (ca. 800 bp), making it more difficult to amplify from degraded DNA, compared to *rbcL* (552 bp) [109]. Differences in sequence recovery from herbarium specimens among studies is not unexpected, and may reflect differences in local storage conditions of herbarium specimens sampled, the rate of initial drying of the specimens in the field, or the primers used to amplify the gene regions.

Despite an increased number of amplification and sequencing problems with herbarium specimens, they are a critically important source of material for barcoding plants. In general, efforts to generate DNA barcodes from herbarium material should focus on the most recently collected specimens available to maximize successful barcode recovery [98], as our results demonstrate for *matK*. Identifications of herbarium material being used to generate barcode data should always be confirmed. We found nomenclature on many herbarium specimens that required updating to reflect current taxonomy, and occasionally we encountered herbarium specimens that had more than one species mounted on a sheet (i.e., mixed collections), particularly in genera with morphologically similar species such as *Carex* and *Draba*. Mixed collections often became evident only upon re-examining the herbarium sheet of a putatively misplaced individual in a neighbour joining tree, and realizing that the sequence of the suspicious individual came from a second species on the sheet—the one from which leaf material was obtained—which had not been noticed previously. We now routinely mark the individual on a herbarium sheet from which leaf material is removed with a small arrow so it is clear from which plant the barcode data were recovered. 

### Genus and Species Resolution

The primary goal of DNA barcoding is to assign unknown individuals to known species by matching their sequences with those of known species in a reference library. Multiple approaches have been explored for evaluating the success of plant barcoding markers for identifying species, including phenetic analyses based on distance measures, phylogenetic methods using parsimony, maximum likelihood and Bayesian criteria, comparisons of inter- and infraspecific genetic distances, sequence similarity analyses using Basic Local Alignment Search Tool (BLAST) searches or other algorithms, and character-based approaches (e.g., [161,180–183]). Unfortunately there is no consensus in the plant barcoding literature, or the barcoding literature more broadly, on criteria for unambiguously discriminating taxa with barcode data. At present, the plant identification tool in BOLD uses the BLAST algorithm and accepts *rbcL* and *matK* sequences, although it does not accept both simultaneously.

We considered a species to be successfully discriminated when all individuals of a species had barcode sequences not shared by any other species in the data set; we did not apply a bootstrap threshold, we did not require an arbitrary minimum level of genetic variation among species, and we did not require all individuals of a species to cluster together, as we were not testing species monophyly. The latter is problematic with the phenetic approaches used here and reliance on plastid data alone, which represents a single linkage group and does not necessarily reflect organismal phylogeny. Fazekas et al. [34] provide a critical discussion of monophyly as a criterion for determining barcoding success. 

Because we have allowed the smallest possible sequence differences (one nucleotide) to discriminate species, we consider the level of resolution obtained here to be at or near the upper limit for the Canadian Arctic flora for the core plant barcoding markers. Application of bootstrap support or genetic variation thresholds would likely result in considerably decreased resolution, given the few nucleotide differences that distinguish many species. A practical limitation of our approach is that an unknown individual with a barcode haplotype not represented in the database for its species may not be assignable to its species if it does not cluster with other conspecific individuals in a neighbour joining tree, or if it does not find a 100% match in a BLAST search. This is an unfortunate reflection of low variation in the core plastid barcode loci among closely related plant species. Extensive sampling from throughout species' geographic ranges will be needed to maximize the probability that all barcode haplotypes for a species are sampled and represented in the barcode reference library. We certainly have not detected the full range of variation in the core barcode loci in most of the nearly 500 species sampled here, considering we sampled only six or fewer individuals per species in over half the species in the data set, and just one individual in 120 of the species studied (Figure S1). Further, we have not sampled any individuals from beyond the northern North American portions of most of the sampled species' ranges, which in most cases are considerably broader, variously extending into southern Canada and the U.S.A., other global Arctic regions to the east and west, and even into the southern hemisphere [20]. 

#### Genus Resolution

Multiple studies have demonstrated that the core barcode loci routinely provide high discrimination at the genus level, usually greater than 90% (e.g., [94,98]). Accordingly, we find that *rbcL* distinguishes 91.4% of genera, and *matK* and *rbcL* + *matK* distinguish 97% of genera in our data set. Considering *matK* and the combined loci, just four genera—all members of Poaceae—cannot be distinguished. Two of these, *Dupontia* R.Br. (a genus of one polymorphic species [184,185]) and *Arctophila* Rupr. (monotypic), are distinct from all other genera, but share identical *rbcL* and *matK* barcodes. *Dupontia* and *Arctophila* are closely related and form a strongly supported clade in phylogenetic analyses; they are distinguished from each other weakly, based on plastid *trnT*–*F* data [186,187], or not at all, based on *trnL* [184] and ITS data [184,186]. *Dupontia* may have an intergeneric hybrid origin, with one parent being *Arctophila fulva* (Trin.) Andersson and the other parent unknown [184]. The other two unresolved genera are also represented by one species each (*Koeleria macrantha* (Ledeb.) Schult. and *Trisetum spicatum* (L.) K. Richt.), which have identical *rbcL* and *matK* barcodes. These results are consistent with the findings of previous phylogenetic work, in which species of *Koeleria* Pers., *Trisetum* Pers. (including *T. spicatum*), and other (non-Arctic) genera were intermixed—a group of taxa in which generic boundaries are not clear [188,189]. 

#### Species Resolution

The core plastid DNA barcode markers, when combined, discriminate a maximum of 56% of the species sampled here. This is a considerable improvement over resolution with *rbcL* alone (42.6–42.9%) and a slight improvement over *matK* alone (55% resolution). Although in a few cases *rbcL* discriminates species that have identical *matK* barcodes, discrimination with the combined loci is not considerably greater than with *matK* alone because the discrimination method we used is based on minimum differences between species. Adding distinguishing characters from a second locus does not increase the resolution if the species is already discriminated by the first locus. However, the increased variation in the two-locus barcode is taken into account in other methods for characterizing species discrimination (listed above).

Since the recommendation of *rbcL* and *matK* as core DNA barcoding loci in 2009 [52], several large plant barcoding studies have included these markers (Table 3), allowing comparisons of the performance of these loci across a diversity of taxa and floristic regions. A caveat to such comparisons is that different studies often use different criteria for species discrimination. Species discrimination for the Canadian Arctic flora with *rbcL* + *matK* is nearly identical to the 54% of 286 species discriminated with these markers for the flora of Churchill, Manitoba [109]. This is not unexpected, as Churchill is located along the southern boundary of the Canadian Arctic ecozone and many of the same species are sampled in both studies. However, given the smaller number of species in the Churchill study, it may be expected that discrimination would be greater in that local flora compared with the broader Arctic flora considered here. Kuzmina et al. [109] used a criterion of monophyly to score species discrimination, a more conservative method than the one we used. Species resolution in the Churchill data set may be greater if the discrimination methods used here were applied. Our results are also similar to those of a larger study in which *rbcL* + *matK* discriminated 49.7% of 765 species sampled mostly from China [58]. 

**Table 3:** Species resolution (%) with *rbcL* + *matK* among floristic studies that sampled multiple families, genera, and species at country and local scales.

**Number of genera/ species in study (number of species analysed for *rbcL* + *matK*)**	**Geographical Scale: Sampling Region**	**Species resolution with *rbcL* + *matK***	**Reference**
455/1143 (808)	Country: Wales	69.4–74.9%	[98]
269/436 (282)	Local: Koffler Scientific Reserve, Ontario, Canada	93.1%	[95]
147/354 (not given)	Local: Churchill, Manitoba	54%	[109]
141/1757 (765)	Country: China	49.7%	[58]
181/296 (205)	Local: tropical forest plots, Panama	92%	[94]

A range is given when percent resolution differed among scoring methods.

Species resolution in the Canadian Arctic with *rbcL + matK* is considerably lower than discrimination rates of 70% or greater reported in other floristic studies for the same two-locus barcode (Table 3). *rbcL* + *matK* have provided the highest species resolution in floristic studies that considered only species found in highly restricted areas—a 5 km^2^ forest plot [94] and a 3.5 km^2^ field station [95]—limiting the total number of species overall and, more critically, the number of closely related species in the data set. Similarly, discrimination with *rbcL* + *matK* in the Welsh flora increased as spatial scales of decreasing size were considered, from up to 74.9% at the country level to 81.6% and 93.3% considering 10 km^2^ and 2 km^2^ plots, respectively [98]. These geographically restricted floristic barcoding studies demonstrate the power and utility of the core plant DNA barcoding loci for species discrimination in well-defined local regions, based on reference barcode libraries developed explicitly to support research in those regions. The utility of a local barcode reference library for addressing local ecological questions was demonstrated by Kesanakurti et al. [99], who used a DNA barcode library generated for the flora of a field station to identify roots and characterize below-ground plant diversity at sites in the field station. However, there is not always increased species resolution at smaller geographical scales, as the Churchill study may demonstrate. The barcoding studies of the Welsh flora and Chinese plants each considered a similar number of species and used a similar metric to score species discrimination, yet resolution was considerably higher for the flora of Wales for *rbcL* + *matK* (74%) compared with the resolution for Chinese plants with these same markers (49.7%). This likely reflects differences in the number of species sampled per genus, which was considerably higher in the study of Chinese plants [98]. 

As we sampled fairly extensively across many families, our results can be compared with taxon-specific barcoding studies of particular families and genera. Pang et al. [190] demonstrated very high species discrimination (93–96%) with *rbcL* and *matK* among ca. 70 species in Rosaceae from ca. 22 genera, but no more than one or a few species in most genera were included. Our Rosaceae sampling for *rbcL* + *matK* includes six genera and 18 species (Figure S43), of which just 44% are resolved. In four of these six genera (*Comarum* L., *Dasiphora* Raf., *Dryas* L., *Sibbaldia* L.) we sampled a single species and in *Rubus* L. we sampled two distantly related species [191]. These six species are all distinguished by the barcode data, whereas the remainder of our sampling (12 species) was in *Potentilla*, in which resolution is poor (see discussion of *Potentilla* below). A study evaluating barcode markers in Asteraceae similarly found high resolution with *rbcL* (87.1%) and *matK* (94.3%) among 63 species in 48 genera from China [192]. The high resolution in this study can be attributed to the one or a few species per genus that were sampled. We sampled 21 genera and 44 species in Asteraceae (Figure S5), and find just 48% species resolution with *rbcL* + *matK*. Twelve of these genera include a single species and are distinct at the genus level (and therefore at the species level, as well) whereas the remaining genera include two or more species, most of which are not distinguished by the barcode data. An exception is *Artemisia* L., in which the three sampled species can be identified. This is not surprising as each of these species is part of a different major clade in the genus [193]. Similarly, the two sampled species of *Arnica* L. are distinguished by *matK*; these two species are not closely related [194].

The ability of plastid loci to discriminate species is related to the rate of molecular evolution in a genus or lineage, the length of time that species have been separated, phylogenetic relationships among sampled species and genes, and other evolutionary and biological phenomena such as polyploidy, recent and past hybridization, apomixis, coalescence failure, and introgression (e.g., [34,51]). In general, closely related species are less likely to be distinguished by plastid DNA barcodes compared with those that are more distantly related, as demonstrated in studies that have explicitly considered closely related taxa (e.g., [175,195]). A recent study of the origins and diversification of the global Arctic flora, based on analyses of molecular phylogenetic studies that included Arctic taxa, found that congeneric Arctic species originated mostly independently in unrelated lineages, and that there were few species' radiations in the Arctic [196]. This study considered about 40% of Arctic genera and some 30% of Arctic species. Based on this study, species identification in the Arctic flora and the proportion of species in a genus that can be discriminated should not be greatly affected as the number of species sampled per genus increases, because most species are not expected to be closely related. Contrary to these expectations, however, overall discrimination in the Arctic flora with the core plastid barcodes is low, and, as the number of species sampled per genus increases in the current data set, species resolution tends to decrease (Figure 8). 

### Resolution of Infraspecific Taxa with DNA Barcodes

Few plant barcoding studies have explicitly considered the ability of barcoding markers to discriminate infraspecific taxa (varieties and subspecies), yet accurate identification of infraspecific taxa can be as important as identification to species level in many avenues of research (e.g., conservation biology, rare species assessments, floristic inventories, phylogeography, etc.). The global Arctic flora contains a large number of infraspecific taxa, many of which have been variously recognized at specific or infraspecific ranks [20]. Although sometimes difficult to identify, most are defined by combinations of morphological characteristics, unique distributions and ploidy differences, all reflecting putatively unique evolutionary origins. Some 30 species from 11 families in our study include more than one infraspecific taxon. Of these all but five cannot be distinguished from one another by the core plastid barcodes (Table S1). This is not surprising given that many closely related species cannot be distinguished by the core barcode loci. There is no infraspecific variation between *P. hartzii* subsp. *hartzii* and subsp. *vrangelica*, the latter a viviparous taxon of plants. This trait is variable within Canadian Arctic populations, and subspecies recognition may not be appropriate in the North American Arctic (LJ Gillespie, personal observation). 

In a few species there is infraspecific variation in the barcoding loci that corresponds to infraspecific taxa. *rbcL* distinguishes *Stuckenia filiformis* subsp. *filiformis* from three additional infraspecific taxa: subsp. *alpina* (Blytt) R.R. Haynes, Les & M. Král, subsp. *occidentalis* (J.W. Robbins) R.R. Haynes, Les & M. Král and subsp. *borealis* (Raf.) Tzvelev & Elven (=var. *borealis* (Raf.) H. St. John). *rbcL* and *matK* sequences of *S. filiformis* subsp. *occidentalis* are identical to those of *S. vaginata* and *S. subretusa* (Hagstr.) Holub. Elven et al. [20] suggested that this taxon may be closer to *S. vaginata* and *S. pectinata* (L.) Börner; however, a *matK* sequence for *S. pectinata* is more similar to individuals of *S. filiformis* subspp. *alpina* and *borealis*. *matK* and *rbcL* + *matK* distinguish *Oxytropis borealis* var. *borealis* from *O. borealis* var. *viscida*, and *rbcL* + *matK* distinguish *Eriophorum scheuchzeri* subsp. *scheuchzeri* and *E. scheuchzeri* subsp. *arcticum*. The *S. filiformis* and *O. borealis* species complexes are both taxonomically problematic, and the differences in their barcode data may be a function of taxonomy that does not reflect evolutionary history [20]. 

The general lack of resolution of infraspecific taxa here parallels the findings of a barcoding study of Japanese pteridophytes that included ca. 40 additional infraspecific taxa and demonstrated that discrimination was lower when infraspecific taxa were considered as distinct species than when considered only at the species level [96], indicating that many infraspecific taxa could not be discriminated with the tested markers (the study did not indicate explicitly if any conspecific infraspecific taxa were discriminated). Supplementary markers such as ITS2 may be more useful in discriminating infraspecific taxa in the Arctic flora.

### Infraspecific Genetic Variation

In a few species we detected considerable infraspecific variation that has not been reported previously in the literature. For example, the four sampled individuals of *Lupinus arcticus* S. Watson form two clusters, each with two individuals, based on the *rbcL* (two substitutions) and *matK* (five substitutions) data (Figure S19). We also found a deep genetic divergence in *rbcL* in *Equisetum variegatum* Schleich. ex F. Weber & D. Mohr, in which five substitutions define two clusters of individuals (Figure S17); we were not able to sequence *matK* in this genus. Infraspecific variation has not been reported in any *Equisetum* species previously, as phylogenetic studies have only sampled a single individual per taxon [197–199]. There is no variation in *rbcL* in any of the other *Equisetum* species that we sampled. Four additional *rbcL* sequences of *E. variegatum* have been published, from collections gathered in Churchill (GenBank accession no: JN965527 [109]), southern Ontario (HQ590086 [34]), Alaska (AY226134 [199]), and Japan (AB574691.1 [96]). The first three of these match the more common haplotype found in 14 individuals, and the Japanese sample matches the less common haplotype found in six individuals (data not shown). In both *L. arcticus* and *E. variegatum* there is no obvious geographical pattern to the observed variation, but it may represent phylogeographic variation or ancestral polymorphisms. Broader sampling from throughout the global ranges of these taxa is needed to properly characterize the observed variation from a geographical perspective. The variation in *Equisetum* is unlikely to be related to taxonomic problems with the circumscription of the species, as plants sampled here belong to the taxonomically stable circumboreal *E. variegatum* subsp. *variegatum*. A second subspecies not sampled here or elsewhere, *E. variegatum* subsp. *alaskanum* (A.A. Eaton) Hultén, is restricted to the Pacific coast in Alaska, British Columbia and Washington [200]. 

In a subset of taxa, most with problematic taxonomy, we detected infraspecific variation that may provide insight into the circumscription of taxa. Variation among individuals originally determined as *Chrysosplenium tetrandrum* Th. Fr. prompted us to re-examine the voucher specimens. We found a subset of these to be misidentified specimens of *C. rosendahlii* Packer, a species described from Somerset Island (Nunavut) [119] that was later reduced to synonymy or ignored [23,201], and more recently recognized again as a distinct species [202]. The barcode data distinguish *C. tetrandrum*, *C. rosendahlii*, and *C. wrightii* Franch. & Sav. (Figure S45). Variation in *matK* segregates the 21 sampled individuals of *Stellaria longipes* Goldie—a notoriously difficult polymorphic and polyploid species complex [20,203]—into two clusters (Figure S11). These clusters are distinguished by three characters, and there is further variation within each cluster, including a six base pair insertion shared by five individuals in the seven-individual cluster. We recognized all members of the complex sampled here as *S. longipes*, following recent treatments in North America (e.g., [25,204,205]), whereas other authors have recognized multiple species or infraspecific taxa in the complex [20,203]. Plastid variation apparently has not been studied within and among *S. longipes* and its closest relatives in North America. Additional study is needed to determine the origins of the plastid variation that we observe, and if it relates to the taxonomy of the species. 

One individual of *Astragalus eucosmus* B.L. Rob., collected on Herschel Island, Yukon, has a *matK* haplotype distinct from other sampled individuals of the species, which cluster with another species, *A. richardsonii* E. Sheld. (Figure S19). The unique individual was previously identified as *A. eucosmus* subsp. *sealei* (Lepage) Hultén, a diploid amphi-Beringian taxon that has also been recognized as a distinct species, *A. sealei* Lepage (e.g., [20]). *Astragalus sealei* is included in *A. eucosmus* in other recent treatments (e.g., [25]), which we followed. Plastid variation has been detected previously in the widespread and tetraploid *A. eucosmus* [206], but that study did not sample subsp. *sealei*. Elven et al. [20] suggested that *A. sealei* is morphologically more similar to *A. norvegicus* Grauer, a Eurasian species, than it is to *A. eucosmus*. The variation in *matK* suggests that *A. sealei* and *A. eucosmus* may represent distinct plastid lineages and may support their recognition as distinct species, but broader samplings of both taxa and other putatively allied species, such as *A. richardsonii* and *A. norvegicus*, are needed to establish this. 

We also detected considerable variation in species of *Luzula* (Figure S2). Some individuals of *L. nivalis* (Laest.) Spreng. are identical to individuals of *L. arcuata* subsp. *unalachkensis* (Buchenau) Hultén, while others have a distinct haplotype; individuals of *L. confusa* Lindb. cluster into two distinct groups; and the two sampled individuals of *L. kjellmaniana* Miyabe and Kudo each have unique haplotypes and do not cluster together. All of these species are classified in *Luzula* sect. *Thyrsanochlamydeae* Satake, along with *L. subcongesta* (S. Watson) Jeps., a Californian endemic [126]. The section was found to be non-monophyletic in phylogenetic analyses, and it has been suggested that the section or some of its species may be of hybrid origin [207–210]. These previous studies sampled only a single individual per taxon and did not detect infraspecific variation as we find here. Given the infraspecific variation uncovered in several *Luzula* species, sampling multiple individuals per taxon may be critical for resolving phylogenetic relationships among these and possibly other species in the genus.

### Towards a Comprehensive DNA Barcode Library for Canadian Arctic Vascular Plants

We generated DNA barcode data for at least one of the core plastid loci for 490 vascular plant species, and for both loci for 418 species (88% of the total number of species sampled). These new barcodes represent nearly half of the some 1100 vascular plant species reported from the Canadian Arctic [20]. Many of the unsampled species are 'borderline' Artic taxa that barely extend into the region [20]. Barcode data for some species not sampled here have been generated from Churchill [109]. Our species sampling is comprehensive in the Canadian Arctic Archipelago. The Flora of the Canadian Arctic Archipelago [25] reported 341 species plus eight additional infraspecific taxa for the region. We have produced new barcode data for 316 (93%) of the vascular plant species known from this region (excluding from consideration the two *Papaver* taxa treated in the Flora due to a differing taxonomic treatment used here for the genus). Species from the Canadian Arctic Archipelago for which barcode data were not obtained are listed in Table S4. Continued taxonomic and geographical sampling is needed to complete the barcode reference library for Canadian Arctic vascular plants. Additional sampling is required for species with only one or a few individuals sampled, for species that have not yet been barcoded, and from poorly represented geographical regions, including northern Quebec and northern Labrador.

### Species Resolution—A Closer Look at Barcode Variation Within Arctic Genera in the Context of Systematic Knowledge

Considering the ability of plastid barcodes to discriminate species in light of knowledge of their phylogenetic relationships and evolutionary history provides context helpful for understanding why some species are resolved by barcode loci and others are not. Barcoding studies at lower taxonomic levels (genus or family levels, for example) often consider patterns of variation in the barcode loci in the context of the systematics of their respective groups, whereas most broader barcoding studies have focused primarily on the overall ability of barcode loci to discriminate species in their data sets. The latter is unfortunate, as the large amounts of new data generated in such studies can provide important contributions to systematic knowledge in these groups, as we demonstrate here. Below we discuss a subset of our barcoding results in detail at the level of genus. In doing so, we highlight the ability of the core barcode loci to discriminate closely and distantly related species and place an individual among its closely related species in a species group. We also discuss putative identification problems, newly detected instances of possible hybridization and/or introgression, and the effects of widespread introgression on species identification with DNA barcodes. Given the large number of genera and species in the data set, we focus on a subset of genera in which we sampled few (two to four) species, and on the ten genera in the data set with the greatest number of species sampled (*Puccinellia*, *Festuca*, *Poa*, *Pedicularis*, *Salix, Draba, Saxifraga, Ranunculus, Potentilla*, *Carex*). We also discuss barcode variation in Arctic taxa of the dandelion genus (*Taraxacum* F.H. Wigg.) in light of previous studies that have demonstrated its complex and poorly understood patterns of plastid DNA variation, as well as barcode variation in the polyphyletic genus *Minuartia*.

#### Species Resolution in Genera with Few Species Sampled

The data set includes 53 genera with two to four species sampled for *rbcL* + *matK*. Among these species, discrimination with *rbcL* + *matK* ranges from 100% in 29 genera (Table 1) to 0% in 17 genera (Table 2). In the former group, phylogenetic work indicates that the few sampled species in many of these genera are not particularly closely related (e.g., *Anemone* L. [211], *Arnica* [194], *Artemisia* [193,212], *Campanula* L. [213], *Cardamine* L. [214], *Kobresia* [215], *Pinguicula* L. [216], *Rhododendron* L. [217], *Stellaria* L. [218], *Tofieldia* Huds. [219], *Vaccinium* L. [220]), and it is therefore unsurprising that the congeneric taxa can be distinguished with the core barcode loci. Complementary molecular data for some of the species have been generated in phylogeographic research. For example, the sampled species of *Sagina* L. (*S. nivalis* (Lindblom) Fr., *S. caespitosa* (J. Vahl) Lange) and *Vaccinium* L. (*V. uliginosum* L., *V. vitis-idaea* L.), all distinguished by *rbcL* and *matK*, have been shown to be similarly distinguished by variation in plastid intergenic spacers [221,222]. 

There are 21 genera with two or more species sampled in which there is no species resolution with *rbcL* + *matK*: 17 have two to four species, two have five species, one has 16 species, and one has 22 species (Table 2). Most of the indistinguishable species are closely related, and in several instances they are taxonomically problematic. There is either no variation in the plastid barcode loci among these species, or the variation in the core barcode loci is not consistent with species boundaries. Among these are several Asteraceae genera (Figure S5): *Petasites*, a genus with three northern taxa recognized here as two species, which have been variously treated as species or infraspecific taxa [129,130,223]; *Tephroseris* (Rchb.) Rchb., whose North American species are poorly defined and closely related [224,225]; and *Antennaria* Gaertn., whose taxonomy is complicated by polyploidy and apomixis [20,226]. There are several Poaceae genera with indistinguishable species (Figure S37): *Agrostis* L., a genus of morphologically similar species whose taxonomy is complicated by polyploidy and hybridization (e.g., [227]); *Calamagrostis* Adans., a large genus of polyploid taxa among which relationships are not clear [20,189]; *Deschampsia* P. Beauv., a genus that includes several closely related northern taxa that are morphologically and molecularly similar, and which may be best recognized as a single species [228]; *Elymus* L., a genus including several morphologically and molecularly similar northern taxa [229,230]; and *Phippsia* (Trin.) R.Br., a genus of two species with complicated taxonomic histories [231–233]. Other genera with indistinguishable species include *Betula* L. (Figure S6), a taxonomically difficult woody genus [20] in which the lack of discrimination among species here is consistent with other barcoding studies of the genus [34,234]; *Papaver* (Figure S33), a difficult genus in the Arctic with taxonomy complicated by hybridization and polyploidy [20,127]; *Hippuris* L. (Figure S35), an aquatic genus in which the three sampled taxa have been variously recognized as species or infraspecific taxa [20]; *Stuckenia* Börner (Figure S40), an aquatic genus with taxonomic problems among the northern species sampled [20,235]; and *Cerastium* L. (Figure S11), which includes several taxonomically difficult northern species with low levels of genetic variation [218,236,237]. 

Some of the congeneric species not distinguished by the barcode data are not closely related. For example, the polymorphic and widely distributed species *Suaeda maritima* (L.) Dumort. and *S. calceoliformis* (Hook.) Moq. [238] are each part of distinct, well-supported clades in a phylogenetic study based on ITS and two plastid intergenic spacer regions [239], whereas in our analyses these taxa have identical *rbcL* and *matK* sequences (Figure S3). In *Primula*, the three sampled taxa (*P. borealis* Duby, *P. stricta* Hornem., *P. egaliksensis* Wormsk.) are part of different well-supported major lineages in a phylogeny based on rapidly evolving plastid intergenic spacers [240], but the barcode data here fail to discriminate them, as haplotypes are shared among species (Figure S41). The inability of the core barcode loci to distinguish these *Suaeda* and *Primula* taxa may reflect identification problems, unresolved taxonomic problems with these species, or simply low variation in *matK* and *rbcL* compared with the more rapidly evolving plastid regions.

#### Puccinellia

*Puccinellia* is a taxonomically difficult grass genus with about 120 species, of which 12 occur in the Canadian Arctic [134,136]. We sampled all of these plus *P. distans* (Jacq.) Parl. from the adjacent boreal region, the amphi-Pacific/Beringian *P. alaskana* Scribn. & Merr., and *P. hauptiana* Scribn. & Merr. from Russia—a total of 15 species. Just four of 13 species with data for *rbcL* + *matK* are discriminated with the combined loci (Figure 7). *Puccinellia* species group into three main clusters, two of which are well-defined by their relatively long branches in the neighbour joining trees (Figure S48). One well-defined cluster includes *P. pumila* (Vasey) Hitchc., *P. nuttalliana* (Schult.) Hitchc., *P. vaginata* (Lange) Fernald & Weath., *P. distans*, and *P. nutkaensis* (J. Presl) Fernald & Weath. The second well-defined cluster includes *P. tenella* subsp. *langeana* (Berlin) Tzvelev, *P. vahliana* (Liebm.) Scribn. & Merr., *P. phryganodes* (Trin.) Scribn. & Merr., and *P. alaskana*. The remaining taxa plus one individual of *P. pumila* are intermixed; this group includes *P. angustata* (R. Br.) E.L. Rand & Redfield, *P. arctica* (Hook.) Fernald & Weath., *P. bruggemannii* T.J. Sørensen, *P. andersonii* Swallen, and *P. banksiensis* Consaul. The latter two groupings are similar in composition to major clades identified in recent systematic work on Arctic *Puccinellia* [137–140]. 

Only one of the recent studies [140] included *P. nuttalliana*, and none included *P. vaginata* or *P. nutkaensis*. Based on the plastid data here these taxa are closely related to each other and to most sampled individuals of *P. pumila*. The single individual of *P. nuttalliana* sampled by Consaul et al. [140] for nuclear ribosomal and plastid DNA was closely related to *P. arctica* and *P. angustata*, whereas our plastid data place the latter taxa and *P. nuttalliana* in different clusters. Individuals of *P. pumila* sampled previously for nuclear ribosomal and plastid DNA were part of a clade with *P. arctica*, *P. banksiensis*, *P. andersonii*, *P. angustata*, and *P. bruggemannii* [139,140]. By contrast, just one of the several individuals of *P. pumila* sampled here for *rbcL* + *matK* shows these affinities, with the remainder clustering with *P. nuttalliana*, *P. vaginata*, *P. distans*, and *P. nutkaensis*. *Puccinellia pumila* was considered to be part of the *P. nuttalliana* complex (also including *P. nutkaensis*) by Davis [241], a grouping supported by the barcode data for most individuals of the taxon. Given the variation in the species, the taxonomy and evolutionary history of *P. pumila* warrants further study. This is a good example of how barcode data can provide new insights into the taxonomy of under-studied or poorly known species. 

The barcode data can readily place an unknown *Puccinellia* individual among a major group of species, which is helpful for narrowing the possibilities for an identification, but within each group multiple haplotypes are shared among most species. This may reflect the fact that many Arctic *Puccinellia* species are polyploids (8/13 species here, excluding *P. distans*, which is diploid and polyploid), a subset of which are known to have evolved from Arctic diploids and likely share plastid haplotypes with them [138,139]. In the first group, only *P. nutkaensis* (two individuals) and *P. distans* (one individual) have unique haplotypes based on the current sampling (Figure S48). In the second group, *matK* does not distinguish any taxa as multiple haplotypes [242] are shared among species within each of the two subclusters, and *rbcL* distinguishes just two taxa, *P. phryganodes* and *P. tenella* subsp. *langeana* (Figure S48). 

The supplementary plastid regions *psbA–trnH* (Table S3, Figure S49) and *psbK–psbI* (Table S3, Figure S51) did not resolve any of the ten *Puccinellia* taxa sampled for these loci. The *atpF–atpH* intergenic spacer was more variable and distinguished four of nine *Puccinellia* species (Table S3, Figure S50), including three species that neither *matK* nor *rbcL* discriminates (*P. alaskana*, *P. vahliana*, *P. arctica*). *Puccinellia tenella* subsp. *langeana*, distinguished by *rbcL*, and *P. nutkaensis* and *P. distans*, distinguished by *matK*, were not sampled for *atpF–atpH*. They should be examined for this locus, as it may be the best plastid locus for resolution of Arctic *Puccinellia* species. 

##### Festuca

We sampled all ten species of *Festuca* that occur in the Canadian Arctic [243]. Of these, only three-F*. rubra* L. s.l., *F. baffinensis* Polunin, and *F. altaica* Trin.—can be discriminated with the combined barcode loci (Figures 7, S52). *Festuca rubra* and *F. altaica* are not closely related to each other or to the remaining taxa [244], of which all but *F. prolifera* (Piper) Fernald are part of a broader group of arctic-alpine and non-Arctic taxa referred to as the *Festuca ovina* L. complex (sheep fescue complex) [245]. Members of this complex sampled here include *F. baffinensis*, *F. brachyphylla*, *F. brevissima* Jurtzev, *F. edlundiae* S.G. Aiken, L.L. Consaul & Lefk., *F. hyperborea* Holmen ex Fred., *F. lenensis* Drobow, and *F. viviparoidea* Krajina ex Pavlick subsp. *viviparoidea*, the latter a pseudoviviparous taxon. 

*Festuca baffinensis*, *F. brachyphylla*, *F. hyperborea*, and *F. edlundiae* have been called the *F. brachyphylla* complex [122]. *Festuca baffinensis* is distinguished from the remainder of the complex by the barcode loci (Figure S52), a finding consistent with other morphological and molecular research [246,247]. Barcode variation among the remaining taxa is represented by a few haplotypes shared among species (Figure S52). Hybridization and/or introgression among the morphologically similar and closely related *F. brachyphylla*, *F. edlundiae*, and *F. hyperborea* [122,247] has been documented in Svalbard [246,247], and hybridization and introgression is also likely among these species in the North American Arctic, which may be the cause of their shared plastid barcode haplotypes. *Festuca brevissima*, a Beringian taxon that is distinct from other taxa in the North American *F. ovina* complex based on isozyme data [242], is not distinguished by the barcode data. Although two individuals of *F. brevissima* have a unique *matK* haplotype, *matK* in a third individual is identical to other members of the complex. 

The plastid barcode data provide new insights into the putative origins of two viviparous Arctic *Festuca* taxa. *Festuca viviparoidea* may be of hybrid origin, possibly with *F. brachyphylla* and *F. baffinensis* as parental taxa [243,248]. If this is correct, we can infer that *F. baffinensis* is not the maternal parent, given that its plastid barcode haplotypes differ from those of other members of the *F. brachyphylla* complex, whose *rbcL* and *matK* sequences are identical to those of *F. viviparoidea*. An individual collected on the Belcher Islands and determined as *F. prolifera*—another vegetatively proliferating taxon—also clusters with the *F. brachyphylla* complex (Figure S52), a placement inconsistent with its putative evolutionary relationships. *Festuca prolifera* is considered part of the *F. rubra* complex based on morphological characteristics, and is readily distinguished from pseudoviviparous members of the *F. ovina* complex (*F. viviparoidea*, *F. frederikseniae* E.B. Alexeev) by its loosely cespitose habit and spreading rhizomes (vs. a densely cespitose habit), and straight leaves (vs. conspicuously curled leaves) [249]. Darbyshire and Pavlick [243] noted that viviparous plants from Greenland named *F. villosa-vivipara* (Rosenv.) E.B. Alexeev are similar to *F. prolifera*, and may be hybrids of *F. rubra* and *F. frederikseniae*, a pseudoviviparous taxon that occurs in Greenland, southern Quebec, and Newfoundland and Labrador. The plant from the Belcher Islands could be such a hybrid, or a hybrid between *F. rubra* and another member of the *F. ovina* complex; if so, its placement among members of the *F. brachyphylla* complex would not be unreasonable if the maternal parent were a member of the complex. Additional plastid and nuclear data from multiple individuals of *F. prolifera*, *F. frederikseniae*, *F. villosa-vivipara, F. rubra* and their putative hybrids are needed to determine their evolutionary relationships, as in recent research that has characterized relationships between *F. ovina* s.s. and the pseudoviviparous species *F. vivipara* (L.) Sm. in the United Kingdom [250].

#### Poa

Eleven species of *Poa* are known from the Canadian Arctic, including several boreal species that extend into the Low Arctic (*P. leptocoma* Trin., *P. palustris* L., *P. paucispicula* Scribn. & Merr., *P. pseudoabbreviata* Roshev.) [131,251]. We sampled all of these except *P. leptocoma*. We also sampled *P. stenantha* Trin., distributed in western North America. Five of the 11 *Poa* species sampled (*P. abbreviata* R.Br., *P. alpina* L., *P. ammophila* A.E. Porsild, *P. pseudoabbreviata* Roshev., *P. paucispicula* Scribn. & Merr.; Figure 7) can be distinguished with *rbcL + matK* (Figure S53). The closely related species *P. abbreviata* and *P. pseudoabbreviata* (*Poa* sect. *Abbreviatae* Nannf. ex Tzvelev [251]) have unique *matK* barcodes (Figure S53). *Poa alpina* is the only species of *Poa* sect. *Alpinae* (Hegetschw. ex Nyman) Stapf that occurs in Canada [251], and its barcodes distinguish it from all other *Poa* taxa sampled. The many sampled individuals of *P. arctica* R.Br. (including three subspecies) and *P. pratensis* L. (including three subspecies) are indistinguishable as they share several haplotypes (Figure S53). These species are closely related, morphologically similar, and are known to hybridize [187,251,252].

The supplementary plastid barcode regions do not distinguish any *Poa* species not distinguished by *rbcL* and/or *matK*. The *psbA–trnH* data distinguish *P. abbreviata* and *P. alpina* (Figure S49), the *psbK–psbI* data distinguish only *P. alpina* (Figure S51), and *atpF–atpH* data distinguish *P. alpina* and *P. ammophila* (Figure S50, Table S3).

Chloroplast capture is a well-known phenomenon in plants, and in such cases plastid barcode data alone will be problematic for discriminating species [34,39,42,51,253]. Previous work, based on restriction site and DNA sequence data, has documented two distinct chloroplast haplotypes in *P. hartzii* Gand. (*Poa* sect. *Secundae* V.L. Marsh ex Soreng): one unique to the species and one identical or similar to the dominant haplotype of the widespread and morphologically distinctive species *P. glauca* Vahl (*Poa* sect. *Stenopoa* Dumort.) [131,132,187]. The *P. glauca* haplotype in *P. hartzii* may have resulted from chloroplast capture of *P. glauca* plastid DNA via inter-sectional hybridization and introgression in the High Arctic [131]. We sampled individuals of *P. hartzii* known to contain either of the two haplotypes, in addition to samples of unknown haplotype, to demonstrate how such introgression is problematic for species identification via DNA barcoding.

The pattern of introgression is not evident in the *rbcL* tree, as *rbcL* sequences are identical in *P. abbreviata*, *P. glauca*, *P. hartzii*, *P. stenantha* Trin. and *P. pseudoabbreviata* (Figures S37, S53). In the *matK* tree, a subset of the sampled individuals of *P. hartzii* (and an identical individual determined as *P. stenantha*) is distinct compared with *P. ammophila* (also classified in *Poa* sect. *Secundae*) and all other taxa, whereas another subset of *P. hartzii* individuals is indistinguishable from individuals of *P. glauca* (Figures S37, S53). These latter individuals are easily identified as *P. hartzii* based on morphological characteristics, but they have plastid DNA introgressed from *P. glauca*. Given the presence of *P. glauca* plastid DNA in *P. hartzii*, these species cannot consistently be distinguished with plastid barcodes. We are able to interpret this introgressant pattern of plastid variation in light of *a priori* knowledge of chloroplast capture in *P. hartzii*, but in general when constructing barcode reference databases, instances of putative chloroplast capture are not usually known in advance or readily identifiable. When putative introgression is detected, factors such as sample misidentification, contamination, and incomplete lineage sorting also need to be considered to explain a putative incongruence between morphology and plastid lineage.

Our survey of plastid variation in *P. hartzii* also expands the distribution of the introgressed form of *P. hartzii* into the western Arctic. Gillespie et al. [132] found the introgressant *P. glauca* haplotype only in High Arctic populations of *P. hartzii* on Ellesmere Island and Axel Heiberg Island; none of the three populations sampled from Cambridge Bay, Victoria Island, had the *P. glauca* haplotype. We sampled three recent collections of *P. hartzii* from western Victoria Island (Ulukhaktok and Minto Inlet), and one of these (*Gillespie et al. 10078*) had the introgressed *P. glauca* haplotype. As barcode data accumulate and the number of individuals sampled per species increases, knowledge of the distribution of taxa with introgressed plastid DNA, as well as many new instances of putative introgression and chloroplast capture are likely to be documented, increasing our understanding of the prevalence of this phenomenon in the Arctic and elsewhere.

#### Pedicularis

*Pedicularis* is a globally distributed genus of hemiparasitic plants with more than 500 species [254]. We sampled the ten *Pedicularis* species that occur in the Canadian Arctic [20], and not more than 40% of these are discriminated by *rbcL* + *matK* (Figures 7, S54). A barcoding study of 88 species of *Pedicularis* from China, where the genus is most diverse, found *rbcL* + *matK* to distinguish 54–70% of species—depending on the criteria used for species discrimination—and suggested rbcL + ITS as the best two-locus barcode for the genus [62]. Most North American and Arctic species have not yet been included in phylogenetic studies of *Pedicularis* [254] and their relationships are unclear, but the barcode data suggest Arctic species are likely represented by several major lineages. There are deep genetic divergences among *P. capitata* Adams; *P. flammea* L.; *P. verticillata* L.; a *P. albolabiata* (Hultén) Kozhevn. and *P. arctoeuropaea* (Hultén) Molau & d.f. Murray cluster; a *P. lapponica* L. and *P. labradorica* Houtt. cluster; and a *P. hirsuta* L., *P. langsdorffii* Fisch. ex Steven, and *P. lanata* Willd. ex Cham. & Schltdl. cluster (Figure S54). 

These divergent species and clusters are readily distinguished with the barcode data, but not all species are distinguishable within the clusters. *Pedicularis albolabiata* and *P. arctoeuropaea*, morphologically similar and closely related taxa recognized only recently at the species level [128], are not distinguished with the barcode data. In the *P. lanata*–*P. hirsuta*–*P. langsdorffii* cluster, the multiple individuals of each species also form clusters, consistent with their taxonomy, with the exception of one individual determined as *P. langsdorffii* that groups with *P. hirsuta*, and two specimens (*Gillespie et al. 6105* from Axel Heiberg Island, and *Gillespie et al. 5218* from Ellesmere Island) that are intermediate in morphology between *P. hirsuta* and *P. langsdorffii* with *matK* sequences identical to those of *P. hirsuta* (Figure S54). Porsild [22] also noted plants with morphologies intermediate between *P. hirsuta* and *P. langsdorffii* from northwest Greenland, and Ellesmere and Baffin Islands. These plants do not have a scientific name, and may represent hybrids (*P. langsdorffii* × *P. hirsuta*). If so, *P. hirsuta* may be the maternal parent, given its shared *matK* haplotype with the intermediate individuals. The individual determined as *P. langsdorffii* may also be a hybrid (or a misidentification), given its placement in the *P. hirsuta* cluster, or we may have detected another instance of introgression. Based on the current data there is no way to unambiguously distinguish *P. hirsuta* from putative hybrids for which it may be the maternal parental—a good example of how hybridization may negatively affect the success of barcoding with plastid markers. *Pedicularis labradorica* and *P. lapponica* are distinct from the rest of the genus. For the most part, individuals of these two species form sister clusters, but one individual of *P. lapponica* (*Consaul et al. 3692* from the Belcher Islands, Nunavut) is identical in *matK* to *P. labradorica*. The specimen is correctly identified, thus this placement may reflect hybridization or introgression—which apparently have not been noted for these species—or contamination. 

#### Salix

There are some 27 *Salix* species in the Canadian Arctic [255]. We sampled 20 of these plus one putative hybrid (*S. arctica* Pall. × *S. polaris* Wahlenb.) and three non-Arctic taxa (*S. silicifolia* Raup, *S.* stolonifera Coville, *S. pseudomyrsinites* Andersson). Discrimination with the core barcode loci fails remarkably in *Salix*, as no species can be discriminated (Figure 7). Although the Arctic species are morphologically diverse—ranging from dwarf, prostrate shrubs (e.g., *S. herbacea* L.) to several-meter-high tree-like bushes (e.g., *S. alaxensis* (Andersson) Coville)—variation in the barcode regions among the sampled species is very low (Figure S55). All but two individuals have identical *rbcL* barcodes. In *matK* there is slightly higher variation, but no *matK* haplotypes are unique to any species. These results are consistent with *rbcL* and *matK* data generated for willow species from Churchill [109] and Finland [234], which were similarly found to be indistinguishable. Kuzmina et al. [109] also found that multiple ITS2 haplotypes did not correspond to willow species boundaries. Low plastid variation among Arctic *Salix* species is also consistent with phylogenetic work in the genus, in which multiple species have been found to share identical plastid haplotypes [256–258]. 

#### Draba

*Draba* is a large and taxonomically complicated genus with extensive morphological variation, frequent hybridization, and polyploidy, and it is one of the largest genera in the Canadian Arctic [20,259]. Seventeen species are reported from the Canadian Arctic Archipelago [25], with additional species on the Arctic mainland [20,23]. Arctic *Draba* species are difficult to identify, in part because there are no up-to-date taxonomic keys for the genus in the Arctic or northern North America. Thus, some of our material may be misidentified. Of the 16 to 19 species we sampled, just one is discriminated by *rbcL*, and none are discriminated by *matK* and combined *rbcL* + *matK* data (Figures 7, S56). Identification of *Draba* species with DNA barcodes has also been problematic in other studies. For example, Yao et al. [56] examined 199 species of *Draba* in their study of ITS2 as a plant barcode, and found that just 27% of species could be discriminated—the second lowest level of success in their large study.

We find that most plastid haplotypes are distributed among two or more *Draba* species. In the *rbcL* tree, some individuals of *D. juvenilis* and *D. glabella* cluster together, while other individuals of these taxa share an identical haplotype with all other sampled taxa, except single individuals of *D. crassifolia* Graham and *D. arctica*, which each have unique haplotypes (Figure S8). We consider *D. crassifolia* to be discriminated by the *rbcL* data, but further sampling and *matK* data, which we were not able to obtain for this sample, are needed to confirm its putative distinctiveness. *matK* is more variable than *rbcL* in *Draba*, but most *matK* haplotypes are distributed among multiple species. No species can be distinguished with the *matK* data (Figure S8). In the *rbcL* + *matK* neighbour joining tree (Figures S8, S56) several clusters include individuals of one species (e.g., *D. nivalis* Lilj.) or a few species (e.g., individuals of *D. corymbosa* R. Br. ex DC. and *D. lactea* Adams; individuals of *D. subcapitata* Simmons, *D. simmonsii* Elven & Al-Shehbaz, *D. pilosa* Adams ex DC., and *D. micropetala* Hook.; individuals of *D. glabella* Pursh, *D. juvenilis* Kom., and *D. arctica* J. Vahl), but other individuals of most of these species share a haplotype with other species. The exceptions may be due to misidentification and/or hybridization, and further investigation combined with a better understanding of the taxonomy is necessary to determine if species and species groups can be resolved. 

#### Saxifraga

We sampled all of the 14 species of *Saxifraga* that occur in the Canadian Arctic [23,25], and 69% of these are distinguished with *rbcL* + *matK*—the second highest species resolution among the ten largest genera sampled (Figure 7). The clustering pattern of *Saxifraga* species, based on the barcode data, is similar to evolutionary tree topology in a phylogenetic study based on *matK* and ITS data that included nine of the species sampled here [260]. In the current sampling only the species pairs *S. cernua* L. and *S. radiata* Small, and *S. bronchialis* L. and *S. tricuspidata* Rottb., are indistinguishable with the combined data (Figure S57). *Saxifraga cernua*, a heterogeneous high polyploid that likely is a complex hybrid species, and *S. radiata*, which includes diploid and low polyploid individuals, are part of the "*Saxifraga sibirica* L. aggregate", and transitional forms and/or hybrids between the species have been documented in various parts of their ranges [20]. This may be the reason for a shared haplotype among the two sampled individuals of *S. radiata* and one individual of *S. cernua*. *Saxifraga bronchialis* (treated as *S. funstonii* (Small) Fedde in Elven et al. [20] and *S. bronchialis* subsp. *funstonii* (Small) Hultén in Brouillet and Elvander [261]) and *S. tricuspidata* are closely related and part of the *S. bronchialis* complex, a group that requires additional taxonomic research [261]. *matK* failed to amplify for *S. rivularis*, a tetraploid species that is distinct from the closely related diploid *S. hyperborea* [262]. *Saxifraga hyperborea* is thought to be the maternal parent of *S. rivularis*, and *matK* sequences generated for these taxa from Svalbard differed by a single substitution [263]; they cannot be discriminated with the *rbcL* data presented here.

#### Ranunculus

*Ranunculus* is represented in the Canadian Arctic by some 13 species, the exact number depending on how some taxa are circumscribed [20,264]. We sampled 12 species for *rbcL* and 11 for *matK* and *rbcL* + *matK*. Seven of 11 species (63%) are resolved with the combined barcode data (Figure 7). In the neighbour joining trees, *Ranunculus* species cluster into two main groups (Figure S58) that generally correspond to two clades identified in several phylogenetic studies [265–269]. One group comprises the water buttercups and includes two subclusters: one of *R. confervoides* (Fr.) Fr., *R. subrigidus* W.B. Drew, and *R. aquatilis* var. *diffusa* With. (*Ranunculus* sect. *Batrachium* DC.); the other of *R. hyperboreus* and *R. gmelinii* DC. (*Ranunculus* sect. *Hecatonia* (Lour.) DC.). The second group includes *R. sulphureus* Sol., *R. allenii* B.L. Rob., *R. sabinei* R.Br., *R. pygmaeus* Wahlenb., *R. nivalis* L., and *R. arcticus* Richardson. This group is classified as *Ranunculus* sect. *Auricomus* Tamura, distributed in Arctic-circumpolar to temperate mountain regions in the northern hemisphere [269]. All of the sampled taxa in this cluster are distinguished by the barcode data. *Ranunculus allenii*, a northeastern North American endemic [20], has not previously been included in phylogenetic analyses. This species clusters near, but is distinct from, *R. pygmaeus*, supporting its inclusion in *Ranunculus* sect. *Auricomus*.

Our data support a close relationship between *R. gmelinii* and *R. hyperboreus*, as the sampled individuals of these species are intermixed and indistinguishable from each other in a cluster that is part of a larger cluster of aquatic taxa (Figure S58). However, the affinities of *R. gmelinii* are contradictory in other studies. *Ranunculus gmelinii* subsp. *gmelinii* is the sister group of the terrestrial clade based on combined *matK*/*trnK* and ITS data in Paun et al. [265] and Hoffmann et al. [266] (the same sequences were used in both analyses). Hoffman et al. [266] also included *matK* and ITS data for a different infraspecific taxon of the species, *R. gmelinii* subsp. *purshii* (Richardson) Hultén, which they found to be closely related to *R. hyperboreus* Rottb. Other studies used these same *R. gmelinii* subsp. *gmelinii* sequences plus data for an additional plastid region (*psbJ*–*petA*), and these analyses placed the taxon firmly among other aquatic taxa—as do our data—either as the sister group of *R. hyperboreus* (parsimony analyses [268]) or as part of a polytomy with other aquatic species [267]. The reason for the discordant placement of *R. gmelinii* subsp. *gmelinii* among these studies is unclear, since all used the same *matK*/*trnK* and ITS data for the taxon; an additional plastid region is unlikely to affect its position in the trees. Curiously, there are no comments on these differences in the more recent studies [267,268]. As in Scott [270] and Whittemore [264] we do not recognize infraspecific taxa in *R. gmelinii*, whereas Elven et al. [20] recognize subspecies *gmelinii* and *purshii*, as in the phylogenetic studies. Among the other sampled taxa in the water buttercup cluster, only *R. confervoides* is distinguished. *Ranunculus subrigidus* and *R. aquatilis* var. *diffusa* (=*R. trichophyllus* Chaix in Elven et al. [20]), which are sometimes considered to be conspecific (e.g., [264]), have identical barcodes.

#### Potentilla

The taxonomy of *Potentilla* in the Arctic is notoriously difficult, complicated by extensive hybridization, polyploidy, agamospermy, phenotypic plasticity, and varying taxonomic viewpoints [20]. Aiken et al. [25] reported 11 species from the Canadian Arctic Archipelago, and some 33 species have been reported for the North American Arctic (excluding western Alaska and western Greenland) [20]. We sampled 13 species for *rbcL* and 12 species for *rbcL* + *matK*; our sampling focused primarily on species from the Canadian Arctic Archipelago. In the barcode data there is a deep divergence between *P. anserina* (Wormsk.) Rydb. and the remainder of the *Potentilla* taxa sampled (Figure S59). This is consistent with the phylogenetic position of *P. anserina* in a clade that is the sister group of a clade comprising most species of *Potentilla* (i.e., *Potentilla* s.s.) and several (non-Arctic) segregate genera in plastid analyses [271,272]. *Potentilla anserina* has been treated in a separate genus, *Argentina* Hill [25,273], a circumscription that is consistent with the phylogenetic evidence, although recently some authors kept the species in *Potentilla* (e.g., [20,272]). If recognized, *Argentina* can be discriminated by the core barcode loci, as is the case for most genera here, including all Rosaceae genera (Figure S43). 

Among *Potentilla* s.s. species, *P. biflora* Willd. ex Schltdl. is distinguished from the rest of the sampled taxa (Figure S59), consistent with its known distant relationship with a large *Potentilla* clade—the "*Potentilla* core group" of Dobeš and Paule [271] and the "*Argentea* clade" of Topel et al. [272]—that includes many taxonomic series/sections, including those in which all other sampled Arctic taxa are classified. Among these taxa there are five *rbcL* + *matK* haplotypes: one unique to *P. bimundorum* Soják and four distributed among the remaining taxa that are present in 40, 18, 10, and one individual, respectively (Figure S59). Most of the taxa sampled are part of *Potentilla* sect. *Niveae* (Rydb.) A. Nelson, a taxonomically challenging group in which species delimitation is problematic [274–276]: *P. subvahliana* Jurtzev, *P. uniflora* Ledeb., and *P. villosa* Pall. ex Pursh (three of five species that are part of the *P. uniflora* aggregate); *P. nivea* (one of two species comprising the *P. nivea* aggregate); *P. arenosa* (including *P. chamissonis* Hultén, a synonym of *P. arenosa* subsp. *chamissonis* (Hultén) Elven & d.f. Murray); and *P. vahliana* and *P. subgorodkovii* Jurtzev (species of putative hybrid origin from crosses between the *P. uniflora* aggregate and the *P. nivea* aggregate [20]). A few taxa are part of other sections: *P. hyparctica* Malte is included in *Potentilla* sect. *Aurea* (Rydb.) Juz; *P. bimundorum* and *P. pulchella* R.Br. are included in *Potentilla* sect. *Pensylvanicae* Poeverl; and *P. rubricaulis* is included in *Potentilla* sect. *Rubricaules* (Rydb.) A. Nelson. Our sampling also includes several presumed intersectional hybrids (*Potentilla* sect. *Nivea* × *Potentilla* sect. *Pensylvanicae*) that are taxonomically problematic [20,30]. 

None of these sections has been recovered as monophyletic in phylogenetic analyses [271,272], and within *Potentilla* sect. *Niveae* only the *P. uniflora* aggregate has been recovered as a monophyletic group, based on *trnS/G* and *trnL*–*F* plastid data [272]. Dobeš and Paule [271] suggested that the inability to recover any of the sections as clades may be due to incomplete lineage sorting, hybridization among sections and series, low molecular variation, or problems with the classification. Hybridization among members of *Potentilla* sect. *Nivea* in northern North America has been documented with molecular data [276]. All of these factors may also contribute to the shared *rbcL* and *matK* barcodes found in these taxa. 

Although most aspects of species relationships in *Potentilla* are unresolved in phylogenetic studies, there is branch length variation in the phylogenetic trees generated from plastid intergenic spacers and introns [271,272], suggesting that rapidly evolving plastid regions may be more informative than the *rbcL* and *matK* DNA barcodes for species identification in the genus. However, haplotypes in other plastid regions are also likely to be shared among species, as in the core barcode regions. Microsatellites have recently been developed for species in the *Potentilla* core group with high cross-transferability across the clade [277]. These may be useful for characterizing hybridization, species limits and genetic diversity in Arctic species of *Potentilla*, which in turn may provide insights into the distribution of plastid lineages among the Arctic taxa.

#### Carex

*Carex* is the most species-rich genus in the Canadian Arctic, and the genus for which we sampled the greatest number of species. *Carex* also has the highest species resolution among genera with multiple species (i.e., >10) sampled (Figure 7), counter to the general trend observed of decreasing resolution with increased species sampling. *matK* alone distinguishes 82% of sampled species (Figure S60), and if the three sampled species of the genus *Kobresia*—which is phylogenetically nested within *Carex* (e.g., [215,278,279])—are considered with *Carex*, *matK* resolves 84% of species (Figure S13). In *Carex* (and more broadly in Cyperaceae), *matK* is much more variable than *rbcL*, which alone resolved only some 18% of *Carex* species. Not unexpectedly, these results are very similar to the findings of Le Clerc-Blain et al. [108] who characterized the success of multiple plastid markers for barcoding sedges (*Carex* and *Kobresia*) of the Canadian Arctic Archipelago, as there is major overlap in the species they studied and those sampled here. They found that *matK* alone could distinguish some 85% of 23 species, and *rbcL* resolved 67% of 12 species (the *rbcL* data set was incomplete). The sampling in the current study includes new barcode data for all of the *Carex* and *Kobresia* species studied by Le Clerc-Blain et al. [108] as well as new data for 11 species previously unsampled for *matK* and 23 *Carex* and two *Kobresia* species previously unsampled for *rbcL*. Despite a 47% increase in the number of species (from 23 to 34) in the *matK* data set, the percent species resolution is similar to the earlier analysis with just 23 species. 

The high species resolution in *Carex* in the Canadian Arctic is partly a function of the numerous distant lineages represented in the Arctic flora. One major cluster in the neighbour joining tree corresponds to *Carex* subg. *Vignea* [278,279] (Figure S61). Phylogenetic studies of *Carex* subg. *Vignea* have variously included a few of the Arctic species sampled here, which are classified in diverse sections, including *C. gynocrates* Wormsk. ex Drejer (*Carex* sect. *Physoglochin* Dumortier [280]), *C. chordorrhiza* Ehrh. ex L. f. (*rbcL* only, Figure S13; *Carex* sect. *Chordorrhizae* (Heuffel) Meinshausen [281]), *C. maritima* Gunnerus (*Carex* sect. *Foetidae* (Tuckerman ex L. H. Bailey) Kükenthal [282]), *C. diandra* Schrank (*Carex* sect. *Heleoglochin* Dumortier [283]), and *C. canescens* L. and *C. lachenalii* Schkuhr (*Carex* sect. *Glareosae* G.Don [284]). These sectional taxa comprise different lineages [285–288], and each of these species is distinguished by the barcode data. *Carex marina* Dewey and *C. ursina* Dewey, also classified in *Carex* sect. *Glareoseae*, are also distinguished. They have not been included in phylogenetic studies, thus their relationships with each other and other taxa in the section are not known. There is particularly deep divergence in the barcode data between *C. diandra* and the other sampled subg. *Vignea* species, consistent with their distant relationships [285]. 

Another major cluster in the neighbour joining tree corresponds to a clade of unispicate taxa (the Core Unispicate Clade [278,279]) found in phylogenetic studies (Figure S61). This clade includes species of *Carex* and *Kobresia* (e.g., [215,289]), and the five species of the clade sampled here are all distinguished by the barcode data (*C. rupestris* All., *C. nardina* Fr., *K. simpliciuscula* (Wahlenb.) Mack., *K. myosuroides* (Vill.) Fiori, *K. sibirica* (Turcz. ex Ledeb.) Boeck.). 

The majority of the *Carex* species sampled are part of the Core *Carex* Clade [278,279]. *Carex membranacea* Hook., *C. saxatilis* L., and *C. rotundata* Wahlenb. are part of *Carex* sect. *Vesicariae* (Heuffel) J. Carey [290]. Le-Clerc Blain et al. [108] found that *C. membranacea* and *C. saxatilis* were distinguished by a single nucleotide polymorphism, consistent with the current data. They did not sample *C. rotundata*, which we find to be distinguished from *C. membranacea* by one *matK* substitution and from *C. saxatilis* by two *matK* substitutions (Figures S60, S61). A recent phylogenetic study of *Carex* sect. *Vesicariae* in Siberia based on *matK*, *atpF–atpH*, ITS2 and the nuclear *hsp90* gene, found that *C. saxatilis* and *C. rotundata* fall in different major clades [291]. *Carex membranacea* was not included in this study. Our *matK* sequences and those generated by Le Clerc-Blain et al. [108] for *C. membranacea* are identical to those of *C. rostrata* Stokes in the study of Siberian plants (data not shown), and *atpF–atpH* sequences [108] are identical to those of several species in the clade that includes *C. rostrata* and *C. rotundata* (data not shown).

*Carex aquatilis* Wahlenb. and *C. subspathacea* Wormsk., both classified in *Carex* sect. *Phacocystis*, share two *matK* haplotypes. These species are closely related, as evidenced by a phylogenetic analysis that found a clade comprising a paraphyletic *C. aquatilis* and a sublineage including the maritime species *C. subspathacea*, *C. paleacea* Schreb. ex Wahlenb. and *C. ramenskii* Kom. (the latter two species are also found in the Canadian Arctic but not sampled here). *Carex subspathacea* hybridizes with *C. aquatilis* and other species [292], and this may be reflected in their shared barcodes. *Carex bigelowii* Torr. ex Schwein., a taxonomically problematic species (e.g., [293]) that is also part of *Carex* sect. *Phacocystis*, is more distantly related to *C. aquatilis* and *C. subspathacea* [294,295]; this is reflected in its distinct *matK* barcode. *Carex spectabilis* Dewey and *C. podocarpa* R.Br. ex Richardson (two of 11 species in *Carex* sect. *Scitae* Kükenthal [296]) cluster together but have distinct *matK* haplotypes. *Carex holostoma* Drejer and *C. norvegica* Retz. (*Carex* sect. *Racemosae* G. Don [297]) are distinguished, but their evolutionary relationships are not known as few of the ca. 60 species of *Carex* sect. *Racemosae* have been included in phylogenetic studies. *Carex bicolor* Bellardi ex All. and *C. garberi* Fernald (*Carex* sect. *Bicolores* (Tuck. ex L.H. Bailey) Rouy [298]) have distinct barcodes, as do *C. vaginata* Tausch and *C. livida* (Wahlenb.) Willd. (*Carex* sect. *Paniceae* G. Don [299]). 

Other species classified in multiple sections are also distinguished with the barcode data, including *C. rariflora* (Wahlenb.) Sm. (*Carex* sect. *Limosae* (Heuffel) Meinshauser [300]), *C. scirpoidea* Michx. (*Carex* sect. *Scirpinae* (Tuckerman) Kükenthal [301]), *C. concinna* R.Br. ex Richardson (*Carex* sect. *Clandestinae* G.Don [302]), *C. supina* Willd. ex Wahlenb., and *C. glacialis* Mack. (*Carex* sect. *Lamprochlaenae* (Drejer) L.H. Bailey [303]). *Carex krausei* Boeck. and *C. capillaris* subsp. *fuscidula* (V.I. Krecz. ex T.V. Egorova) Á. Löve & D. Löve (*Carex* sect. *Chlorostachyae* Tuckerman ex Meinshauser [304]), taxa sometimes considered conspecific but currently recognized as species (see 30), are distinguished by a substitution in *rbcL* (their *matK* sequences are identical). *Carex supina* and *C. glacialis* have not been included together in phylogenetic analyses thus their relationships are not known. 

*Carex petricosa* Dewey*, C. fuliginosa* subsp. *misandra* (R. Br.) Nyman and *C. atrofusca* Schkuhr are classified in *Carex* sect. *Aulocystis* Dumortier [305]. Each is readily distinguished by the barcode data, and each species clusters in distinctly separate parts of the neighbour joining tree, suggesting that one or all of these may be misclassified in *Carex* sect. *Aulocystis*. Ball and Mastrogiuseppe [305] noted that *C. atrofusca* may be misplaced to section, as it is the only species in the section with papillose perigynia, and the barcode data support this possibility. Hendrichs et al. [306] included *C. fuliginosa* and *C. atrofusca* in their phylogenetic study, and found their relationships to be unresolved with respect to each other and two other sampled species of *Carex* sect. *Aulocystis*. *Carex petricosa* has not been included in any phylogenetic studies to date. A proper phylogenetic analysis including these taxa and other species of *Carex* sect. *Aulocystis* in the context of the Core *Carex* Clade is needed to resolve their affinities. 

#### Taraxacum

*Taraxacum* is a complicated agamic complex of sexual diploid (10% of species) and apomictic polyploid (90% agamospecies) taxa, in which over 2000 species (microspecies) have been described [307]. Species are classified into 47 sections based on morphological and geographical criteria [308]. A few sections include only sexual diploids, and most include sexual diploids and apomictic polyploids [307,309,310]. *Taraxacum* sections are considered to be either ancestral (mostly sexual, diploid, ancestral morphology) or derived (polyploid or diploid, asexual or sexual, derived morphology) [307], the latter thought to have originated from the ancestral taxa via hybridization and extensive reticulation [310]. Ancestral sections are geographically restricted, occurring in mostly unglaciated regions in west central Asia and the Mediterranean, whereas derived sections are more widespread occurring on coastal Eurasia, major Eurasian mountain ranges and arctic-alpine regions. Apomicts are thought to have originated via autopolyploidy or hybridization among sexual species, although the ancestors of most agamospermous taxa and their evolutionary origins are unknown [310] and it is probable that some or all of the parental lineages are themselves of hybrid origin [311]. Little is known about the interrelationships among sections and their evolutionary histories [307,309,311]. 

Several studies of chloroplast DNA have shown that multiple plastid haplotypes are shared among sections and species of *Taraxacum* (i.e., these sections and species are paraphyletic or polyphyletic), based on variation in *trnL*–*F* (sequence and restriction fragment length polymorphism data) and *psbA–trnH* (sequence data) [309,311,312]. In a large study of plastid variation in *Taraxacum* including 237 species from Europe, Asia, and North America, Witzell [309] found that several morphologically defined sections often had multiple shared haplotypes from up to seven plastid DNA lineages. Similarly, Majeský et al. [313] found five *trnL*–*F* haplotypes in nine microspecies of *Taraxacum* sect. *Taraxacum* (=*T. officinale* F.H. Wigg. aggregate), of which one was found in seven taxa and one was unique to one microspecies. Based on a lack of congruence between the morphologically defined sections and the plastid data, Kirschner et al. [311] suggested that either *Taraxacum* supraspecific taxonomy is wrong, or the plastid gene tree does not reflect the extensive reticulation that has occurred in the genus. Witzell [309] noted that the maternal lineages in most sections are polyphyletic as a result of repeated hybridization. In addition to shared plastid haplotypes among sections, King [312] found infraspecific plastid variation with restriction enzymes in non-native asexual microspecies sampled from North America (non-Arctic) and Europe, suggesting these microspecies may have multiple origins. Witzell did not find any infraspecific variation in 12 apomicts in which only two individuals were sampled. 

Aside from recently published barcode data for *Taraxacum* species from Churchill, on which Kuzmina et al. [109] did not comment, genetic variation within and among Canadian Arctic dandelions has not previously been studied. The species sampled here include seven native and one introduced species classified in four sections: *T. officinale* (introduced; *Taraxacum* sect. *Ruderalia* Kirschner, H. Øllg. & Štepánek), *T. ceratophorum* (Ledeb.) DC. (*Taraxacum* sect. *Borealia* Hand.-Mazz.), *T. lapponicum* Kihlm. ex Hand.-Mazz. (*Taraxacum* sect. *Spectabilia* Dahlstedt), *T. carneocoloratum* A. Nelson, *T. holmenianum* Sahlin, *T. hyparcticum* Dahlst., *T. phymatocarpum* J. Vahl, and *T. scopulorum* (A. Gray) Rydb. (*Taraxacum* sect. *Arctica* Jurtzev) [314]. In the treatment of *Taraxacum* for the Flora of North America, which we followed, Brouillet [314] recognized 15 species circumscribed broadly (compare this, for example, with Porsild and Cody [23] who recognized 16 species in the continental Northwest Territories and Nunavut). Variation in *rbcL* and *matK* among the multiple *Taraxacum* individuals and taxa sampled here is low, and haplotypes are shared among species (Figure S5). In *rbcL*, we detect four haplotypes: one found in most individuals, one in an individual of *T. lapponicum*, one in an individual of *T. carneocoloratum*, and one in one individual each of *T. ceratophorum* and *T. hyparcticum*. In *matK*, we detect four common haplotypes found in 21, six, nine, and nine individuals, respectively, with each group including two to six taxa. A single individual in two of these groups has a unique haplotype (one of these is from the only individual of *T. officinale* sampled), and two individuals in the largest group each have unique haplotypes. 

*matK* has not been sequenced extensively for dandelions and therefore we cannot compare our sequences with those from other *Taraxacum* lineages. Nevertheless, discovery of multiple haplotypes distributed among three of the four sampled sections of *Taraxacum* is fully consistent with the previous studies that have detected this same plastid DNA pattern across an even greater number of species and sections [309,311,312]. Furthermore, our finding of multiple plastid haplotypes within species (infraspecific variation) is consistent with the results of King [312]. There are several possible explanations for the plastid patterns observed. The infraspecific variation in Arctic *Taraxacum* may reflect multiple origins of species and, by direct implication, multiple origins of the sections. Alternatively, the infraspecific variation may be a function, wholly or in part, of incorrect taxonomy. This may be attributed to incorrect identification of the material that we studied, a subset of which was difficult to identify, or may be a function of the broad species circumscriptions currently in use in North America, which may not accurately reflect the species' evolutionary histories. For example, *T. ceratophorum* and *T. lapponicum* have both been subdivided into numerous microspecies [314], and infraspecific variation may be detected if the microspecies have independent origins. We did not attempt to identify the samples to putative microspecies, as there is no satisfactory treatment available. Several authors have cautioned that correct identification is critically important when attempting to characterize genetic variation and gene flow in *Taraxacum* apomicts [309,315]. 

Another explanation for the observed pattern is that a subset of the Arctic taxa may be parental to some or all of the other taxa. Five of the seven native Arctic taxa are exclusively polyploids and their origins are unknown, *T. holmenianum* is diploid and putatively sexual, and a range of ploidy levels has been documented in *T. ceratophorum*. *Taraxacum ceratophorum* reproduces sexually in Colorado, where all populations studied were diploid [316], but its mode of reproduction in the Arctic is unknown. Three of the four main plastid haplotypes are found in individuals of *T. holmenianum* and in other taxa. The origin of the infraspecific plastid variation in this diploid taxon is unknown. *Taraxacum holmenianum* may be one of the parental taxa of the polyploid species, perhaps representing the maternal lineage from which they have obtained their plastid haplotypes. Plastid variation has similarly been found in a sexual species from Poland (*T. tenuifolium* Koch) [311]. This putative scenario does not explain the presence of the same shared haplotypes in *T. ceratophorum*, which is morphologically distinct from *T. holmenianum* and part of a separate section. Whatever the origins of the plastid variation, the multiple shared haplotypes found in multiple species here and elsewhere suggest that plastid DNA barcodes are of little use for species identification in dandelions. 

#### Minuartia

Our data are consistent with previous studies that found the genus *Minuartia* to be polyphyletic [218,317], as two clusters of species are widely separated in our neighbour joining trees. One cluster includes *M. biflora* (L.) Schinz & Thell. and *M. arctica* (Steven ex Ser.) Graebn., and the other includes two subclusters: one comprises *M. rubella* (Wahlenb.) Hiern, the other comprises the closely related *M. elegans* (Cham. & Schltdl.) Schischk., *M. stricta* (Sw.) Hiern, and *M. rossii* (R.Br. ex Richardson) Graebn. (Figure S11) [318]. Based on the considerable genetic distance between these subclusters, they are probably not closely related. The polyphyletic *Minuartia* has not yet been re-classified pending further sampling of the genus [317]. Arctic species will likely be placed in two or three different genera, which the current barcode data will distinguish. 

Our sampling in *Minuartia* has revealed the misidentification of a sequence used in phylogenetic analyses of Caryophyllaceae [218,317]. The *M. rossii matK* sequence (FJ404849) in these studies was generated from the collection *Gillespie et al. 6726* (CAN) [317], which we sequenced independently. Our *matK* sequence is identical to the published sequence and falls in a cluster with individuals of *M. biflora* (Figure S11), similar to one of the phylogenetic studies [218]. Other individuals of *M. rossii*, however, are part of a distant cluster that also includes *M. stricta* and *M. elegans* (Figure S11). Re-examination of the *Gillespie et al. 6726* voucher specimen revealed the original identification to be an error; the correct identification for the specimen is *M. biflora*. 

### DNA Barcode Data and the Post-Glacial Origins of the Arctic flora

Molecular data from several arctic-alpine species have been studied to identify potential glacial refugia and to reconstruct the post-glacial origins of the Arctic flora (reviewed in 319). Many of these studies have been based on plastid sequence data from intergenic spacer regions, which are thought to evolve more rapidly than protein coding genes. Variation in plastid protein-coding genes such as *matK* and *rbcL* has not thus far been explored in phylogeographic studies of arctic-alpine species. Nevertheless, these genes may be sufficiently variable to also provide insights into the historical biogeography of Arctic and other taxa. If so, sequencing of the core barcode loci for plant taxa from throughout their geographical ranges may provide data informative for this field of research. The data we generated for the Arctic flora, in combination with recent data from other barcoding studies, provide an opportunity to see if the geographical distribution of infraspecific variation in the core barcode loci in arctic-alpine species correlates with geographically structured plastid variation found previously in these taxa.

#### *Oxyria digyna* (L.) Hill

*Oxyria digyna* is an arctic-alpine species widely distributed across the northern hemisphere in which six major plastid lineages have been identified, based on sequence data of the *trnH*–*psbA* and *trnT*–*trnL* intergenic spacers [320,321]. The seven individuals sampled by Allen et al. [321] from the Canadian Arctic were part of a broader Arctic clade that ranges from Greenland to eastern Siberia, and the individuals they sampled from the Northwest Territories and Nunavut—the range of the specimens we sampled here—all had an identical haplotype ("haplotype 3"). We found no variation in *rbcL* (n = 6) or *matK* (n = 8) among the plants we sampled (Figure S39). This is not unexpected, as we likely sampled only one of the plastid lineages in the species. Broader geographical sampling is needed to determine if the known plastid lineages can be detected in *rbcL* and *matK*.

#### *Vaccinium uliginosum* L

Alsos et al. [222] identified three major plastid lineages in the widespread blueberry species *V. uliginosum* that correspond to geography: a Beringian lineage, an amphi-Atlantic lineage, and a widespread arctic-alpine lineage. Nearly all their sampled specimens from the Canadian Arctic were part of the globally widespread arctic-alpine lineage. However, two populations from Churchill were part of the amphi-Atlantic lineage. There is no variation in *rbcL* and *matK* among our Canadian Arctic samples and one boreal sample from Yellowknife, NT (Figure S18). However, *V. uliginosum matK* sequences from a Churchill (Manitoba) collection (JN966729.1 [109]) and a St. John's (Newfoundland and Labrador) collection (AF419717.1, *vander Kloet 217995* [322]), which are identical, differ from our Arctic *matK* sequences (data not shown). These two *matK* haplotypes may represent the amphi-Atlantic and arctic-alpine lineages, respectively. (The provenance of the *vander Kloet 217995* collection in Powell and Kron [322] is given only as 'circumboreal'. The specimen could not be located in the E.C. Smith Herbarium (ACAD), but the collector's field notes indicate the place of collection as Signal Hill, St. John's, 17 Sep 1995 (R. Newell, personal communication, 28 Jan 2013)). *rbcL* sequences for the species have been published for collections from Churchill (JN966056.1 [109]) and Sweden (AF421107.1 [323]). These are identical to those we generated from the Canadian Arctic. No variation in *rbcL* in *V. uliginosum* has thus far been detected.

#### *Saxifraga oppositifolia* (L.)

Considerable research has been conducted on the phylogeography of purple saxifrage (*Saxifraga oppositifolia*), a widely distributed species that occurs throughout the global Arctic and further south in European and North American mountain ranges [324]. Two distinct lineages in the species have been found in studies of RFLPs, plastid sequence data and AFLPs [324–327]. Abbott et al. [325] called these the "Eurasian clade" (the "European-centred clade" of Winkler et al. [327])—from Newfoundland and Labrador and Greenland, across the Atlantic Arctic to the Taymyr Peninsula in northern Siberia, and south in the Pyrenees, Alps, and Tatra Mountains—and the "American-Beringian clade" (the "North American clade" of Winkler et al. [327])—from northern Greenland, west through Beringia to Central Asia and into southern and south-eastern European mountain ranges. Sampling from Canada in these studies was sparse [326,327]; the Eurasian clade is known only from Newfoundland and Labrador and southern Baffin Island, and the American-Beringian clade is known from the High Arctic and south-western Canada. Given the distribution of known populations of the American-Beringian clade, it is reasonable to assume that this lineage extends throughout much of the Canadian Arctic, but its precise limits are unclear. Likewise, the geographical extent of the Eurasian clade in Canada is not known. 

The current barcode data may provide some insight into this. There are two haplotypes represented in the 11 *rbcL* sequences we obtained for *S. oppositifolia*: one in an individual from the Lower Savage Islands, Nunavut (southeast of Baffin Island), and one in the remaining ten individuals, which were all collected in the western Arctic and High Arctic (Ellesmere Island) (Figure S45). Sequences from Churchill, Manitoba, Canada (JN965986) [109] and the United Kingdom (JN891054.1) [98], sampled as part of barcoding studies, represent the former *rbcL* haplotype (data not shown). The two *rbcL* haplotypes in the North American Arctic may represent the two major lineages of *S. oppositifolia*, as they generally correspond to the distributions of the two clades as supported by other plastid markers. Indeed, all plants sampled from the United Kingdom are part of the Eurasian clade, and the putative presence of this lineage on the Savage Islands is consistent with current knowledge of its extent in eastern Canada [324] (the Savage Islands are geographically intermediate between the Baffin Island and the Newfoundland and Labrador populations of the Eurasian clade). If this *rbcL* haplotype represents the Eurasian clade, the distribution of the lineage would be extended considerably westwards in Canada to Churchill, where the haplotype is also present. Broader *rbcL* sampling is needed to confirm whether the variation detected here reflects the previously detected lineages, ideally of the samples used in previous work. Corresponding data for *matK* are not available as we failed to recover *matK* sequences from all but one sample of *S. oppositifolia*, as noted previously. 

## Conclusions

We have generated new plastid DNA barcodes for nearly half of the vascular plant species in the Canadian Arctic ecozone, and 93% of the species in the Canadian Arctic Archipelago. Although the core plastid barcode loci distinguish just over 50% of the sampled Arctic species, this figure does not convey the full utility of a comprehensive DNA barcode reference library for facilitating species identification. Almost all species can readily be assigned to their correct genus, a level of identification that may be sufficient for some purposes (e.g., [107,328]), and in many families the core plastid loci—even *matK* alone—will routinely place an unknown taxon amongst its most similar congeneric species, with the exception of some genera (e.g., *Draba*, *Potentilla*, *Salix, Taraxacum*) where levels of variation are too low to discriminate species, and/or the distribution of barcode haplotypes among individuals is not consistent with species boundaries as they are currently understood. 

The barcode data facilitated identification of taxonomic and identification problems, as the local placement of misidentified specimens in neighbour joining trees was often insightful in helping us make correct morphology-based identifications, even when several closely related taxa shared barcode haplotypes. Detailed examination of the barcode data in a subset of taxa indicated, not unexpectedly, that the ability of the core plastid barcode loci to discriminate species is closely related to the evolutionary relationships of sampled taxa. Such in-depth analysis of barcode data is not included in many barcoding studies. By considering variation in a few exemplar species, we have demonstrated that plastid barcode data may provide major new insights into the biogeographical history of the Arctic flora, when barcode data are available from throughout species' global geographical ranges. 

As only a small fraction of the planet's plant diversity has been sequenced for both of the core plastid barcoding loci, the upper limit for species resolution on a global level with these markers is not yet known. Nevertheless, given the low resolution for these markers in studies that sampled a large number of taxa, including the current one, it is clear that additional loci are necessary to improve discrimination of plant species with DNA barcodes, as recommended by the CBOL Plant Working Group [329]. 

## Supporting Information

Data Set S1 **Voucher specimen information, including the scientific names of taxa sampled, locality information, collection dates, collectors and collection numbers, herbarium accession numbers, and GenBank accession numbers.**
(XLSX)

Table S1 **Species with two or more infraspecific taxa sampled, and the ability of *rbcL*, *matK*, and *rbcL* + *matK* to resolve the conspecific infraspecific taxa.**
(PDF)

Table S2 **Statistics on the recovery of *matK* from samples obtained from silica-gel dried leaf material.**
(PDF)

Table S3 **Ability of the supplementary plastid DNA barcode loci *psbA–trnH*, *psbK–I*** and *atpF–atpH* to discriminate species and infraspecific taxa of *Poa* and *Puccinellia*.
(PDF)

Table S4 **Species from the Canadian Arctic Archipelago for which barcode data were.** not obtained in the current study.
(PDF)

Figure S1 **Frequency histogram showing the distribution of the number of individuals sampled per species.** Putative hybrids are not included.
(PDF)

Figure S2 **Numbers of *rbcL* and *matK* sequences recovered from each plant family.** (A) Families with less than 50 samples recovered per family. (B) Families with more than 50 samples recovered per family.
(PDF)

Figure S3 **Neighbour joining analyses of uncorrected *p*-distances of *rbcL* and *matK* sequence data for Amaranthaceae.** A. *rbcL*. B. *matK*. C. *rbcL* + *matK*.
(PDF)

Figure S4 **Neighbour joining analyses of uncorrected *p*-distances of *rbcL* and *matK* sequence data for Apiaceae.** A. *rbcL*. B. *matK*. C. *rbcL* + *matK*.
(PDF)

Figure S5 **Neighbour joining analyses of uncorrected *p*-distances of *rbcL* and *matK* sequence data for Asteraceae.** A. *rbcL*. B. *matK*. C. *rbcL* + *matK*.
(PDF)

Figure S6 **Neighbour joining analyses of uncorrected *p*-distances of *rbcL* and *matK* sequence data for Betulaceae.** A. *rbcL*. B. *matK*. C. *rbcL* + *matK*.
(PDF)

Figure S7 **Neighbour joining analysis of uncorrected *p*-distances of *matK* sequence data for Boraginaceae.**
(PDF)

Figure S8 **Neighbour joining analyses of uncorrected *p*-distances of *rbcL* and *matK* sequence data for Brassicaceae.** A. *rbcL*. B. *matK*. C. *rbcL* + *matK*.
(PDF)

Figure S9 **Neighbour joining analyses of uncorrected *p*-distances of *rbcL* and *matK* sequence data for Campanulaceae.** A. *rbcL*. B. *matK*. C. *rbcL* + *matK*.
(PDF)

Figure S10 **Neighbour joining analyses of uncorrected *p*-distances of *rbcL* and *matK* sequence data for Caprifoliaceae.** A. *rbcL*. B. *matK*. C. *rbcL* + *matK*.
(PDF)

Figure S11 **Neighbour joining analyses of uncorrected *p*-distances of *rbcL* and *matK* sequence data for Caryophyllaceae.** A. *rbcL*. B. *matK*. C. *rbcL* + *matK*.
(PDF)

Figure S12 **Neighbour joining analyses of uncorrected *p*-distances of *rbcL* and *matK* sequence data for Celastraceae.** A. *rbcL*. B. *matK*. C. *rbcL* + *matK*.
(PDF)

Figure S13 **Neighbour joining analyses of uncorrected *p*-distances of *rbcL* and *matK* sequence data for Cyperaceae.** A. *rbcL*. B. *matK*. C. *rbcL* + *matK*.
(PDF)

Figure S14 **Neighbour joining analyses of uncorrected *p*-distances of *rbcL* and *matK* sequence data for Diapensiaceae.** A. *rbcL*. B. *matK*. C. *rbcL* + *matK*.
(PDF)

Figure S15 **Neighbour joining analysis of uncorrected *p*-distances of *rbcL* sequence data for Dryopteridaceae.**
(PDF)

Figure S16 **Neighbour joining analyses of uncorrected *p*-distances of *rbcL* and *matK* sequence data for Elaeagnaceae.** A. *rbcL*. B. *matK*. C. *rbcL* + *matK*.
(PDF)

Figure S17 **Neighbour joining analysis of uncorrected *p*-distances of *rbcL* sequence data for Equisetaceae.**
(PDF)

Figure S18 **Neighbour joining analyses of uncorrected *p*-distances of *rbcL* and *matK* sequence data for Ericaceae.** A. *rbcL*. B. *matK*. C. *rbcL* + *matK*.
(PDF)

Figure S19 **Neighbour joining analyses of uncorrected *p*-distances of *rbcL* and *matK* sequence data for Fabaceae.** A. *rbcL*. B. *matK*. C. *rbcL* + *matK*.
(PDF)

Figure S20 **Neighbour joining analyses of uncorrected *p*-distances of *rbcL* and *matK* sequence data for Gentianaceae.** A. *rbcL*. B. *matK*. C. *rbcL* + *matK*.
(PDF)

Figure S21 **Neighbour joining analysis of uncorrected *p*-distances of *rbcL* sequence data for Haloragaceae.**
(PDF)

Figure S22 **Neighbour joining analysis of uncorrected *p*-distances of *rbcL* sequence data for Juncaceae.**
(PDF)

Figure S23 **Neighbour joining analyses of uncorrected *p*-distances of *rbcL* and *matK* sequence data for Juncaginaceae.** A. *rbcL*. B. *matK*. C. *rbcL* + *matK*.
(PDF)

Figure S24 **Neighbour joining analyses of uncorrected *p*-distances of *rbcL* and *matK* sequence data for Lentibulariaceae.** A. *rbcL*. B. *matK*. C. *rbcL* + *matK*.
(PDF)

Figure S25 **Neighbour joining analyses of uncorrected *p*-distances of *rbcL* sequence data for Linaceae.**
(PDF)

Figure S26 **Neighbour joining analysis of uncorrected *p*-distances of *rbcL* sequence data for Lycopodiaceae.**
(PDF)

Figure S27 **Neighbour joining analyses of uncorrected *p*-distances of *rbcL* and *matK* sequence data for Melanthiaceae.** A. *rbcL*. B. *matK*. C. *rbcL* + *matK*.
(PDF)

Figure S28 **Neighbour joining analyses of uncorrected *p*-distances of *rbcL* and *matK* sequence data for Menyanthaceae.** A. *rbcL*. B. *matK*. C. *rbcL* + *matK*.
(PDF)

Figure S29 **Neighbour joining analyses of uncorrected *p*-distances of *rbcL* and *matK* sequence data for Montiaceae.** A. *rbcL*. B. *matK*. C. *rbcL* + *matK*.
(PDF)

Figure S30 **Neighbour joining analyses of uncorrected *p*-distances of *rbcL* and *matK* sequence data for Onagraceae.** A. *rbcL*. B. *matK*. C. *rbcL* + *matK*.
(PDF)

Figure S31 **Neighbour joining analyses of uncorrected *p*-distances of *rbcL* and *matK* sequence data for Orchidaceae.** A. *rbcL*. B. *matK*. C. *rbcL* + *matK*.
(PDF)

Figure S32 **Neighbour joining analyses of uncorrected *p*-distances of *rbcL* and *matK* sequence data for Orobanchaceae.** A. *rbcL*. B. *matK*. C. *rbcL* + *matK*.
(PDF)

Figure S33 **Neighbour joining analyses of uncorrected *p*-distances of *rbcL* and *matK* sequence data for Papaveraceae.** A. *rbcL*. B. *matK*. C. *rbcL* + *matK*.
(PDF)

Figure S34 **Neighbour joining analysis of uncorrected *p*-distances of *rbcL* sequence data for Pinales (Pinaceae, Cupressaceae).**
(PDF)

Figure S35 **Neighbour joining analyses of uncorrected *p*-distances of *rbcL* and *matK* sequence data for Plantaginaceae.** A. *rbcL*. B. *matK*. C. *rbcL* + *matK*.
(PDF)

Figure S36 **Neighbour joining analyses of uncorrected *p*-distances of *rbcL* and *matK* sequence data for Plumbaginaceae.** A. *rbcL*. B. *matK*. C. *rbcL* + *matK*.
(PDF)

Figure S37 **Neighbour joining analyses of uncorrected *p*-distances of *rbcL* and *matK* sequence data for Poaceae.** A. *rbcL*. B. *matK*. C. *rbcL* + *matK*.
(PDF)

Figure S38 **Neighbour joining analyses of uncorrected *p*-distances of *rbcL* and *matK* sequence data for Polemoniaceae.** A. *rbcL*. B. *matK*. C. *rbcL* + *matK*.
(PDF)

Figure S39 **Neighbour joining analyses of uncorrected *p*-distances of *rbcL* and *matK* sequence data for Polygonaceae.** A. *rbcL*. B. *matK*. C. *rbcL* + *matK*.
(PDF)

Figure S40 **Neighbour joining analyses of uncorrected p-distances of *rbcL* and *matK* sequence data for Potamogetonaceae.** A. *rbcL*. B. *matK*. C. *rbcL* + *matK*.
(PDF)

Figure S41 **Neighbour joining analyses of uncorrected *p*-distances of *rbcL* and *matK* sequence data for Primulaceae.** A. *rbcL*. B. *matK*. C. *rbcL* + *matK*.
(PDF)

Figure S42 **Neighbour joining analyses of uncorrected *p*-distances of *rbcL* and *matK* sequence data for Ranunculaceae.** A. *rbcL*. B. *matK*. C. *rbcL* + *matK*.
(PDF)

Figure S43 **Neighbour joining analyses of uncorrected *p*-distances of *rbcL* and *matK* sequence data for Rosaceae.** A. *rbcL*. B. *matK*. C. *rbcL* + *matK*.
(PDF)

Figure S44 **Neighbour joining analyses of uncorrected *p*-distances of *rbcL* and *matK* sequence data for Salicaceae.** A. *rbcL*. B. *matK*. C. *rbcL* + *matK*.
(PDF)

Figure S45 **Neighbour joining analyses of uncorrected *p*-distances of *rbcL* and *matK* sequence data for Saxifragaceae.** A. *rbcL*. B. *matK*. C. *rbcL* + *matK*.
(PDF)

Figure S46 **Neighbour joining analyses of uncorrected *p*-distances of *rbcL* and *matK* sequence data for Tofieldiaceae.** A. *rbcL*. B. *matK*. C. *rbcL* + *matK*.
(PDF)

Figure S47 **Neighbour joining analyses of uncorrected *p*-distances of *rbcL* and *matK* sequence data for Typhaceae.** A. *rbcL*. B. *matK*. C. *rbcL* + *matK*.
(PDF)

Figure S48 **Neighbour joining analysis of uncorrected *p*-distances of combined *rbcL + matK* sequence data for *Puccinellia* (Poaceae).**
(PDF)

Figure S49 **Neighbour joining analysis of uncorrected *p*-distances of *psbA–trnH* sequence data for *Puccinellia* and *Poa* (Poaceae).**
(PDF)

Figure S50 **Neighbour joining analysis of uncorrected *p*-distances of *atpF–atpH* sequence data for *Puccinellia* and *Poa* (Poaceae).**
(PDF)

Figure S51 **Neighbour joining analysis of uncorrected *p*-distances of *psbK–psbI* sequence data for *Puccinellia* and *Poa* (Poaceae).**
(PDF)

Figure S52 **Neighbour joining analysis of uncorrected *p*-distances of combined *rbcL + matK* sequence data for *Festuca* (Poaceae).**
(PDF)

Figure S53 **Neighbour joining analysis of uncorrected *p*-distances of combined *rbcL + matK* sequence data for *Poa* (Poaceae).**
(PDF)

Figure S54 **Neighbour joining analysis of uncorrected *p*-distances of combined *rbcL + matK* sequence data for *Pedicularis* (Orobanchaceae).**
(PDF)

Figure S55 **Neighbour joining analysis of uncorrected *p*-distances of combined *rbcL + matK* sequence data for *Salix* (Salicaceae).**
(PDF)

Figure S56 **Neighbour joining analysis of uncorrected *p*-distances of combined *rbcL + matK* sequence data for *Draba* (Brassicaceae).**
(PDF)

Figure S57 **Neighbour joining analysis of uncorrected *p*-distances of combined *rbcL + matK* sequence data for *Saxifraga* (Saxifragaceae).**
(PDF)

Figure S58 **Neighbour joining analysis of uncorrected *p*-distances of combined *rbcL + matK* sequence data for *Ranunculus* (Ranunculaceae).**
(PDF)

Figure S59 **Neighbour joining analysis of uncorrected *p*-distances of combined *rbcL + matK* sequence data for *Potentilla* (Rosaceae).**
(PDF)

Figure S60 **Neighbour joining analysis of uncorrected *p*-distances of combined *rbcL + matK* sequence data for *Carex* (Cyperaceae).**
(PDF)

Figure S61 **Neighbour joining analysis of uncorrected *p*-distances of *matK* sequence data for *Carex* and *Kobresia* (Cyperaceae).** The tree combines data from the current study and previously published data from Le Clerc-Blain et al. [108], the latter prefaced with the project code "CAREX". .
(PDF)
