# Supplementary material for: DNA Barcoding the Canadian Arctic Flora: Core Plastid Barcodes (rbcL + matK) for 490 Vascular Plant Species
Source: PLoS One. 2013 Oct 22;8(10):e77982. doi: 10.1371/journal.pone.0077982 (PMC3865322; doi:10.1371/journal.pone.0077982)
Supplement: Table S1 — Species with two or more infraspecific taxa sampled, and the ability of rbcL, matK, and rbcL + matK to resolve the conspecific infraspecific taxa. (PDF) [file pone.0077982.s002.pdf]

Table S1. Species with two or more infraspecific taxa sampled, and the ability of *rbcL*, *matK* and *rbcL* + *matK* to resolve the conspecific infraspecific taxa.

Family	Taxon	*rbcL*	*matK*	*rbcL* + *matK*
Asteraceae	*Artemisia borealis* Pall. subsp. *borealis*			
	Artemisia borealis subsp. *richardsoniana* (Besser) Korobkov	No	No	No
	Petasites frigidus (L.) Fr. subsp. *frigidus*			
	Petasites frigidus subsp. *palmatus* (Aiton) Cody	No	No	No
Brassicaceae	*Braya glabella* Richardson subsp. *glabella*			
	Braya glabella subsp. *purpurascens* (R. Br.) Cody	No	No	No
Caryophyllaceae	*Silene involucrata* (Cham. & Schltdl.) Bocquet subsp. *involucrata*			
	Silene involucrata subsp. *tenella* (Tolm.) Bocquet	No	No	No
	Silene uralensis (Rupr.) Bocquet subsp. *arctica* (Fr.) Bocquet			
	Silene uralensis subsp. *uralensis*	No	No	No
Cyperaceae	*Carex aquatilis* Wahlenb. var. *aquatilis*			
	Carex aquatilis var. *minor* Boott	No	No	No
	Carex bigelowii Torr. ex Schwein. subsp. *bigelowii*			
	Carex bigelowii subsp. *lugens* (Holm) T.V. Egorova	No	No	No
	Eriophorum scheuchzeri Hoppe subsp. *scheuchzeri*			
	Eriophorum scheuchzeri subsp. *arcticum* M.S. Novos.	No	No	Yes
	Eriophorum vaginatum L. subsp. *vaginatum*			
	Eriophorum vaginatum subsp. *spissum* (Fernald) Hultén	No	No	No
Equisetaceae	*Equisetum arvense* L. subsp. *alpestre* (Wahlenb.) Schönsw. & Elven			
	Equisetum arvense L. subsp. *arvense*	No	–	–
Fabaceae	*Oxytropis borealis* DC. var. *borealis*			
	Oxytropis borealis var. *viscida* (Nutt.) S.L. Welsh	No	Yes	Yes
	Oxytropis deflexa (Pall.) DC. subsp. *foliolosa* (Hook.) Cody			
	Oxytropis deflexa var. *sericea* Torr. & A. Gray	No	No	No
Gentianaceae	*Gentianella propinqua* (Richardson) J.M. Gillett subsp. *arctophila* (Griseb.) Tzvelev			
	Gentianella propinqua subsp. *propinqua*	No	No	No
	Lomatogonium rotatum (L.) Fr. ex Nyman subsp. *rotatum*			
	Lomatogonium rotatum subsp. *tenuifolium* (Griseb.) A.E. Porsild	No	No	No
Poaceae	*Arctagrostis latifolia* (R. Br.) Griseb. subsp. *latifolia*			
	Arctagrostis latifolia subsp. *arundinacea* (Trin.) Tzvelev	No	No	No
	Calamagrostis stricta (Timm) Koeler subsp. *inexpansa* (A. Gray) C.W. Greene			
	Calamagrostis stricta subsp. *stricta*	No	No	No
	Elymus alaskanus (Scribn. & Merr.) Á. Löve subsp. *alaskanus*			
	Elymus alaskanus subsp. *hyperarcticus* (Polunin) Á. Löve & D. Löve	No	No	No
	Festuca rubra L. subsp. *arctica* (Hack.) Govor.			
	Festuca rubra subsp. *rubra*	No	No	No
	Hordeum jubatum L. subsp. *intermedium* Bowden			
	Hordeum jubatum subsp. *jubatum*	No	No	No
	Poa hartzii Gand. subsp. *hartzii*			
	Poa hartzii subsp. *vrangelica* (Tzvelev) R.J. Soreng & L.J. Gillespie	No	No	No
	Poa pratensis L. subsp. *alpigena* (Lindm.) Hiitonen			

	Poa pratensis subsp. *colpodea* (Th. Fr.) Tzvelev			
	Poa pratensis subsp. *irrigata* (Lindm.) H. Lindb.	No	No	No
	Puccinellia tenella (Lange) Holmb. subsp. *langeana* (Berlin) Tzvelev			
	Puccinellia tenella subsp. *tenella*	No	No	No
Potamogetonaceae	*Stuckenia filiformis* (Pers.) Börner subsp. *alpina* (Blytt) R.R. Haynes, Les & M. Král			
	Stuckenia filiformis subsp. *filiformis*			
	Stuckenia filiformis var. *borealis* (Raf.) H. St. John			
	Stuckenia filiformis subsp. *occidentalis* (J.W. Robbins) R.R. Haynes, Les & M. Král	No[1]	No	No
Saxifragaceae	*Saxifraga flagellaris* Willd. subsp. *platysepala* (Trautv.) A.E. Porsild			
	Saxifraga flagellaris subsp. *setigera* (Pursh) Tolm.	No	No	No

[1]*rbcL* distinguishes *Stuckenia filiformis* subsp. *filiformis* from subsp. *alpina*, var. *borealis* and subsp. *occidentalis*.

A dash (–) indicates data are not available for all infraspecific taxa.
