# Supplementary material for: DNA Barcoding the Canadian Arctic Flora: Core Plastid Barcodes (rbcL + matK) for 490 Vascular Plant Species
Source: PLoS One. 2013 Oct 22;8(10):e77982. doi: 10.1371/journal.pone.0077982 (PMC3865322; doi:10.1371/journal.pone.0077982)
Supplement: Table S2 — Statistics on the recovery of matK from samples obtained from silica-gel dried leaf material. (PDF) [file pone.0077982.s003.pdf]

Table S2. Statistics on the recovery of *matK* from samples obtained from silica-gel dried leaf material.

Family	Total number of specimens sampled from silica	Number of *matK* sequences recovered	Number of failed *matK* sequences	*matK* failure (%)
Amaranthaceae	3	3	0	0.0
Araceae	1	1	0	0.0
Asteraceae	106	104	2	1.9
Betulaceae	9	9	0	0.0
Boraginaceae	2	2	0	0.0
Brassicaceae	127	116	11	8.7
Campanulaceae	4	4	0	0.0
Caryophyllaceae	67	64	3	4.5
Celastraceae	7	6	1	14.3
Cupressaceae	2	0	2	100.0
Cyperaceae	133	103	30	22.6
Diapensiaceae	1	1	0	0.0
Dryopteridaceae	9	0	9	100.0
Elaeagnaceae	3	3	0	0.0
Equisetaceae	19	0	19	100.0
Ericaceae	52	47	5	9.6
Fabaceae	47	44	3	6.4
Gentianaceae	3	3	0	0.0
Haloragaceae	4	2	2	50.0
Juncaceae	35	0	35	100.0
Juncaginaceae	4	4	0	0.0
Lentibulariaceae	6	4	2	33.3
Linaceae	3	2	1	33.3
Lycopodiaceae	4	0	4	100.0
Menyanthaceae	3	3	0	0.0
Onagraceae	11	10	1	9.1
Ophioglossaceae	1	0	1	100.0
Orchidaceae	7	6	1	14.3
Orobanchaceae	59	57	2	3.4
Papaveraceae	38	37	1	2.6
Pinaceae	3	0	3	100.0
Plantaginaceae	8	7	1	12.5
Plumbaginaceae	3	3	0	0.0
Poaceae	310	296	14	4.5
Polygonaceae	22	17	5	22.7
Potamogetonaceae	2	2	0	0.0
Primulaceae	15	12	3	20.0
Ranunculaceae	23	17	6	26.1
Rosaceae	75	74	1	1.3
Salicaceae	72	66	6	8.3
Santalaceae	1	1	0	0.0
**Saxifragaceae**	62	33	29	46.8
**Tofieldiaceae**	10	10	0	0.0
**Typhaceae**	1	1	0	0.0
