# Supplementary material for: DNA Barcoding the Canadian Arctic Flora: Core Plastid Barcodes (rbcL + matK) for 490 Vascular Plant Species
Source: PLoS One. 2013 Oct 22;8(10):e77982. doi: 10.1371/journal.pone.0077982 (PMC3865322; doi:10.1371/journal.pone.0077982)
Supplement: Table S3 — Ability of the supplementary plastid DNA barcode loci psbA–trnH, psbK–I and atpF–atpH to discriminate species and infraspecific taxa of Poa and Puccinellia. (PDF) [file pone.0077982.s004.pdf]

Table S3. Ability of the supplementary plastid DNA barcode loci *psbA-trnH*, *psbK-I*, and *atpF-atpH* to discriminate species and infraspecific taxa of *Poa* and *Puccinellia*.

	psbA-trnH	***psbK-I***	***atpF-atpH***
Poa			
Poa abbreviata subsp. *abbreviata*	Yes	No	No
Poa alpina	Yes	Yes	Yes
Poa ammophila	No	No	Yes
Poa arctica subsp. *arctica*	No	No	No
Poa arctica subsp. *caespitans*	No	No	No
Poa glauca subsp. *glauca*	No	No	No
Poa hartzii subsp. *hartzii*	No	No	No
Poa hartzii subsp. *vrangelica*	No	No	No
Poa pratensis subsp. *alpigena*	No	No	No
Poa pratensis subsp. *colpodea*	Yes	No	No
Total species/taxa	6/10	6/10	6/10
Percent discrimination of species/taxa	50%/38%	17%/10%	33%/20%
Puccinellia			
Puccinellia alaskana	No	No	Yes
Puccinellia andersonii	No	No	No
Puccinellia angustata	No	No	No
Puccinellia arctica	No	No	Yes
Puccinellia bruggemannii	No	No	No
Puccinellia hauptiana	No	No	No
Puccinellia phryganodes	No	No	Yes
Puccinellia pumila	No	No	No
Puccinellia tenella subsp. *tenella*	–	No	–
Puccinellia vahliana	No	no	Yes
Total species	9	10	9
Percent discrimination of species	0%	0%	44%

A dash (–) indicates that sequences were not obtained.
