# Supplementary material for: DNA Barcoding the Canadian Arctic Flora: Core Plastid Barcodes (rbcL + matK) for 490 Vascular Plant Species
Source: PLoS One. 2013 Oct 22;8(10):e77982. doi: 10.1371/journal.pone.0077982 (PMC3865322; doi:10.1371/journal.pone.0077982)
Supplement: Table S4 — Species from the Canadian Arctic Archipelago for which barcode data were. not obtained in the current study. (PDF) [file pone.0077982.s005.pdf]

Table S4. Species from the Canadian Arctic Archipelago for which barcode data were not obtained in the current study.

Family	Species
Brassicaceae	*Draba borealis* DC.
Caryophyllaceae	*Cerastium arvense* L.
Caryophyllaceae	*Cerastium cerastoides* (L.) Britton
Caryophyllaceae	*Sagina nodosa* (L.) Fenzl subsp. *borealis* G.E. Crow
Crassulaceae	*Rhodiola rosea* L.
Cyperaceae	*Carex arctogena* Harry Sm.
Cyperaceae	*Carex microglochin* Wahlenb.
Cyperaceae	*Eriophorum* ×medium Andersson subsp. *album* Cayouette
Fabaceae	*Oxytropis hudsonica* (Greene) Fernald
Fabaceae	*Oxytropis podocarpa* A.Gray
Fabaceae	*Oxytropis terrae-novae* Fernald
Gentianaceae	*Comastoma tenellum* (Rottb.) Toyok.
Orobanchaceae	*Euphrasia disjuncta* Fernald and Wiegand
Plantaginaceae	*Veronica wormskjoldii* Roem. and Schult
Poaceae	×*Pucciphippsia vacillans* (Th. Fr.) Tzvelev
Poaceae	*Deschampsia alpina* (L.) Roem. and Schult.
Ranunculaceae	*Coptis trifolia* (L.) Salisb.
Rosaceae	*Potentilla crantzii* (Crantz) G. Beck ex Fritsch
Rosaceae	*Potentilla uniflora* Ledeb.
Salicaceae	*Salix vestita* Pursh
