# Supplementary figures and images for: DNA Barcoding the Canadian Arctic Flora: Core Plastid Barcodes (rbcL + matK) for 490 Vascular Plant Species
Source: PLoS One. 2013 Oct 22;8(10):e77982. doi: 10.1371/journal.pone.0077982 (PMC3865322; doi:10.1371/journal.pone.0077982)

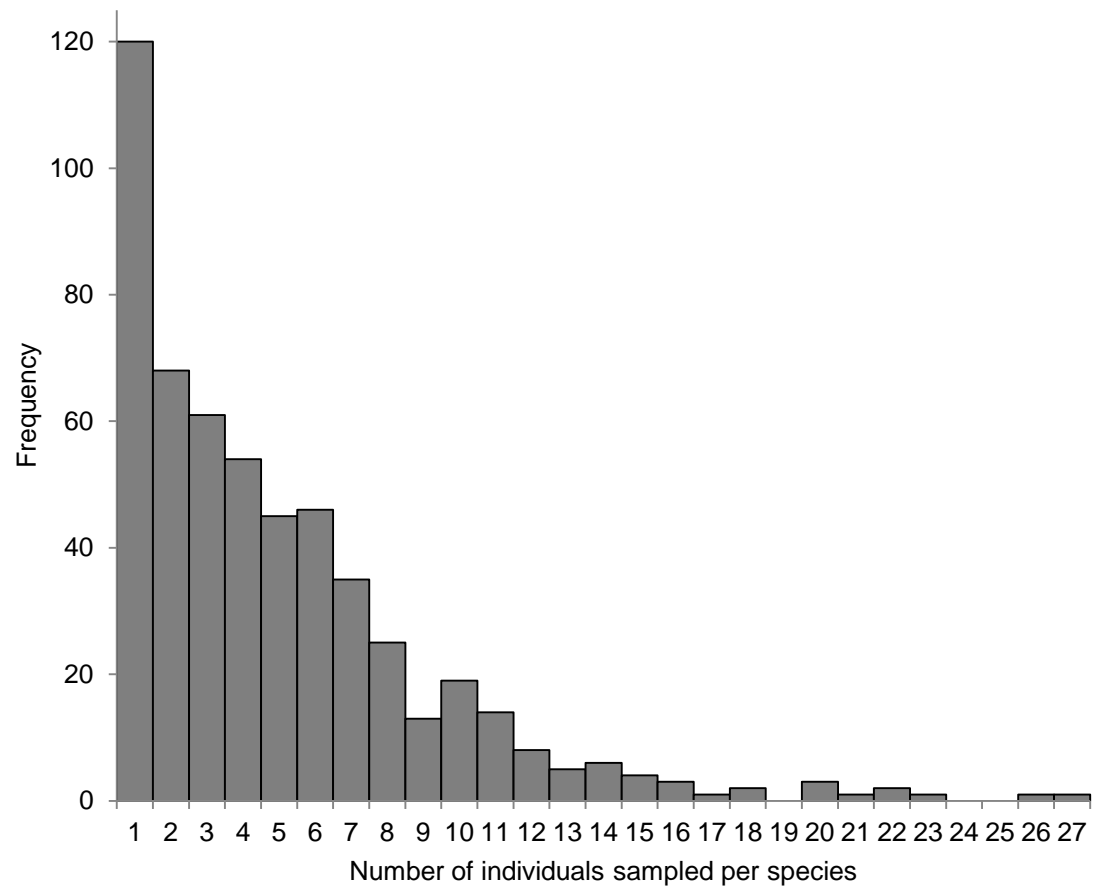

Supplement: Figure S1 — Frequency histogram showing the distribution of the number of individuals sampled per species. Putative hybrids are not included. (PDF) [file pone.0077982.s006.pdf]

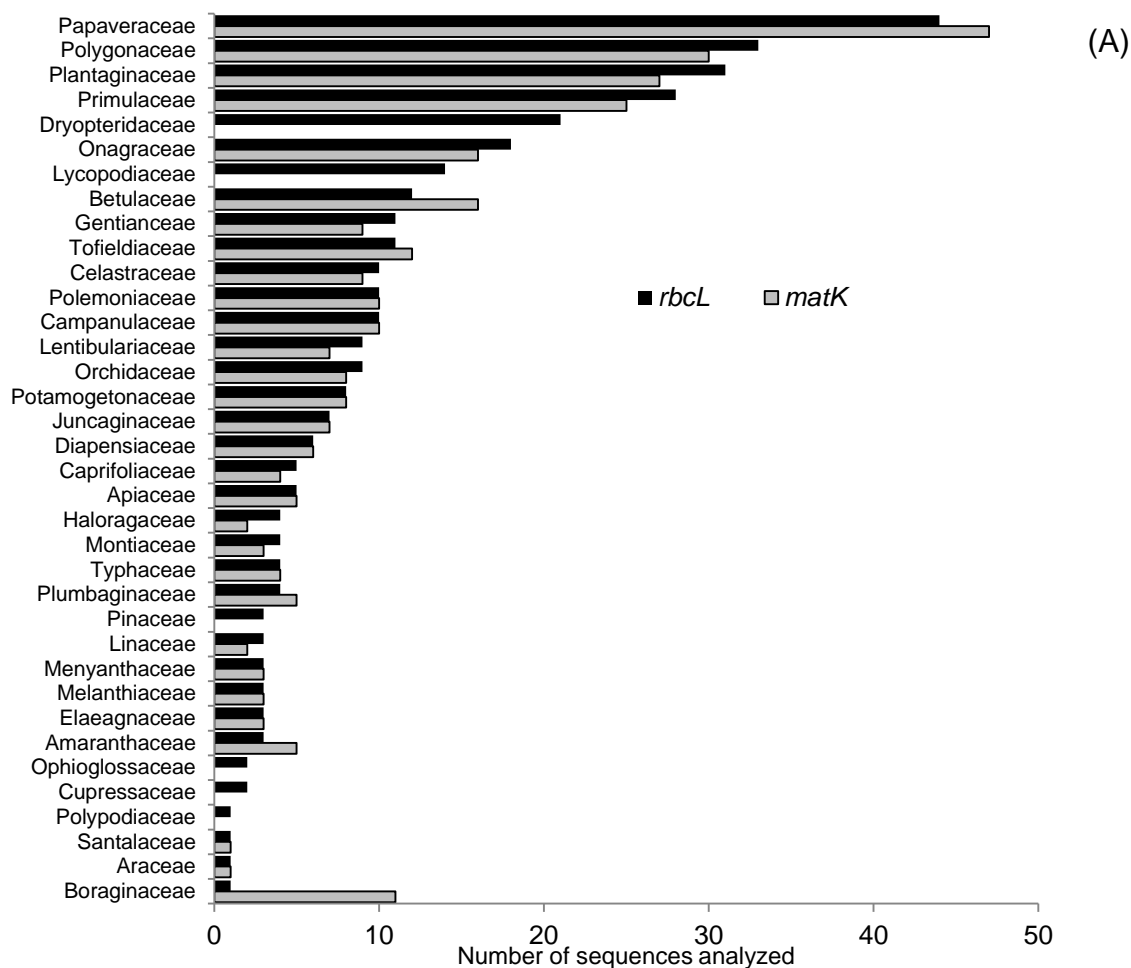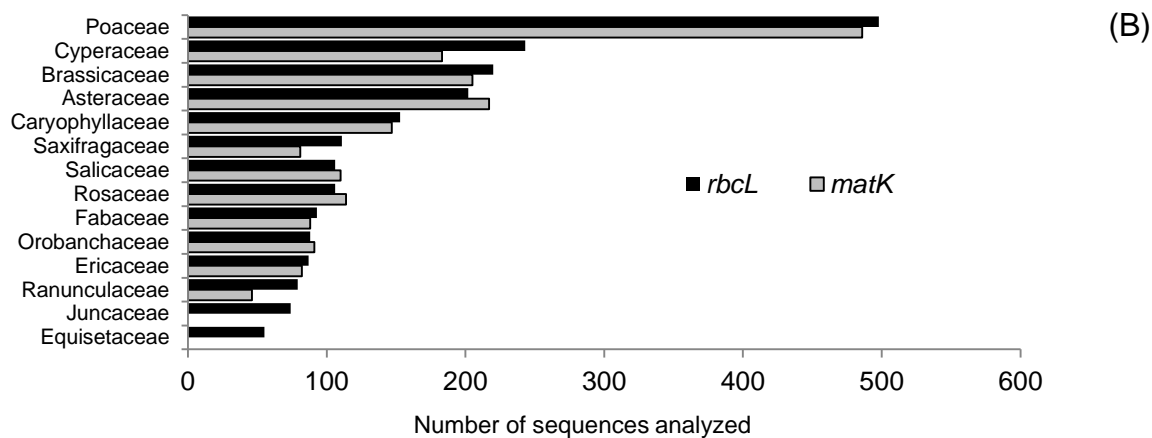

Supplement: Figure S2 — Numbers of rbcL and matK sequences recovered from each plant family. (A) Families with less than 50 samples recovered per family. (B) Families with more than 50 samples recovered per family. (PDF) [file pone.0077982.s007.pdf]

Amaranthaceae

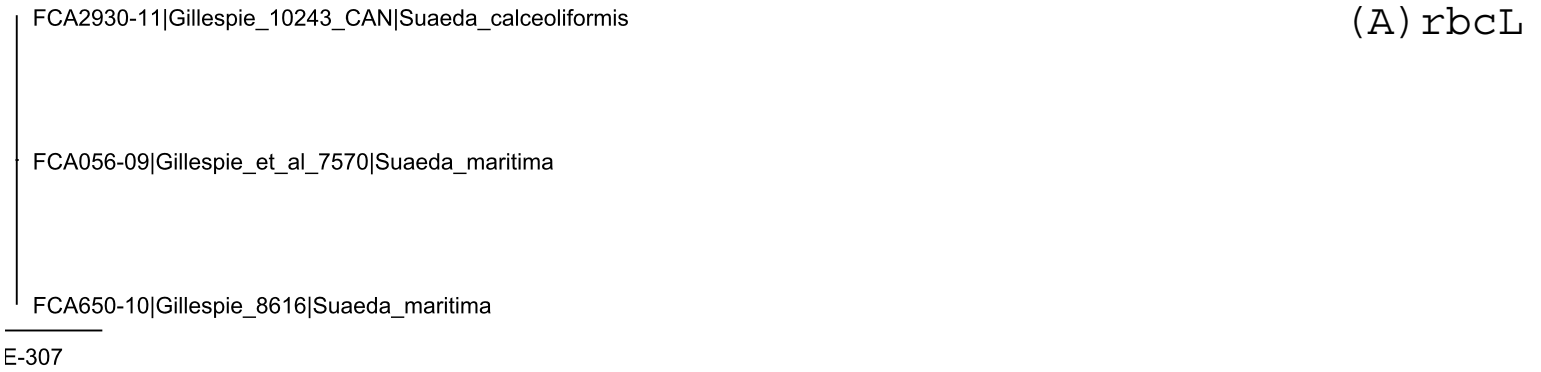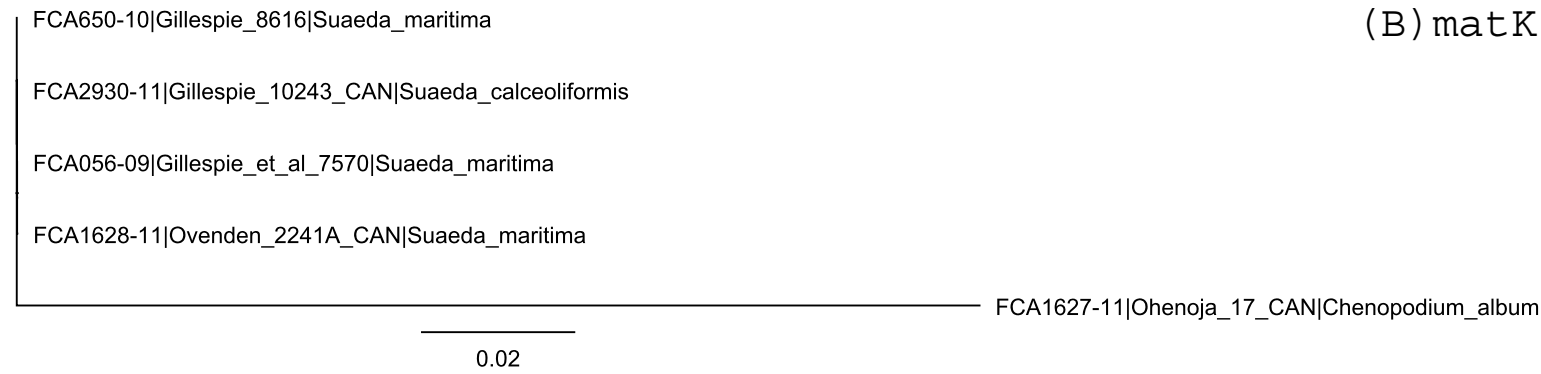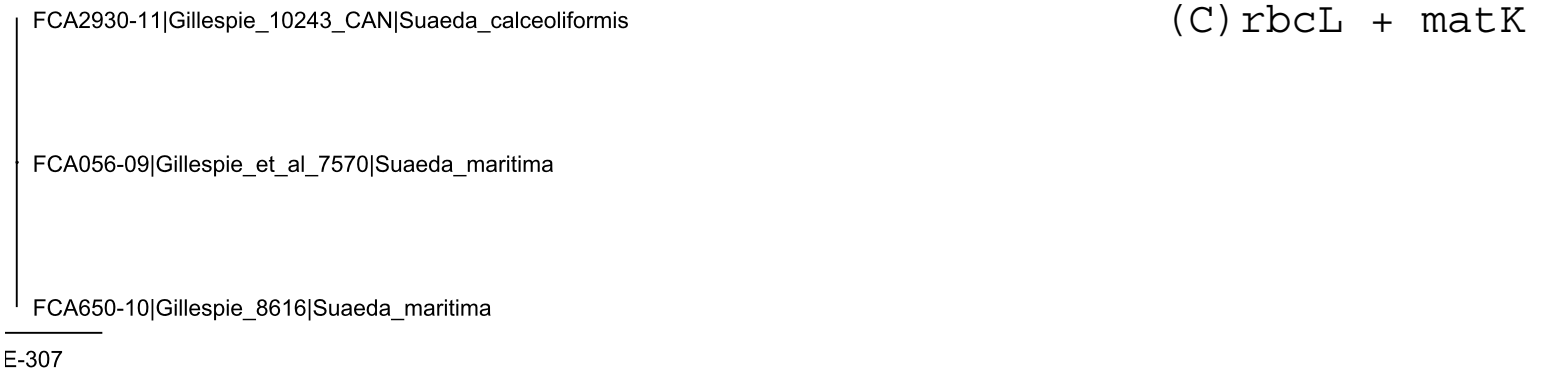

Supplement: Figure S3 — Neighbour joining analyses of uncorrected p-distances of rbcL and matK sequence data for Amaranthaceae. A. rbcL. B. matK. C. rbcL + matK. (PDF) [file pone.0077982.s008.pdf]

Asteraceae

(A)rbclL

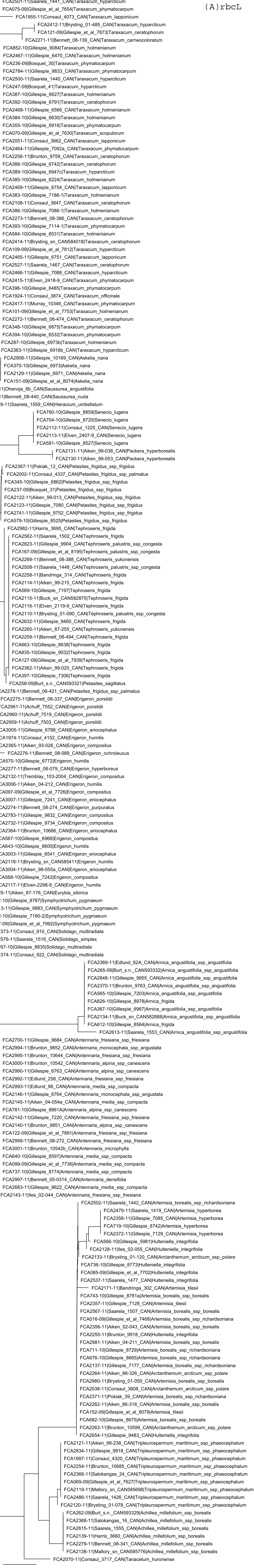

Asteraceae

(B) mat.K

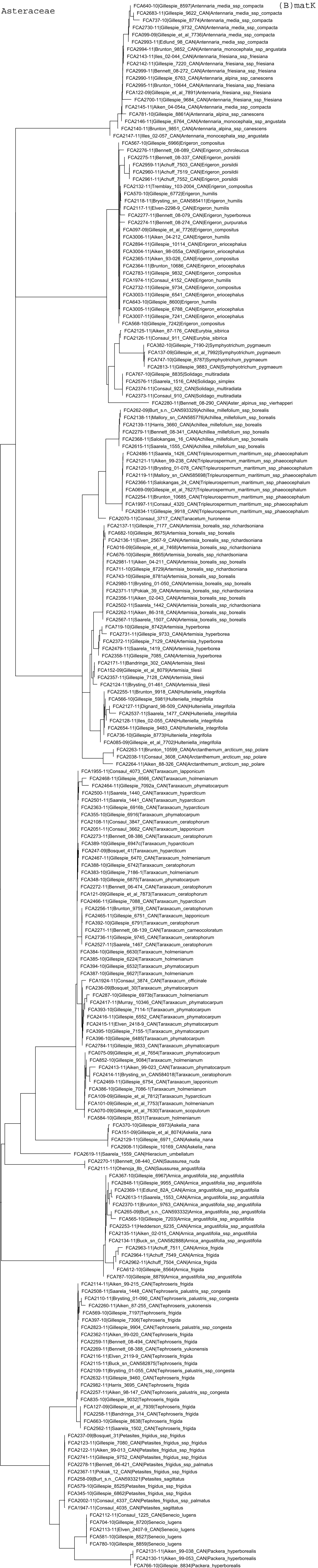

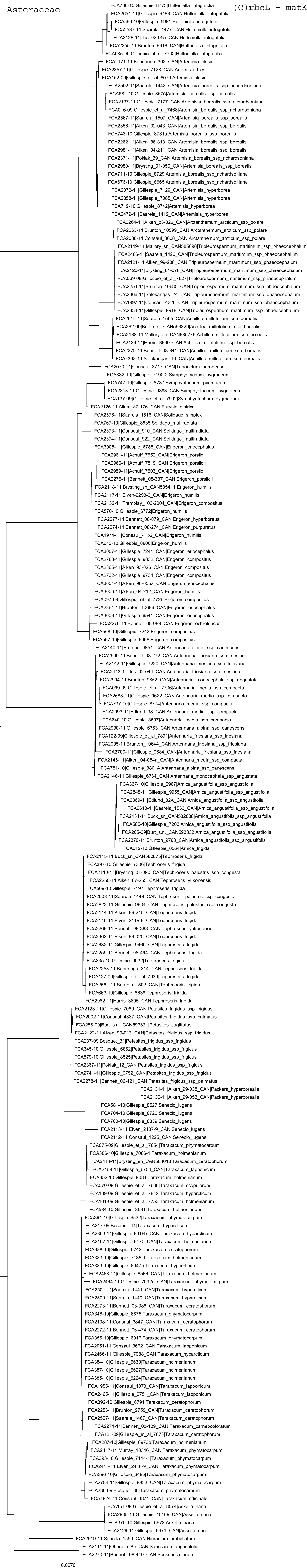

Supplement: Figure S5 — Neighbour joining analyses of uncorrected p-distances of rbcL and matK sequence data for Asteraceae. A. rbcL. B. matK. C. rbcL + matK. (PDF) [file pone.0077982.s010.pdf]

Betulaceae

(A) rbcL

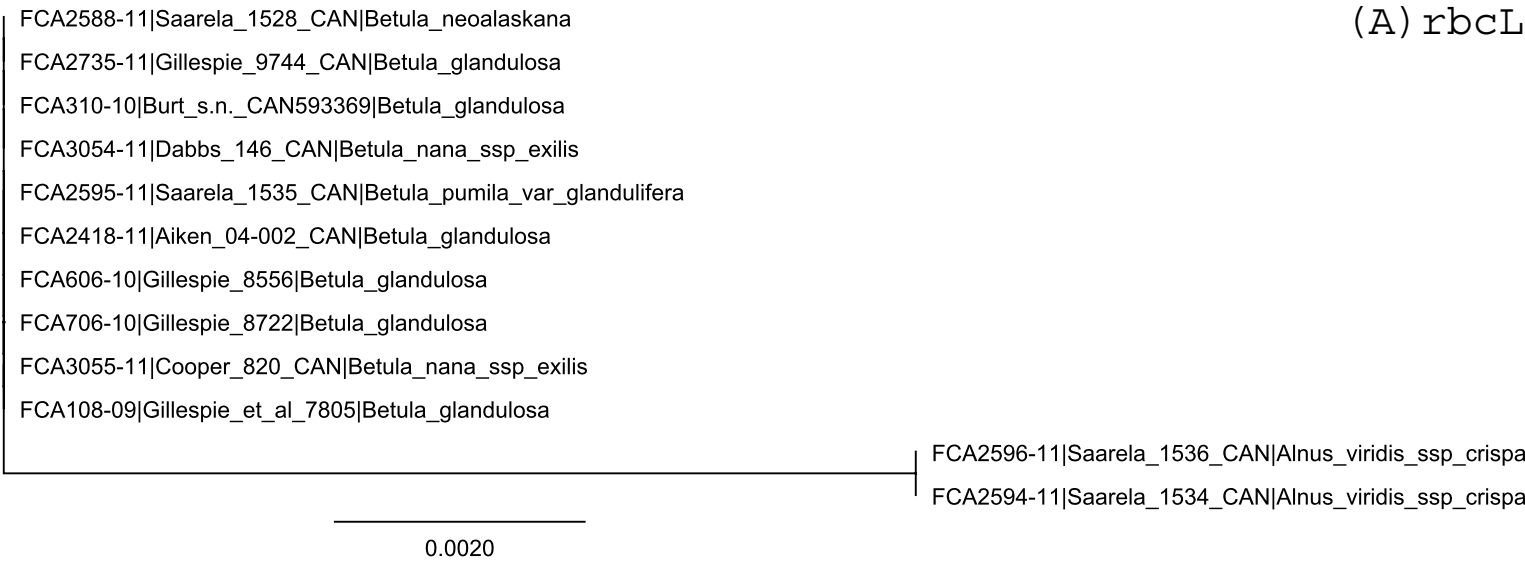

(B) matK

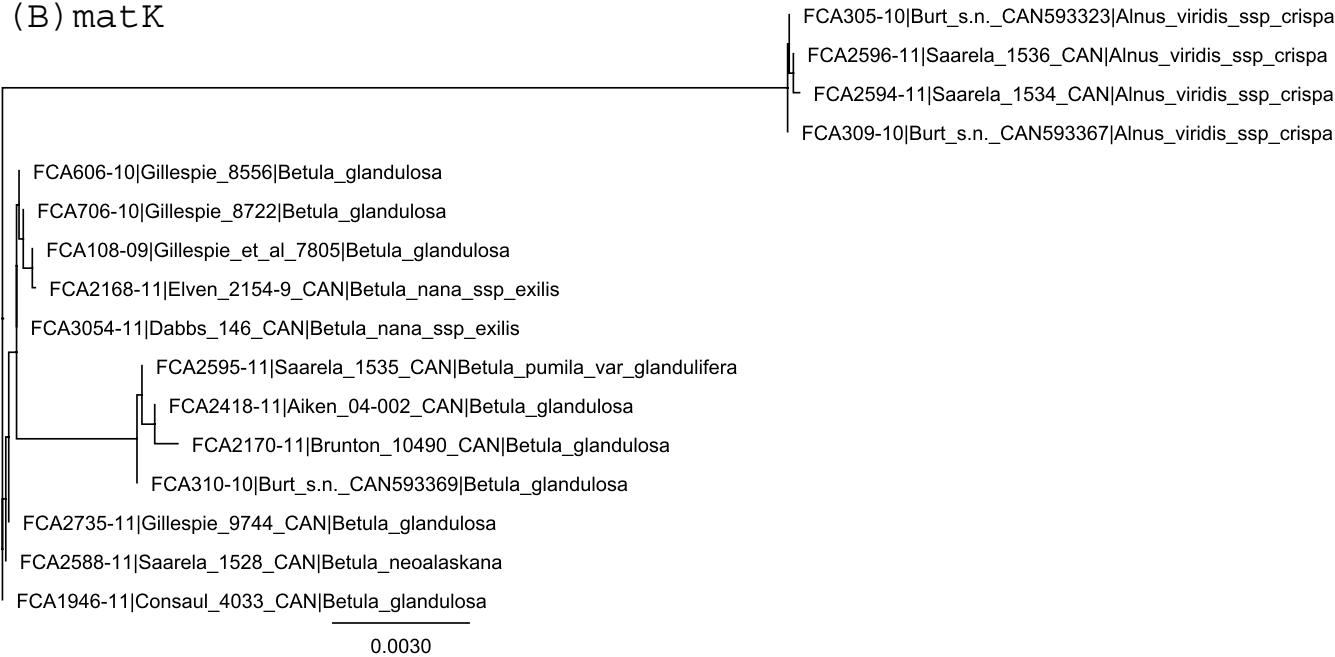

(C) rbcL + matK

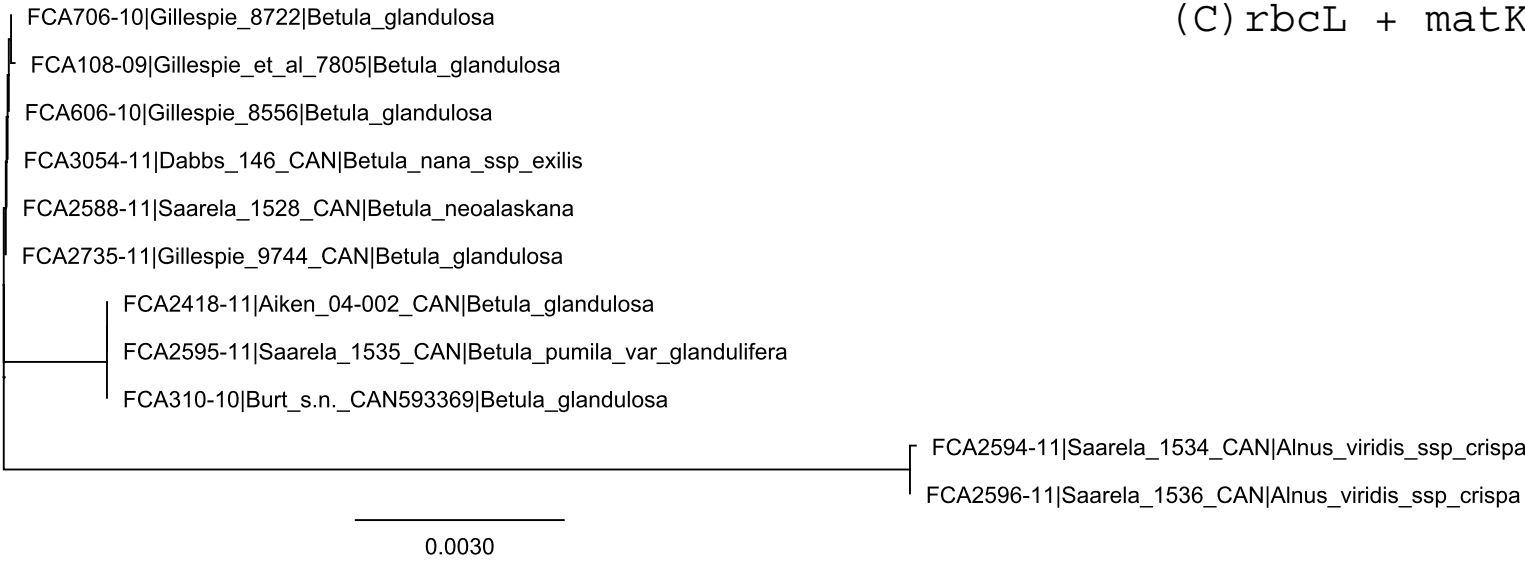

Supplement: Figure S6 — Neighbour joining analyses of uncorrected p-distances of rbcL and matK sequence data for Betulaceae. A. rbcL. B. matK. C. rbcL + matK. (PDF) [file pone.0077982.s011.pdf]

Brassicaceae

(A) rbcL

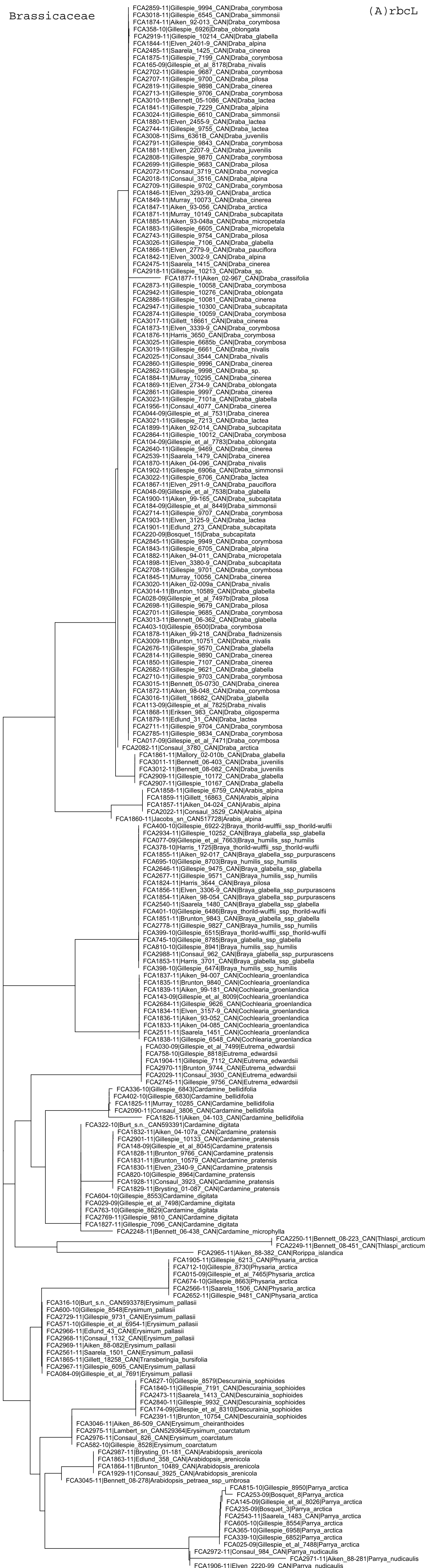

0.0030

Brassicaceae

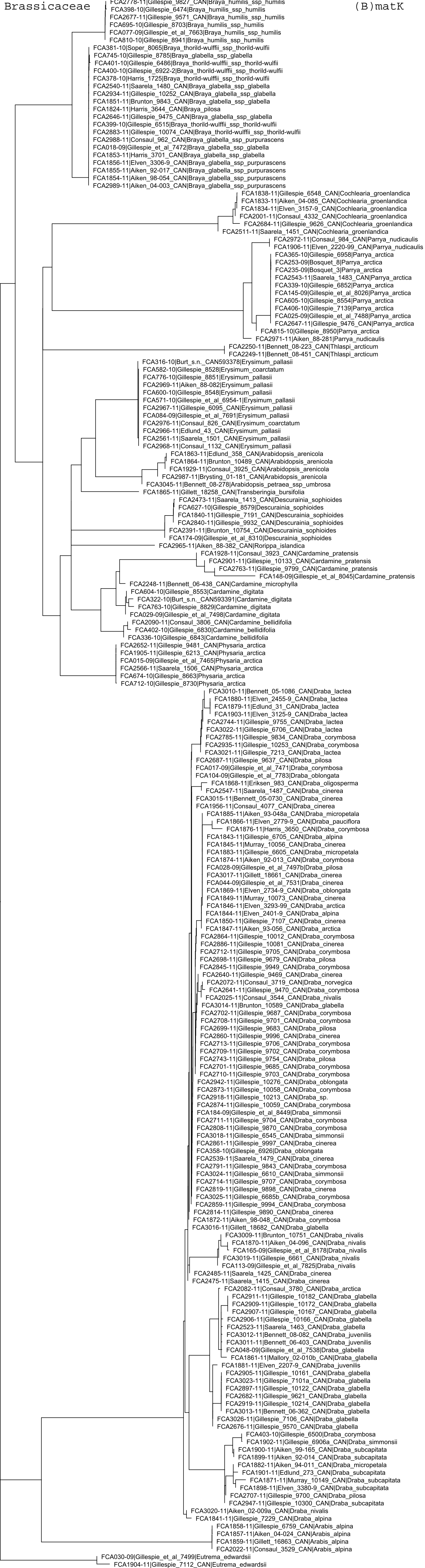

0.0070

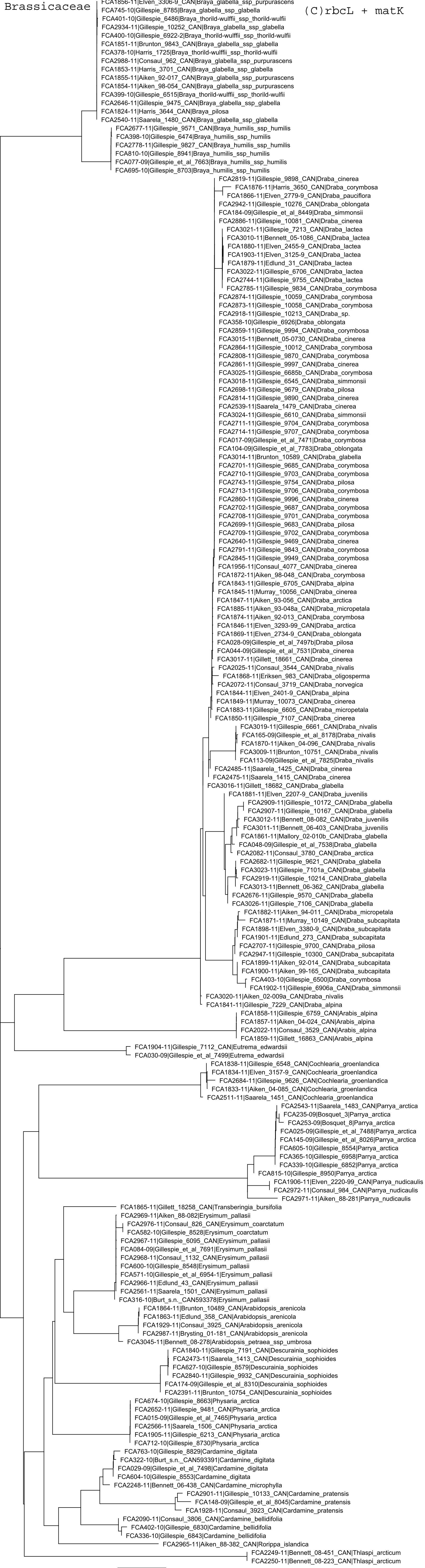

Supplement: Figure S8 — Neighbour joining analyses of uncorrected p-distances of rbcL and matK sequence data for Brassicaceae. A. rbcL. B. matK. C. rbcL + matK. (PDF) [file pone.0077982.s013.pdf]

Campanulaceae

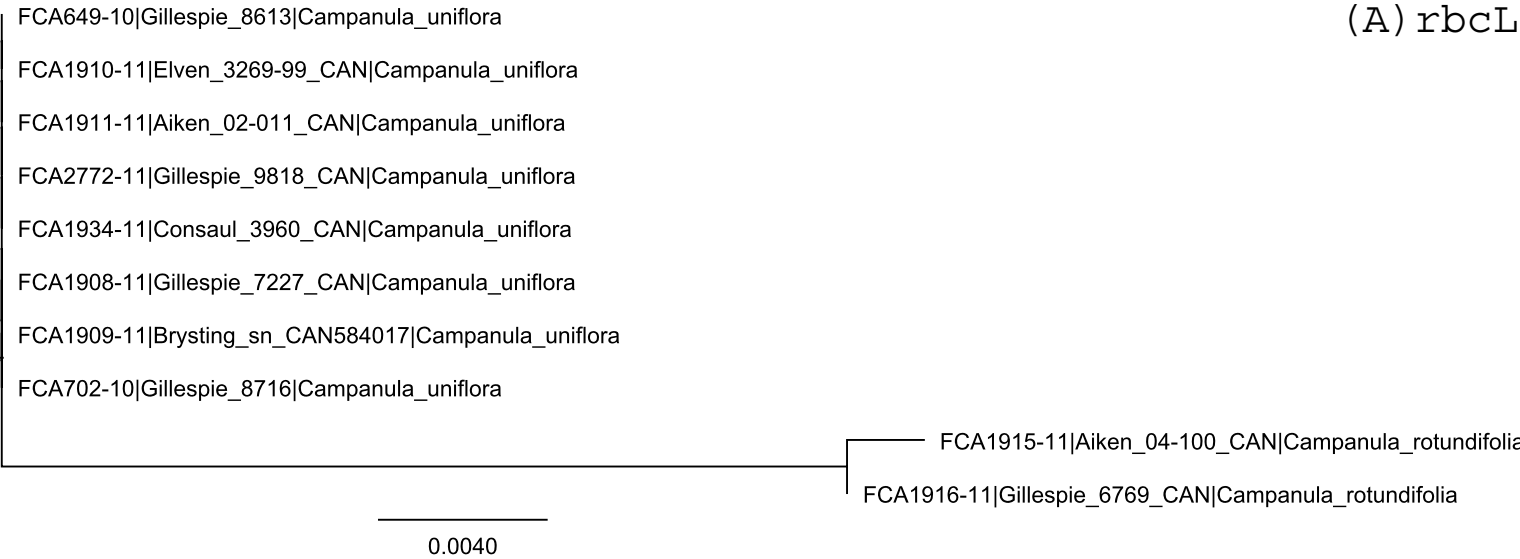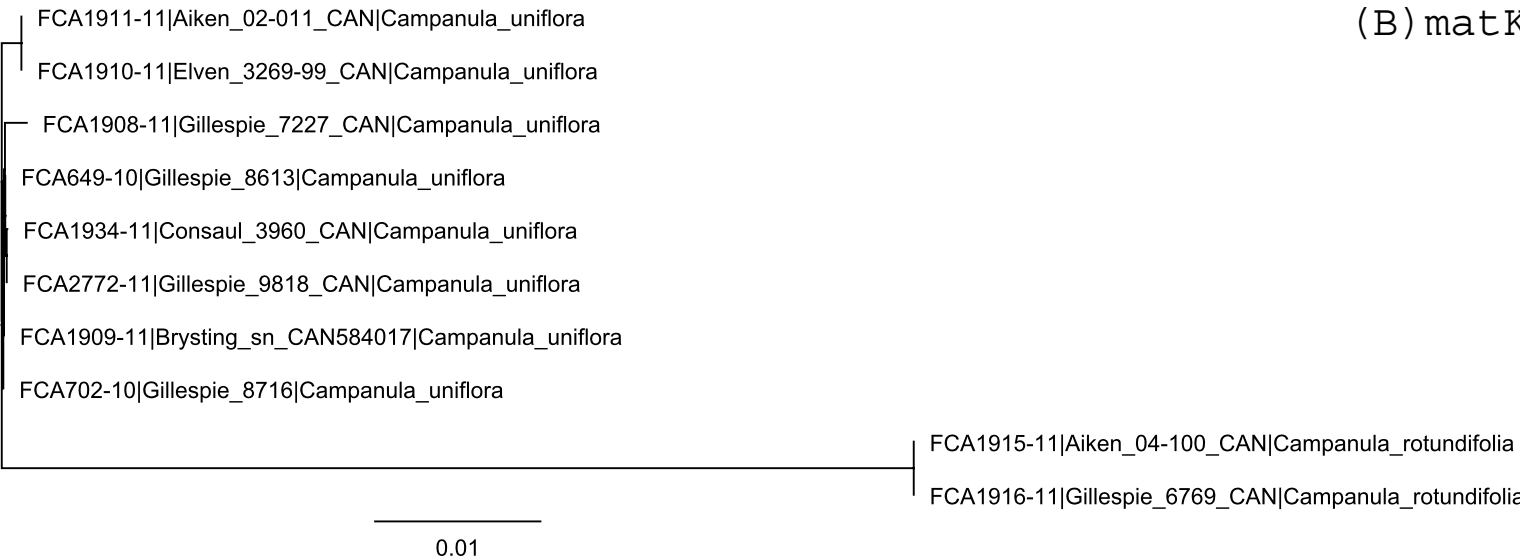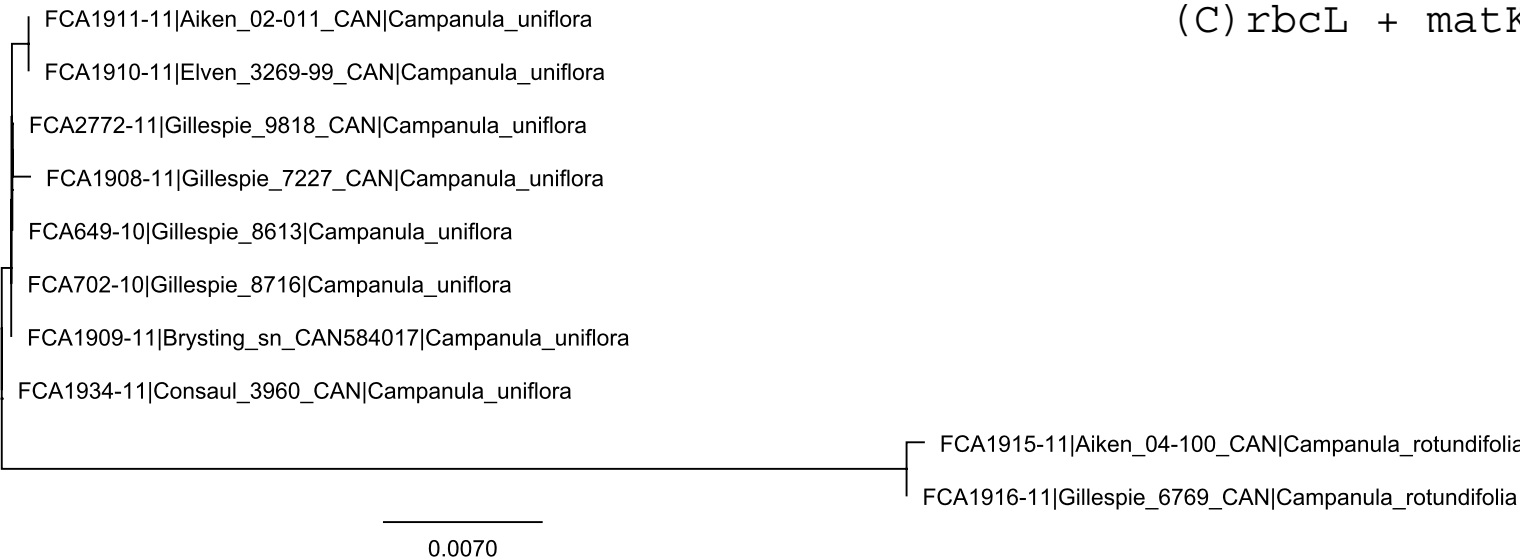

Supplement: Figure S9 — Neighbour joining analyses of uncorrected p-distances of rbcL and matK sequence data for Campanulaceae. A. rbcL. B. matK. C. rbcL + matK. (PDF) [file pone.0077982.s014.pdf]

Caprifoliaceae

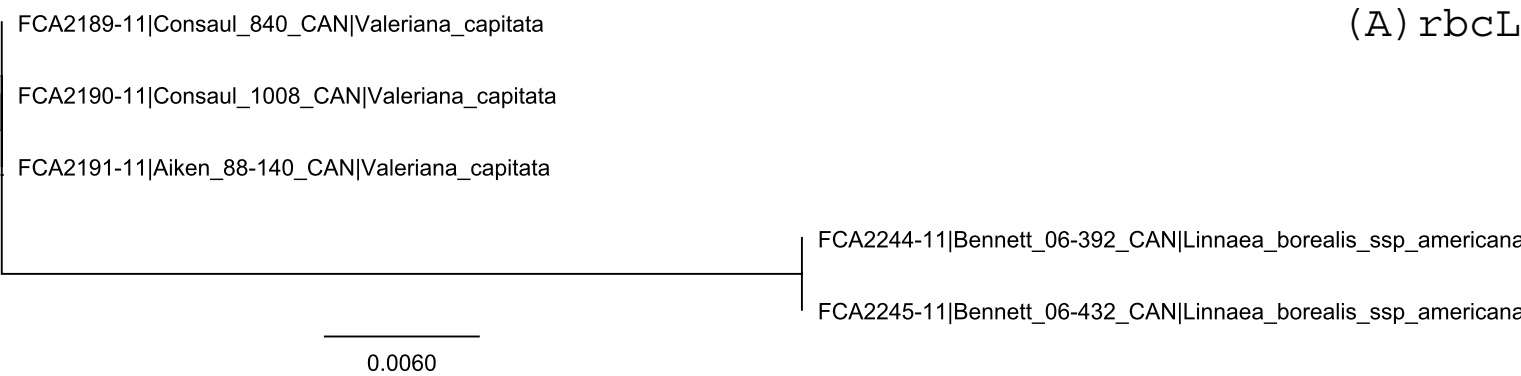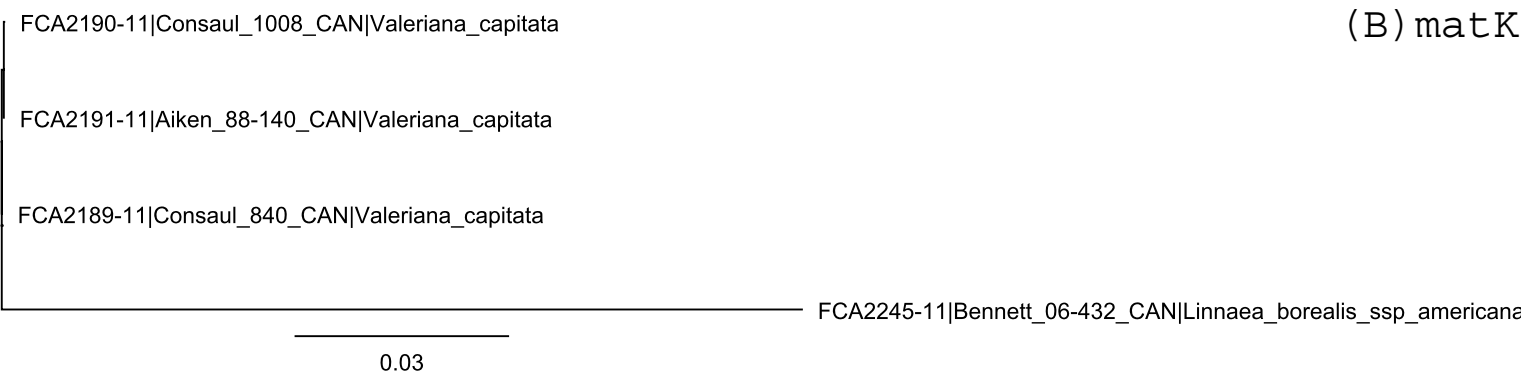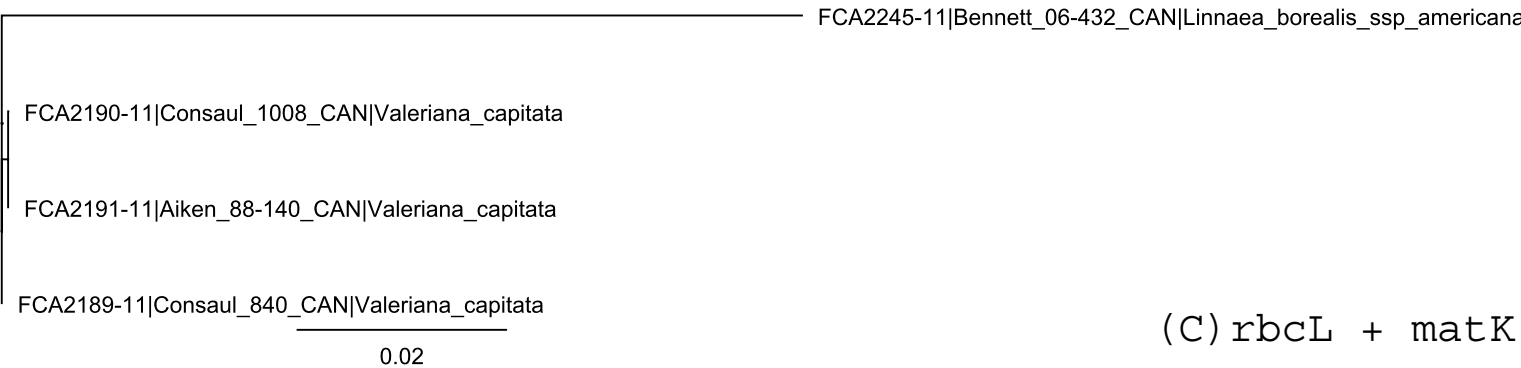

Supplement: Figure S10 — Neighbour joining analyses of uncorrected p-distances of rbcL and matK sequence data for Caprifoliaceae. A. rbcL. B. matK. C. rbcL + matK. (PDF) [file pone.0077982.s015.pdf]

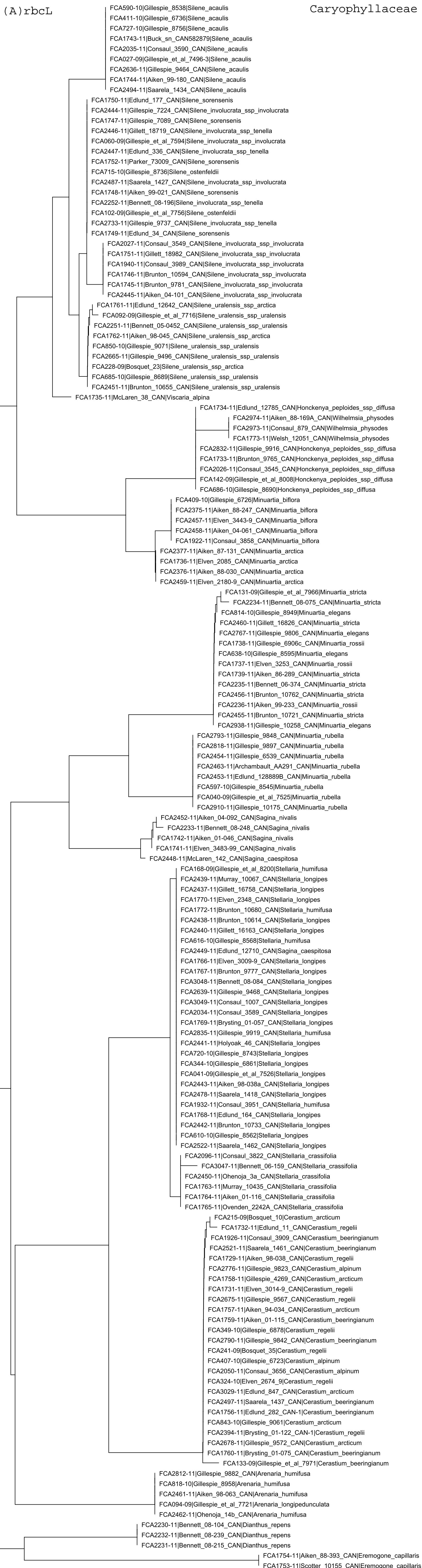

Caryophyllaceae (B)matK

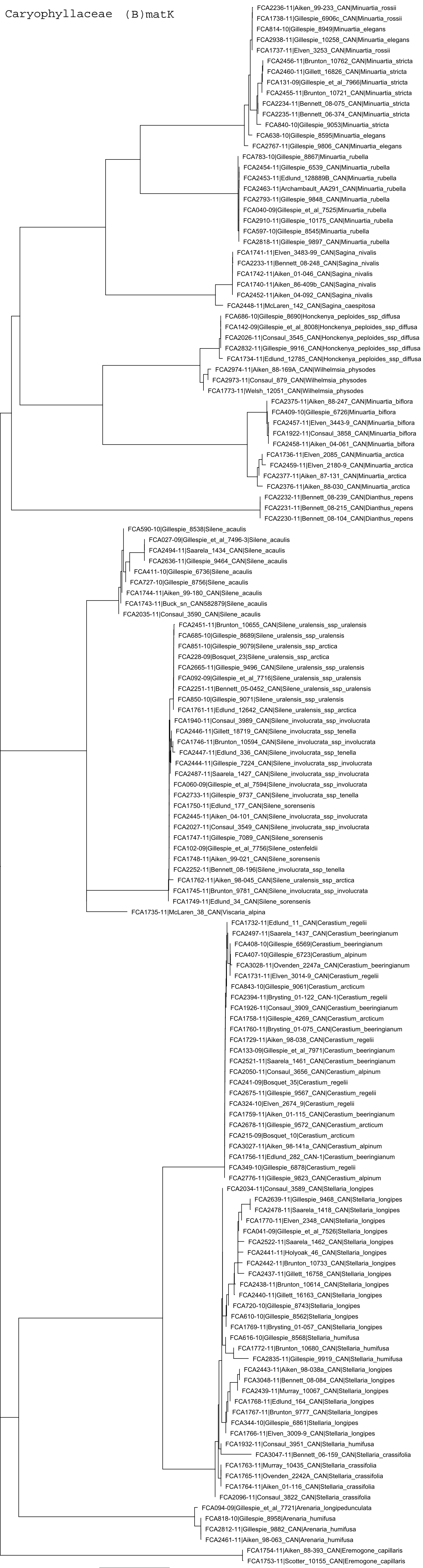

Caryophyllaceae

(C)rbcL + matK

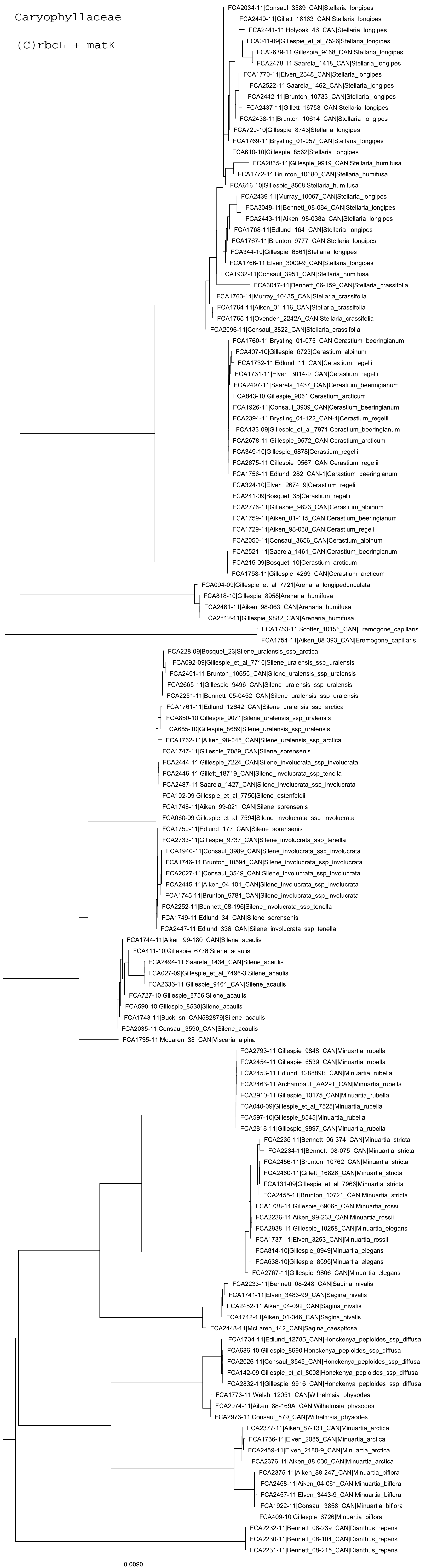

0.0090

Supplement: Figure S11 — Neighbour joining analyses of uncorrected p-distances of rbcL and matK sequence data for Caryophyllaceae. A. rbcL. B. matK. C. rbcL + matK. (PDF) [file pone.0077982.s016.pdf]

Celastraceae

(A) rbcL

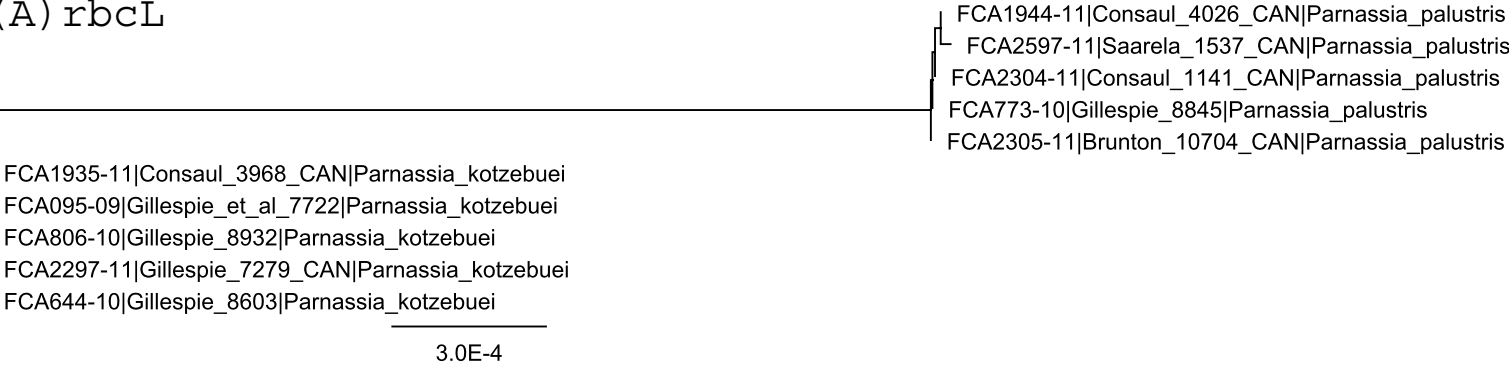

(B) matK

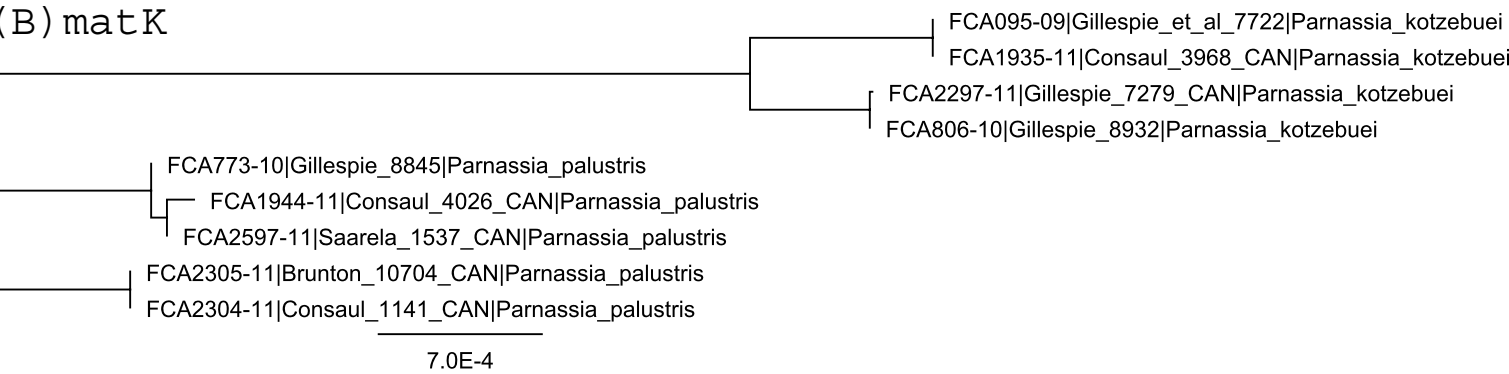

(C) rbcL + matK

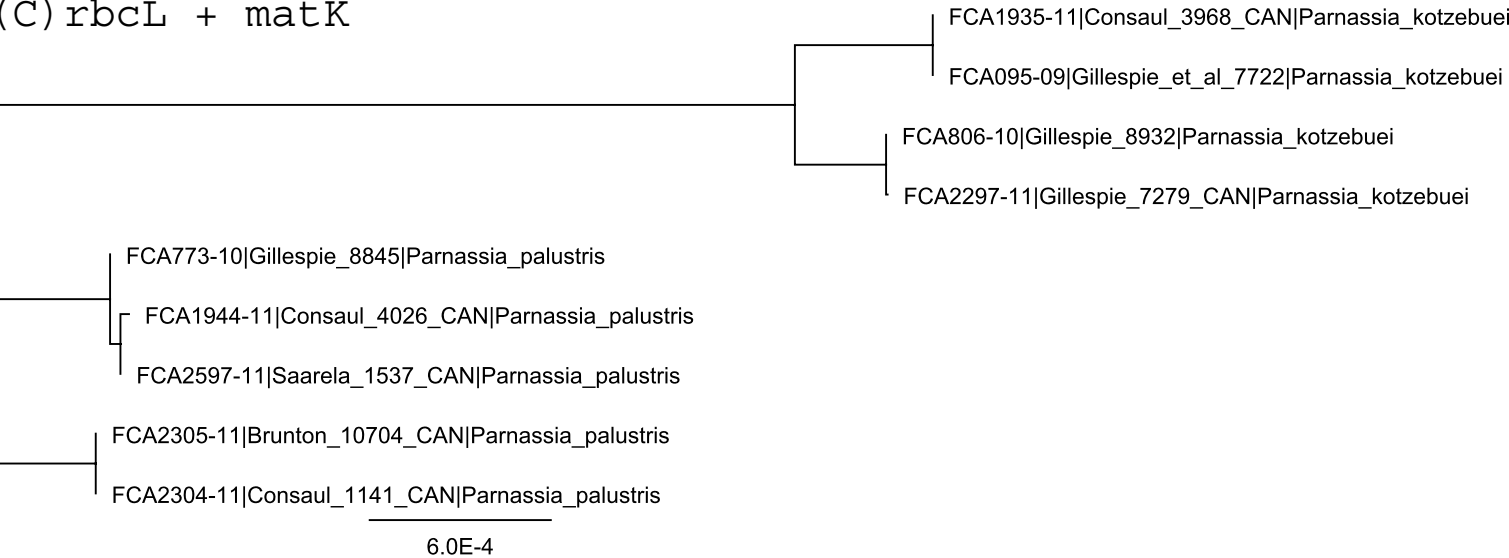

Supplement: Figure S12 — Neighbour joining analyses of uncorrected p-distances of rbcL and matK sequence data for Celastraceae. A. rbcL. B. matK. C. rbcL + matK. (PDF) [file pone.0077982.s017.pdf]

# Diapensiaceae

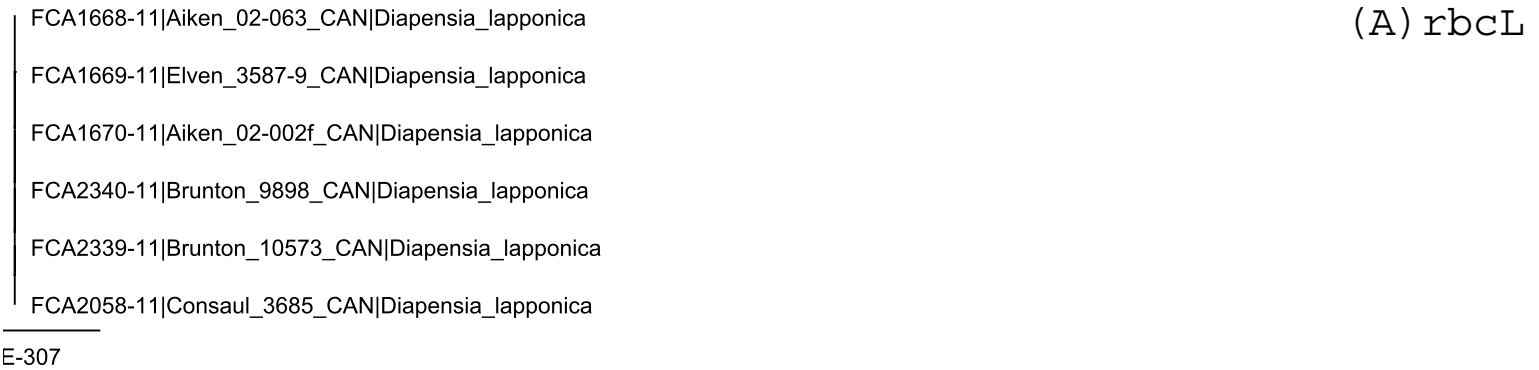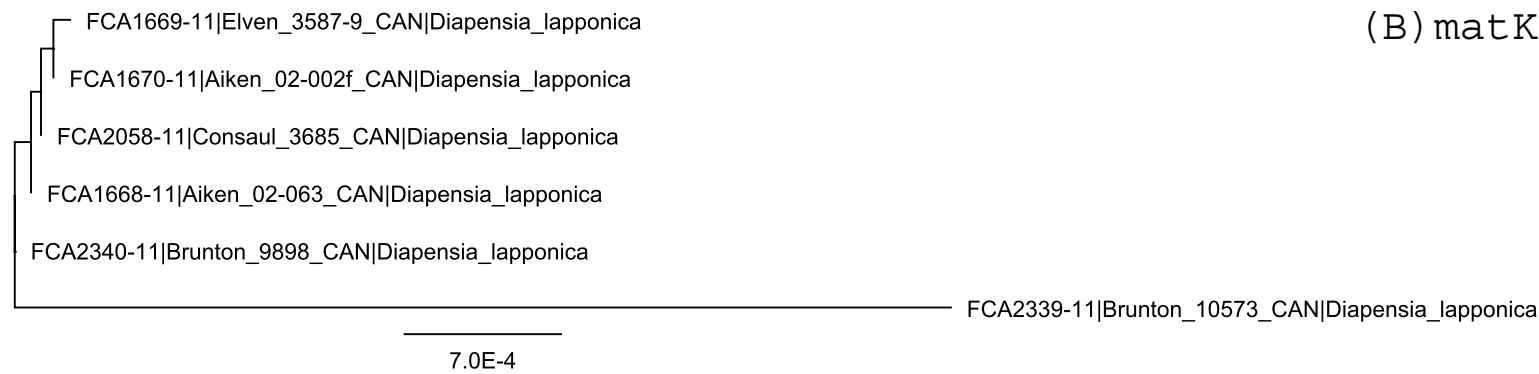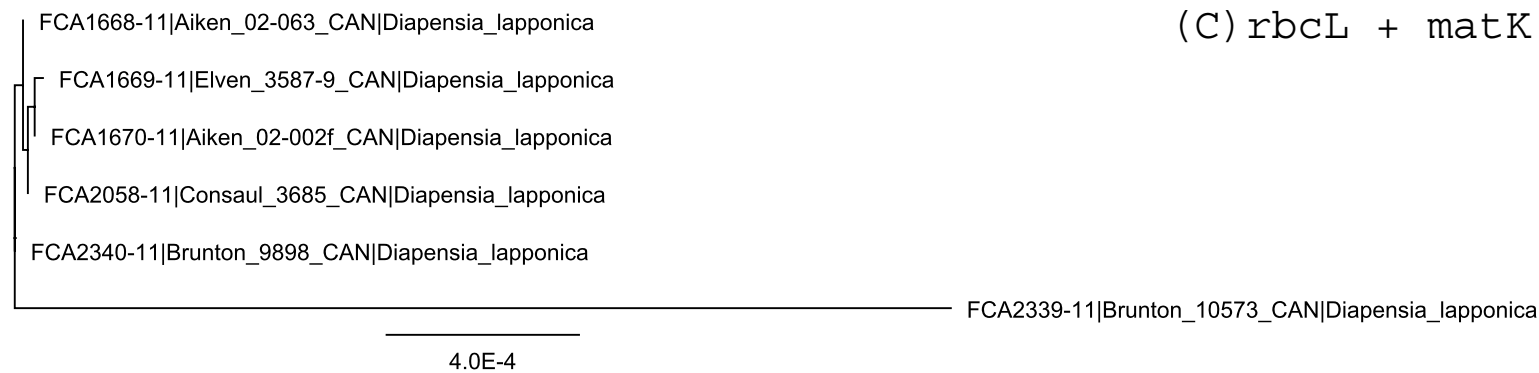

Supplement: Figure S14 — Neighbour joining analyses of uncorrected p-distances of rbcL and matK sequence data for Diapensiaceae. A. rbcL. B. matK. C. rbcL + matK. (PDF) [file pone.0077982.s019.pdf]

Dryopteridaceae      rbcL

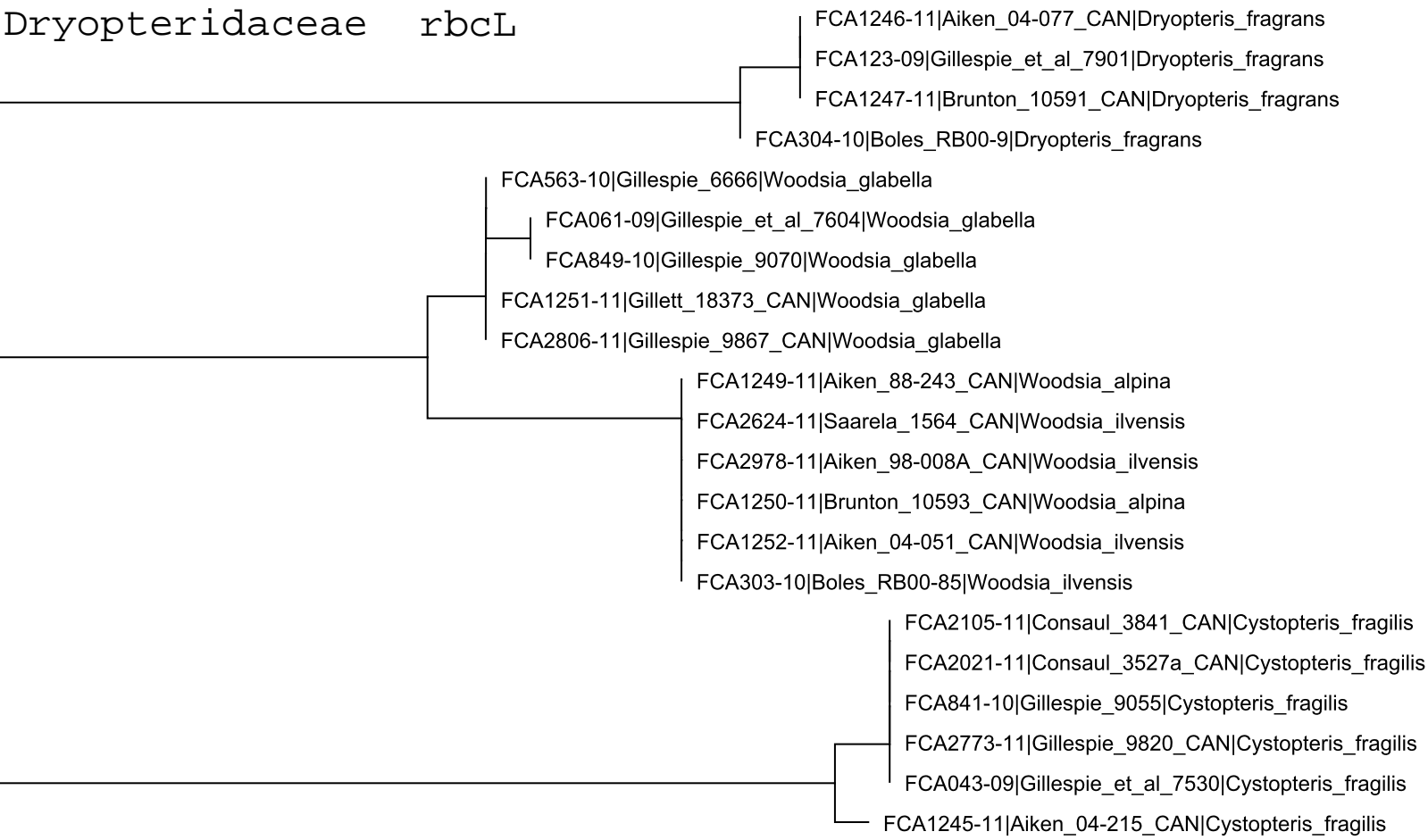

0.0060

Supplement: Figure S15 — Neighbour joining analysis of uncorrected p-distances of rbcL sequence data for Dryopteridaceae. (PDF) [file pone.0077982.s020.pdf]

Elaeagnaceae

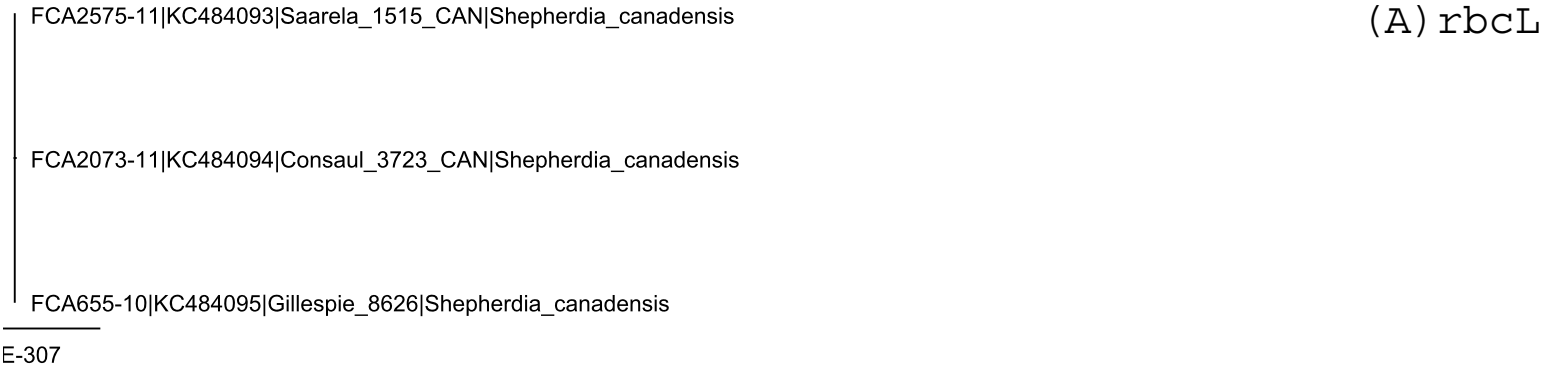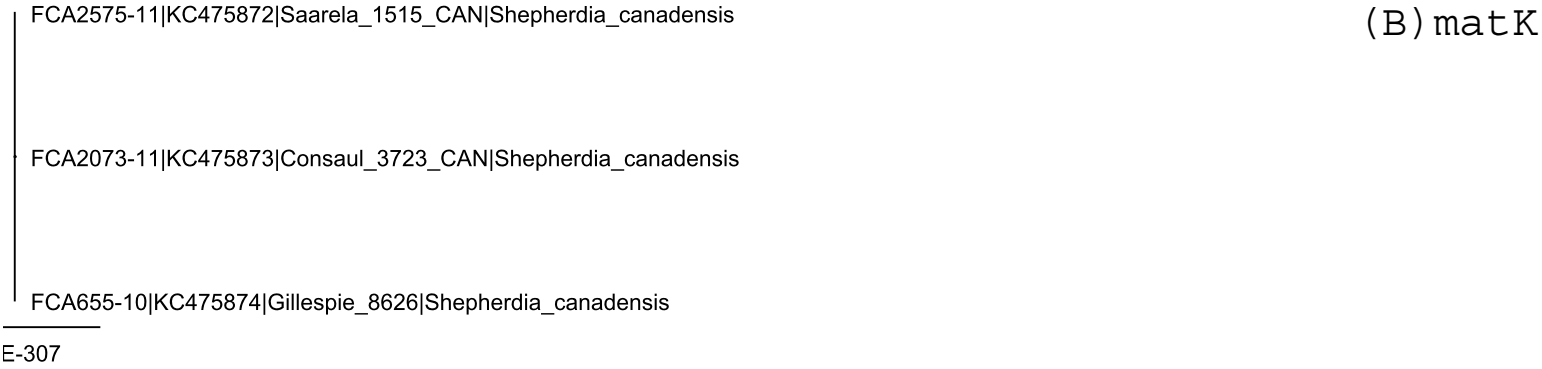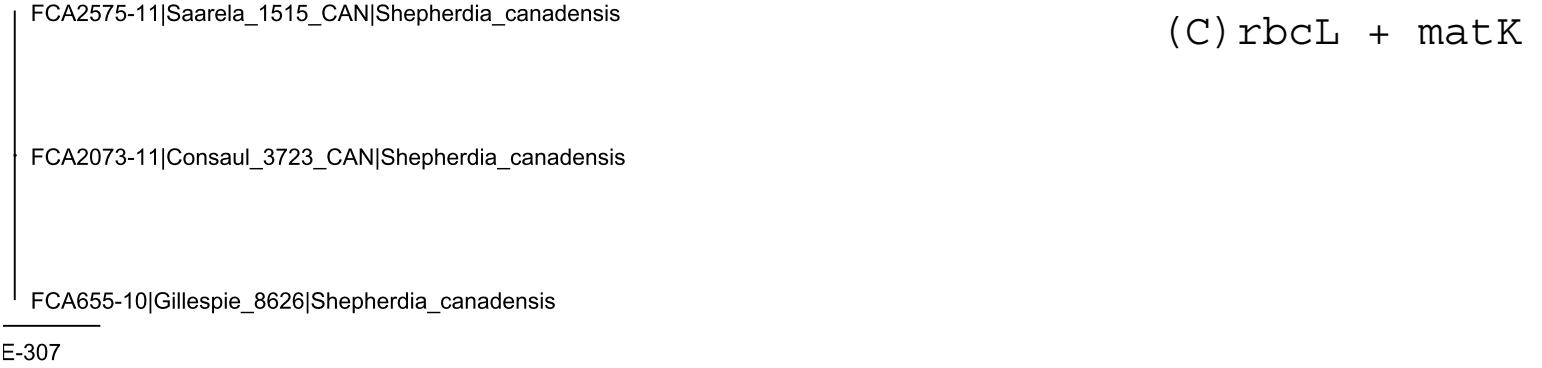

Supplement: Figure S16 — Neighbour joining analyses of uncorrected p-distances of rbcL and matK sequence data for Elaeagnaceae. A. rbcL. B. matK. C. rbcL + matK. (PDF) [file pone.0077982.s021.pdf]

Equisetaceae

rbcL

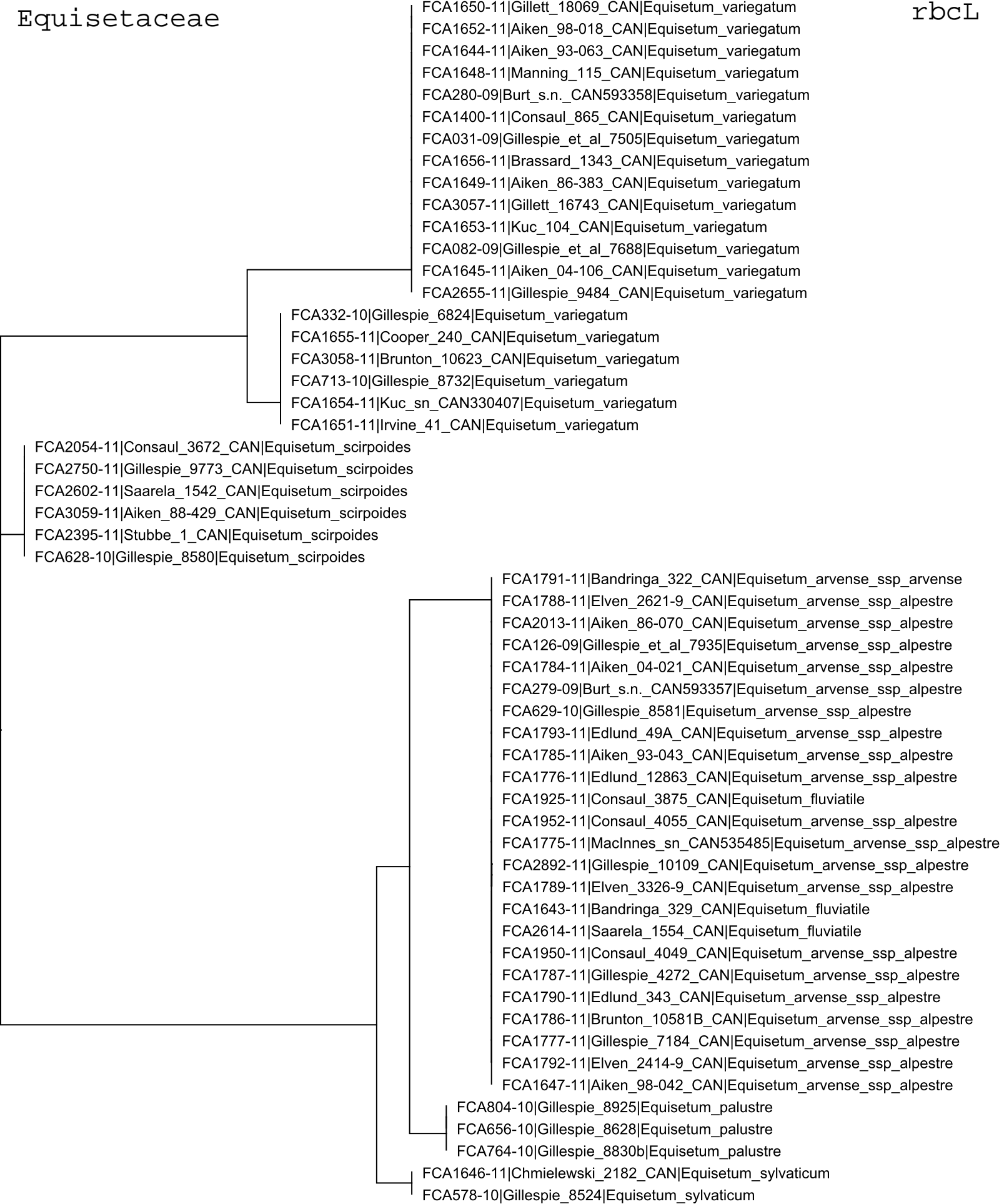

0.0050

Supplement: Figure S17 — Neighbour joining analysis of uncorrected p-distances of rbcL sequence data for Equisetaceae. (PDF) [file pone.0077982.s022.pdf]

Ericaceae

(A) rbcL

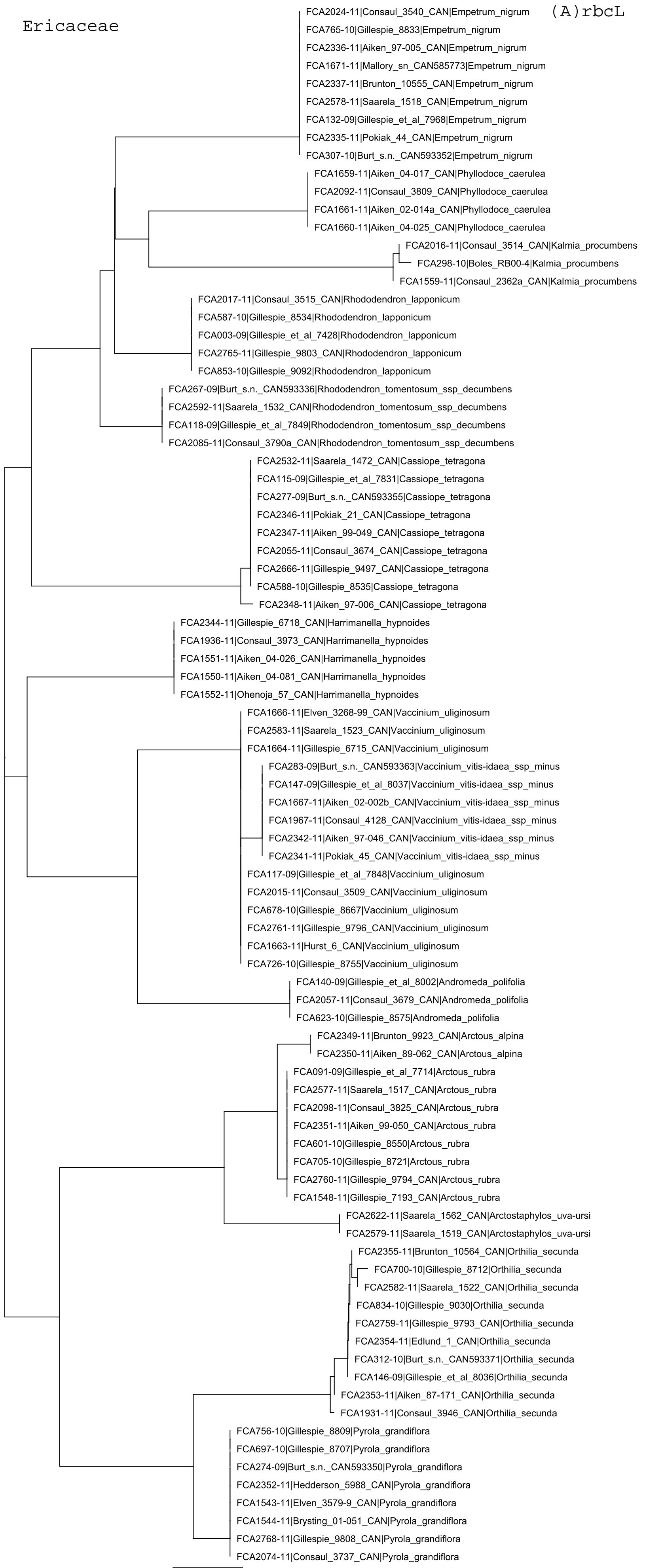

0.0060

Ericaceae (B)matK

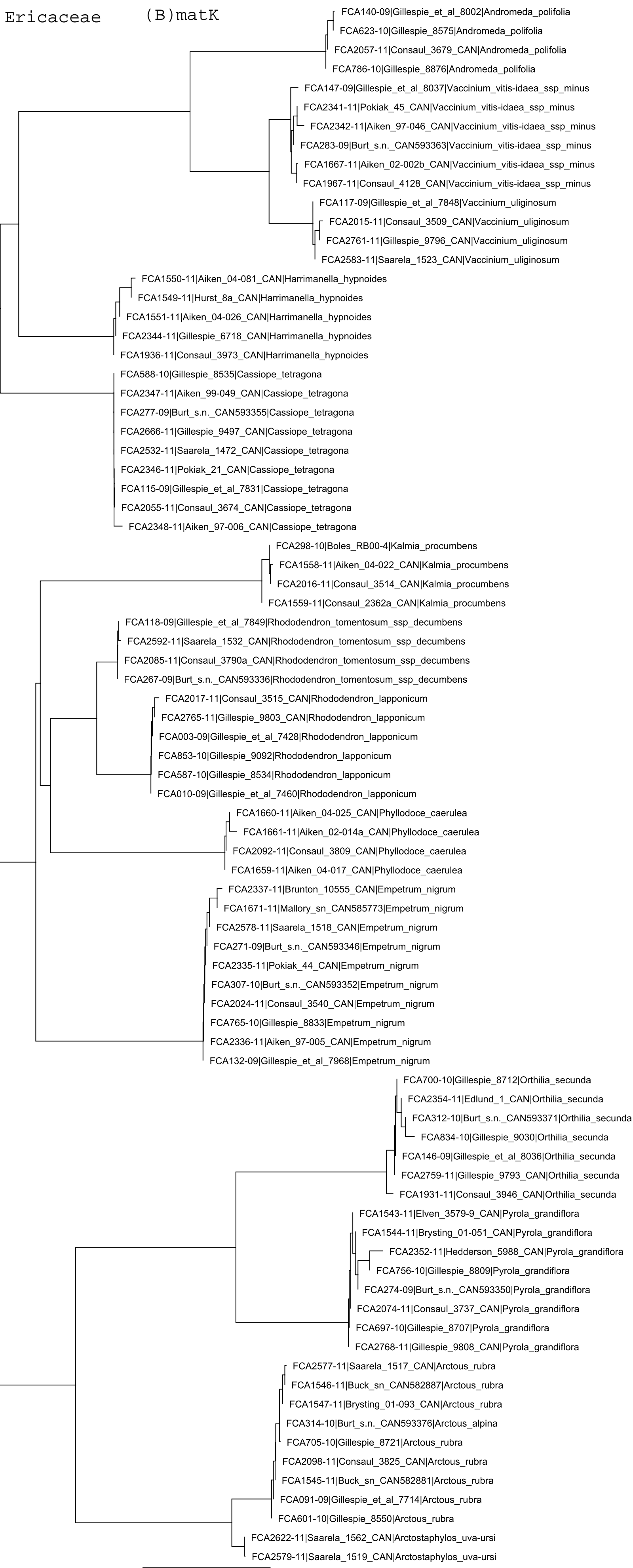

0.02

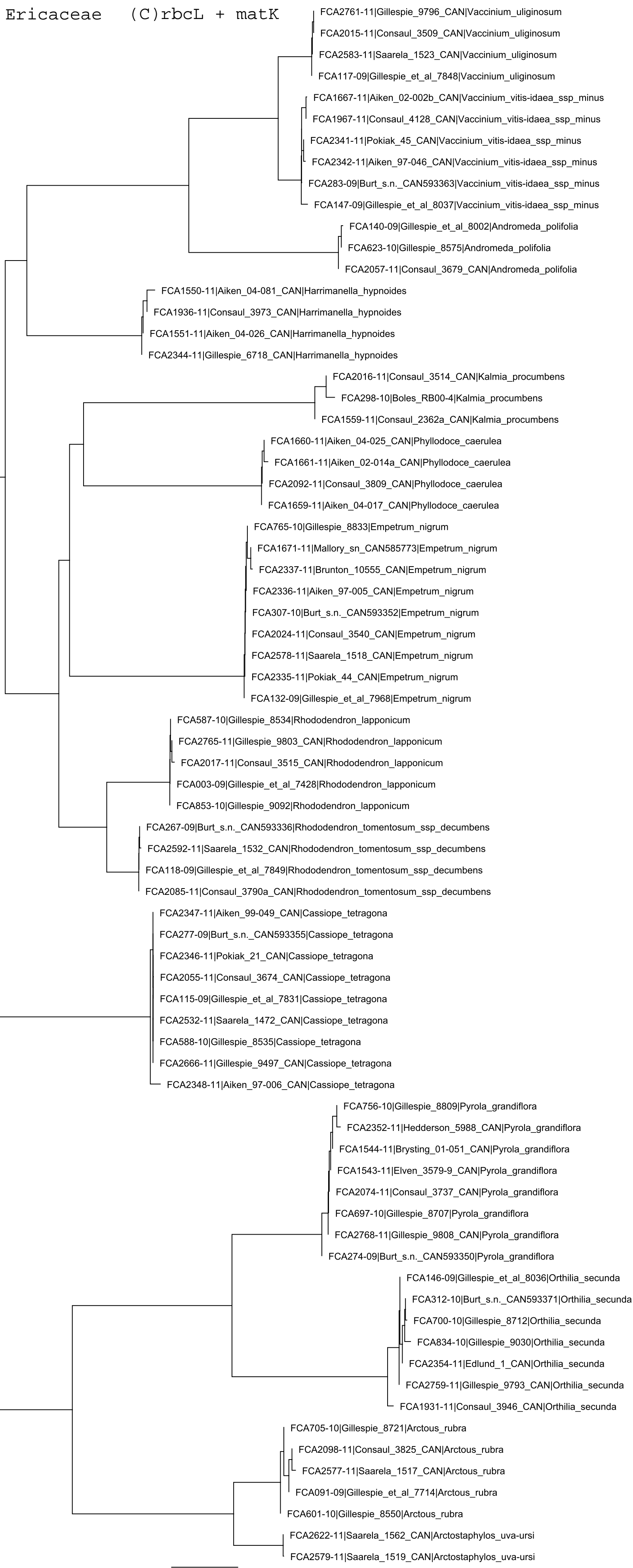

Supplement: Figure S18 — Neighbour joining analyses of uncorrected p-distances of rbcL and matK sequence data for Ericaceae. A. rbcL. B. matK. C. rbcL + matK. (PDF) [file pone.0077982.s023.pdf]

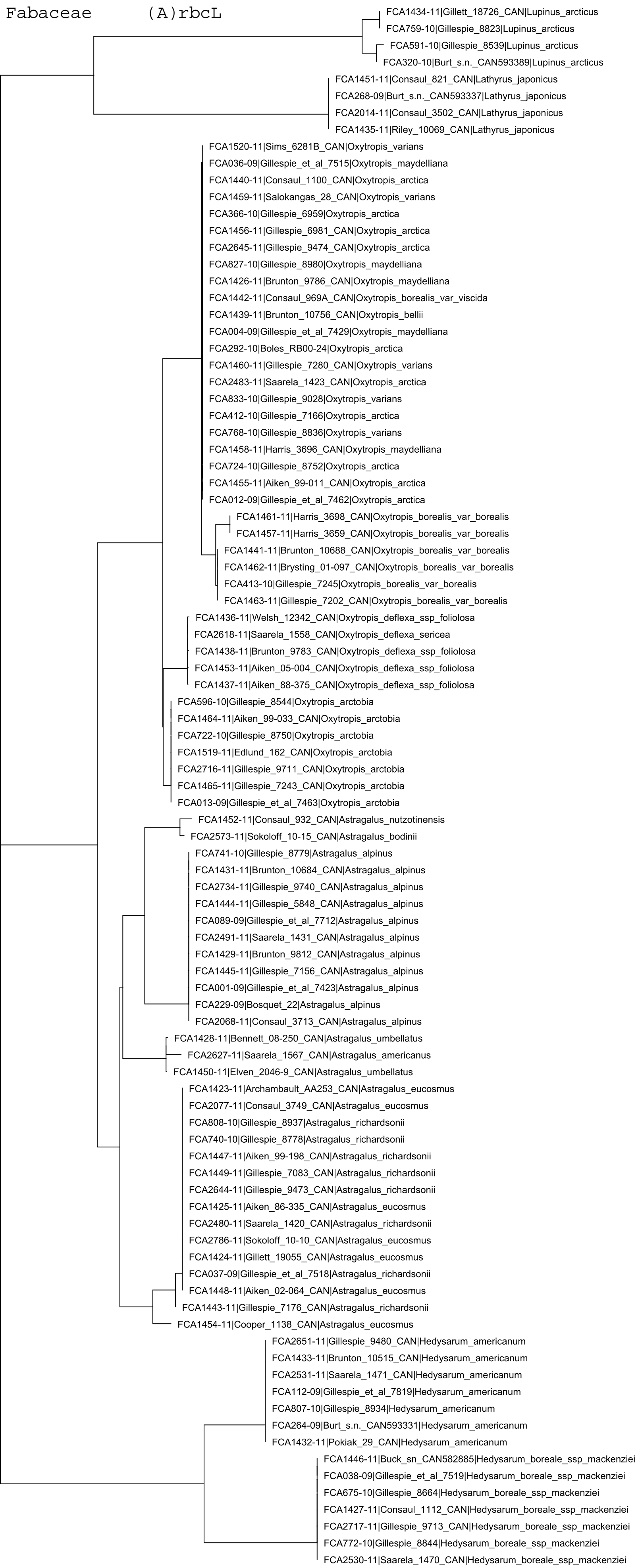

(B)matK

Fabaceae

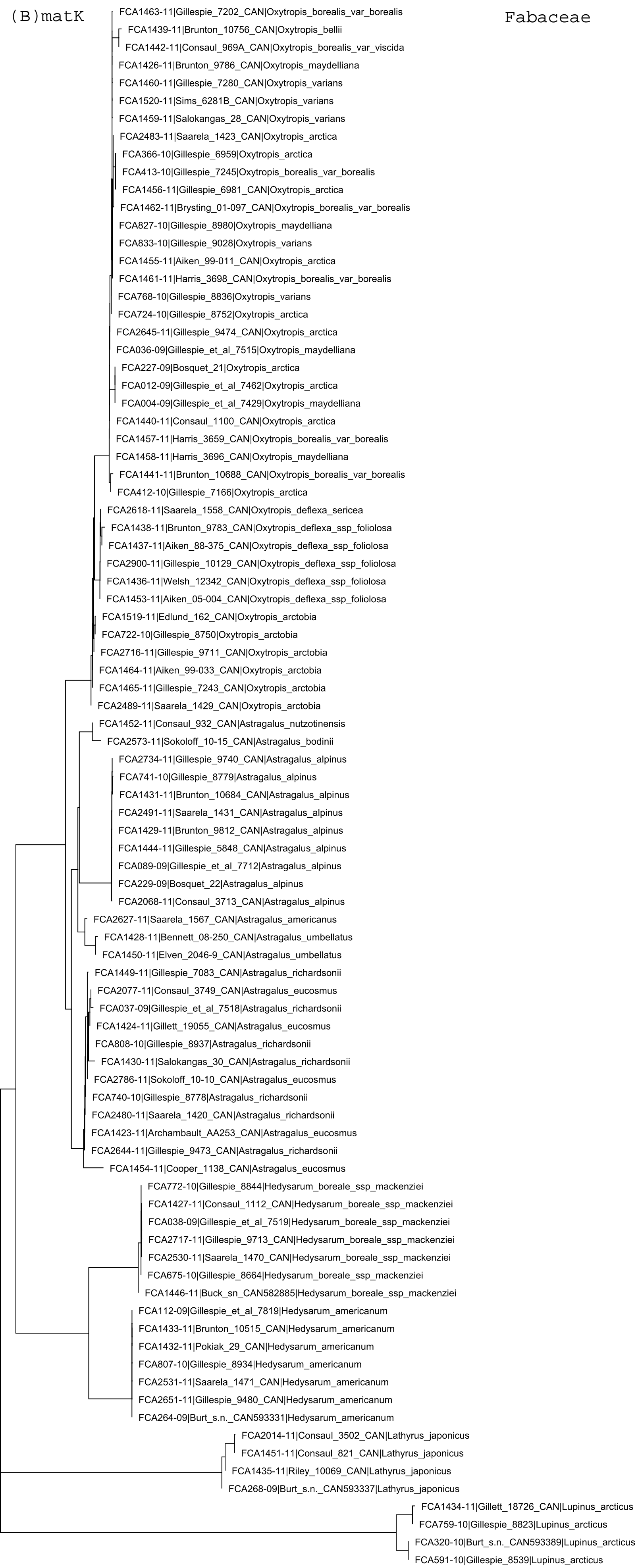

0.02

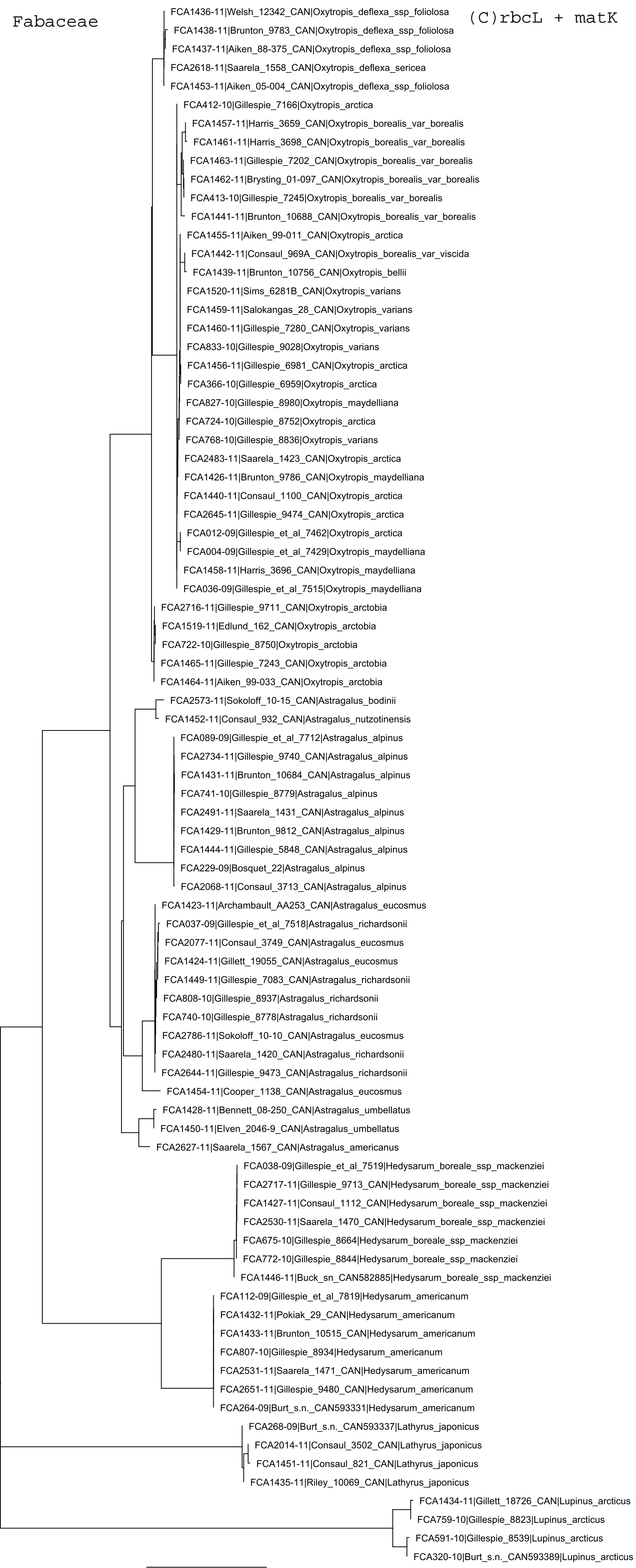

Supplement: Figure S19 — Neighbour joining analyses of uncorrected p-distances of rbcL and matK sequence data for Fabaceae. A. rbcL. B. matK. C. rbcL + matK. (PDF) [file pone.0077982.s024.pdf]

Gentianeaceae

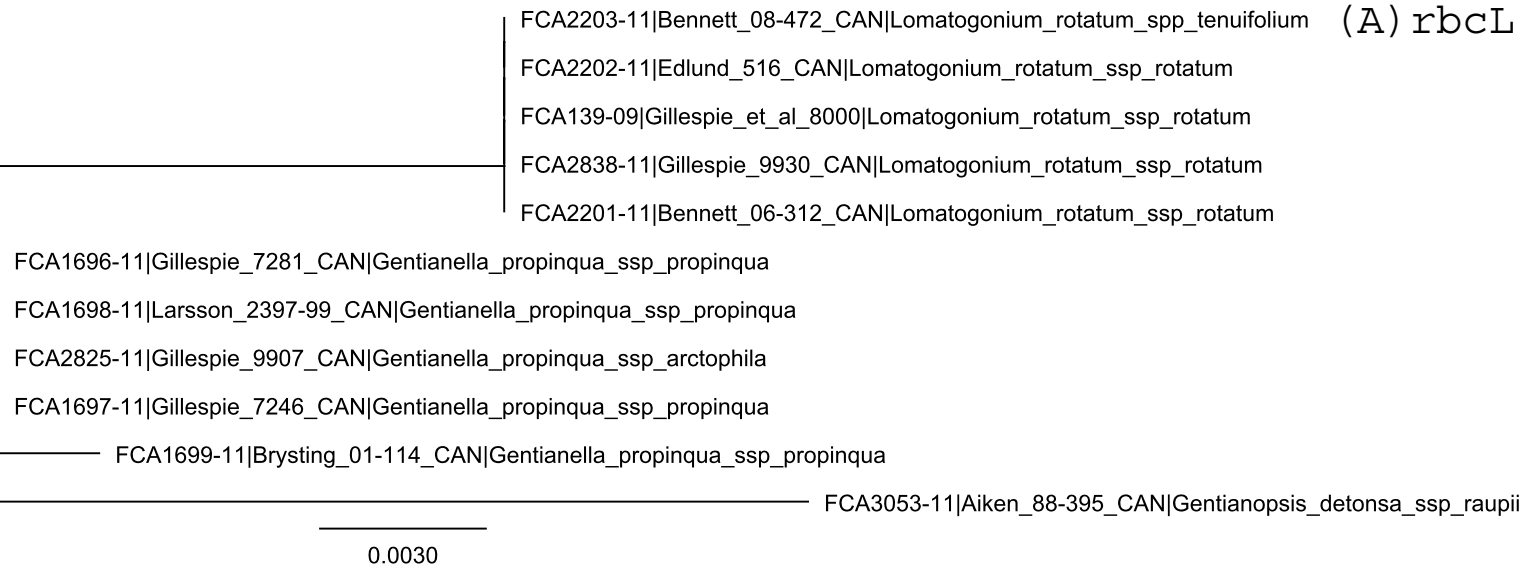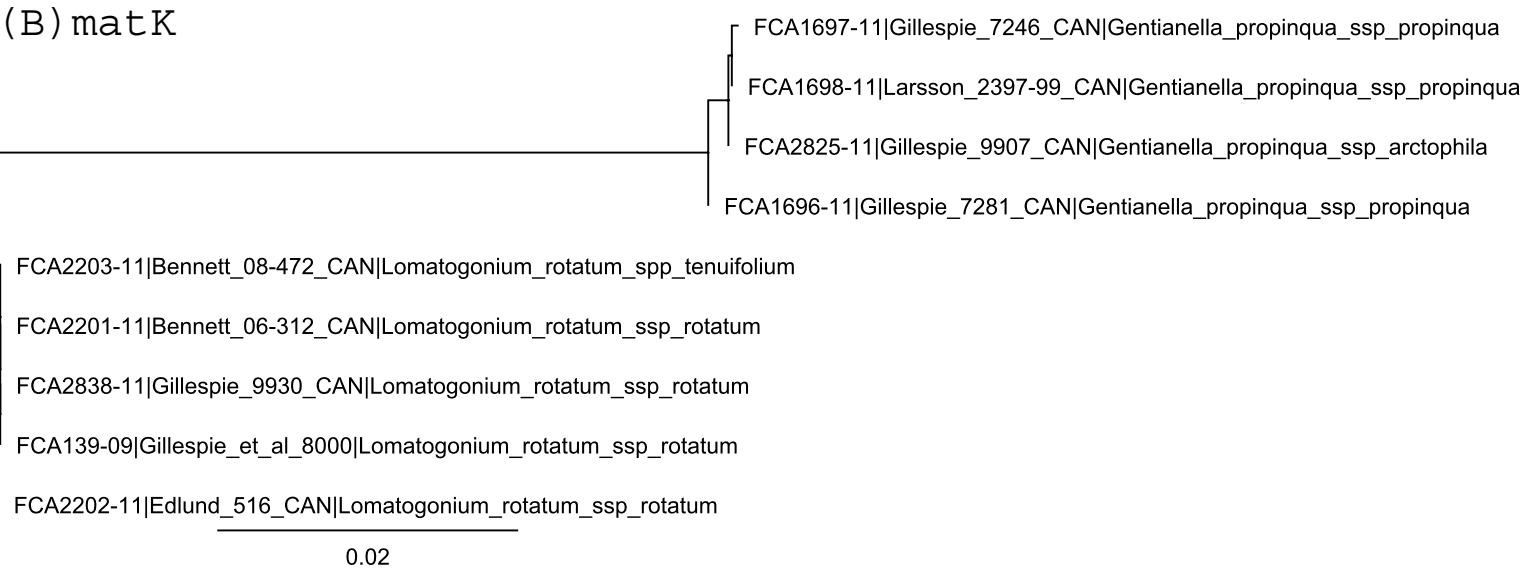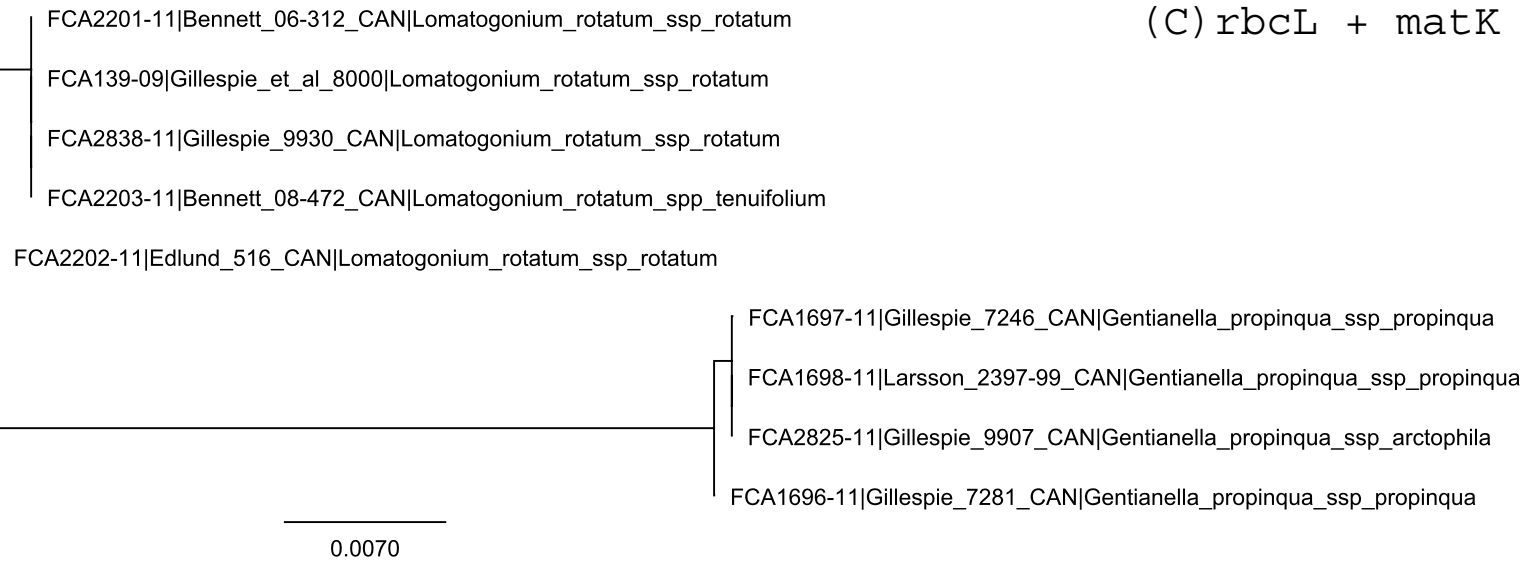

Supplement: Figure S20 — Neighbour joining analyses of uncorrected p-distances of rbcL and matK sequence data for Gentianaceae. A. rbcL. B. matK. C. rbcL + matK. (PDF) [file pone.0077982.s025.pdf]

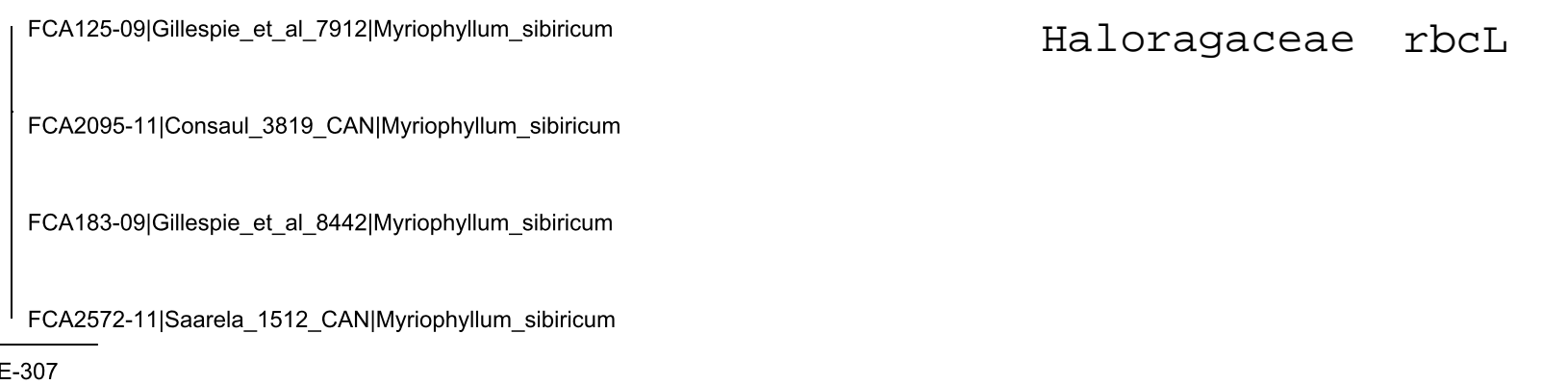

Supplement: Figure S21 — Neighbour joining analysis of uncorrected p-distances of rbcL sequence data for Haloragaceae. (PDF) [file pone.0077982.s026.pdf]

Juncaceae

rbcl

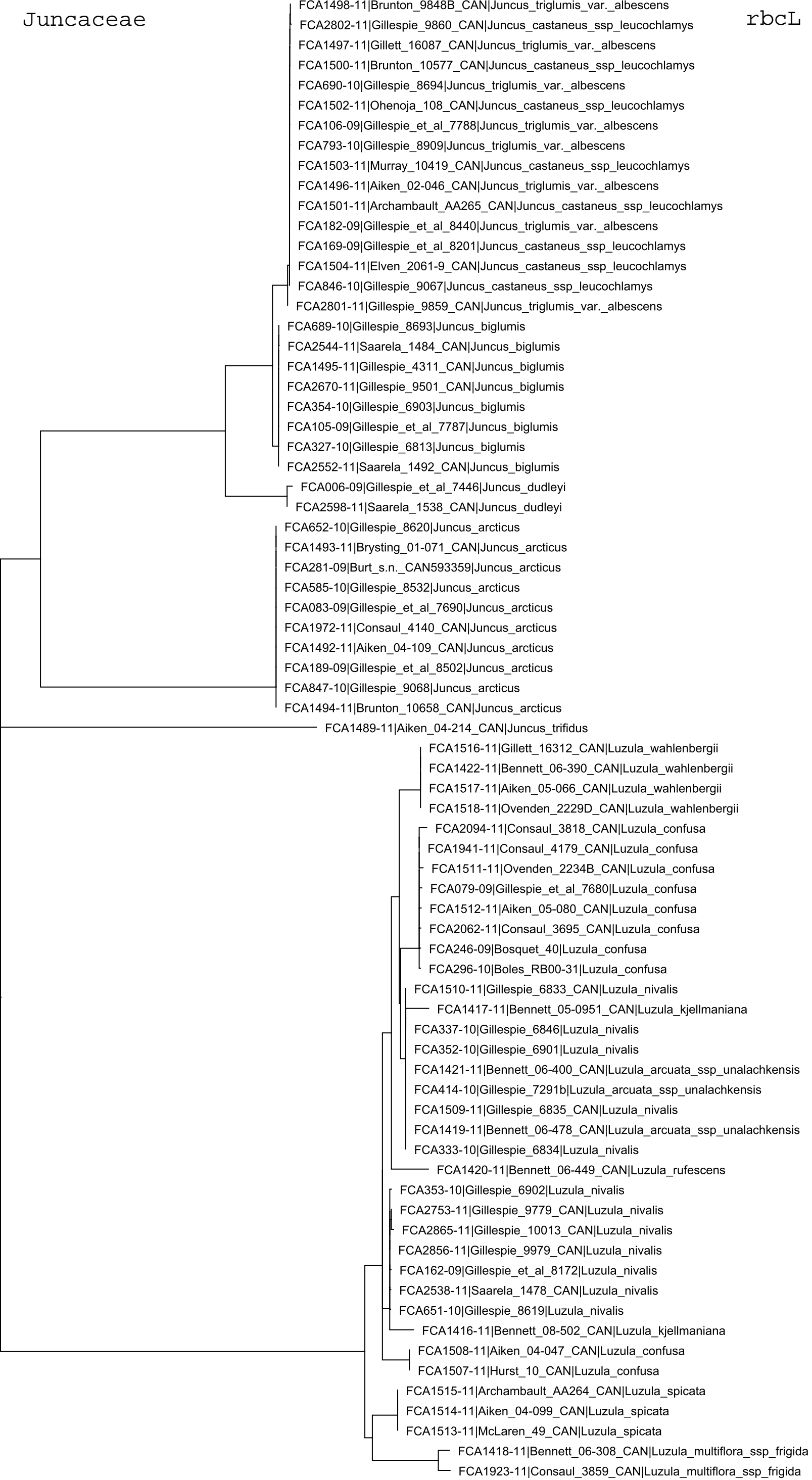

0.0070

Supplement: Figure S22 — Neighbour joining analysis of uncorrected p-distances of rbcL sequence data for Juncaceae. (PDF) [file pone.0077982.s027.pdf]

Juncaginaceae

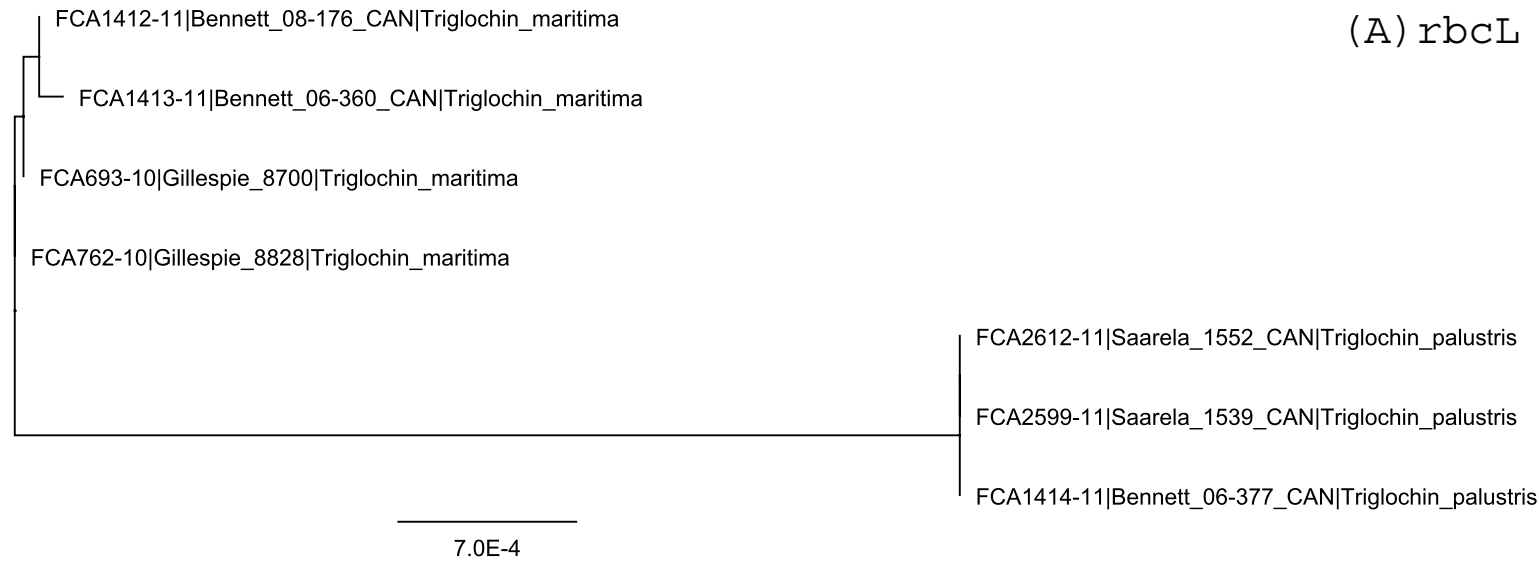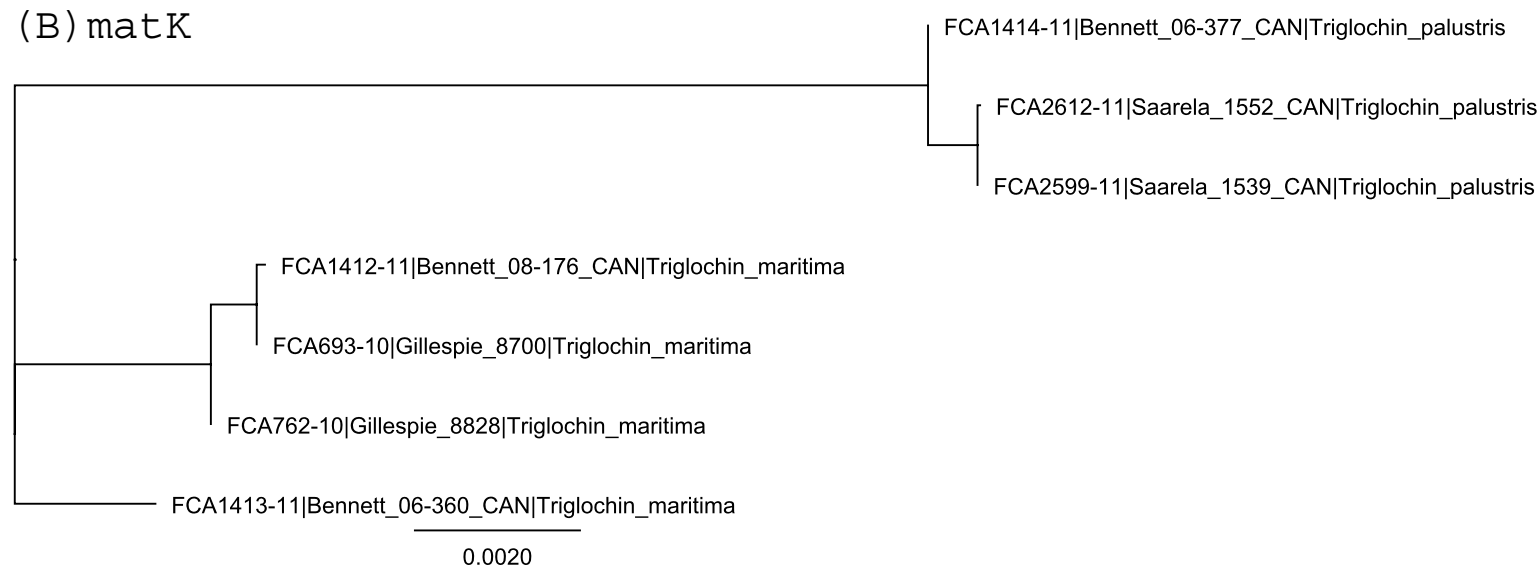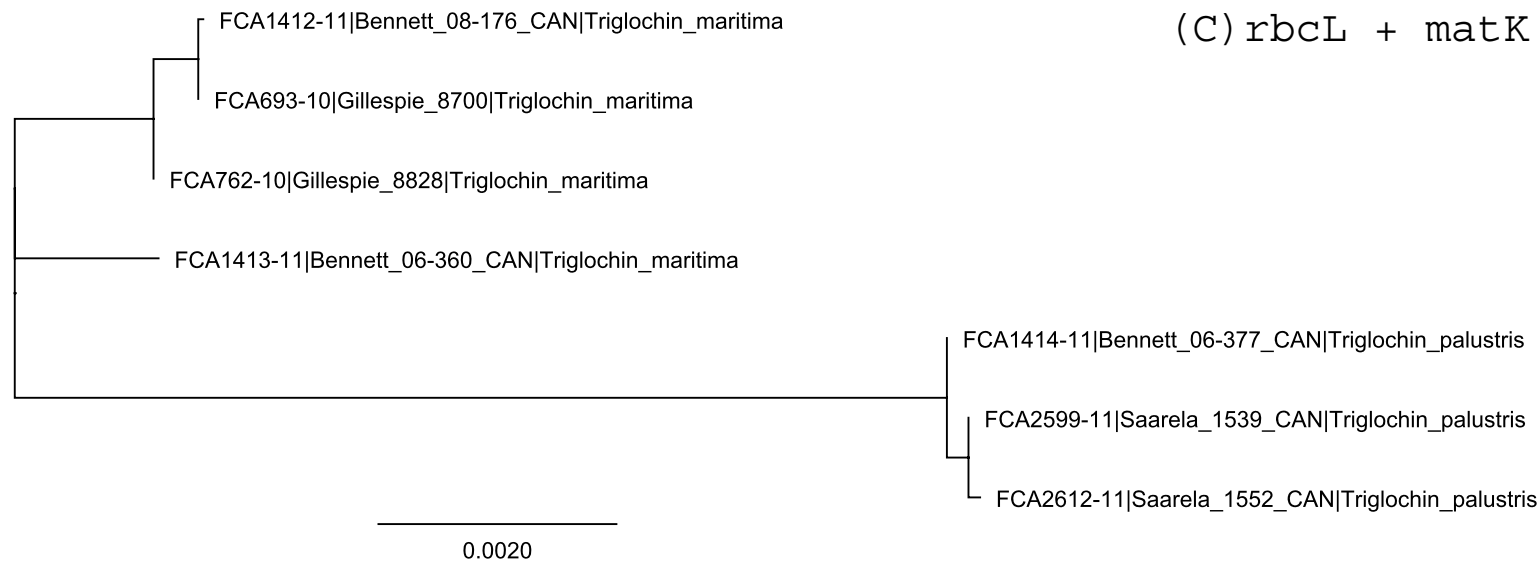

Supplement: Figure S23 — Neighbour joining analyses of uncorrected p-distances of rbcL and matK sequence data for Juncaginaceae. A. rbcL. B. matK. C. rbcL + matK. (PDF) [file pone.0077982.s028.pdf]

# Lentibulariaceae

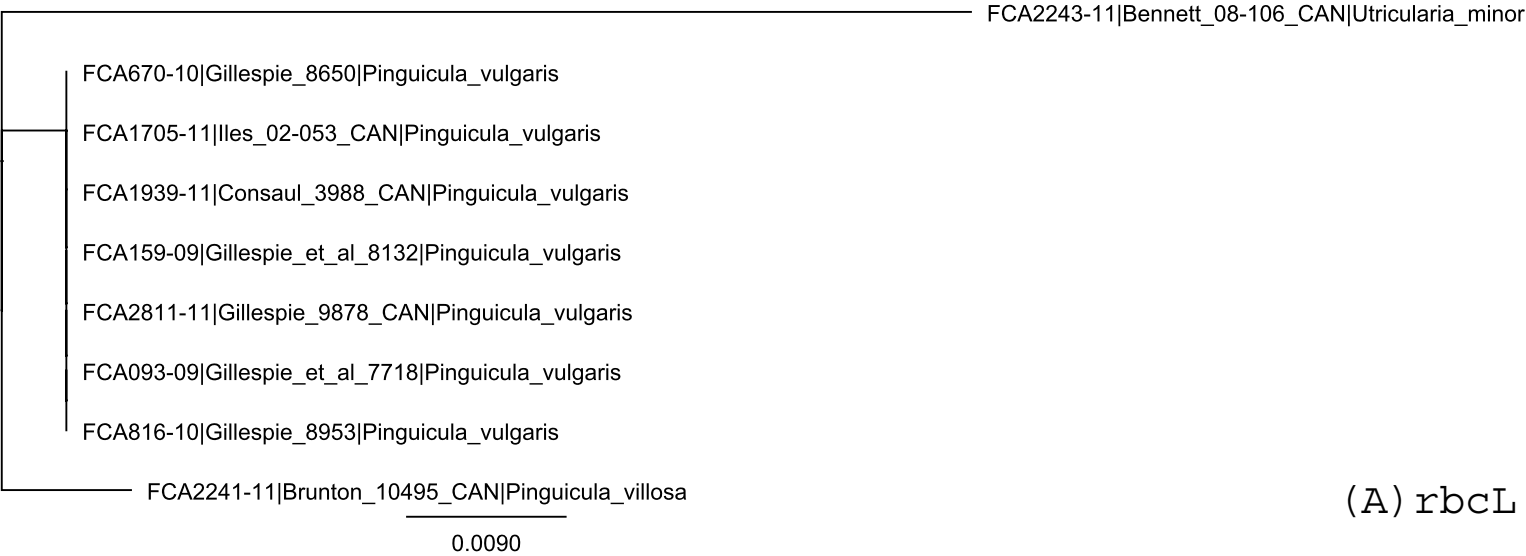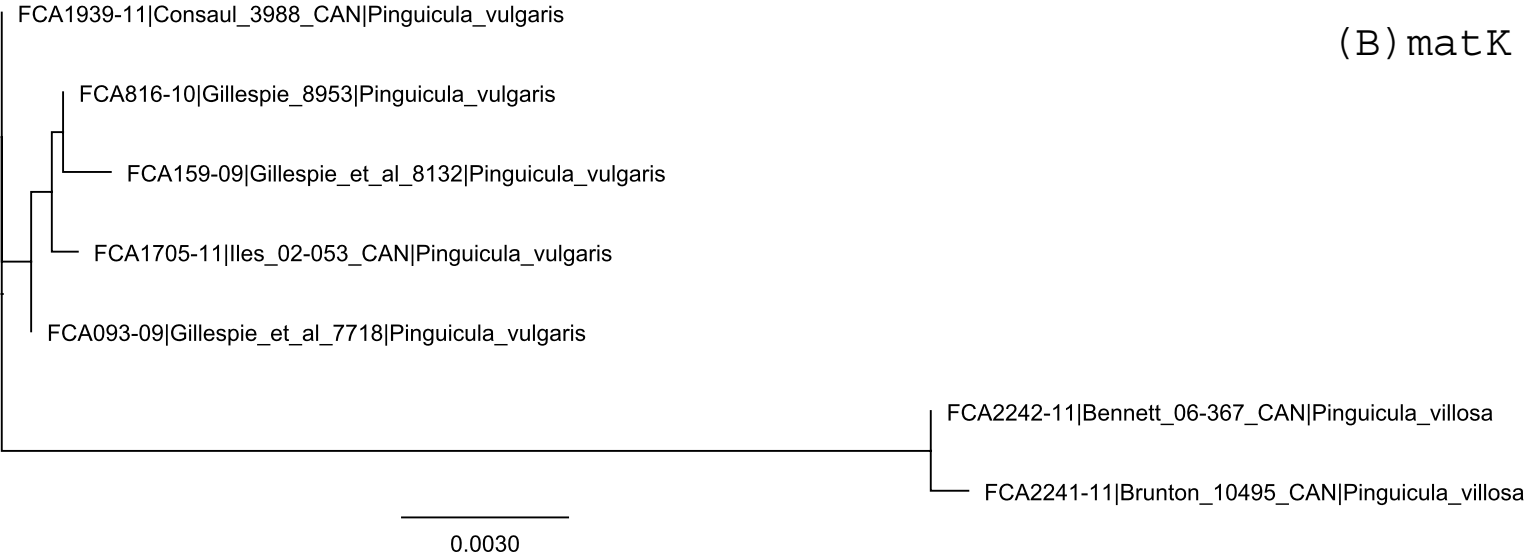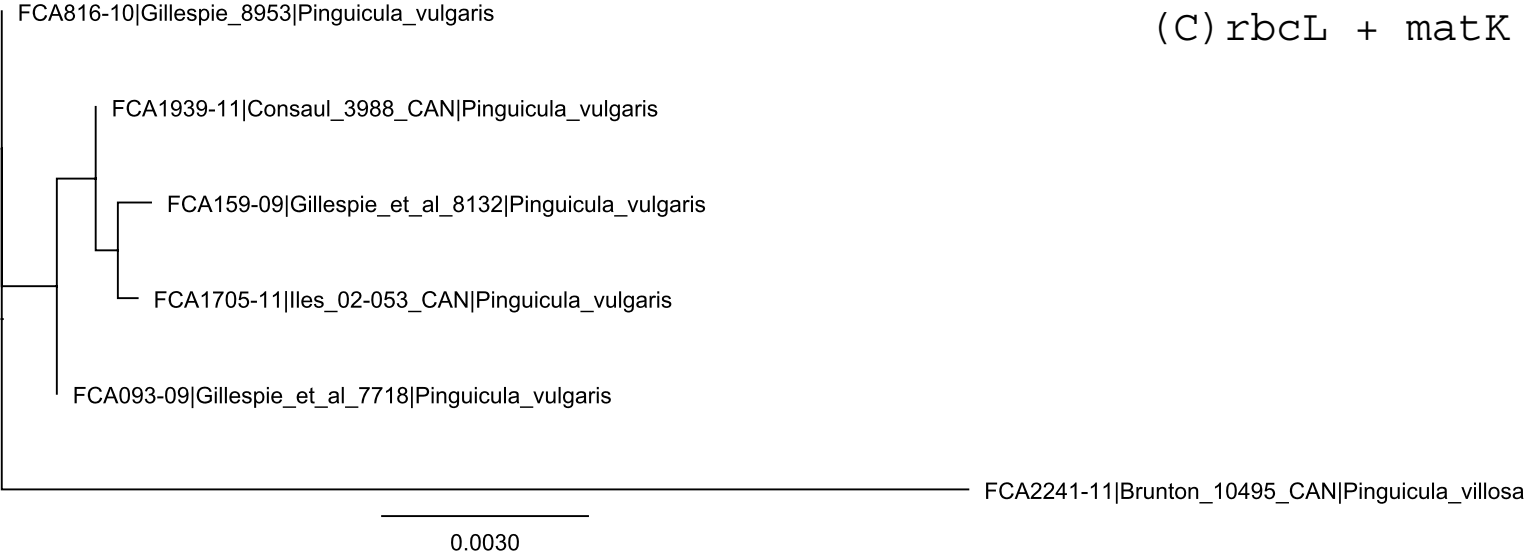

Supplement: Figure S24 — Neighbour joining analyses of uncorrected p-distances of rbcL and matK sequence data for Lentibulariaceae. A. rbcL. B. matK. C. rbcL + matK. (PDF) [file pone.0077982.s029.pdf]

Melanthiaceae

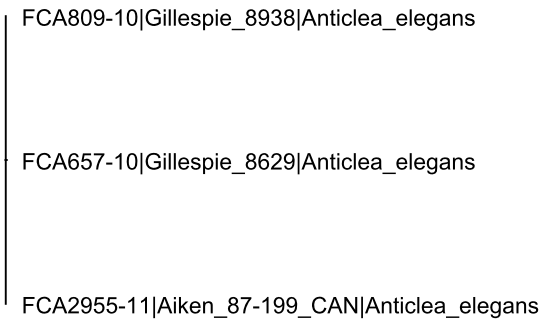

(A) rbcL

E-307

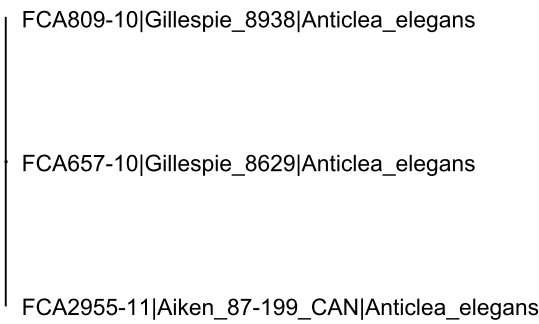

(B) matK

E-307

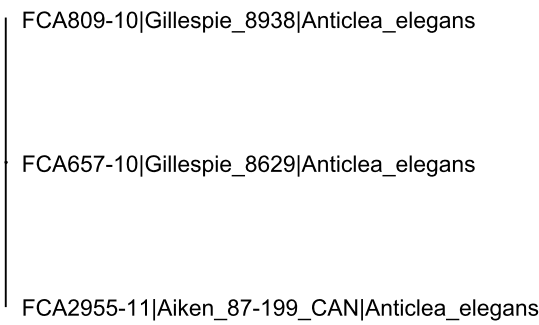

(C) rbcL + matK

E-307

Supplement: Figure S27 — Neighbour joining analyses of uncorrected p-distances of rbcL and matK sequence data for Melanthiaceae. A. rbcL. B. matK. C. rbcL + matK. (PDF) [file pone.0077982.s032.pdf]

# Menyanthaceae

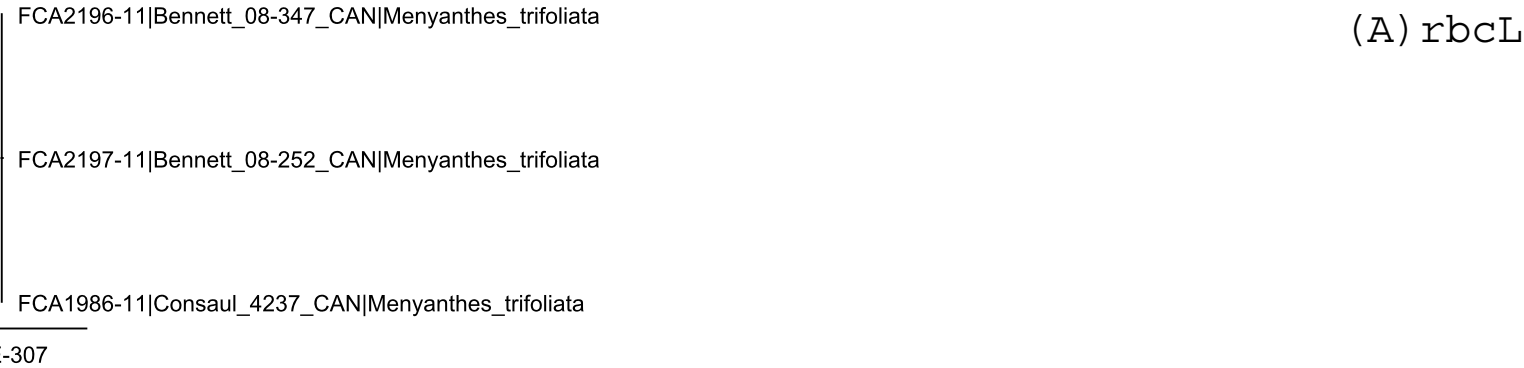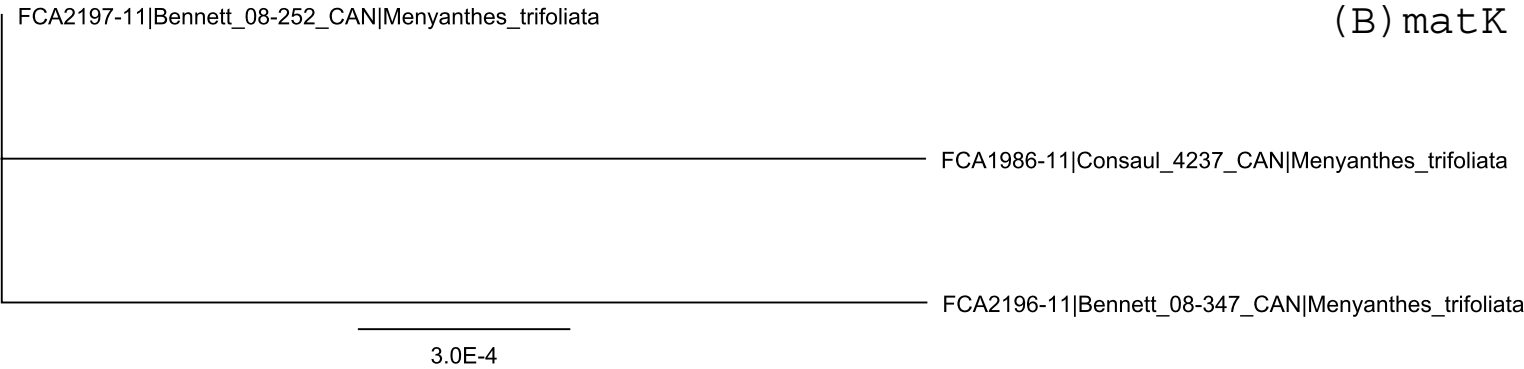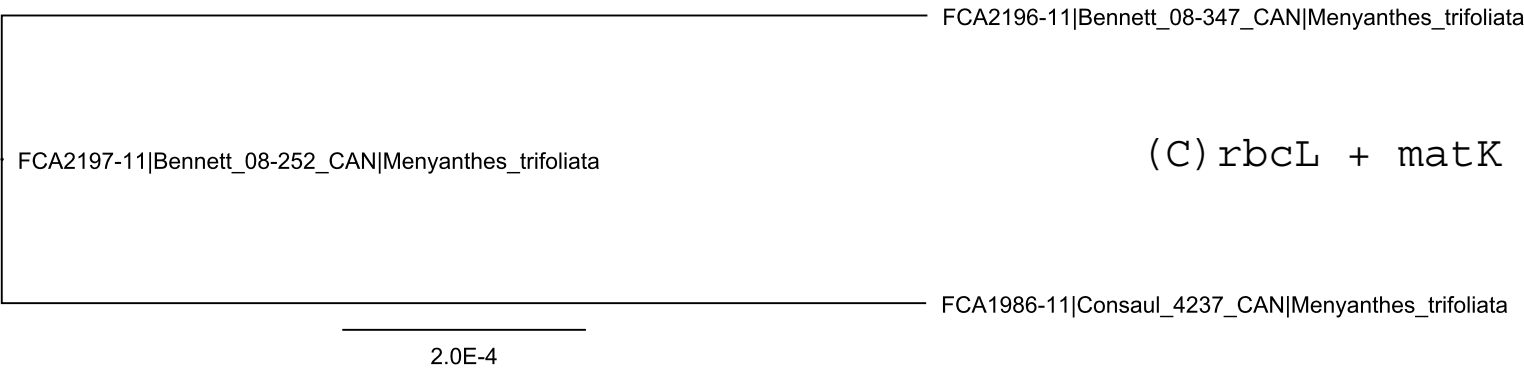

Supplement: Figure S28 — Neighbour joining analyses of uncorrected p-distances of rbcL and matK sequence data for Menyanthaceae. A. rbcL. B. matK. C. rbcL + matK. (PDF) [file pone.0077982.s033.pdf]

Montiaceae

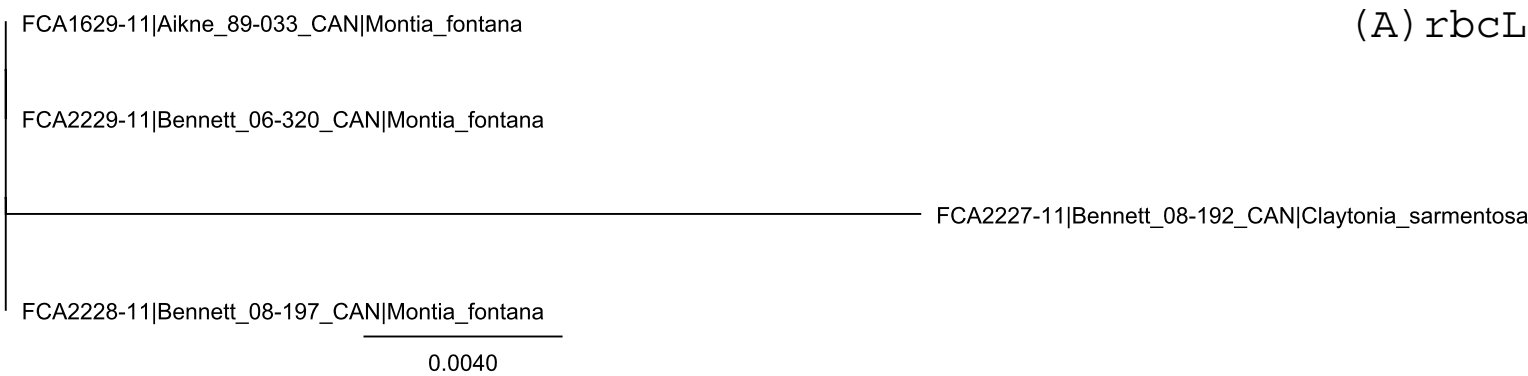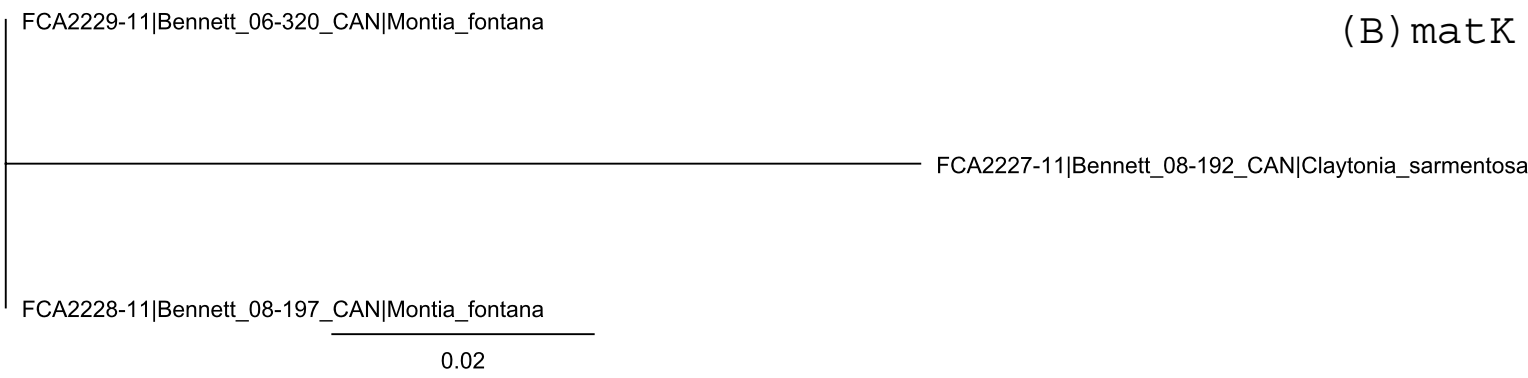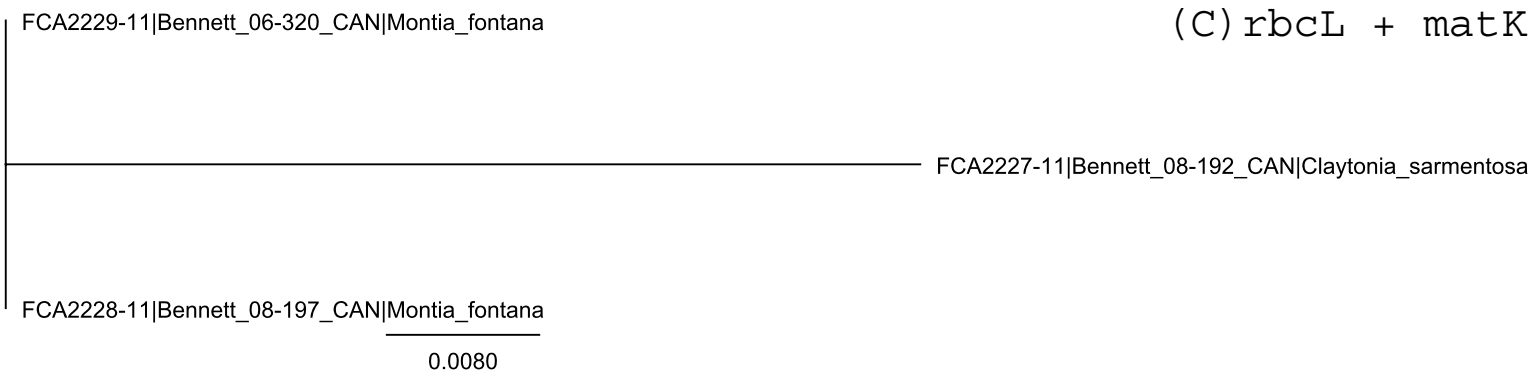

Supplement: Figure S29 — Neighbour joining analyses of uncorrected p-distances of rbcL and matK sequence data for Montiaceae. A. rbcL. B. matK. C. rbcL + matK. (PDF) [file pone.0077982.s034.pdf]

Onagraceae

( A ) rbcL

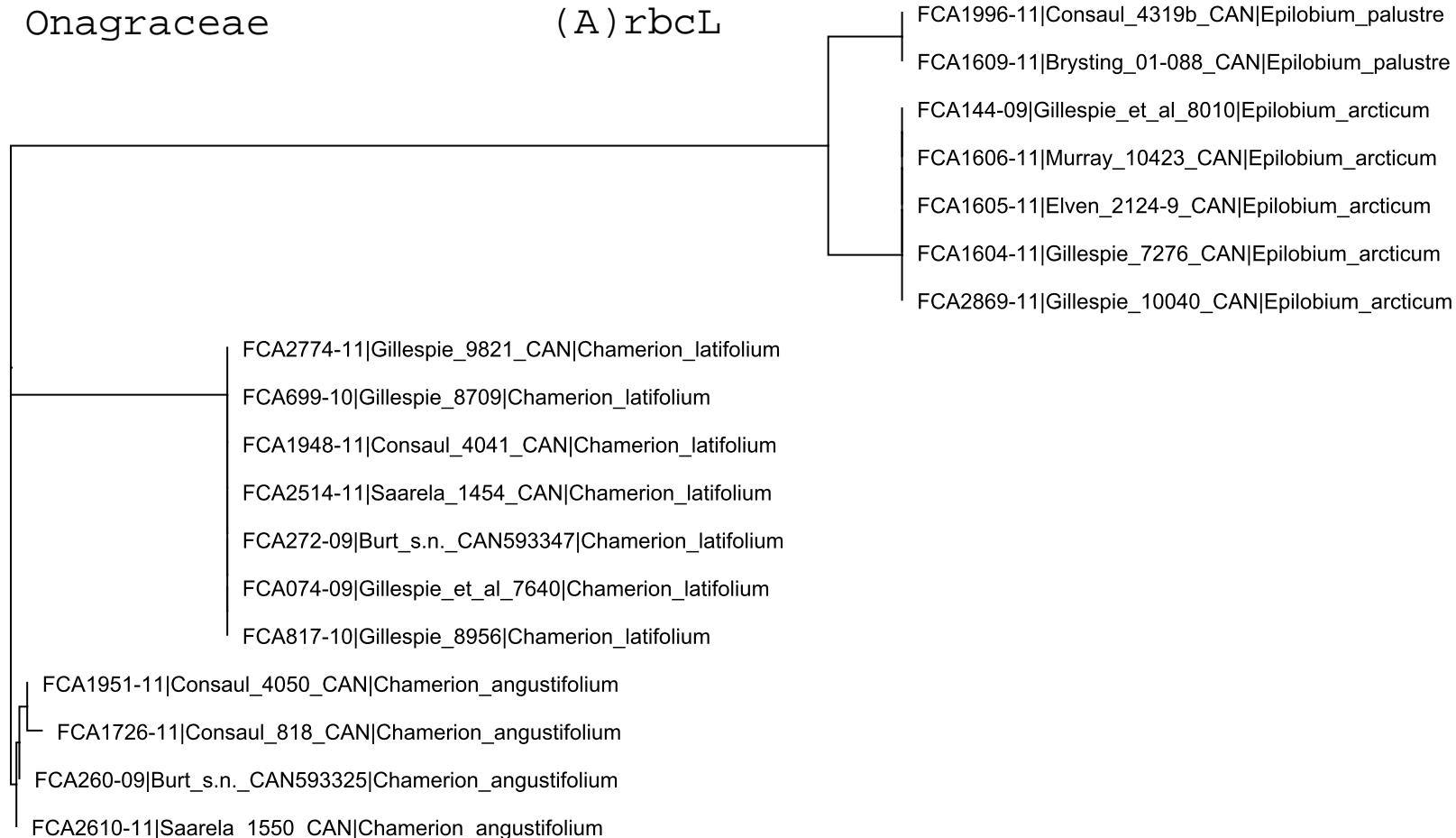

0.0040

Onagraceae

( B ) matK

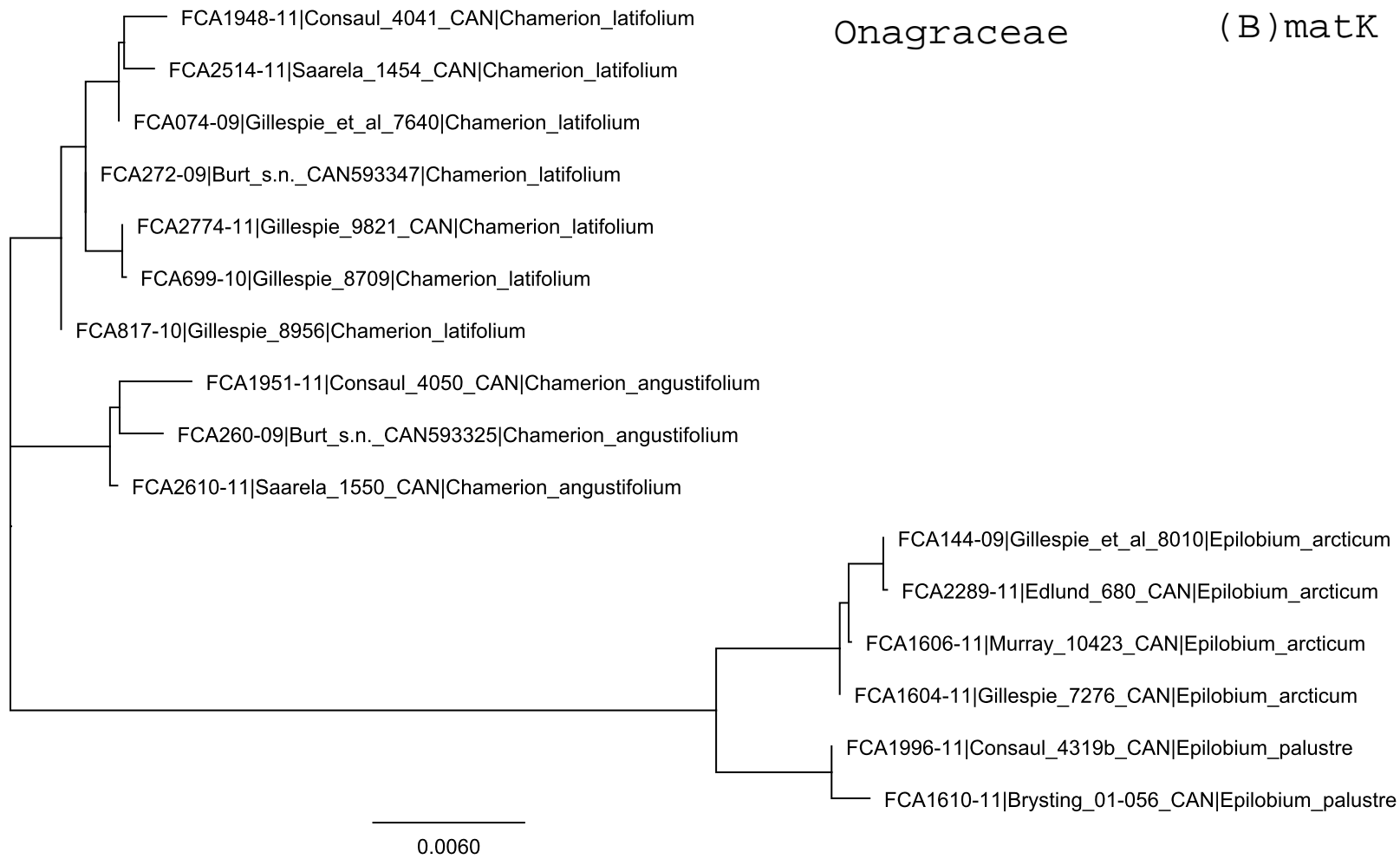

Onagraceae (C)rbcL + matK

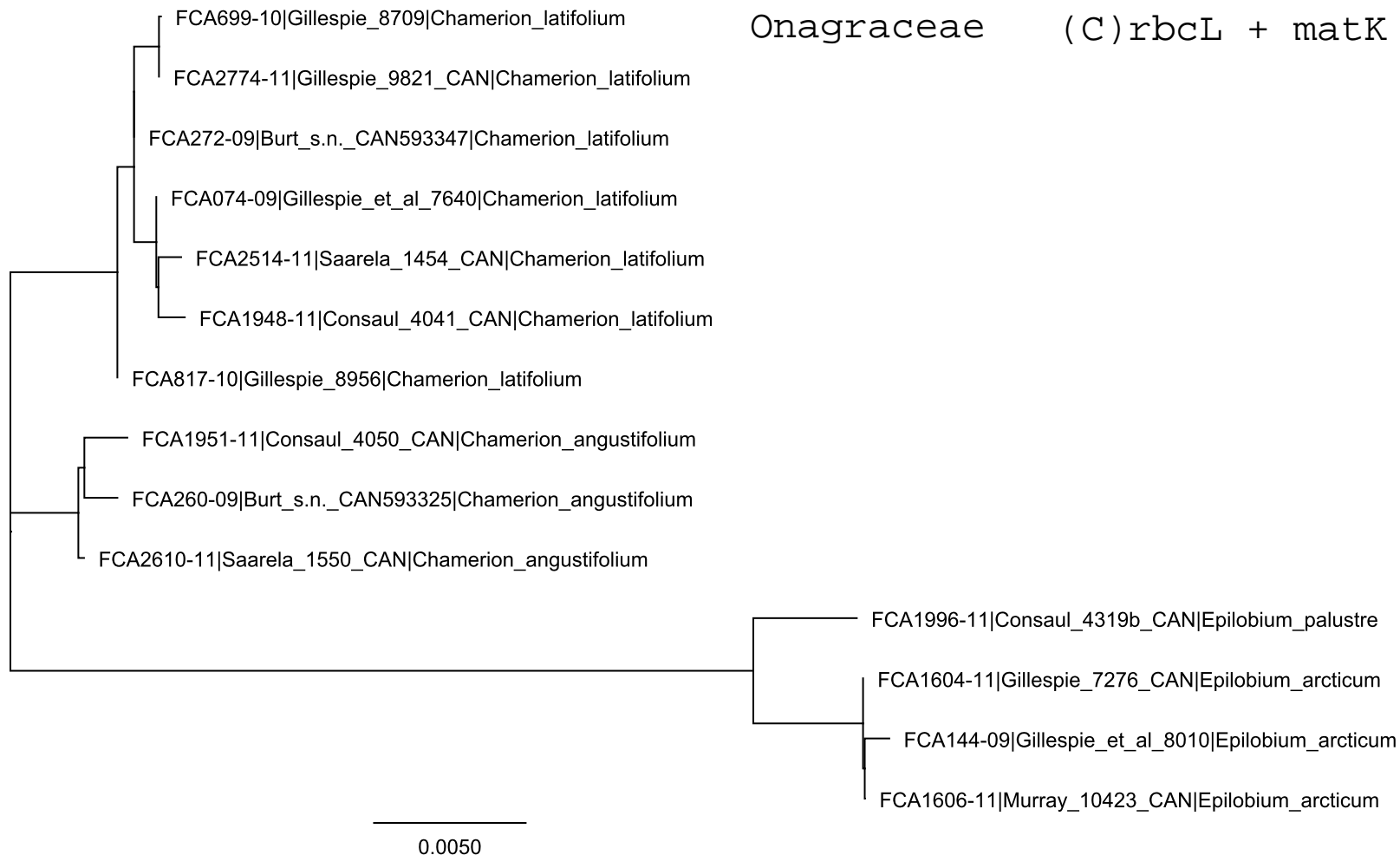

Supplement: Figure S30 — Neighbour joining analyses of uncorrected p-distances of rbcL and matK sequence data for Onagraceae. A. rbcL. B. matK. C. rbcL + matK. (PDF) [file pone.0077982.s035.pdf]

Orchidaceae

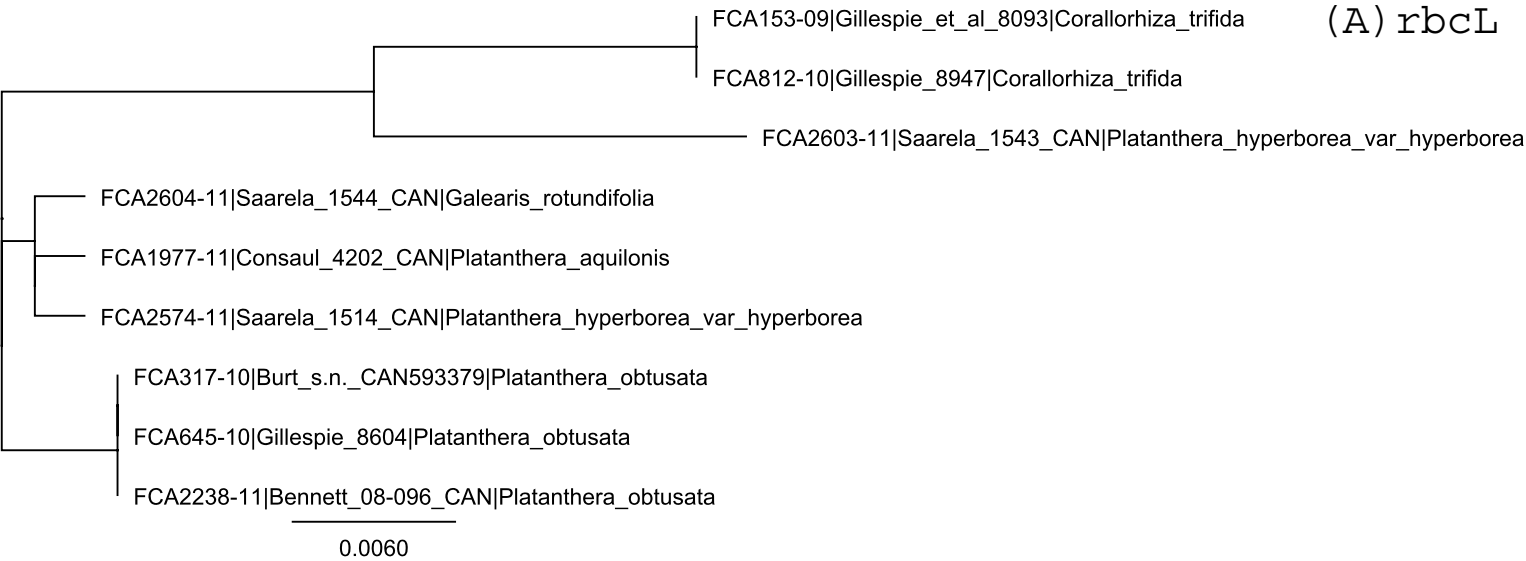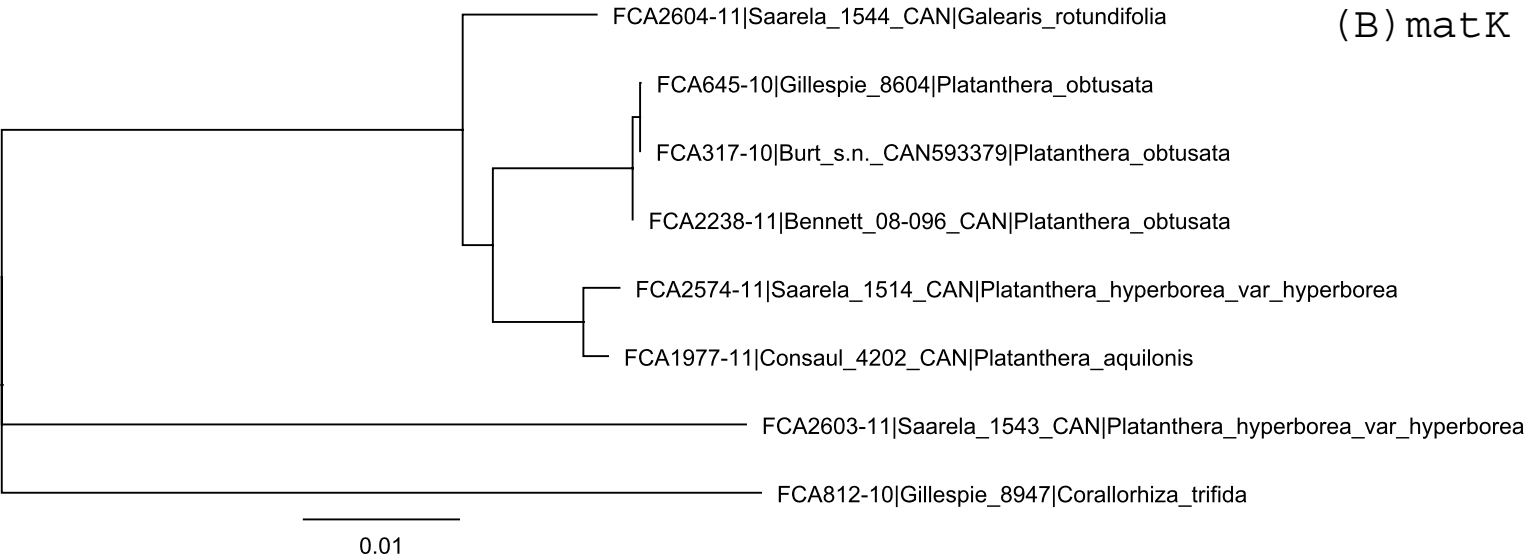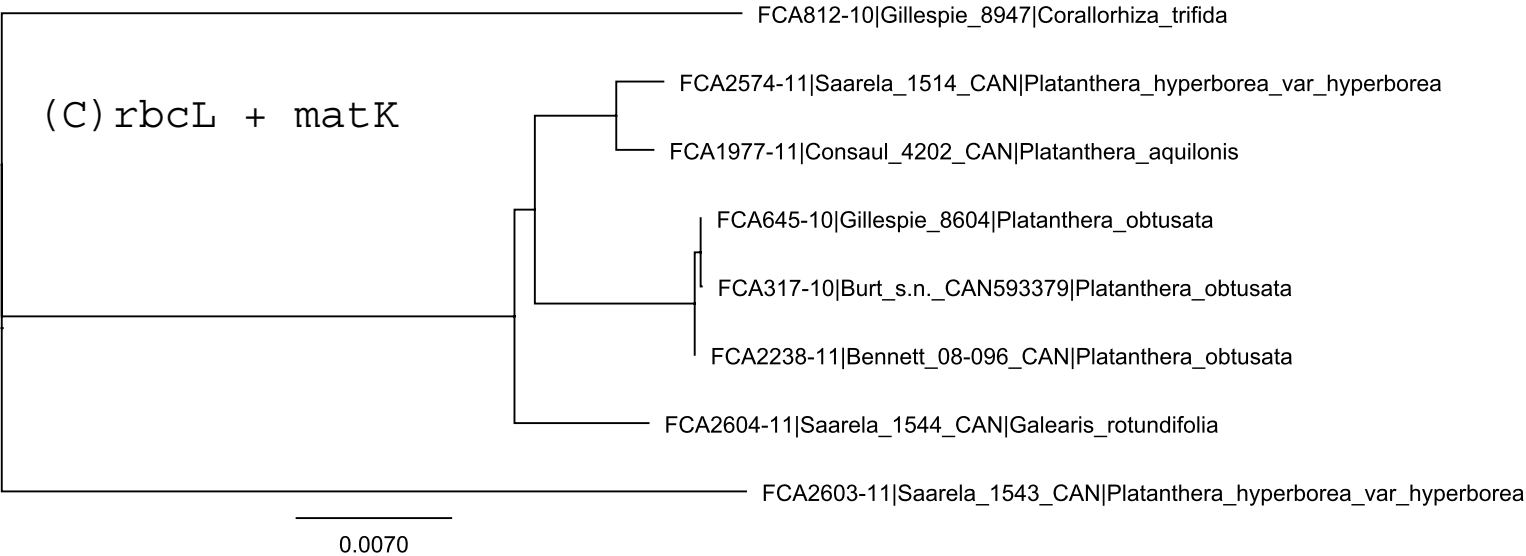

Supplement: Figure S31 — Neighbour joining analyses of uncorrected p-distances of rbcL and matK sequence data for Orchidaceae. A. rbcL. B. matK. C. rbcL + matK. (PDF) [file pone.0077982.s036.pdf]

## Orobanchaceae

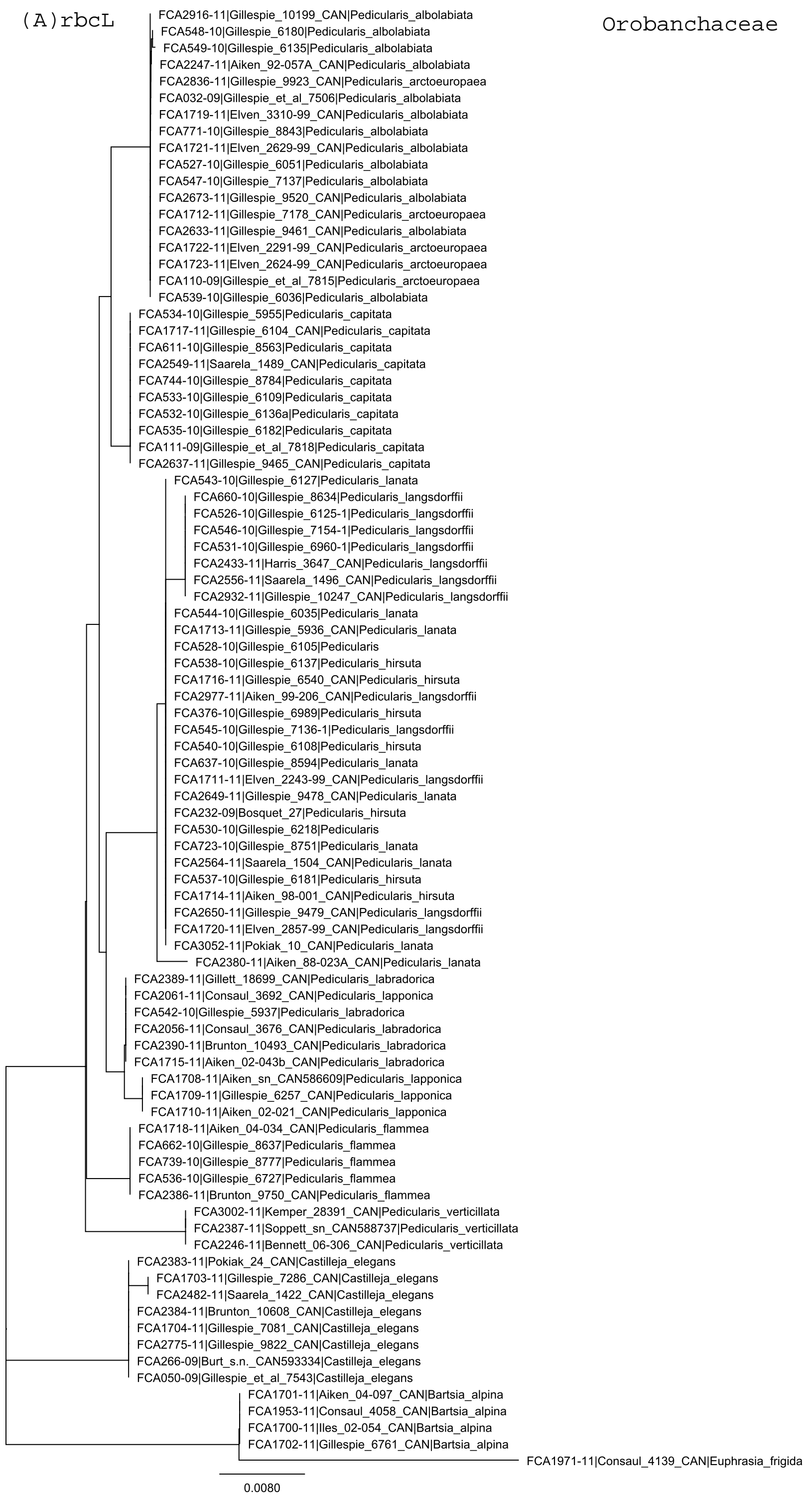

( B ) matK

Orobanchaceae

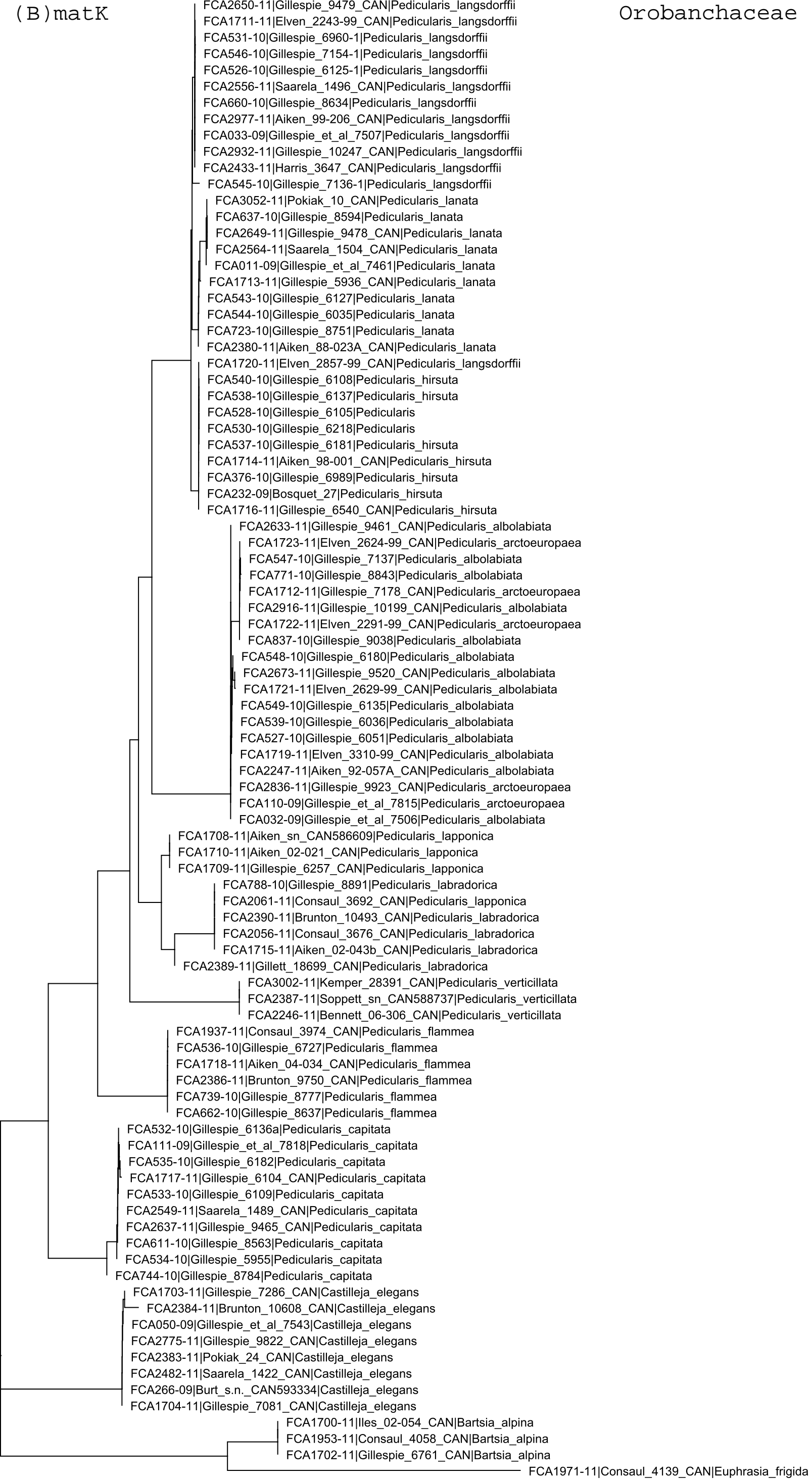

0.02

(C)rbcl + matK

Orobanchaceae

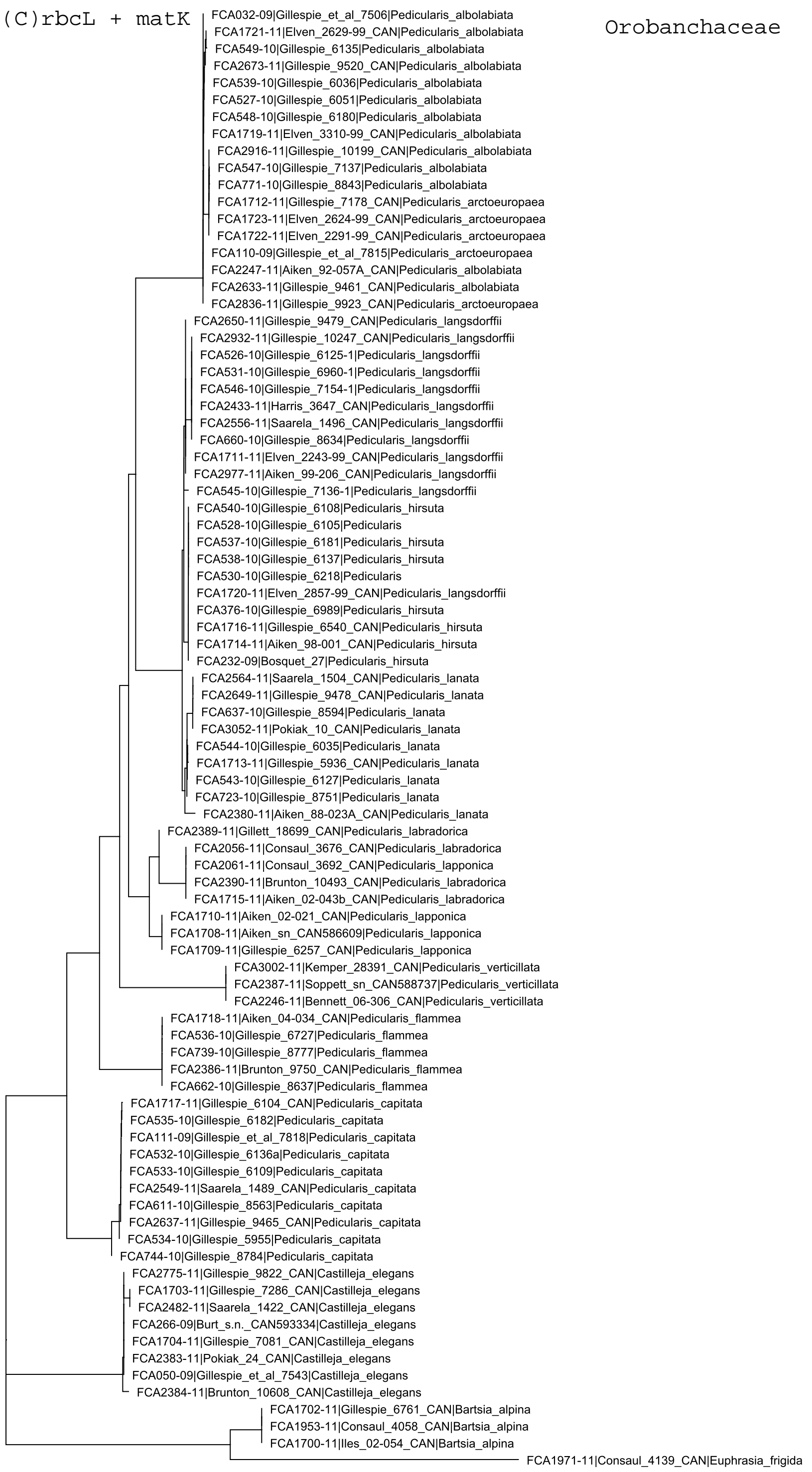

0.02

Supplement: Figure S32 — Neighbour joining analyses of uncorrected p-distances of rbcL and matK sequence data for Orobanchaceae. A. rbcL. B. matK. C. rbcL + matK. (PDF) [file pone.0077982.s037.pdf]

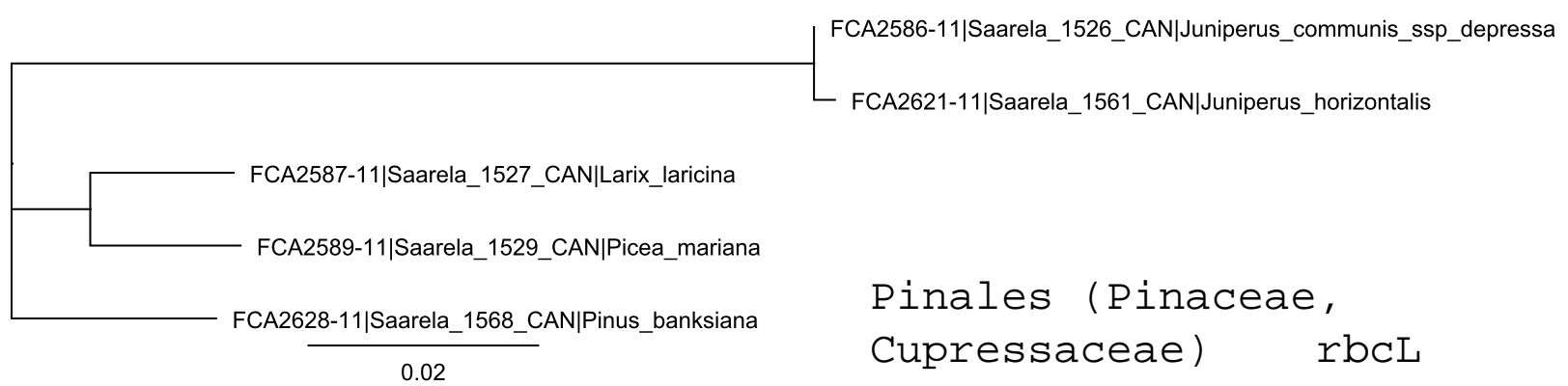

Supplement: Figure S34 — Neighbour joining analysis of uncorrected p-distances of rbcL sequence data for Pinales (Pinaceae, Cupressaceae). (PDF) [file pone.0077982.s039.pdf]

Plantaginaceae

( A ) rbcL

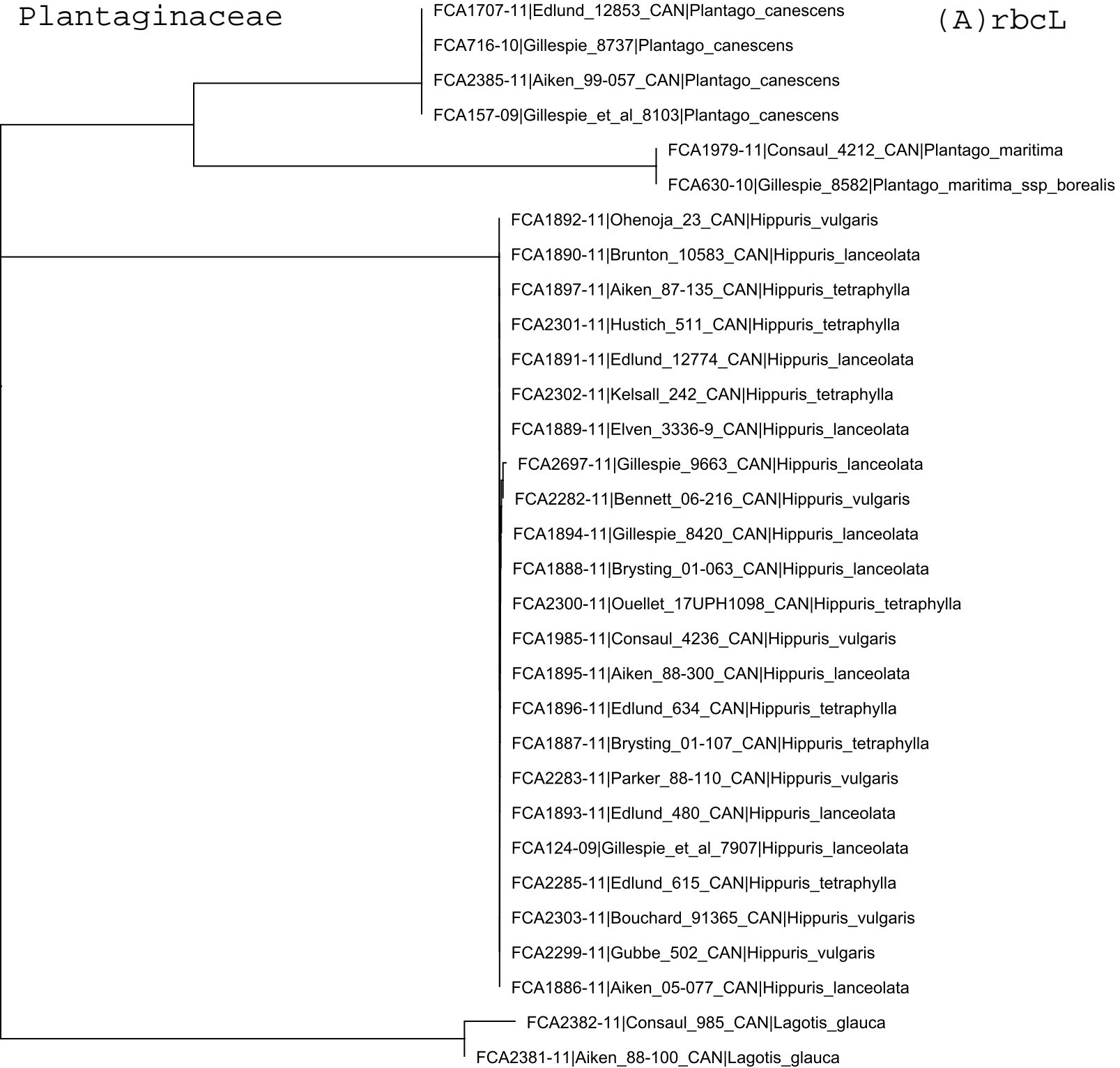

0.0050

Plantaginaceae (B)matK

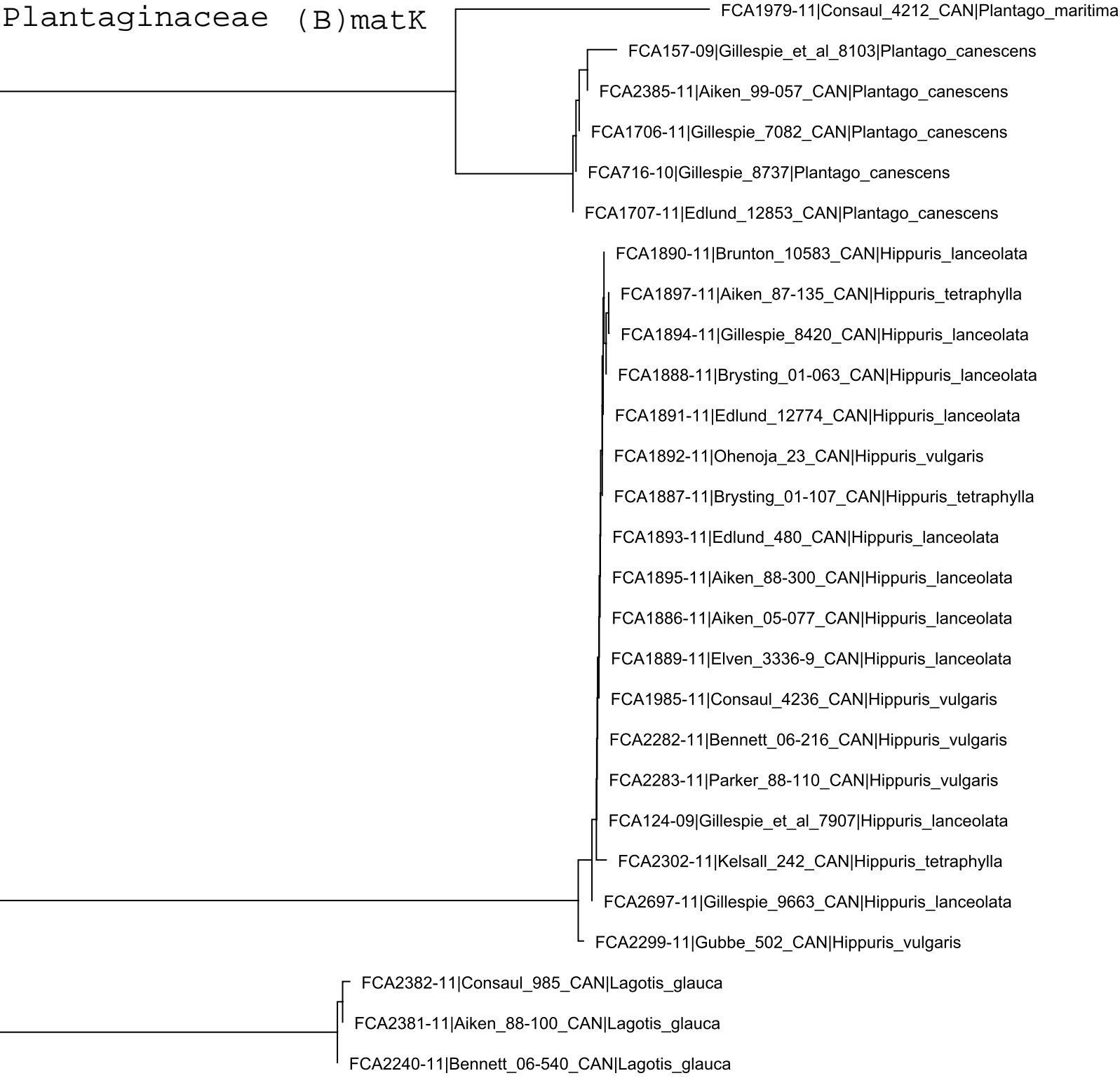

0.02

Plantaginaceae (C)rbcL + matK

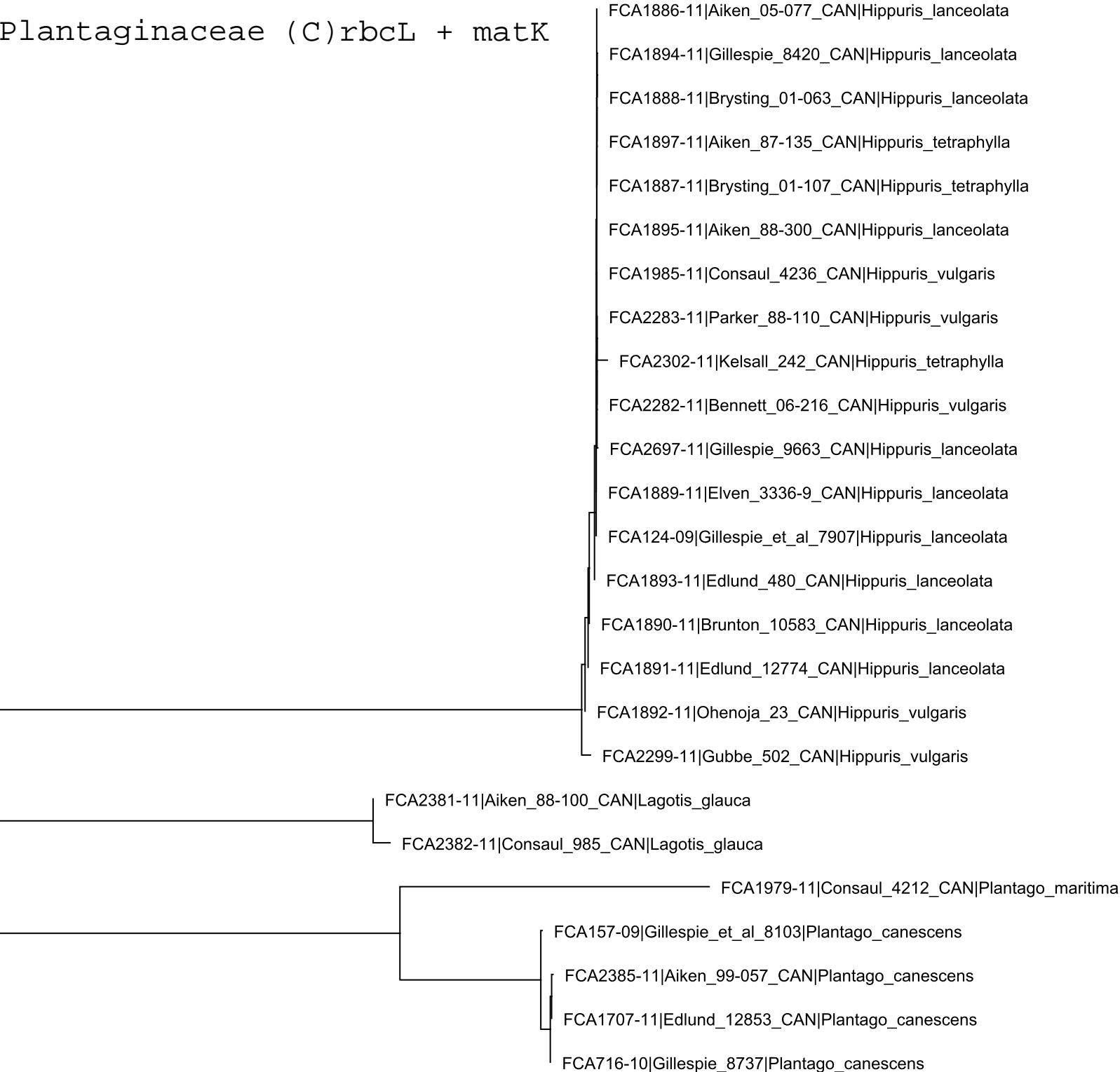

0.0090

Supplement: Figure S35 — Neighbour joining analyses of uncorrected p-distances of rbcL and matK sequence data for Plantaginaceae. A. rbcL. B. matK. C. rbcL + matK. (PDF) [file pone.0077982.s040.pdf]

Plumbaginaceae

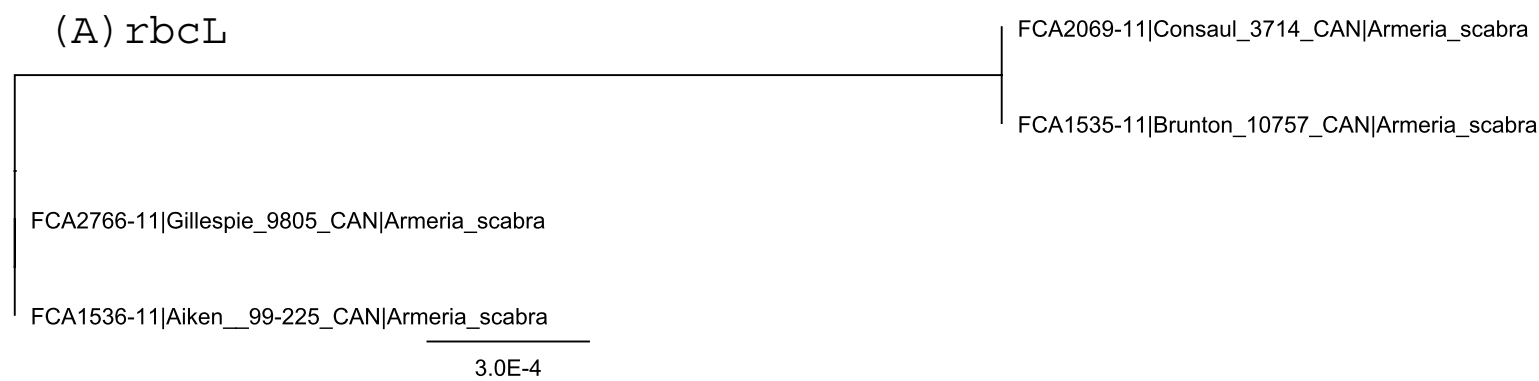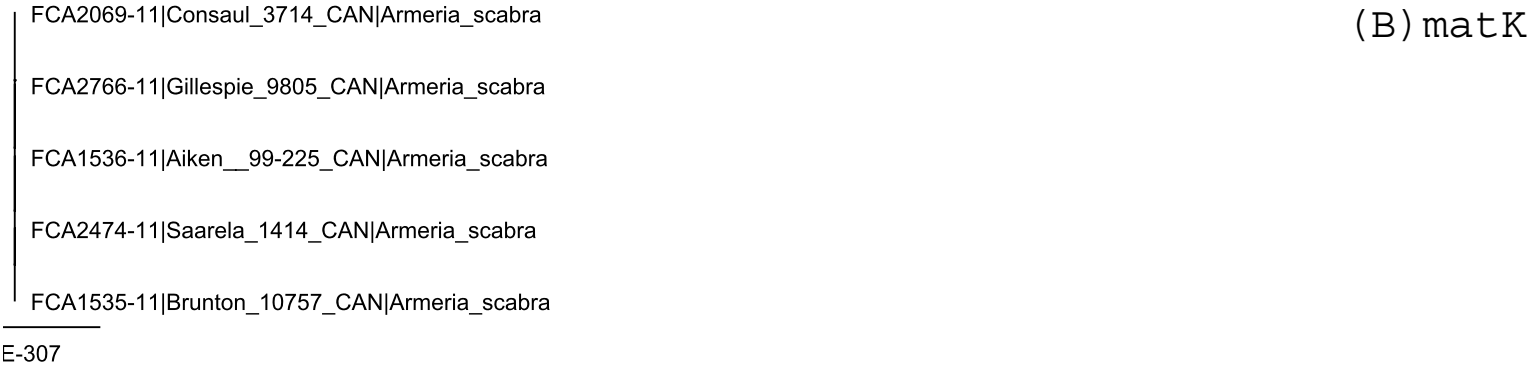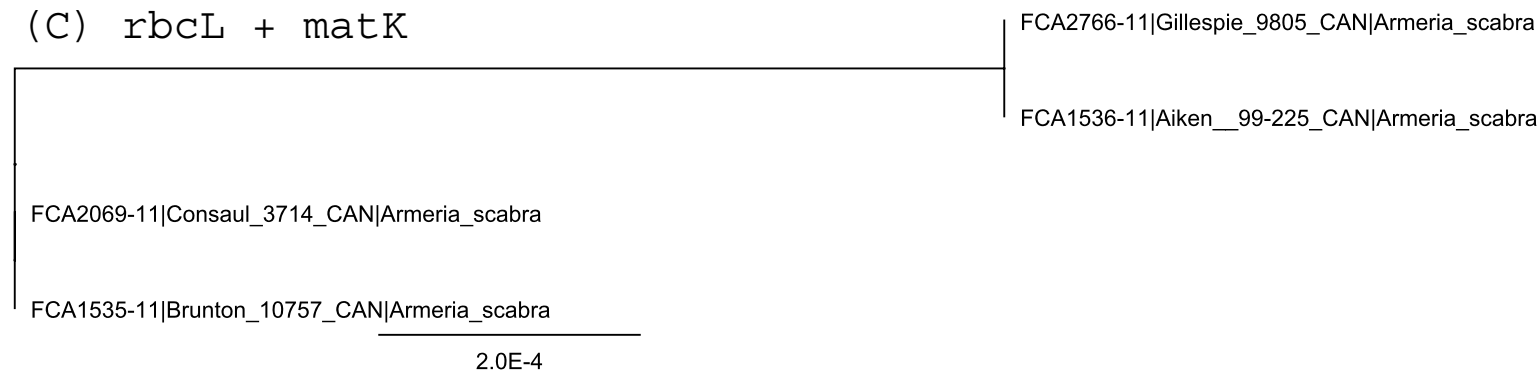

Supplement: Figure S36 — Neighbour joining analyses of uncorrected p-distances of rbcL and matK sequence data for Plumbaginaceae. A. rbcL. B. matK. C. rbcL + matK. (PDF) [file pone.0077982.s041.pdf]

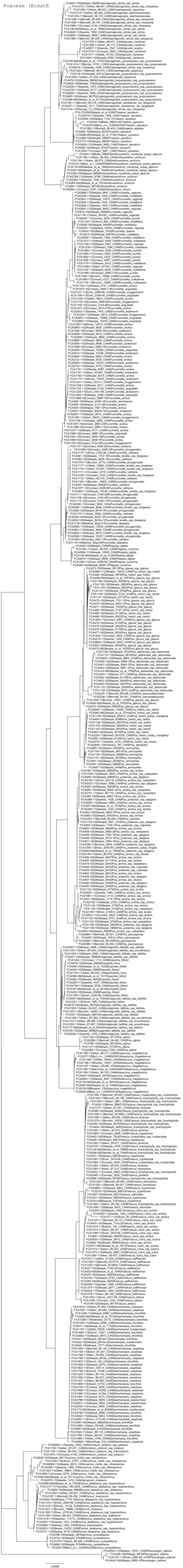

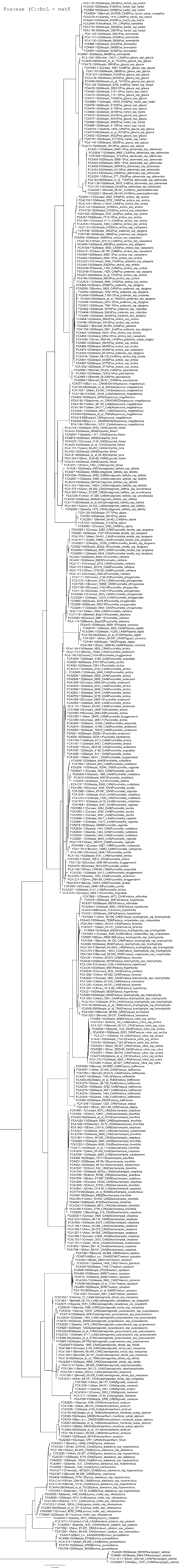

Supplement: Figure S37 — Neighbour joining analyses of uncorrected p-distances of rbcL and matK sequence data for Poaceae. A. rbcL. B. matK. C. rbcL + matK. (PDF) [file pone.0077982.s042.pdf]

Polemoniaceae

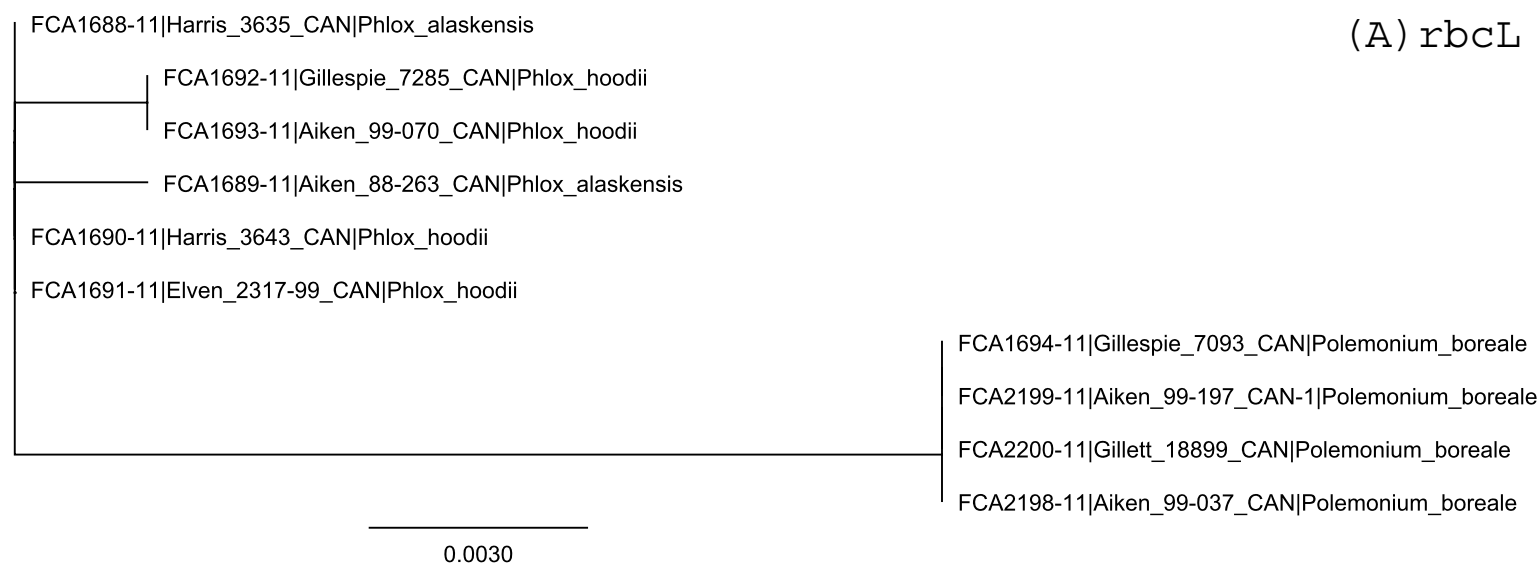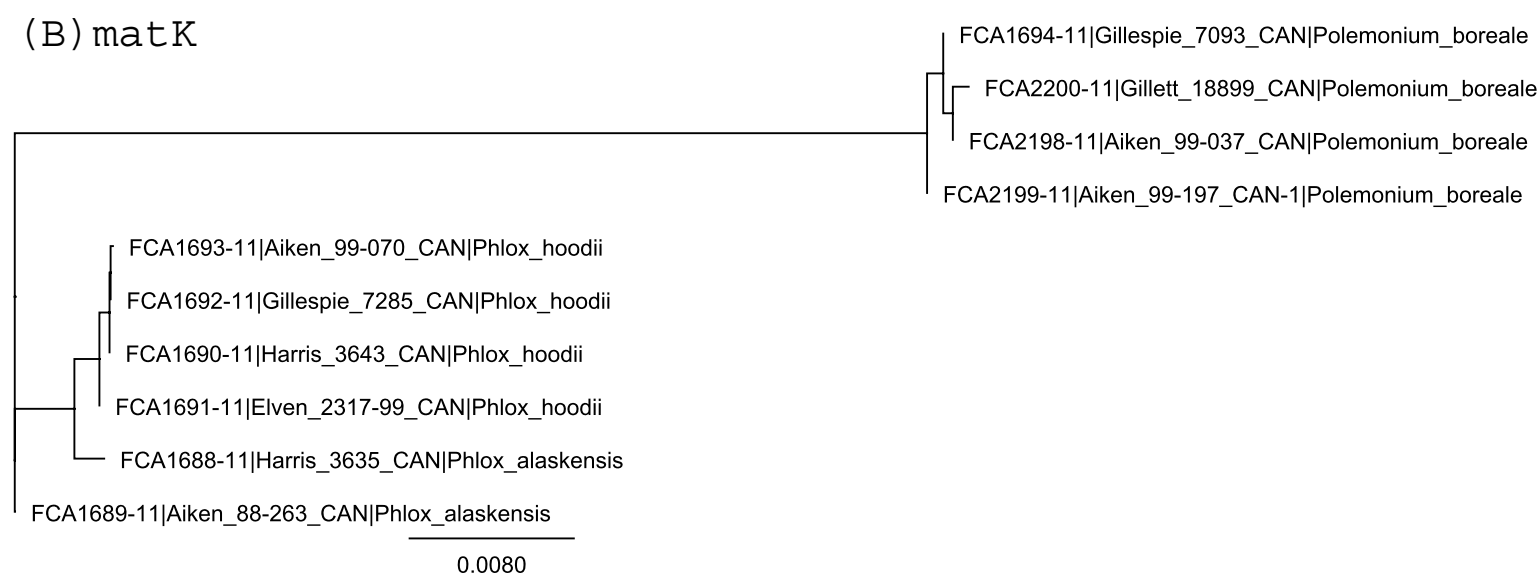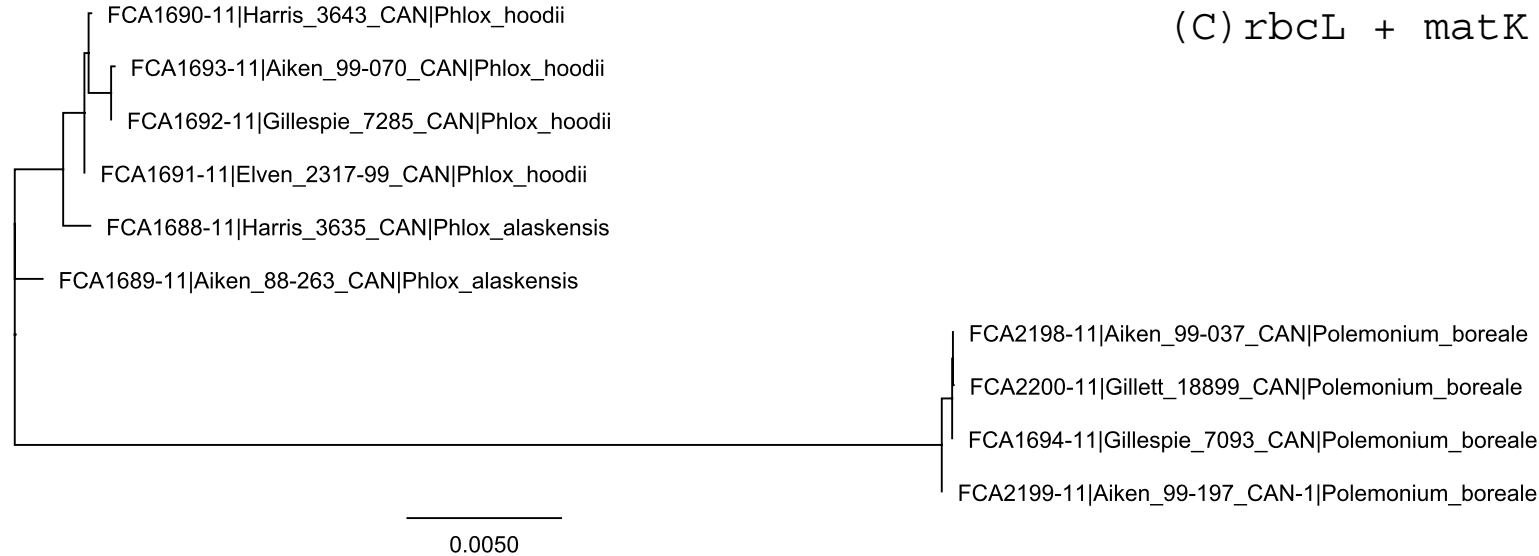

Supplement: Figure S38 — Neighbour joining analyses of uncorrected p-distances of rbcL and matK sequence data for Polemoniaceae. A. rbcL. B. matK. C. rbcL + matK. (PDF) [file pone.0077982.s043.pdf]

(A) rbcL

Polygonaceae

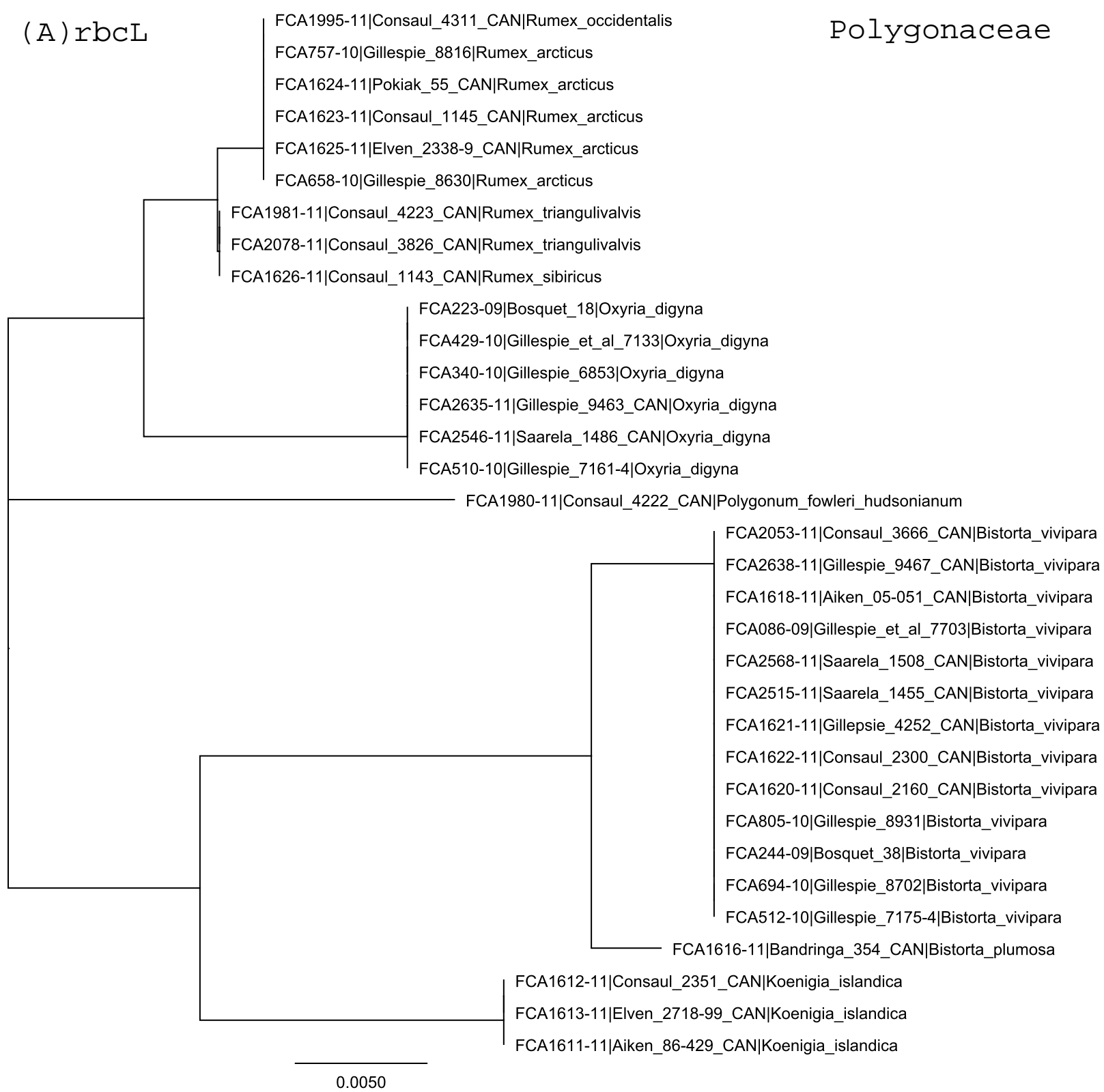

Polygonaceae

(B) matK

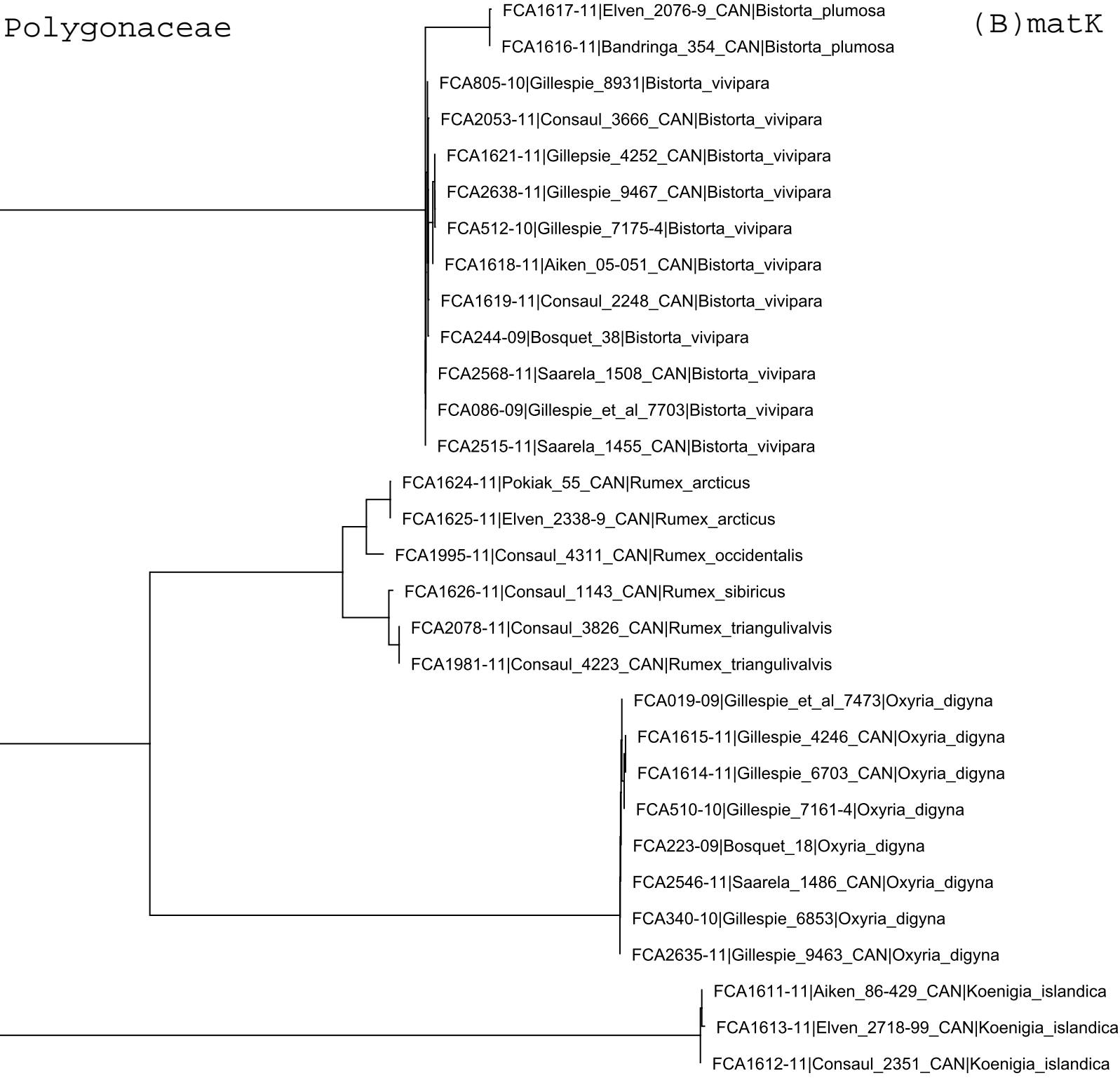

0.02

Polygonaceae (C) rbcL + matK

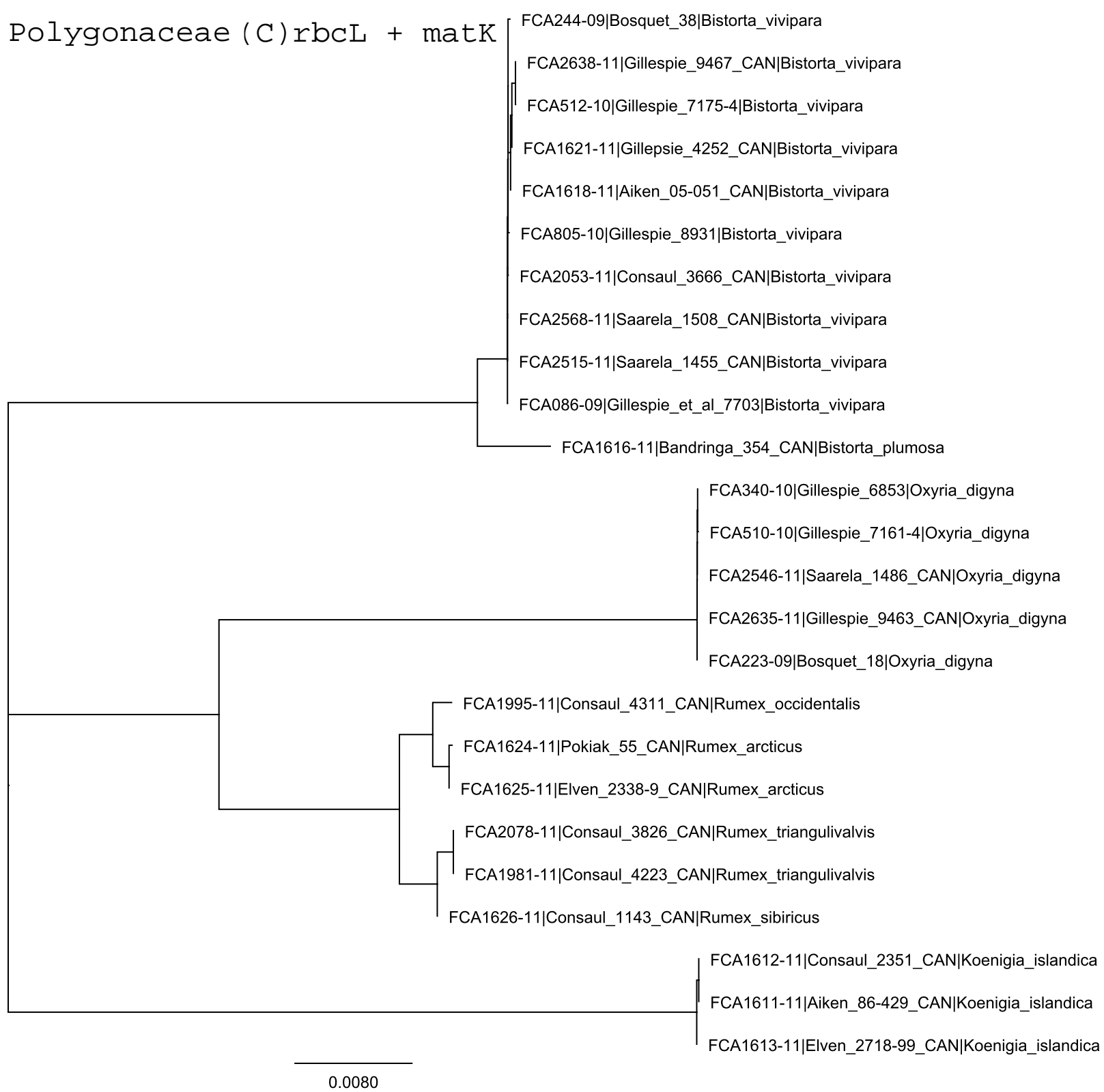

Supplement: Figure S39 — Neighbour joining analyses of uncorrected p-distances of rbcL and matK sequence data for Polygonaceae. A. rbcL. B. matK. C. rbcL + matK. (PDF) [file pone.0077982.s044.pdf]

Potamogetonaceae

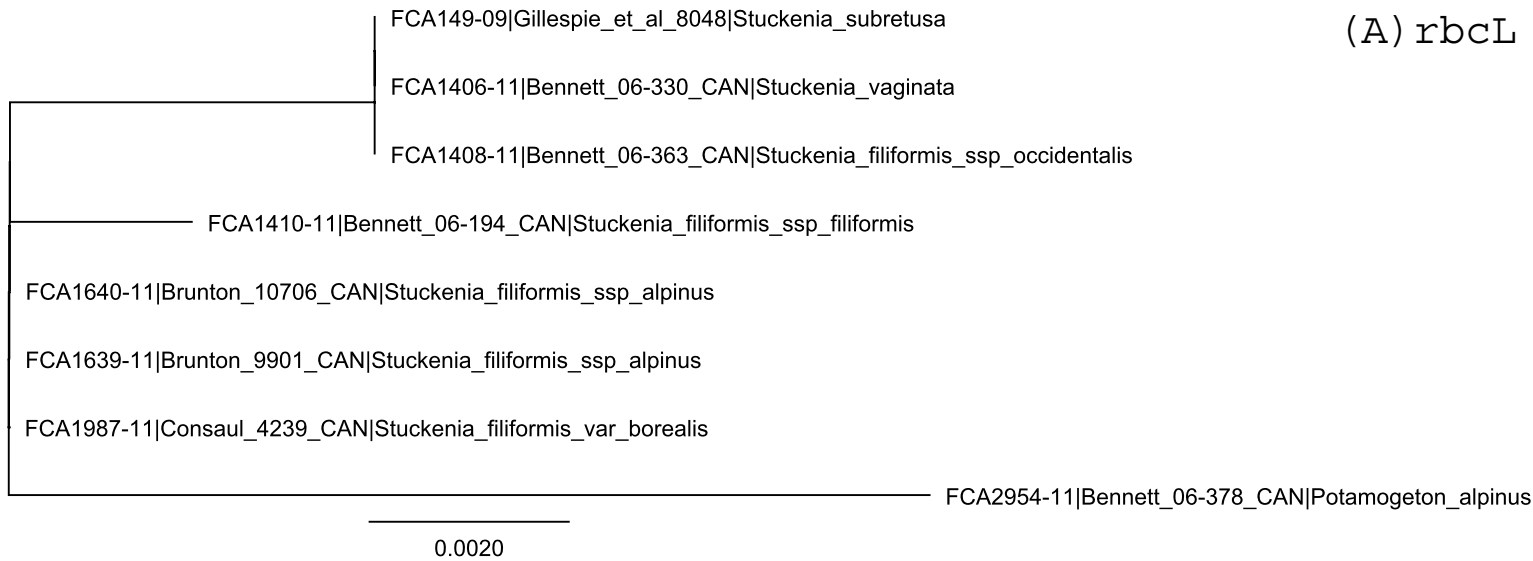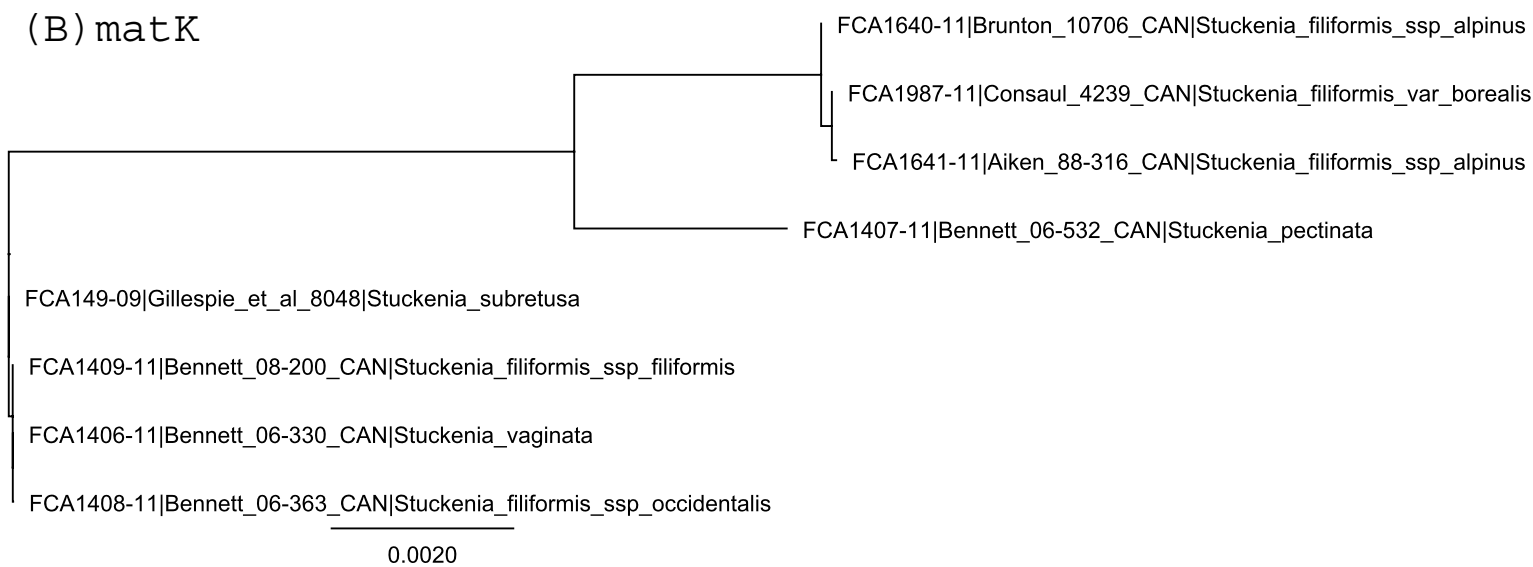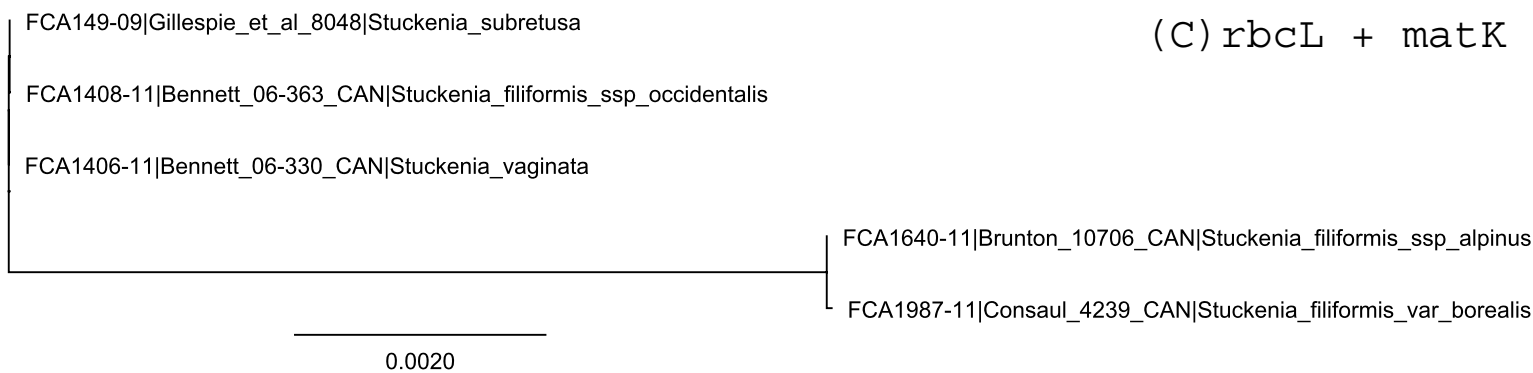

Supplement: Figure S40 — Neighbour joining analyses of uncorrected p-distances of rbcL and matK sequence data for Potamogetonaceae. A. rbcL. B. matK. C. rbcL + matK. (PDF) [file pone.0077982.s045.pdf]

Primulaceae (A) rbcL

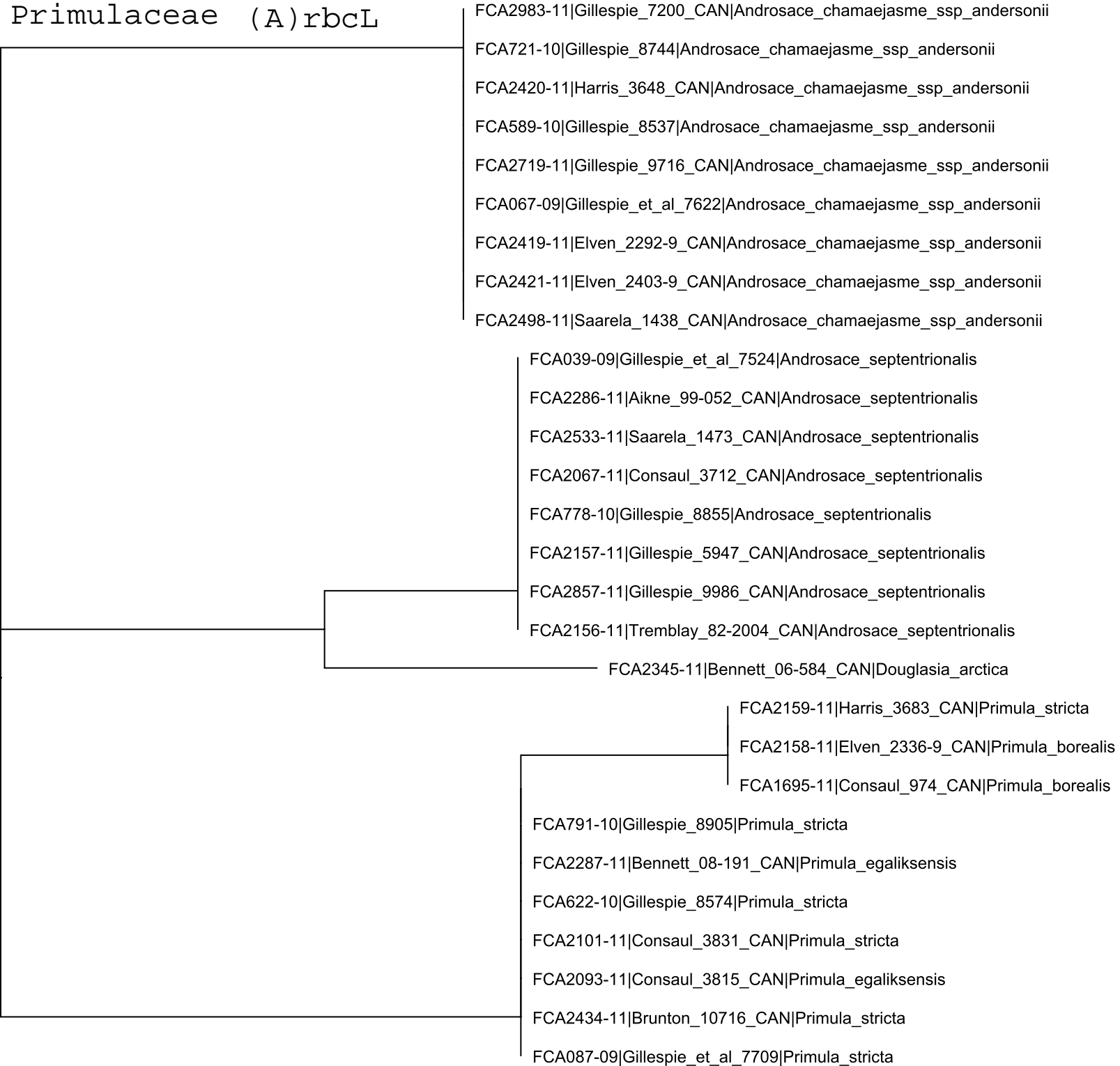

0.0040

Primulaceae (B)matK

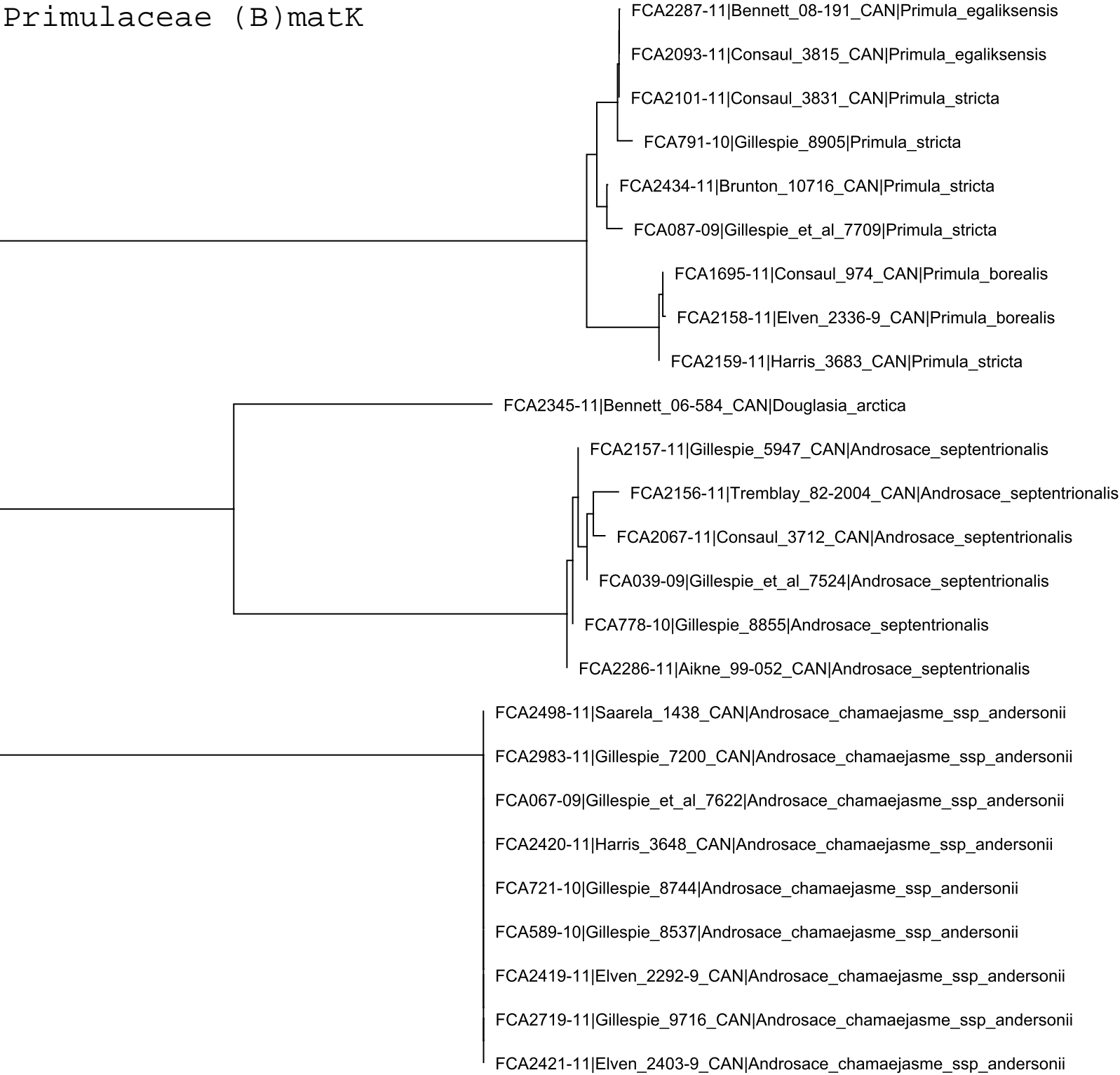

0.01

Primulaceae (C)rbcL + matK

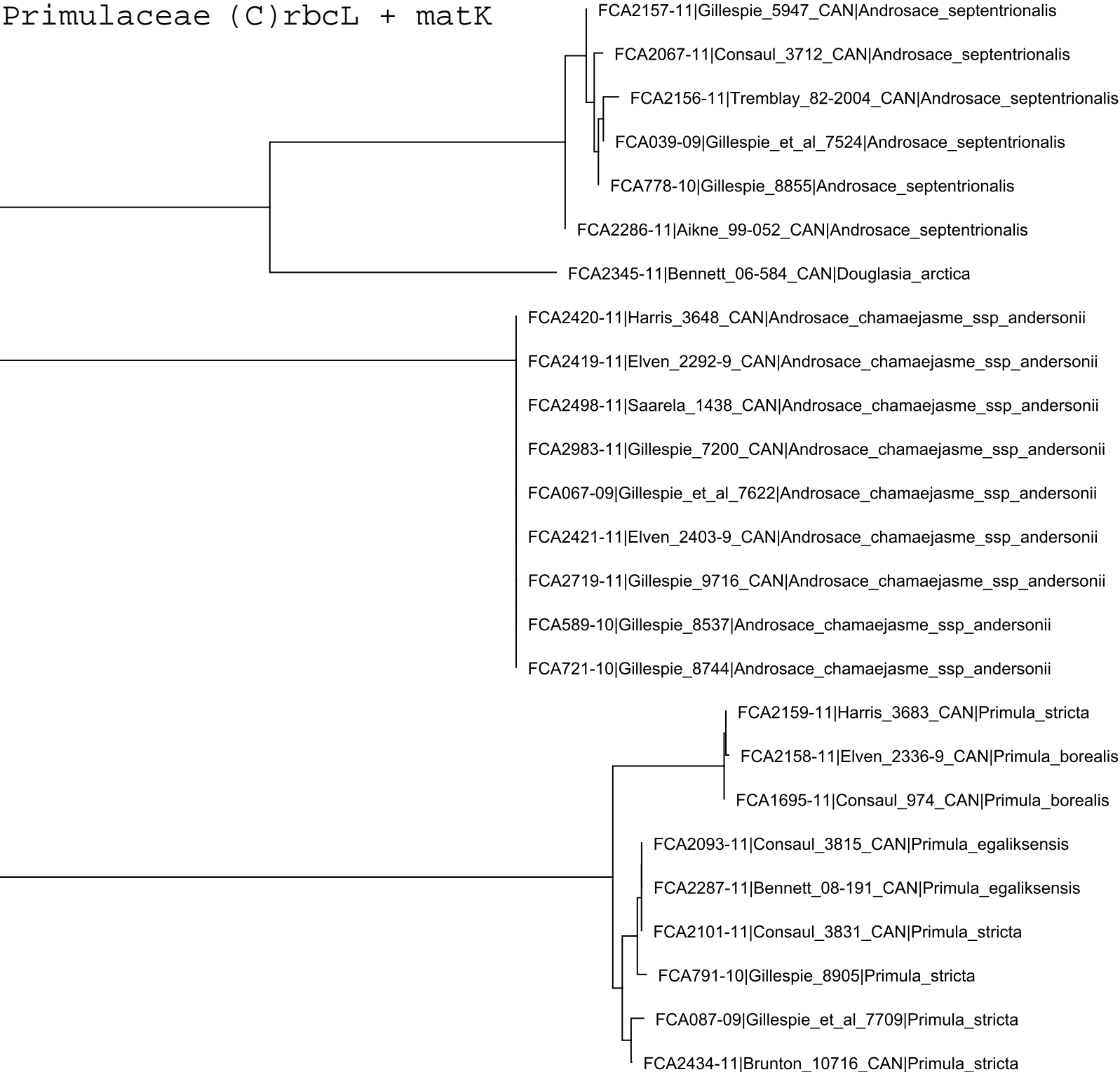

0.0070

Supplement: Figure S41 — Neighbour joining analyses of uncorrected p-distances of rbcL and matK sequence data for Primulaceae. A. rbcL. B. matK. C. rbcL + matK. (PDF) [file pone.0077982.s046.pdf]

Rosaceae

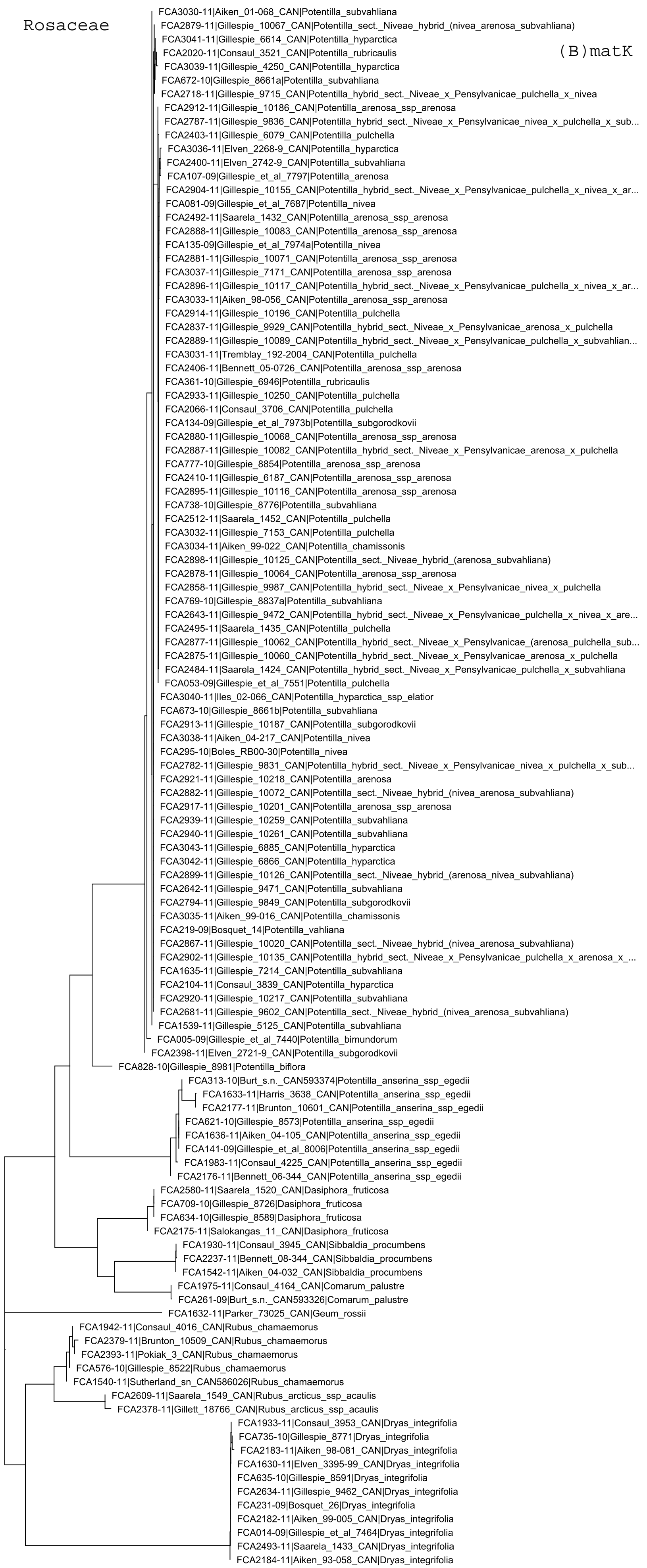

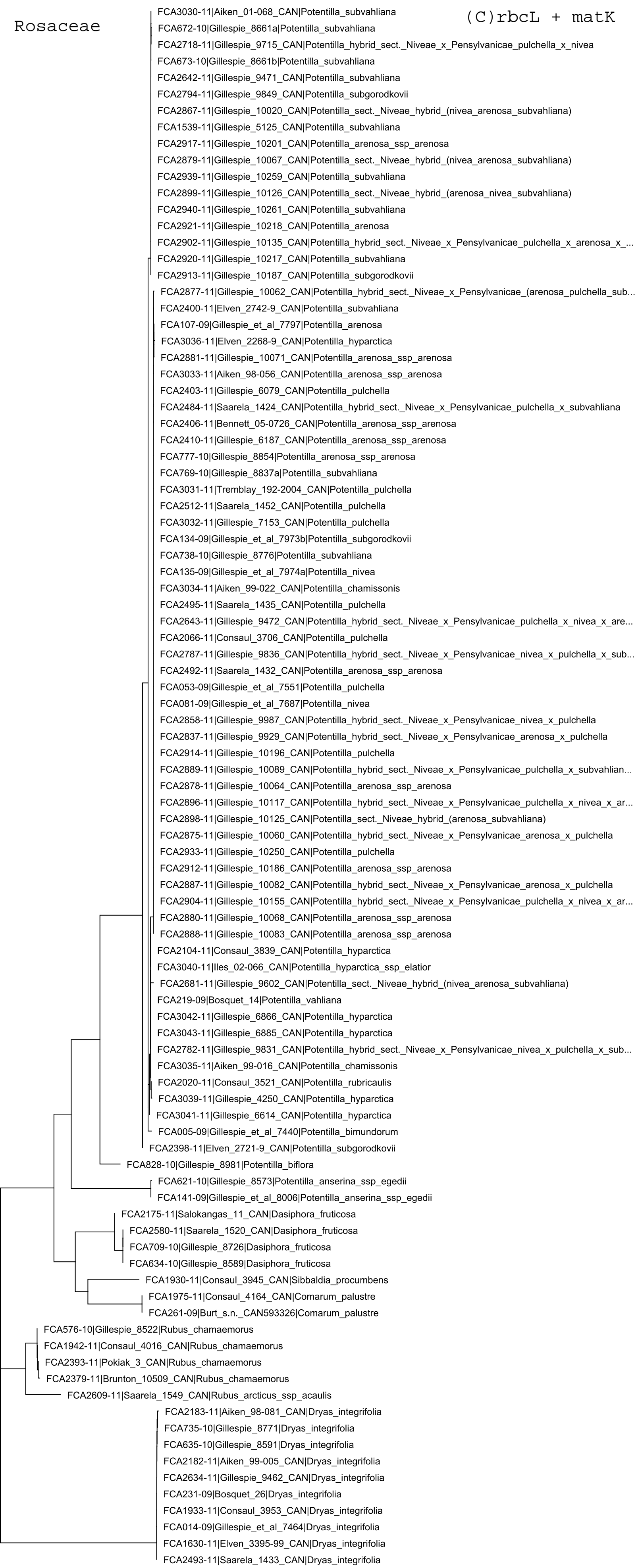

Supplement: Figure S43 — Neighbour joining analyses of uncorrected p-distances of rbcL and matK sequence data for Rosaceae. A. rbcL. B. matK. C. rbcL + matK. (PDF) [file pone.0077982.s048.pdf]

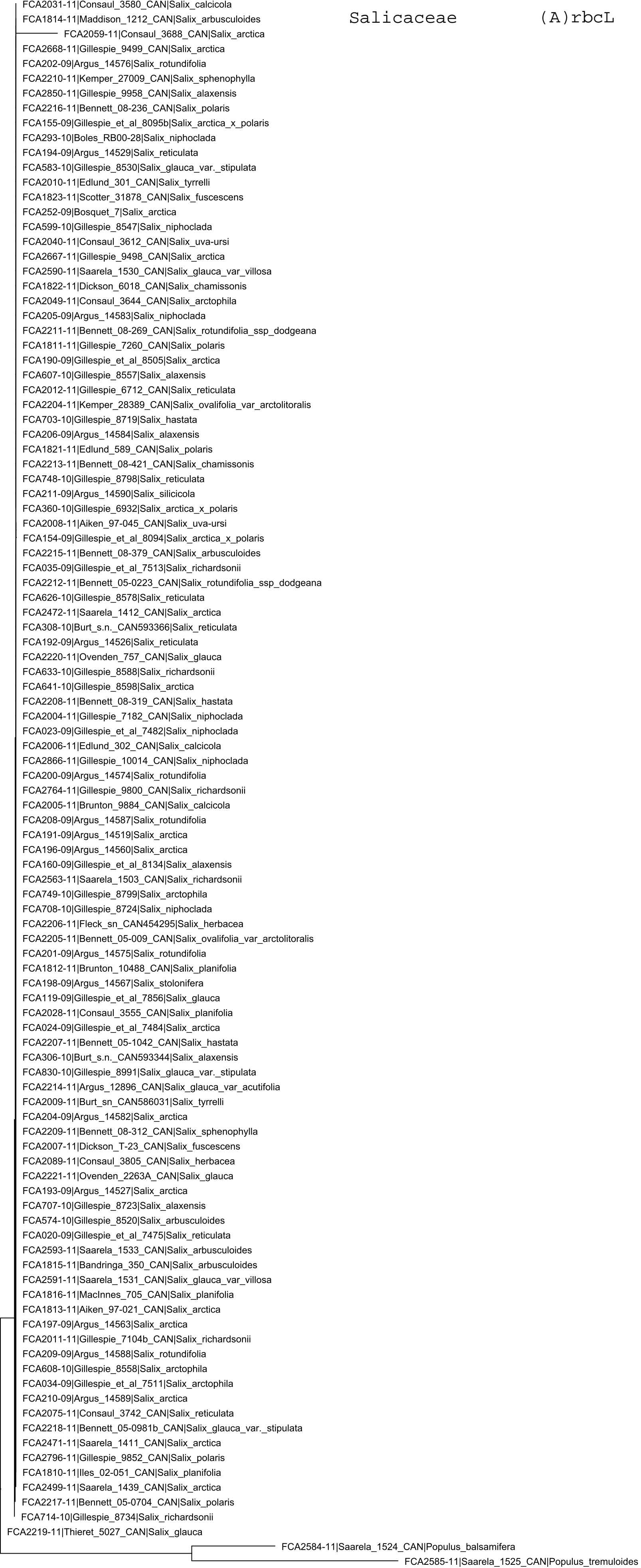



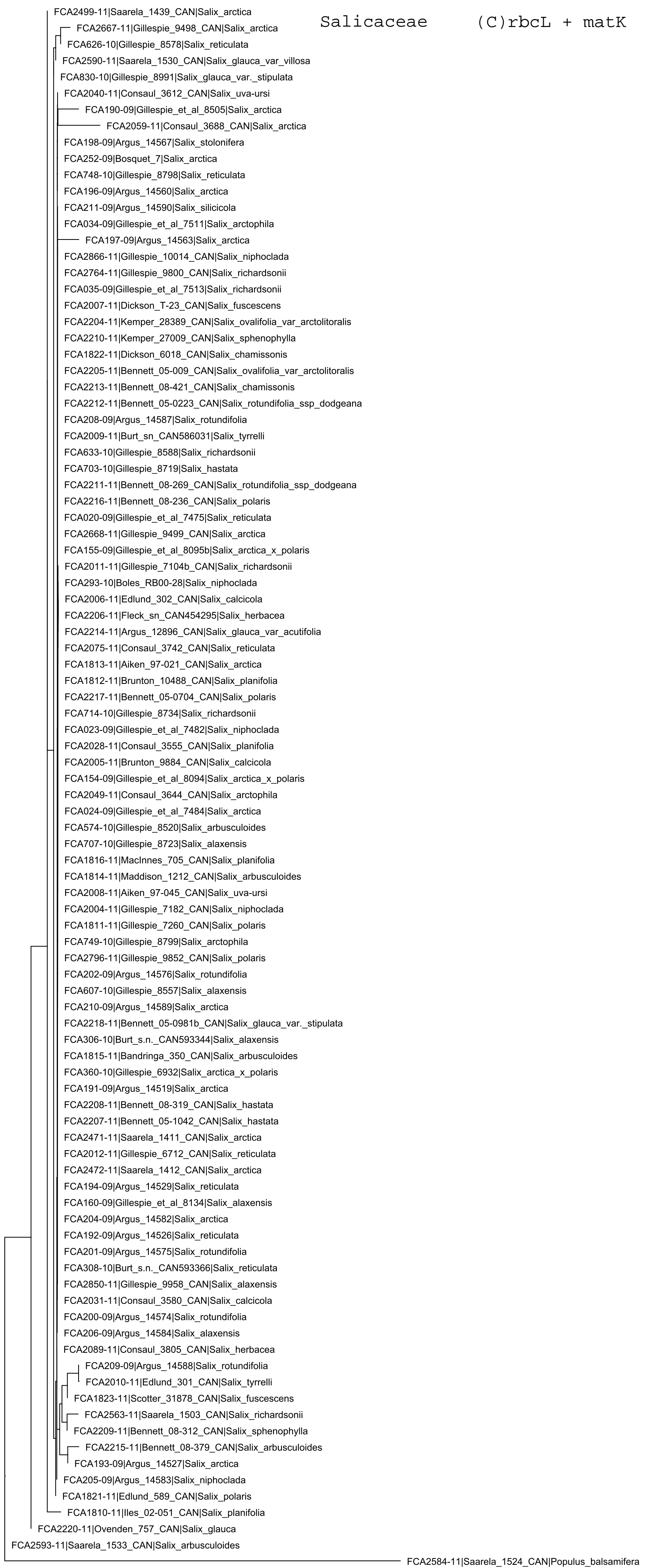

Supplement: Figure S44 — Neighbour joining analyses of uncorrected p-distances of rbcL and matK sequence data for Salicaceae. A. rbcL. B. matK. C. rbcL + matK. (PDF) [file pone.0077982.s049.pdf]

Saxifragaceae

(A) rbcL

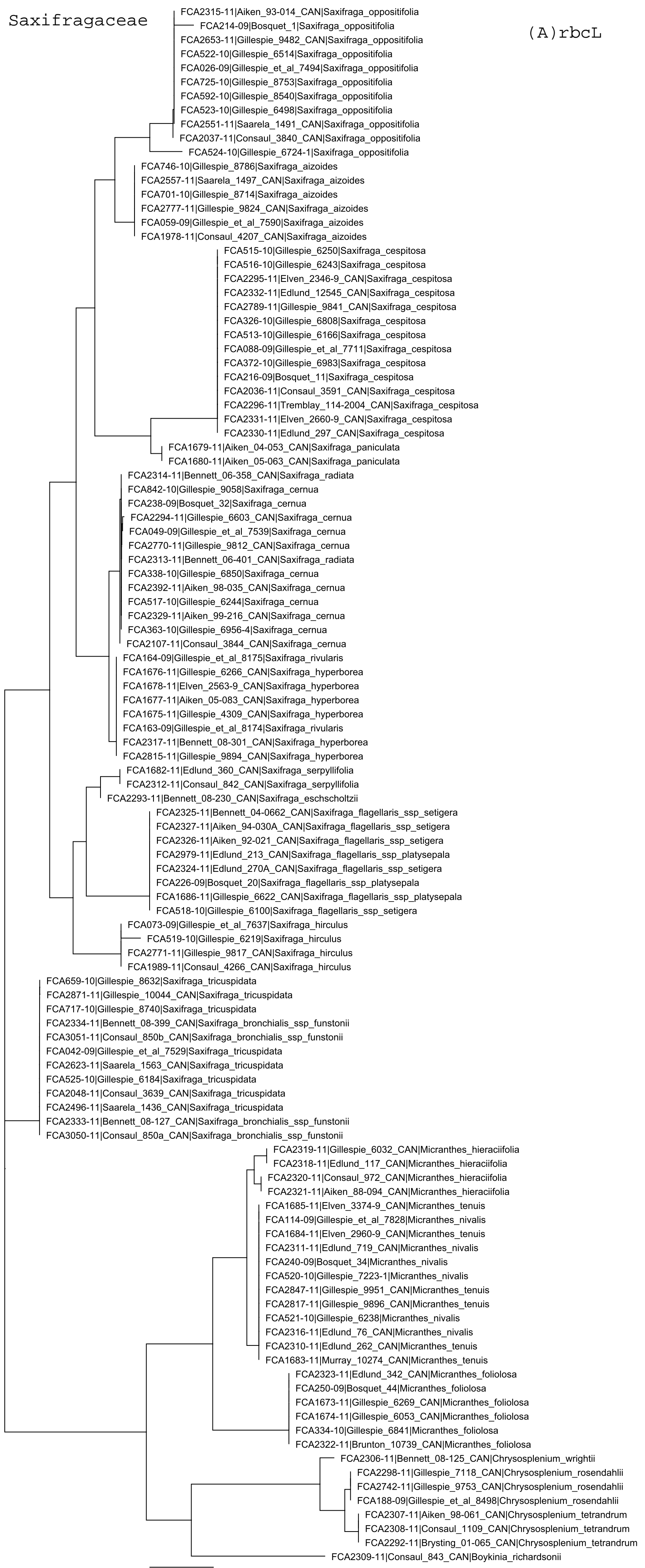

Saxifragaceae (B)matK

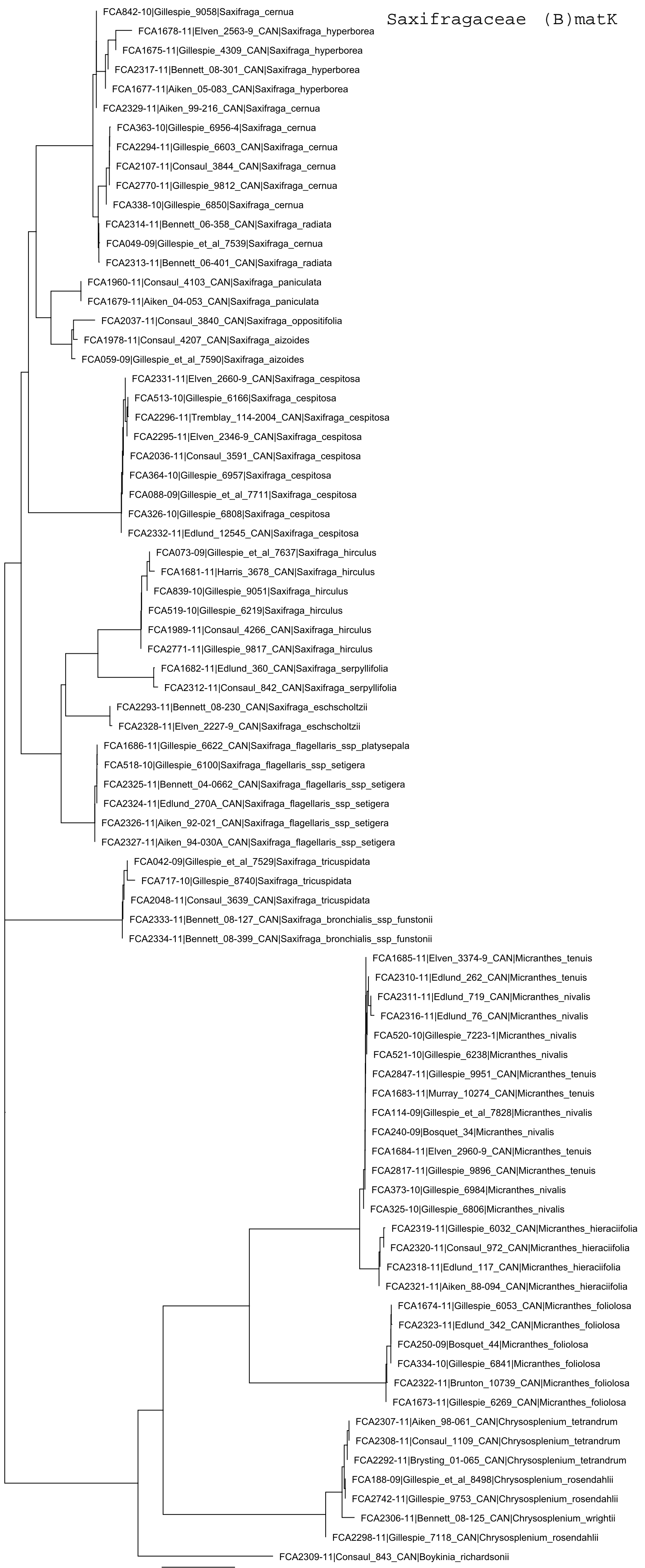

0.02

Saxifragaceae

(C) rbcL + matK

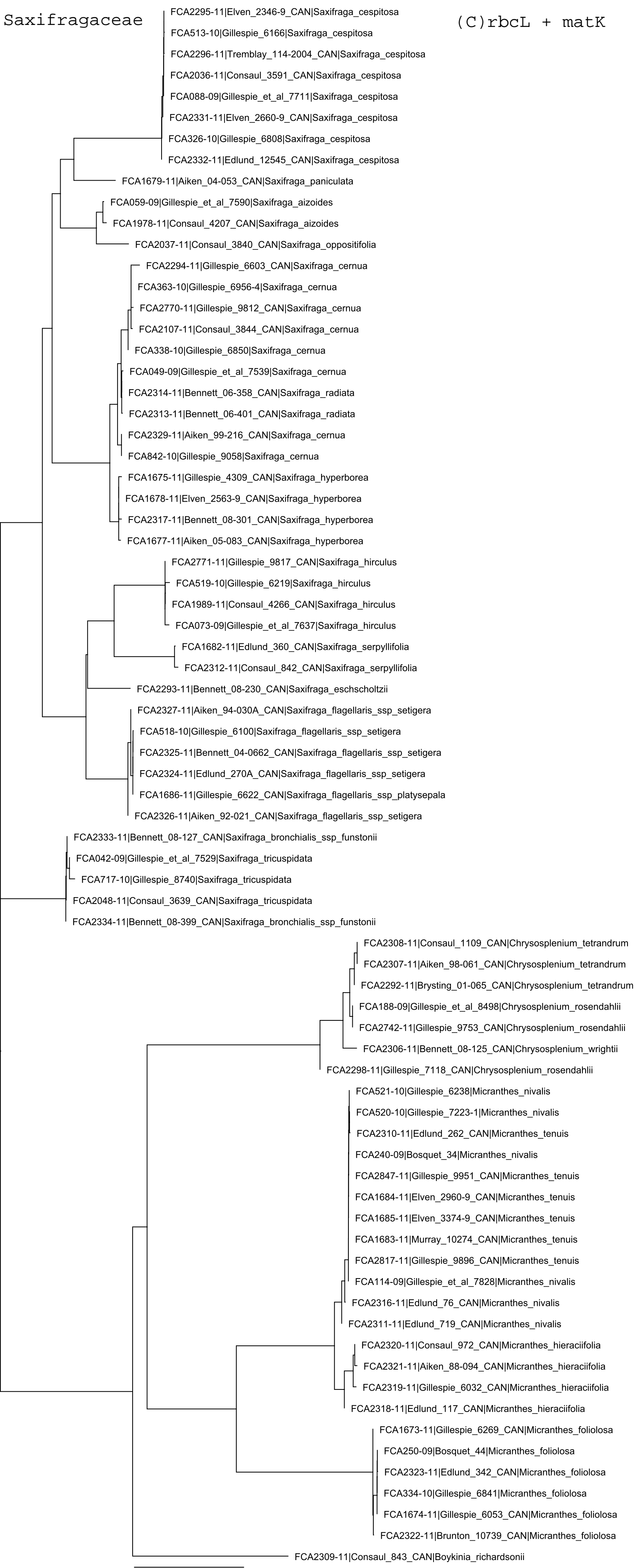

0.02

Supplement: Figure S45 — Neighbour joining analyses of uncorrected p-distances of rbcL and matK sequence data for Saxifragaceae. A. rbcL. B. matK. C. rbcL + matK. (PDF) [file pone.0077982.s050.pdf]

# Tofieldiaceae

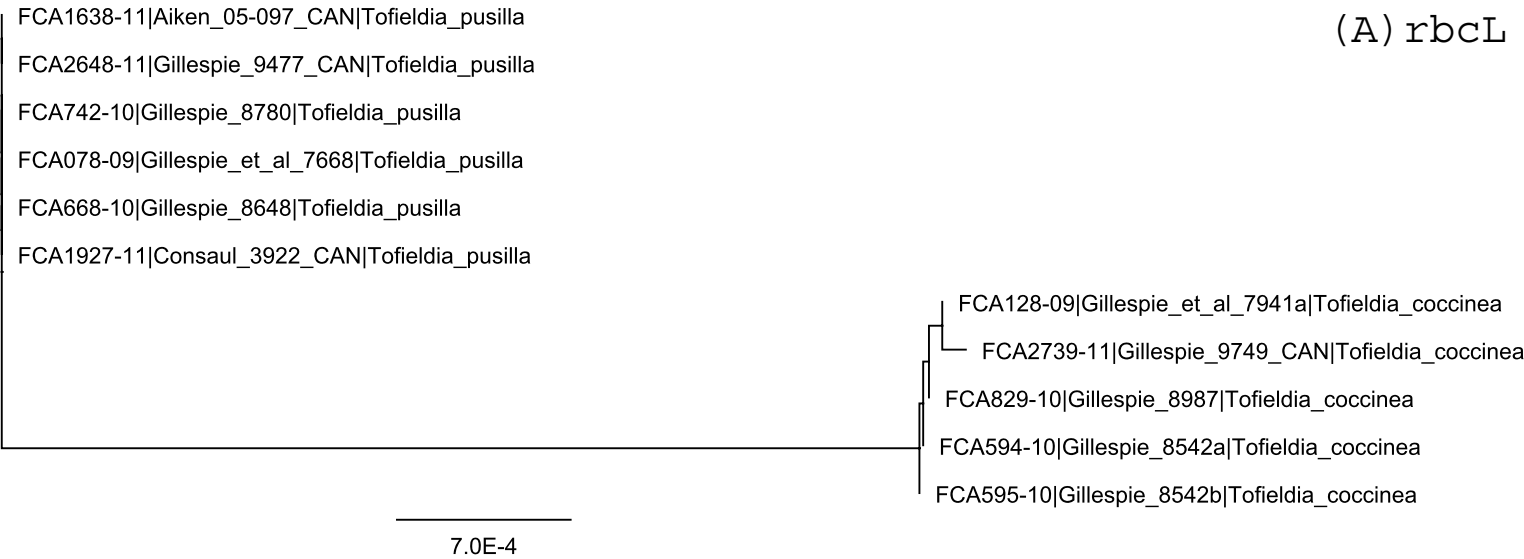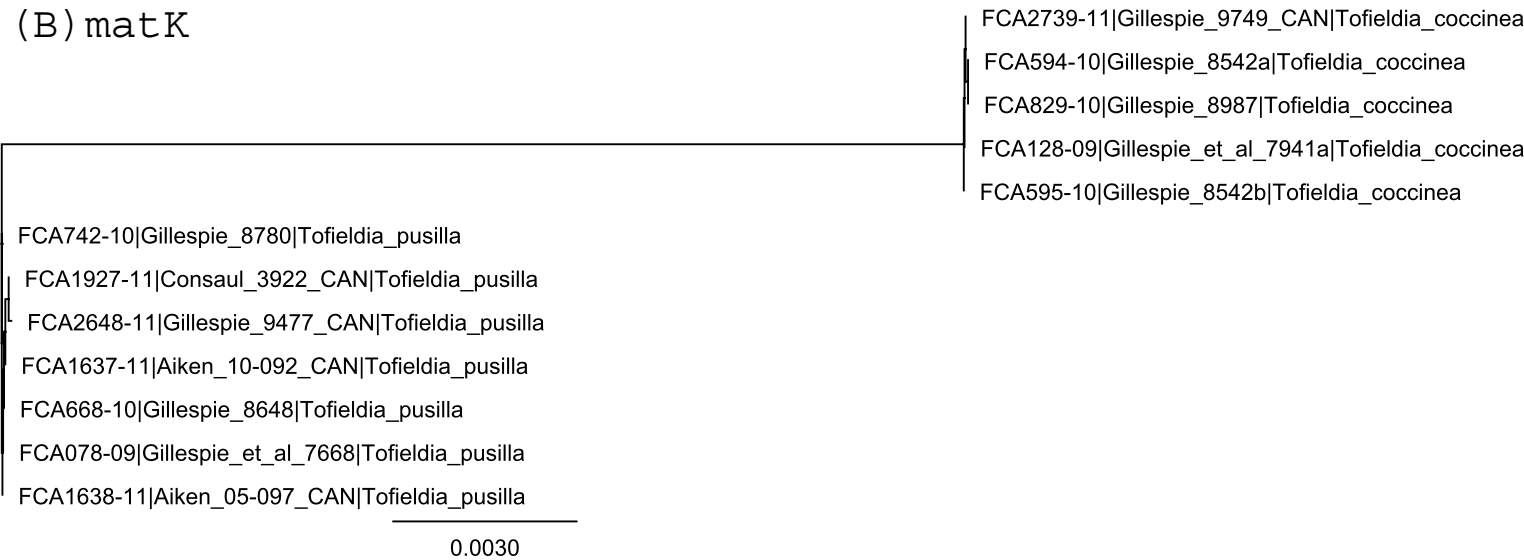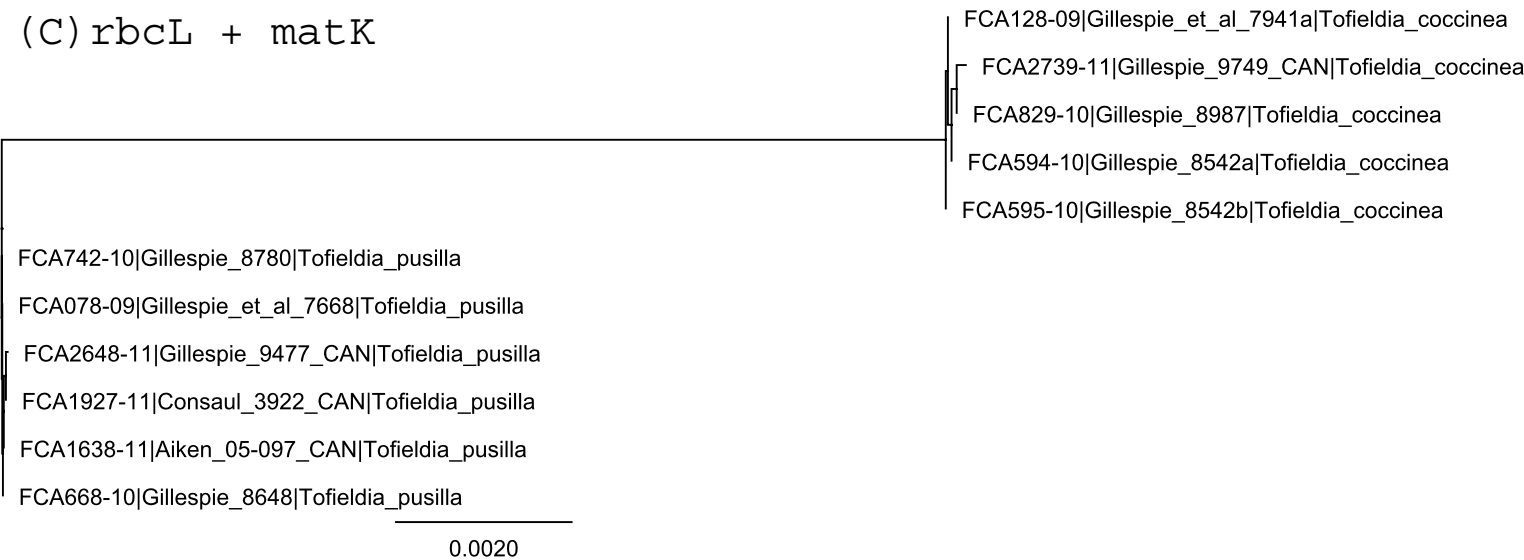

Supplement: Figure S46 — Neighbour joining analyses of uncorrected p-distances of rbcL and matK sequence data for Tofieldiaceae. A. rbcL. B. matK. C. rbcL + matK. (PDF) [file pone.0077982.s051.pdf]

Typhaceae

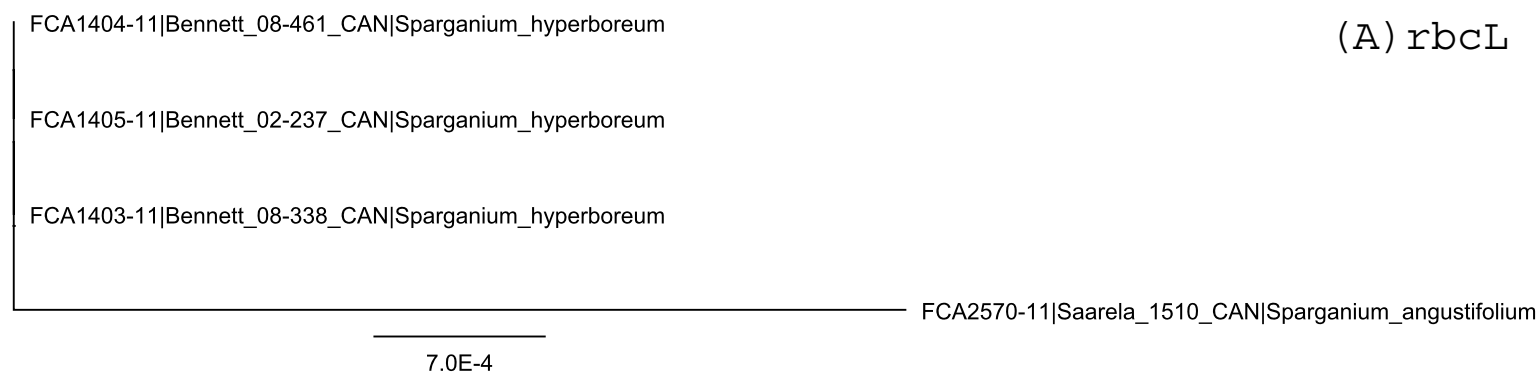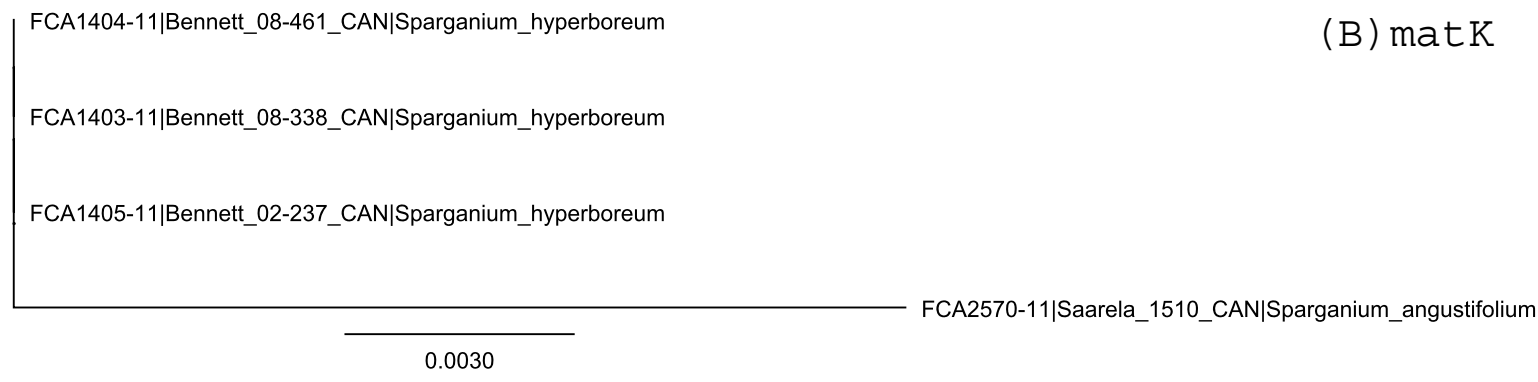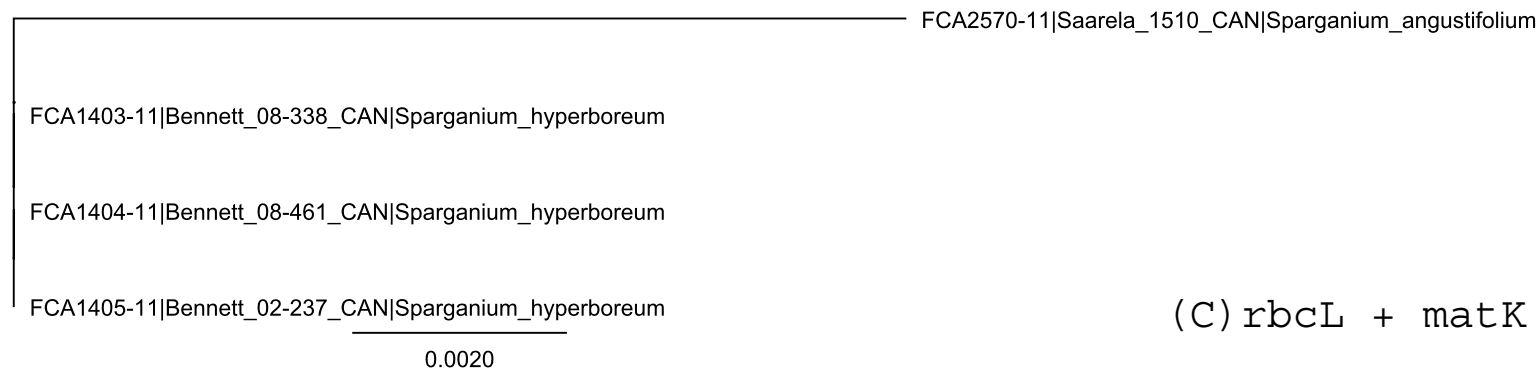

Supplement: Figure S47 — Neighbour joining analyses of uncorrected p-distances of rbcL and matK sequence data for Typhaceae. A. rbcL. B. matK. C. rbcL + matK. (PDF) [file pone.0077982.s052.pdf]

Puccinellia  
rbcL + matK

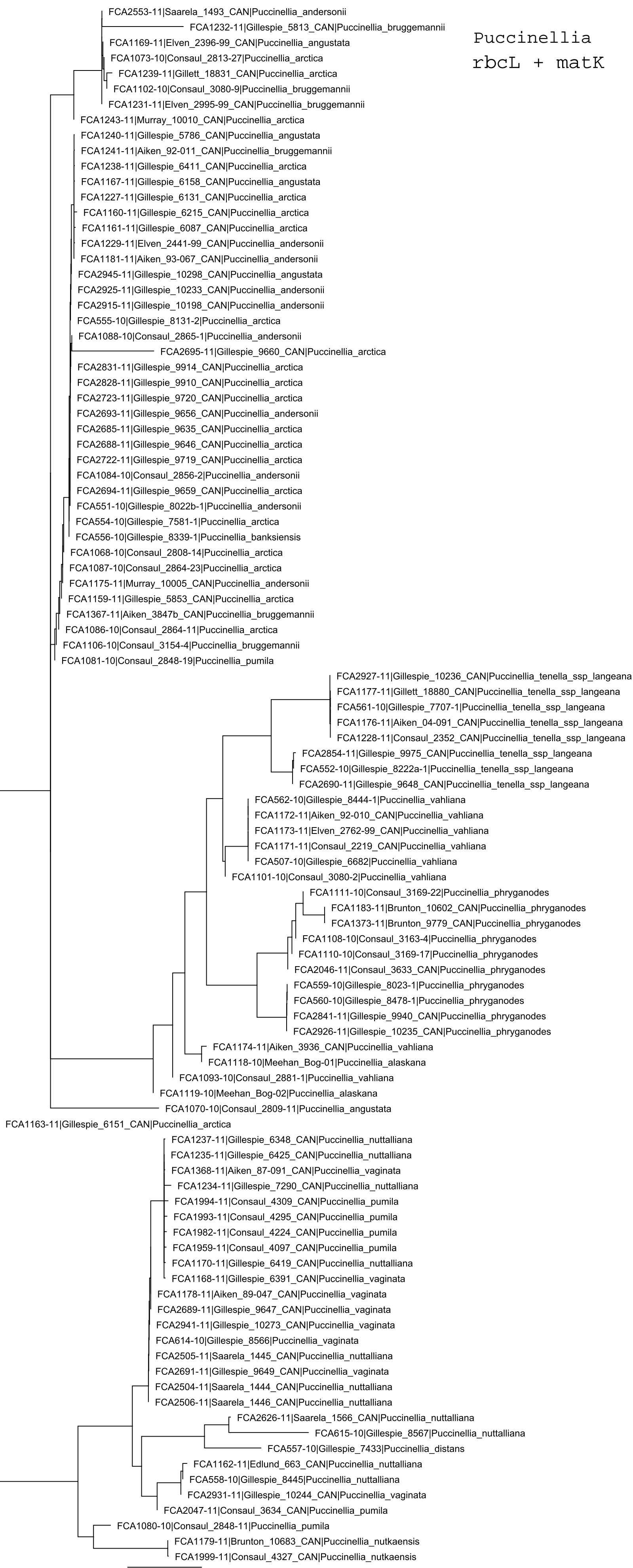

Supplement: Figure S48 — Neighbour joining analysis of uncorrected p-distances of combined rbcL + matK sequence data for Puccinellia (Poaceae). (PDF) [file pone.0077982.s053.pdf]

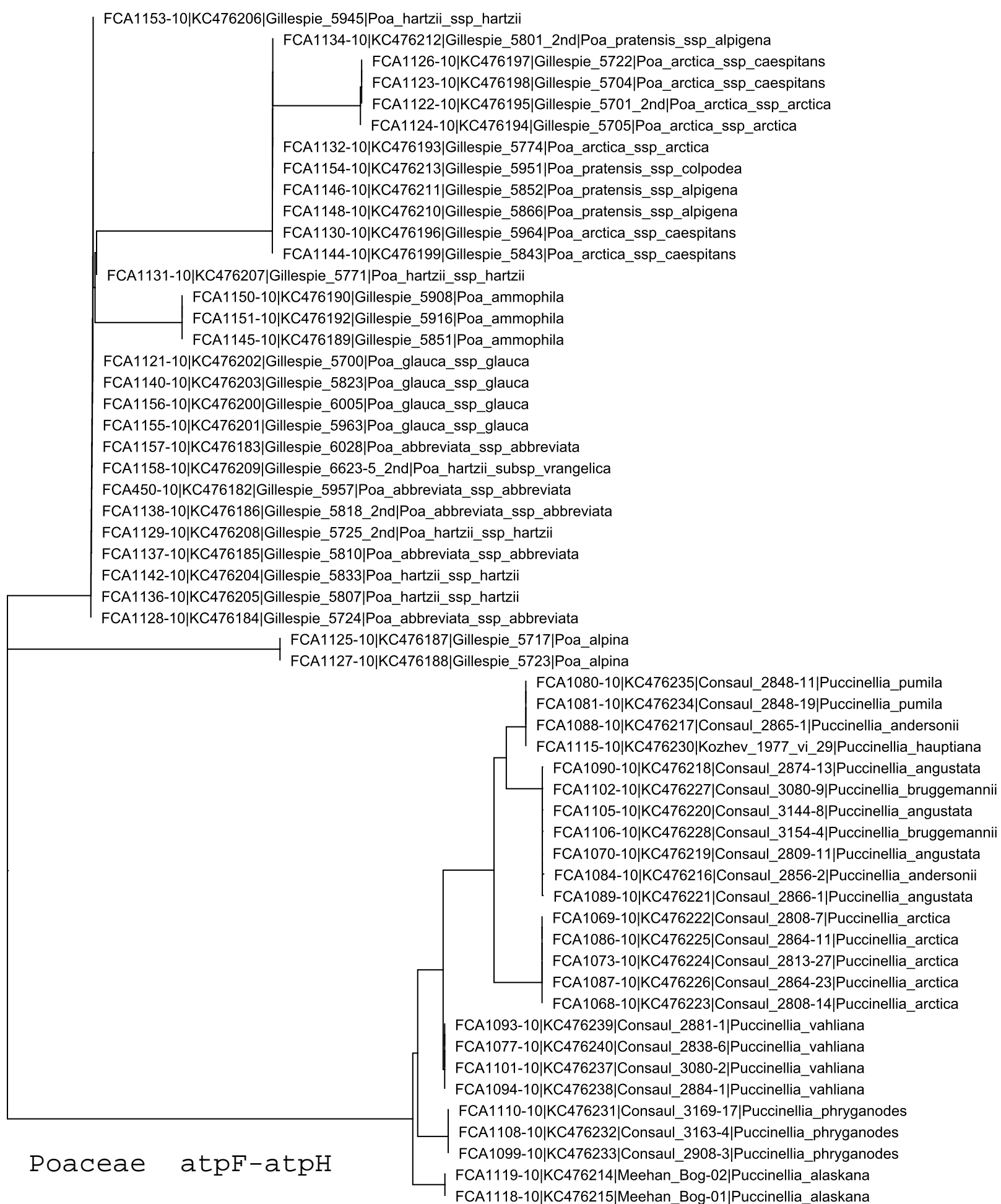

0.0040

Supplement: Figure S50 — Neighbour joining analysis of uncorrected p-distances of atpF–atpH sequence data for Puccinellia and Poa (Poaceae). (PDF) [file pone.0077982.s055.pdf]

Poaceae    psbK-psbI

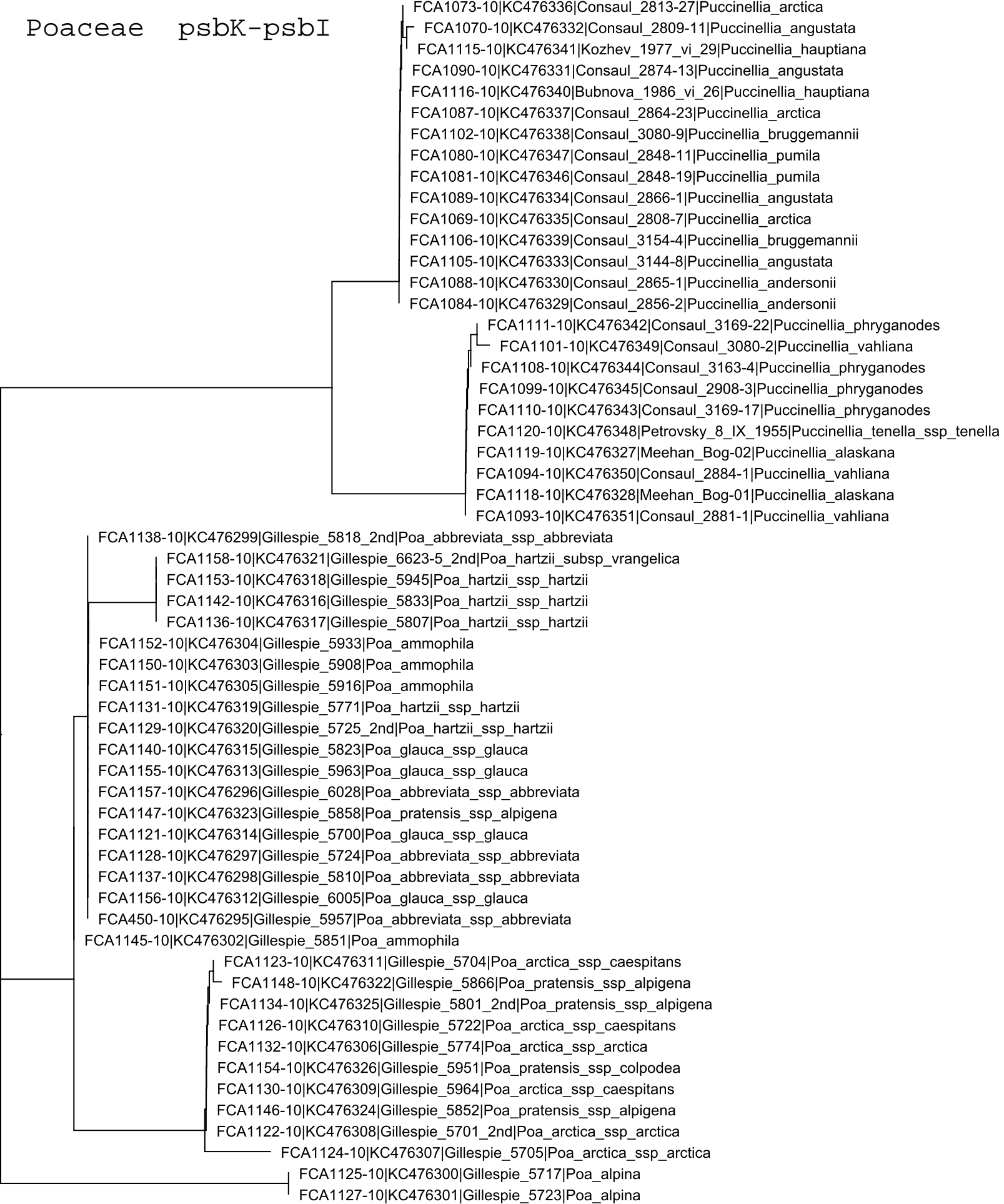

0.0040

Supplement: Figure S51 — Neighbour joining analysis of uncorrected p-distances of psbK–psbI sequence data for Puccinellia and Poa (Poaceae). (PDF) [file pone.0077982.s056.pdf]

Festuca

rbcl + matK

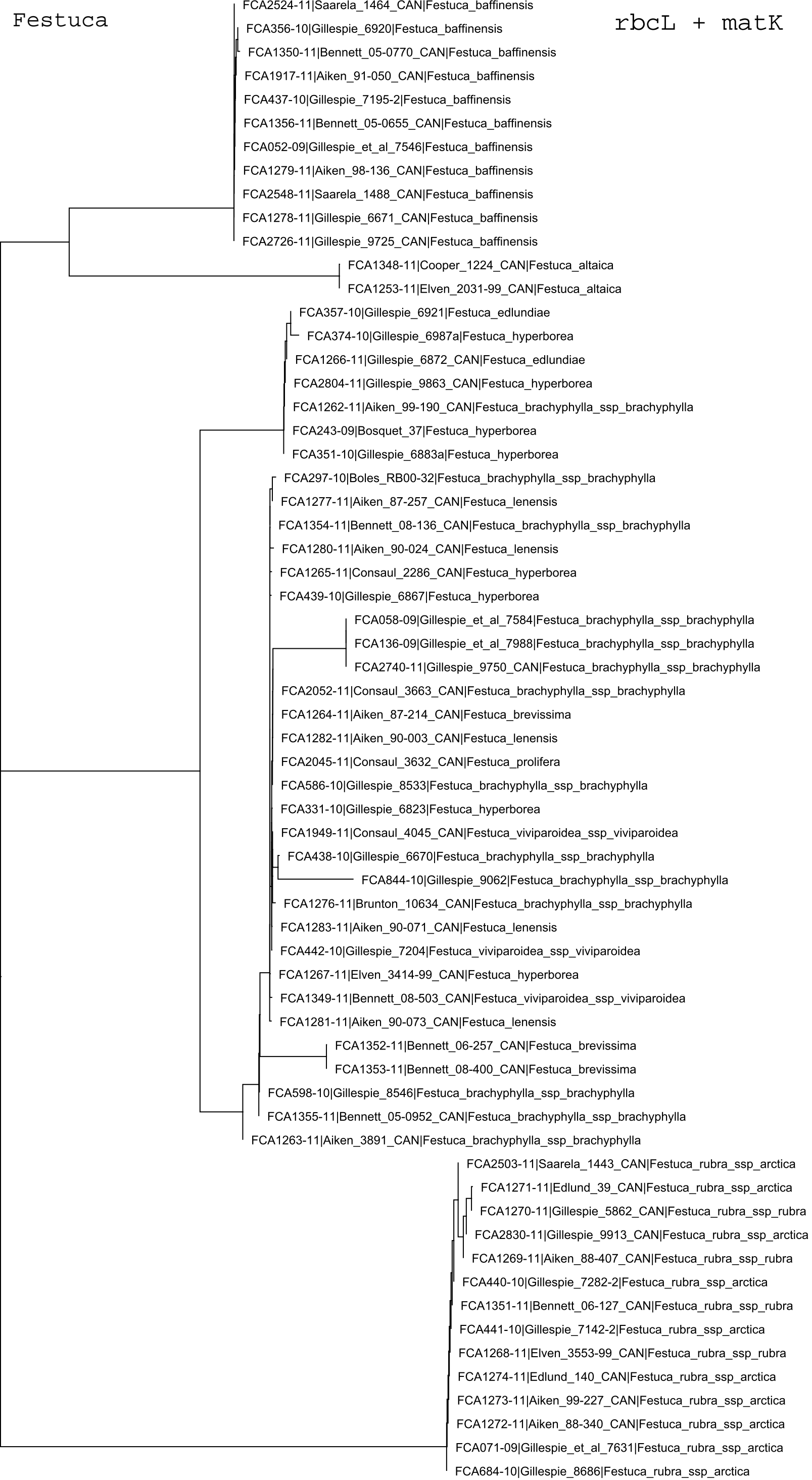

Supplement: Figure S52 — Neighbour joining analysis of uncorrected p-distances of combined rbcL + matK sequence data for Festuca (Poaceae). (PDF) [file pone.0077982.s057.pdf]

rbcl + matK

Poa

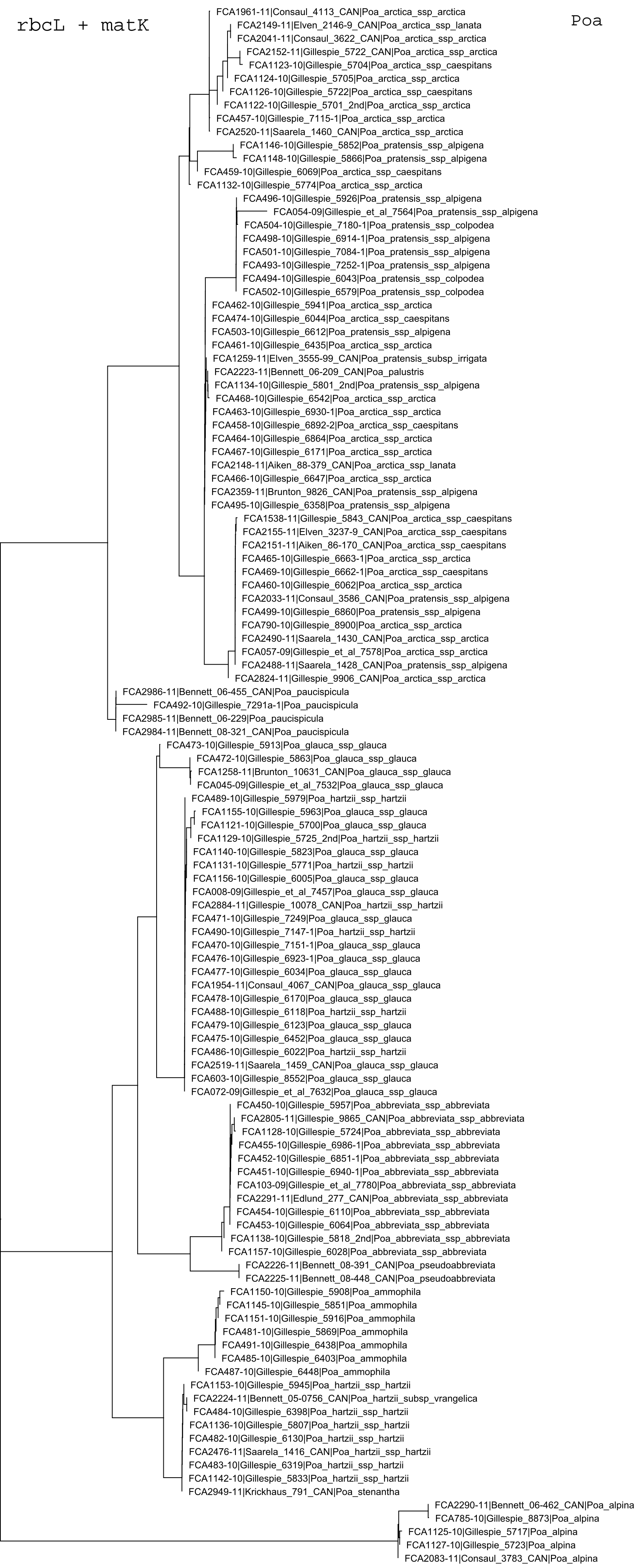

0.0020

Supplement: Figure S53 — Neighbour joining analysis of uncorrected p-distances of combined rbcL + matK sequence data for Poa (Poaceae). (PDF) [file pone.0077982.s058.pdf]

rbcL + matK

Pedicularis

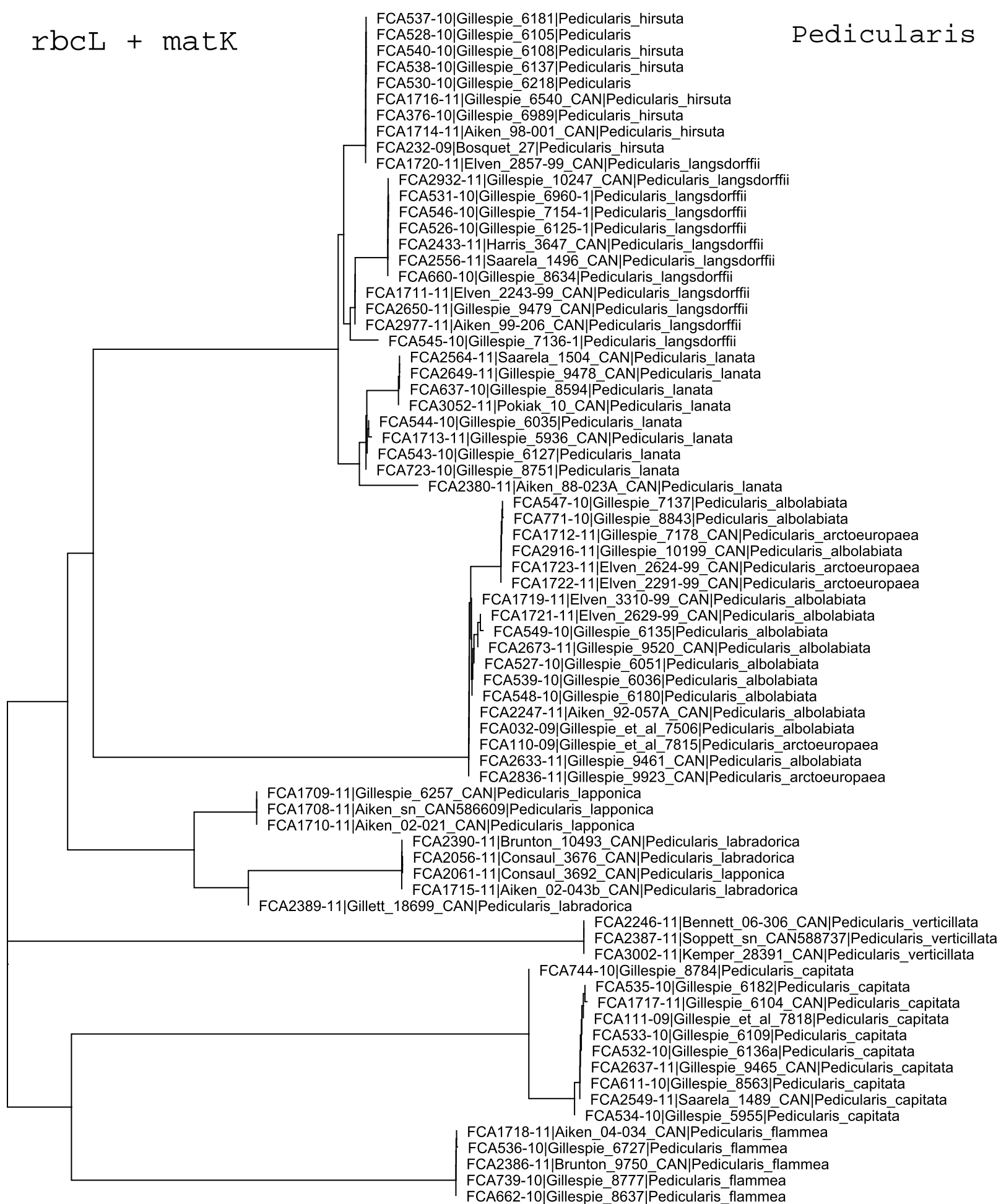

0.0030

Supplement: Figure S54 — Neighbour joining analysis of uncorrected p-distances of combined rbcL + matK sequence data for Pedicularis (Orobanchaceae). (PDF) [file pone.0077982.s059.pdf]

Salix  
rbcL + matK

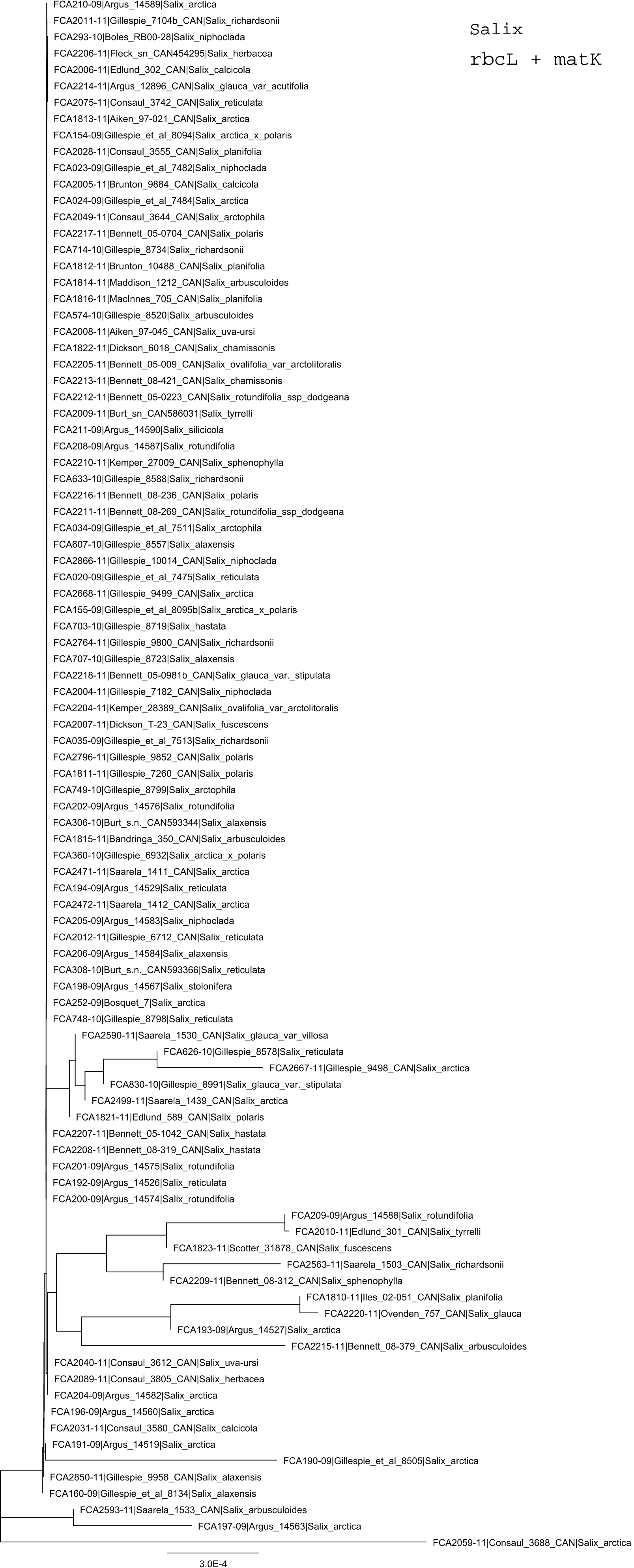

Supplement: Figure S55 — Neighbour joining analysis of uncorrected p-distances of combined rbcL + matK sequence data for Salix (Salicaceae). (PDF) [file pone.0077982.s060.pdf]

Draba  
rbcl + matK

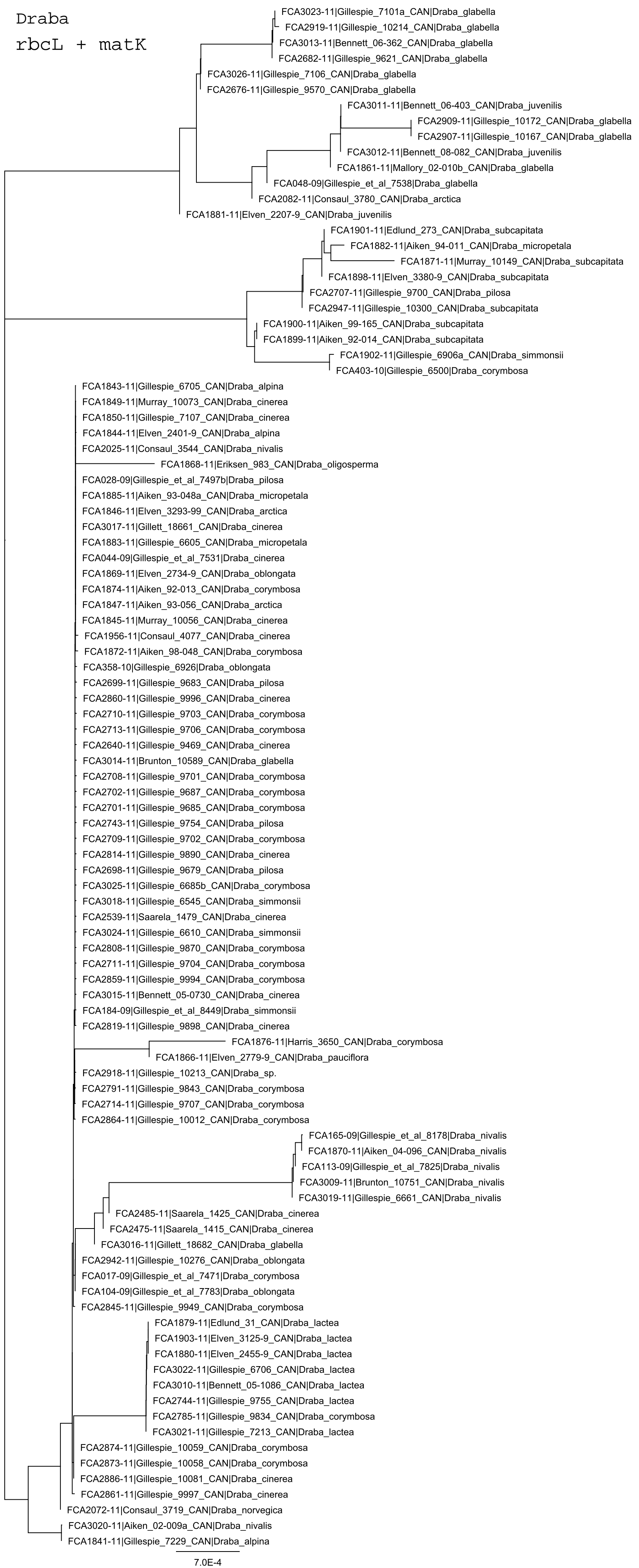

Supplement: Figure S56 — Neighbour joining analysis of uncorrected p-distances of combined rbcL + matK sequence data for Draba (Brassicaceae). (PDF) [file pone.0077982.s061.pdf]

Saxifraga

rbcL + matK

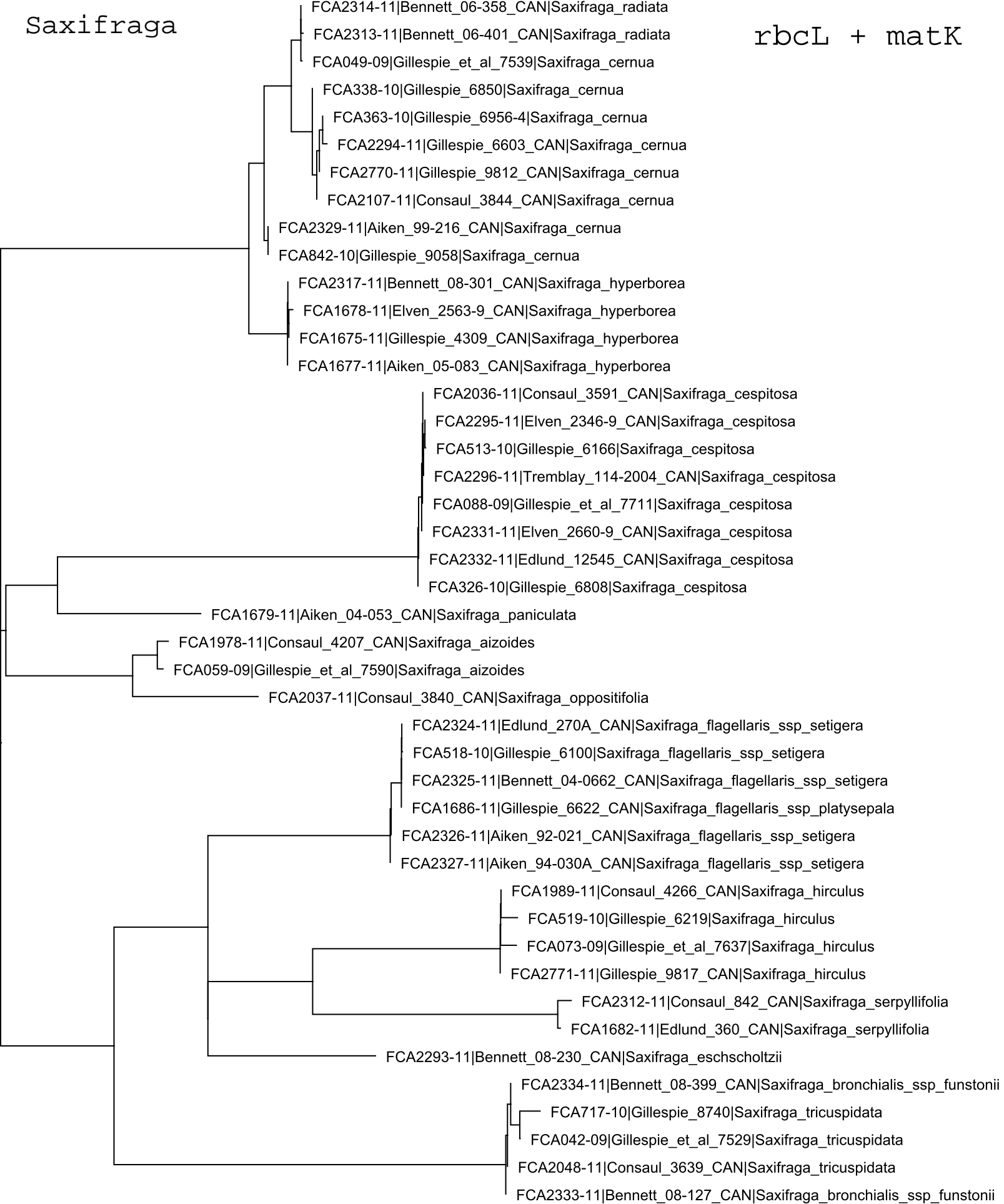

0.0050

Supplement: Figure S57 — Neighbour joining analysis of uncorrected p-distances of combined rbcL + matK sequence data for Saxifraga (Saxifragaceae). (PDF) [file pone.0077982.s062.pdf]

Ranunculus  
rbcL + matK

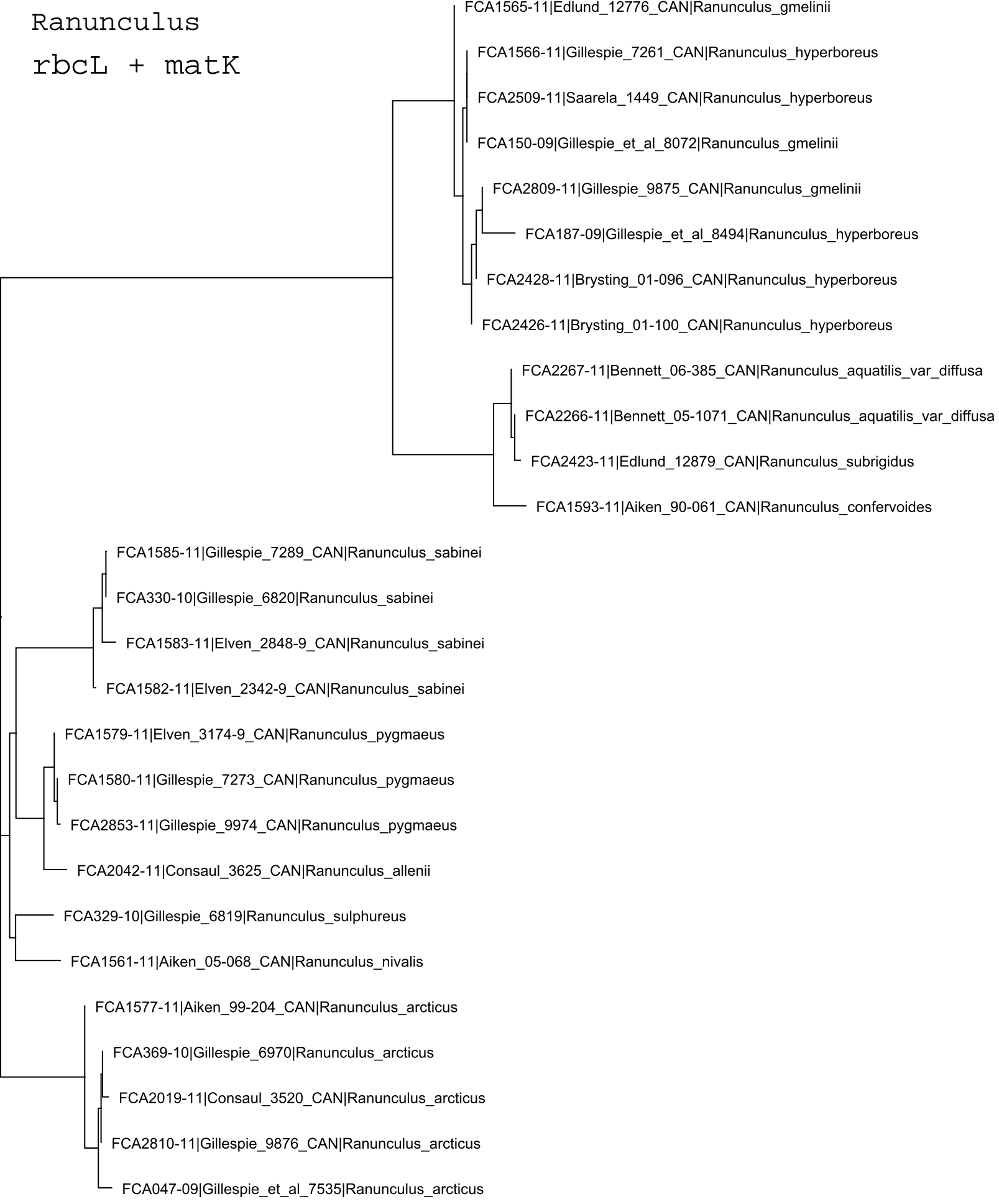

0.0050

Supplement: Figure S58 — Neighbour joining analysis of uncorrected p-distances of combined rbcL + matK sequence data for Ranunculus (Ranunculaceae). (PDF) [file pone.0077982.s063.pdf]

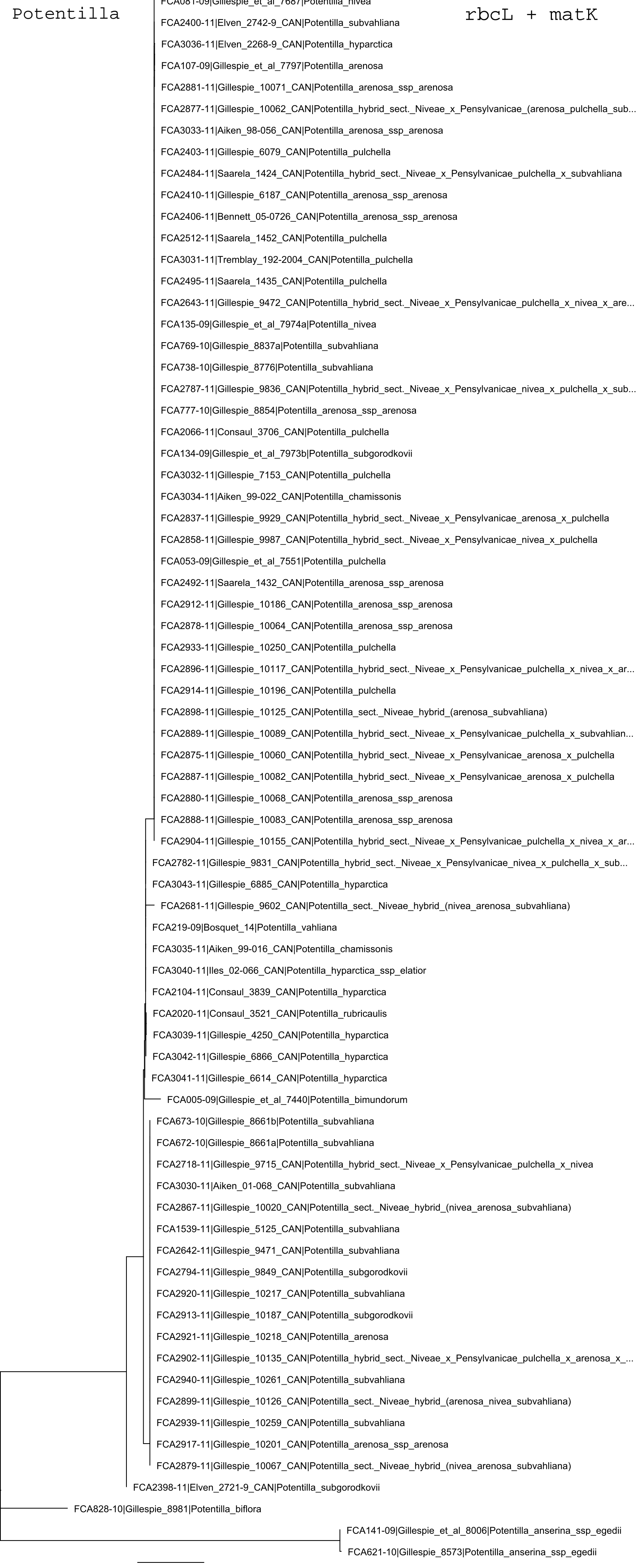

Supplement: Figure S59 — Neighbour joining analysis of uncorrected p-distances of combined rbcL + matK sequence data for Potentilla (Rosaceae). (PDF) [file pone.0077982.s064.pdf]

Carex

rbcL + matK

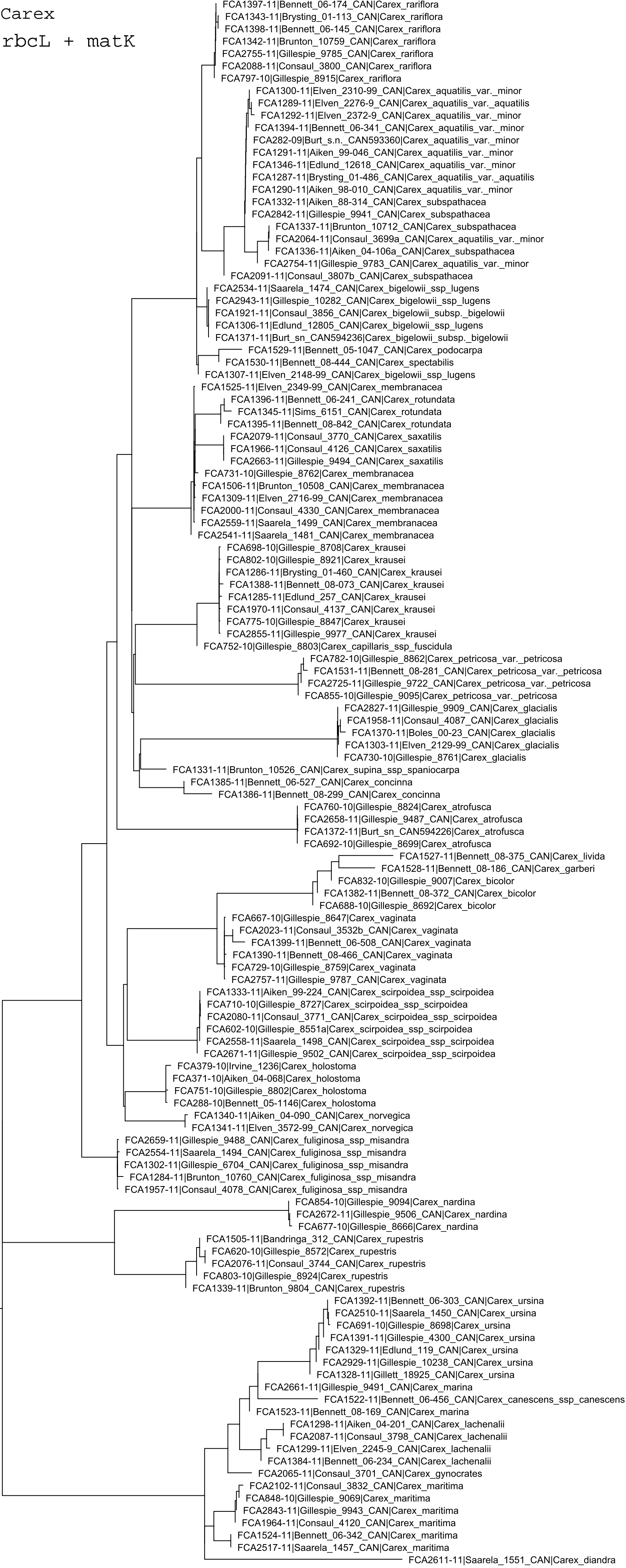

0.0030

Supplement: Figure S60 — Neighbour joining analysis of uncorrected p-distances of combined rbcL + matK sequence data for Carex (Cyperaceae). (PDF) [file pone.0077982.s065.pdf]

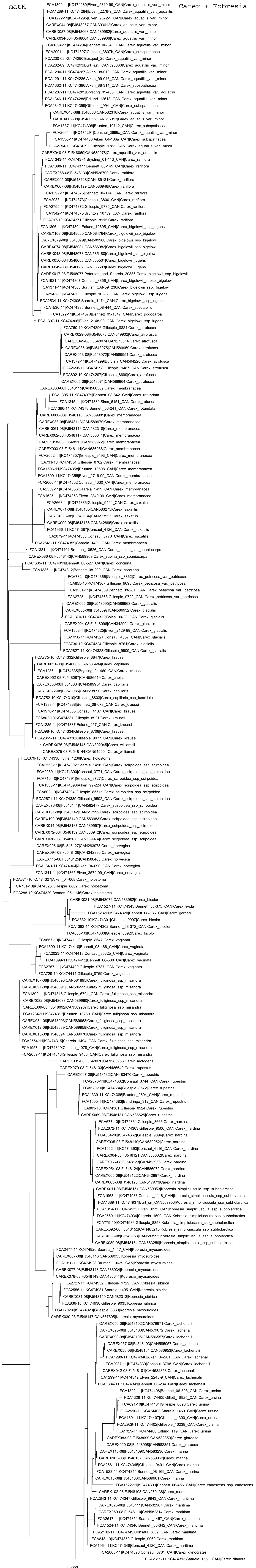

Supplement: Figure S61 — Neighbour joining analysis of uncorrected p-distances of matK sequence data for Carex and Kobresia (Cyperaceae). The tree combines data from the current study and previously published data from Le Clerc-Blain et al. [108], the latter prefaced with the project code "CAREX". . (PDF) [file pone.0077982.s066.pdf]
