# Supplementary material for: DNA Barcoding the Canadian Arctic Flora: Core Plastid Barcodes (rbcL + matK) for 490 Vascular Plant Species
Source: PLoS One. 2013 Oct 22;8(10):e77982. doi: 10.1371/journal.pone.0077982 (PMC3865322; doi:10.1371/journal.pone.0077982)

Apiaceae

(A) rbcL

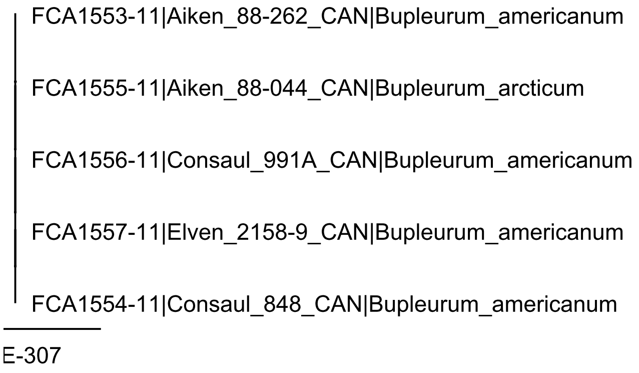

(B) matK

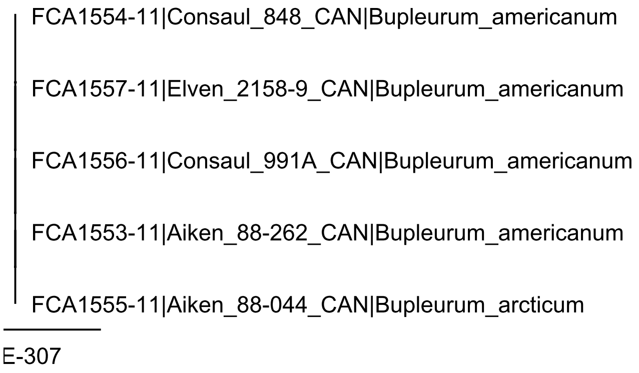

(C) rbcL + matK

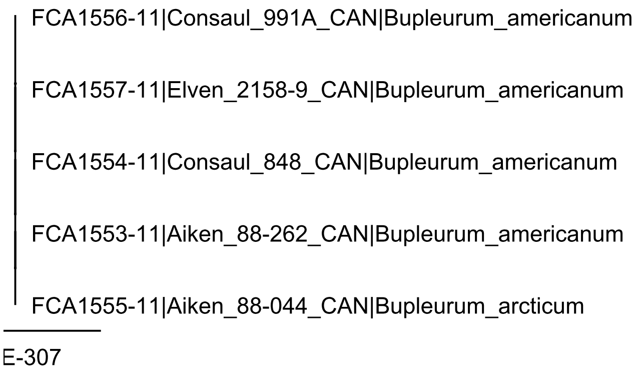

Supplement: Figure S4 — Neighbour joining analyses of uncorrected p-distances of rbcL and matK sequence data for Apiaceae. A. rbcL. B. matK. C. rbcL + matK. (PDF) [file pone.0077982.s009.pdf]
