# Supplementary material for: DNA Barcoding the Canadian Arctic Flora: Core Plastid Barcodes (rbcL + matK) for 490 Vascular Plant Species
Source: PLoS One. 2013 Oct 22;8(10):e77982. doi: 10.1371/journal.pone.0077982 (PMC3865322; doi:10.1371/journal.pone.0077982)
Supplement: Figure S7 — Neighbour joining analysis of uncorrected p-distances of matK sequence data for Boraginaceae. (PDF) [file pone.0077982.s012.pdf]

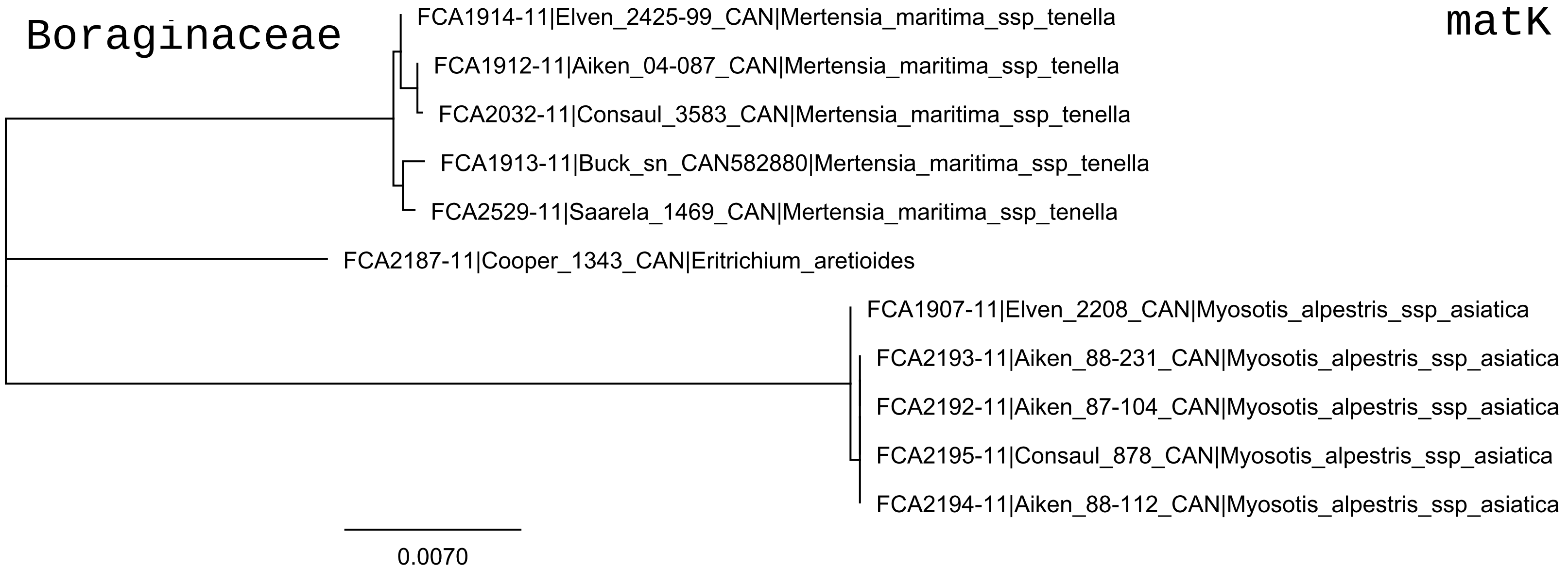
Boraginaceae
matK
FCA1914-11|Elven_2425-99_CAN|Mertensia_maritima_ssp_tenella
FCA1912-11|Aiken_04-087_CAN|Mertensia_maritima_ssp_tenella
FCA2032-11|Consaul_3583_CAN|Mertensia_maritima_ssp_tenella
FCA1913-11|Buck_sn_CAN582880|Mertensia_maritima_ssp_tenella
FCA2529-11|Saarela_1469_CAN|Mertensia_maritima_ssp_tenella
FCA2187-11|Cooper_1343_CAN|Eritrichium_aretioides
FCA1907-11|Elven_2208_CAN|Myosotis_alpestris_ssp_asiatica
FCA2193-11|Aiken_88-231_CAN|Myosotis_alpestris_ssp_asiatica
FCA2192-11|Aiken_87-104_CAN|Myosotis_alpestris_ssp_asiatica
FCA2195-11|Consaul_878_CAN|Myosotis_alpestris_ssp_asiatica
FCA2194-11|Aiken_88-112_CAN|Myosotis_alpestris_ssp_asiatica
0.0070
