# Supplementary material for: DNA Barcoding the Canadian Arctic Flora: Core Plastid Barcodes (rbcL + matK) for 490 Vascular Plant Species
Source: PLoS One. 2013 Oct 22;8(10):e77982. doi: 10.1371/journal.pone.0077982 (PMC3865322; doi:10.1371/journal.pone.0077982)
Supplement: Figure S13 — Neighbour joining analyses of uncorrected p-distances of rbcL and matK sequence data for Cyperaceae. A. rbcL. B. matK. C. rbcL + matK. (PDF) [file pone.0077982.s018.pdf]

Cyperaceae (A)rbcL

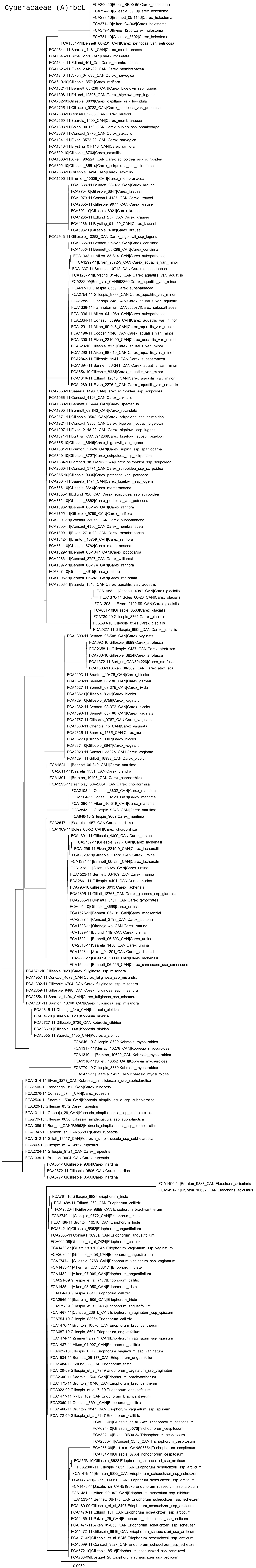

Cyperaceae (B)matK

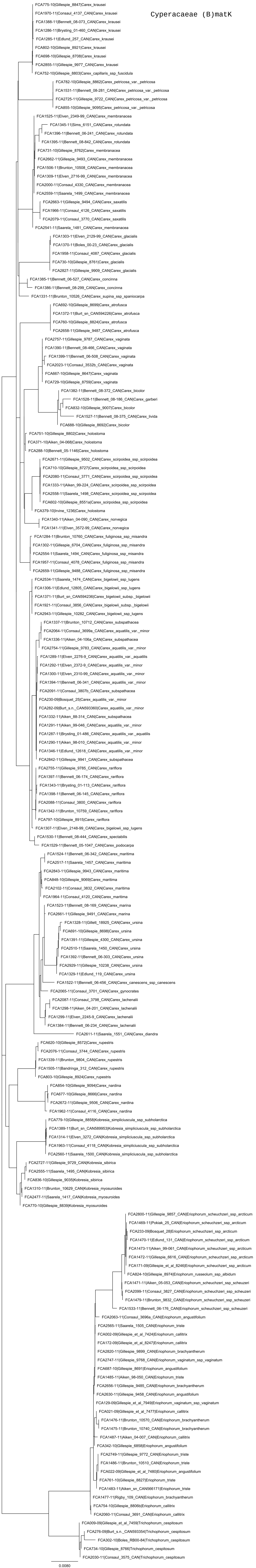

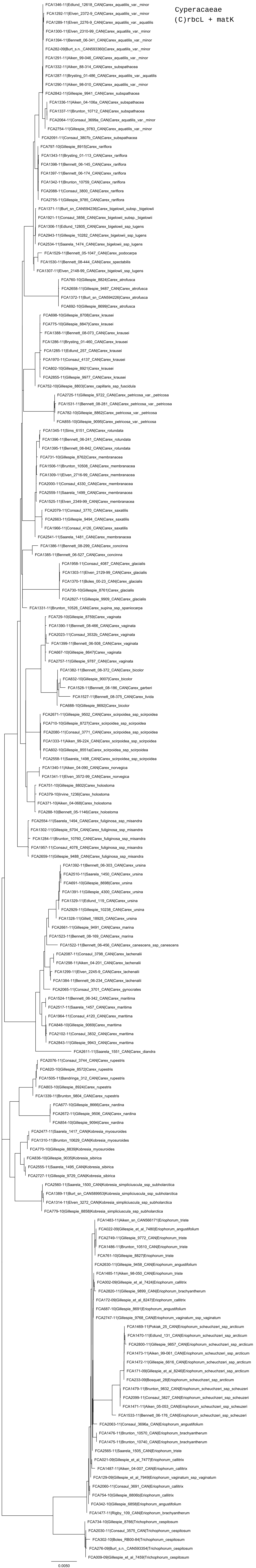

Cyperaceae
(C)rbcL + matK
FCA1346-11|Edlund_12618_CAN|Carex_aquatilis_var._minor
FCA1292-11|Elven_2372-9_CAN|Carex_aquatilis_var._minor
FCA1289-11|Elven_2276-9_CAN|Carex_aquatilis_var._aquatilis
FCA1300-11|Elven_2310-99_CAN|Carex_aquatilis_var._minor
FCA1394-11|Bennett_06-341_CAN|Carex_aquatilis_var._minor
FCA282-09|Burt_s.n._CAN593360|Carex_aquatilis_var._minor
FCA1291-11|Aiken_99-046_CAN|Carex_aquatilis_var._minor
FCA1332-11|Aiken_88-314_CAN|Carex_subspathacea
FCA1287-11|Brysting_01-486_CAN|Carex_aquatilis_var._aquatilis
FCA1290-11|Aiken_98-010_CAN|Carex_aquatilis_var._minor
FCA2842-11|Gillespie_9941_CAN|Carex_subspathacea
FCA1336-11|Aiken_04-106a_CAN|Carex_subspathacea
FCA1337-11|Brunton_10712_CAN|Carex_subspathacea
FCA2064-11|Consaul_3699a_CAN|Carex_aquatilis_var._minor
FCA2754-11|Gillespie_9783_CAN|Carex_aquatilis_var._minor
FCA2091-11|Consaul_3807b_CAN|Carex_subspathacea
FCA797-10|Gillespie_8915|Carex_rariflora
FCA1343-11|Brysting_01-113_CAN|Carex_rariflora
FCA1398-11|Bennett_06-145_CAN|Carex_rariflora
FCA1397-11|Bennett_06-174_CAN|Carex_rariflora
FCA1342-11|Brunton_10759_CAN|Carex_rariflora
FCA2088-11|Consaul_3800_CAN|Carex_rariflora
FCA2755-11|Gillespie_9785_CAN|Carex_rariflora
FCA1371-11|Burt_sn_CAN594236|Carex_bigelowii_subsp._bigelowii
FCA1921-11|Consaul_3856_CAN|Carex_bigelowii_subsp._bigelowii
FCA1306-11|Edlund_12805_CAN|Carex_bigelowii_ssp_lugens
FCA2943-11|Gillespie_10282_CAN|Carex_bigelowii_ssp_lugens
FCA2534-11|Saarela_1474_CAN|Carex_bigelowii_ssp_lugens
FCA1529-11|Bennett_05-1047_CAN|Carex_podocarpa
FCA1530-11|Bennett_08-444_CAN|Carex_spectabilis
FCA1307-11|Elven_2148-99_CAN|Carex_bigelowii_ssp_lugens
FCA760-10|Gillespie_8824|Carex_atrofusca
FCA2658-11|Gillespie_9487_CAN|Carex_atrofusca
FCA1372-11|Burt_sn_CAN594226|Carex_atrofusca
FCA692-10|Gillespie_8699|Carex_atrofusca
FCA698-10|Gillespie_8708|Carex_krausei
FCA775-10|Gillespie_8847|Carex_krausei
FCA1388-11|Bennett_08-073_CAN|Carex_krausei
FCA1286-11|Brysting_01-460_CAN|Carex_krausei
FCA1285-11|Edlund_257_CAN|Carex_krausei
FCA1970-11|Consaul_4137_CAN|Carex_krausei
FCA802-10|Gillespie_8921|Carex_krausei
FCA2855-11|Gillespie_9977_CAN|Carex_krausei
FCA752-10|Gillespie_8803|Carex_capillaris_ssp_fuscidula
FCA2725-11|Gillespie_9722_CAN|Carex_petricosa_var._petricosa
FCA1531-11|Bennett_08-281_CAN|Carex_petricosa_var._petricosa
FCA782-10|Gillespie_8862|Carex_petricosa_var._petricosa
FCA855-10|Gillespie_9095|Carex_petricosa_var._petricosa
FCA1345-11|Sims_6151_CAN|Carex_rotundata
FCA1396-11|Bennett_06-241_CAN|Carex_rotundata
FCA1395-11|Bennett_08-842_CAN|Carex_rotundata
FCA731-10|Gillespie_8762|Carex_membranacea
FCA1506-11|Brunton_10508_CAN|Carex_membranacea
FCA1309-11|Elven_2716-99_CAN|Carex_membranacea
FCA2000-11|Consaul_4330_CAN|Carex_membranacea
FCA2559-11|Saarela_1499_CAN|Carex_membranacea
FCA1525-11|Elven_2349-99_CAN|Carex_membranacea
FCA2079-11|Consaul_3770_CAN|Carex_saxatilis
FCA2663-11|Gillespie_9494_CAN|Carex_saxatilis
FCA1966-11|Consaul_4126_CAN|Carex_saxatilis
FCA2541-11|Saarela_1481_CAN|Carex_membranacea
FCA1386-11|Bennett_08-299_CAN|Carex_concinna
FCA1385-11|Bennett_06-527_CAN|Carex_concinna
FCA1958-11|Consaul_4087_CAN|Carex_glacialis
FCA1303-11|Elven_2129-99_CAN|Carex_glacialis
FCA1370-11|Boles_00-23_CAN|Carex_glacialis
FCA730-10|Gillespie_8761|Carex_glacialis
FCA2827-11|Gillespie_9909_CAN|Carex_glacialis
FCA1331-11|Brunton_10526_CAN|Carex_supina_ssp_spaniocarpa
FCA729-10|Gillespie_8759|Carex_vaginata
FCA1390-11|Bennett_08-466_CAN|Carex_vaginata
FCA2023-11|Consaul_3532b_CAN|Carex_vaginata
FCA1399-11|Bennett_06-508_CAN|Carex_vaginata
FCA667-10|Gillespie_8647|Carex_vaginata
FCA2757-11|Gillespie_9787_CAN|Carex_vaginata
FCA1382-11|Bennett_08-372_CAN|Carex_bicolor
FCA832-10|Gillespie_9007|Carex_bicolor
FCA1528-11|Bennett_08-186_CAN|Carex_garberi
FCA1527-11|Bennett_08-375_CAN|Carex_livida
FCA688-10|Gillespie_8692|Carex_bicolor
FCA2671-11|Gillespie_9502_CAN|Carex_scirpoidea_ssp_scirpoidea
FCA710-10|Gillespie_8727|Carex_scirpoidea_ssp_scirpoidea
FCA2080-11|Consaul_3771_CAN|Carex_scirpoidea_ssp_scirpoidea
FCA1333-11|Aiken_99-224_CAN|Carex_scirpoidea_ssp_scirpoidea
FCA602-10|Gillespie_8551a|Carex_scirpoidea_ssp_scirpoidea
FCA2558-11|Saarela_1498_CAN|Carex_scirpoidea_ssp_scirpoidea
FCA1340-11|Aiken_04-090_CAN|Carex_norvegica
FCA1341-11|Elven_3572-99_CAN|Carex_norvegica
FCA751-10|Gillespie_8802|Carex_holostoma
FCA379-10|Irvine_1236|Carex_holostoma
FCA371-10|Aiken_04-068|Carex_holostoma
FCA288-10|Bennett_05-1146|Carex_holostoma
FCA2554-11|Saarela_1494_CAN|Carex_fuliginosa_ssp_misandra
FCA1302-11|Gillespie_6704_CAN|Carex_fuliginosa_ssp_misandra
FCA1284-11|Brunton_10760_CAN|Carex_fuliginosa_ssp_misandra
FCA1957-11|Consaul_4078_CAN|Carex_fuliginosa_ssp_misandra
FCA2659-11|Gillespie_9488_CAN|Carex_fuliginosa_ssp_misandra
FCA1392-11|Bennett_06-303_CAN|Carex_ursina
FCA2510-11|Saarela_1450_CAN|Carex_ursina
FCA691-10|Gillespie_8698|Carex_ursina
FCA1391-11|Gillespie_4300_CAN|Carex_ursina
FCA1329-11|Edlund_119_CAN|Carex_ursina
FCA2929-11|Gillespie_10238_CAN|Carex_ursina
FCA1328-11|Gillett_18925_CAN|Carex_ursina
FCA2661-11|Gillespie_9491_CAN|Carex_marina
FCA1523-11|Bennett_08-169_CAN|Carex_marina
FCA1522-11|Bennett_06-456_CAN|Carex_canescens_ssp_canescens
FCA2087-11|Consaul_3798_CAN|Carex_lachenalii
FCA1298-11|Aiken_04-201_CAN|Carex_lachenalii
FCA1299-11|Elven_2245-9_CAN|Carex_lachenalii
FCA1384-11|Bennett_06-234_CAN|Carex_lachenalii
FCA2065-11|Consaul_3701_CAN|Carex_gynocrates
FCA1524-11|Bennett_06-342_CAN|Carex_maritima
FCA2517-11|Saarela_1457_CAN|Carex_maritima
FCA1964-11|Consaul_4120_CAN|Carex_maritima
FCA848-10|Gillespie_9069|Carex_maritima
FCA2102-11|Consaul_3832_CAN|Carex_maritima
FCA2843-11|Gillespie_9943_CAN|Carex_maritima
FCA2611-11|Saarela_1551_CAN|Carex_diandra
FCA2076-11|Consaul_3744_CAN|Carex_rupestris
FCA620-10|Gillespie_8572|Carex_rupestris
FCA1505-11|Bandringa_312_CAN|Carex_rupestris
FCA803-10|Gillespie_8924|Carex_rupestris
FCA1339-11|Brunton_9804_CAN|Carex_rupestris
FCA677-10|Gillespie_8666|Carex_nardina
FCA2672-11|Gillespie_9506_CAN|Carex_nardina
FCA854-10|Gillespie_9094|Carex_nardina
FCA2477-11|Saarela_1417_CAN|Kobresia_myosuroides
FCA1310-11|Brunton_10629_CAN|Kobresia_myosuroides
FCA770-10|Gillespie_8839|Kobresia_myosuroides
FCA836-10|Gillespie_9035|Kobresia_sibirica
FCA2555-11|Saarela_1495_CAN|Kobresia_sibirica
FCA2727-11|Gillespie_9729_CAN|Kobresia_sibirica
FCA2560-11|Saarela_1500_CAN|Kobresia_simpliciuscula_ssp_subholarctica
FCA1389-11|Burt_sn_CAN589953|Kobresia_simpliciuscula_ssp_subholarctica
FCA1314-11|Elven_3272_CAN|Kobresia_simpliciuscula_ssp_subholarctica
FCA779-10|Gillespie_8858|Kobresia_simpliciuscula_ssp_subholarctica
FCA1483-11|Aiken_sn_CAN566171|Eriophorum_triste
FCA022-09|Gillespie_et_al_7480|Eriophorum_angustifolium
FCA2749-11|Gillespie_9772_CAN|Eriophorum_triste
FCA1486-11|Brunton_10510_CAN|Eriophorum_triste
FCA761-10|Gillespie_8827|Eriophorum_triste
FCA2630-11|Gillespie_9458_CAN|Eriophorum_angustifolium
FCA1485-11|Aiken_98-050_CAN|Eriophorum_triste
FCA002-09|Gillespie_et_al_7424|Eriophorum_callitrix
FCA2820-11|Gillespie_9899_CAN|Eriophorum_brachyantherum
FCA172-09|Gillespie_et_al_8247|Eriophorum_callitrix
FCA687-10|Gillespie_8691|Eriophorum_angustifolium
FCA2747-11|Gillespie_9768_CAN|Eriophorum_vaginatum_ssp_vaginatum
FCA1469-11|Pokiak_25_CAN|Eriophorum_scheuchzeri_ssp_arcticum
FCA1470-11|Edlund_131_CAN|Eriophorum_scheuchzeri_ssp_arcticum
FCA2800-11|Gillespie_9857_CAN|Eriophorum_scheuchzeri_ssp_arcticum
FCA1473-11|Aiken_99-061_CAN|Eriophorum_scheuchzeri_ssp_arcticum
FCA1472-11|Gillespie_6616_CAN|Eriophorum_scheuchzeri_ssp_arcticum
FCA171-09|Gillespie_et_al_8246|Eriophorum_scheuchzeri_ssp_arcticum
FCA233-09|Bosquet_28|Eriophorum_scheuchzeri_ssp_arcticum
FCA1479-11|Brunton_9832_CAN|Eriophorum_scheuchzeri_ssp_scheuzeri
FCA2099-11|Consaul_3827_CAN|Eriophorum_scheuchzeri_ssp_scheuzeri
FCA1471-11|Aiken_05-053_CAN|Eriophorum_scheuchzeri_ssp_scheuzeri
FCA1533-11|Bennett_06-176_CAN|Eriophorum_scheuchzeri_ssp_scheuzeri
FCA2063-11|Consaul_3696a_CAN|Eriophorum_angustifolium
FCA1476-11|Brunton_10570_CAN|Eriophorum_brachyantherum
FCA1475-11|Brunton_10740_CAN|Eriophorum_brachyantherum
FCA2565-11|Saarela_1505_CAN|Eriophorum_triste
FCA021-09|Gillespie_et_al_7477|Eriophorum_callitrix
FCA1487-11|Aiken_04-007_CAN|Eriophorum_callitrix
FCA129-09|Gillespie_et_al_7949|Eriophorum_vaginatum_ssp_vaginatum
FCA2060-11|Consaul_3691_CAN|Eriophorum_callitrix
FCA754-10|Gillespie_8806b|Eriophorum_callitrix
FCA342-10|Gillespie_6858|Eriophorum_angustifolium
FCA1477-11|Rigby_109_CAN|Eriophorum_brachyantherum
FCA734-10|Gillespie_8766|Trichophorum_cespitosum
FCA2030-11|Consaul_3575_CAN|Trichophorum_cespitosum
FCA302-10|Boles_RB00-84|Trichophorum_cespitosum
FCA276-09|Burt_s.n._CAN593354|Trichophorum_cespitosum
FCA009-09|Gillespie_et_al_7459|Trichophorum_cespitosum
0.0050
