# Supplementary material for: DNA Barcoding the Canadian Arctic Flora: Core Plastid Barcodes (rbcL + matK) for 490 Vascular Plant Species
Source: PLoS One. 2013 Oct 22;8(10):e77982. doi: 10.1371/journal.pone.0077982 (PMC3865322; doi:10.1371/journal.pone.0077982)

Linaceae rbcL

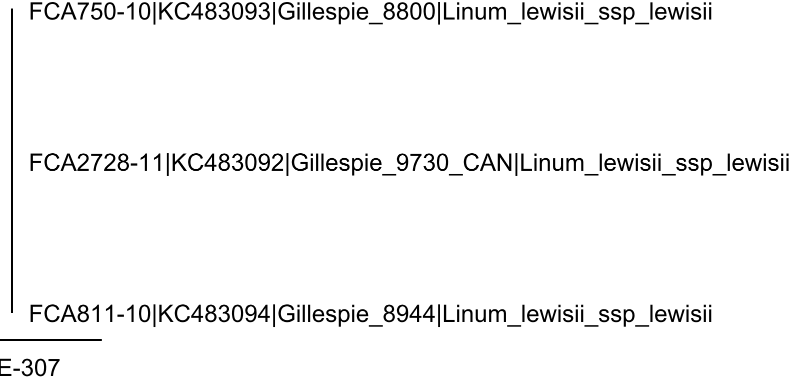

Supplement: Figure S25 — Neighbour joining analyses of uncorrected p-distances of rbcL sequence data for Linaceae. (PDF) [file pone.0077982.s030.pdf]
