# Supplementary material for: DNA Barcoding the Canadian Arctic Flora: Core Plastid Barcodes (rbcL + matK) for 490 Vascular Plant Species
Source: PLoS One. 2013 Oct 22;8(10):e77982. doi: 10.1371/journal.pone.0077982 (PMC3865322; doi:10.1371/journal.pone.0077982)
Supplement: Figure S26 — Neighbour joining analysis of uncorrected p-distances of rbcL sequence data for Lycopodiaceae. (PDF) [file pone.0077982.s031.pdf]

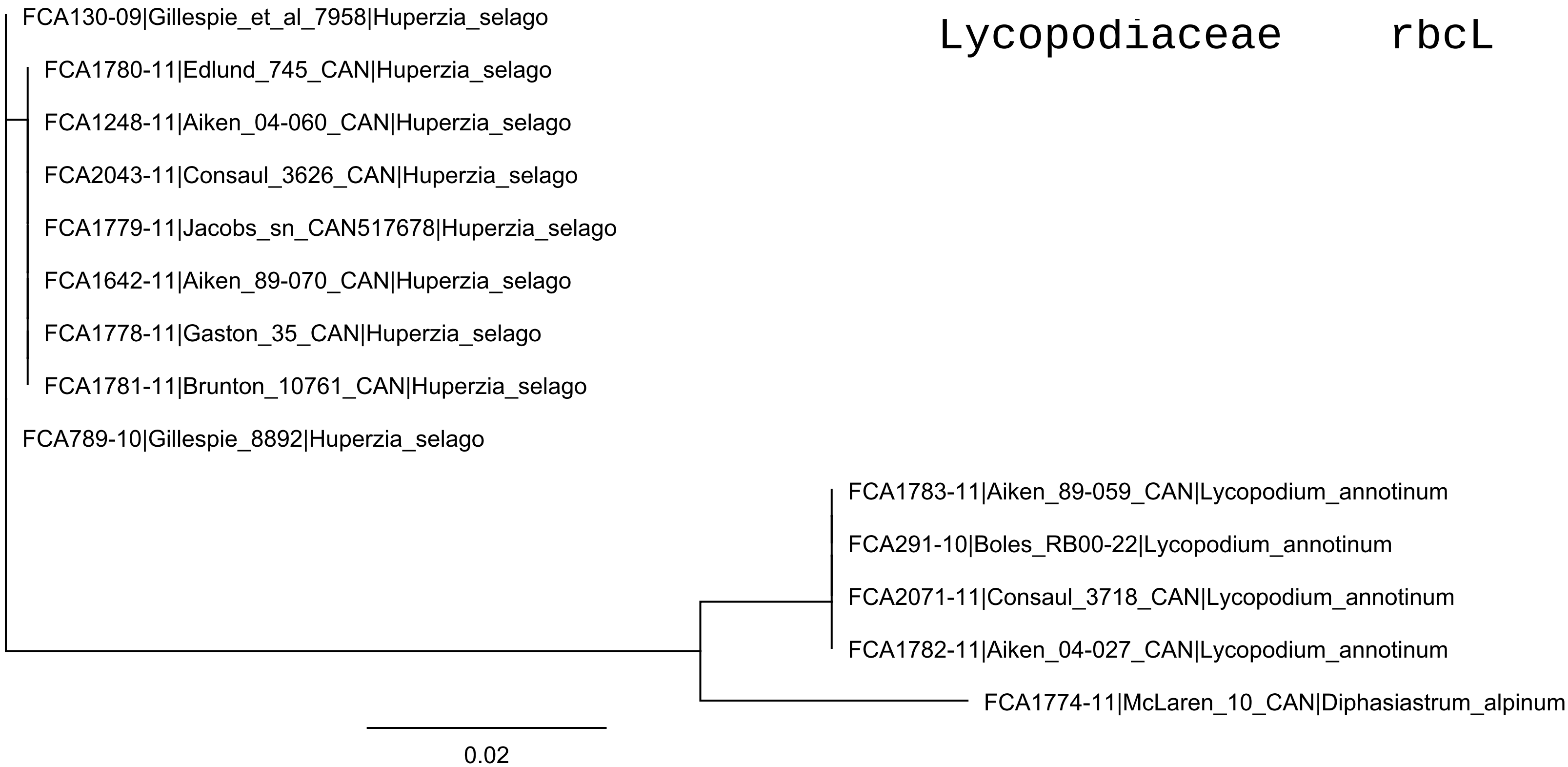
Lycopodiaceae rbcL
FCA130-09|Gillespie_et_al_7958|Huperzia_selago
FCA1780-11|Edlund_745_CAN|Huperzia_selago
FCA1248-11|Aiken_04-060_CAN|Huperzia_selago
FCA2043-11|Consaul_3626_CAN|Huperzia_selago
FCA1779-11|Jacobs_sn_CAN517678|Huperzia_selago
FCA1642-11|Aiken_89-070_CAN|Huperzia_selago
FCA1778-11|Gaston_35_CAN|Huperzia_selago
FCA1781-11|Brunton_10761_CAN|Huperzia_selago
FCA789-10|Gillespie_8892|Huperzia_selago
FCA1783-11|Aiken_89-059_CAN|Lycopodium_annotinum
FCA291-10|Boles_RB00-22|Lycopodium_annotinum
FCA2071-11|Consaul_3718_CAN|Lycopodium_annotinum
FCA1782-11|Aiken_04-027_CAN|Lycopodium_annotinum
FCA1774-11|McLaren_10_CAN|Diphasiastrum_alpinum
0.02
