# Supplementary material for: DNA Barcoding the Canadian Arctic Flora: Core Plastid Barcodes (rbcL + matK) for 490 Vascular Plant Species
Source: PLoS One. 2013 Oct 22;8(10):e77982. doi: 10.1371/journal.pone.0077982 (PMC3865322; doi:10.1371/journal.pone.0077982)
Supplement: Figure S33 — Neighbour joining analyses of uncorrected p-distances of rbcL and matK sequence data for Papaveraceae. A. rbcL. B. matK. C. rbcL + matK. (PDF) [file pone.0077982.s038.pdf]

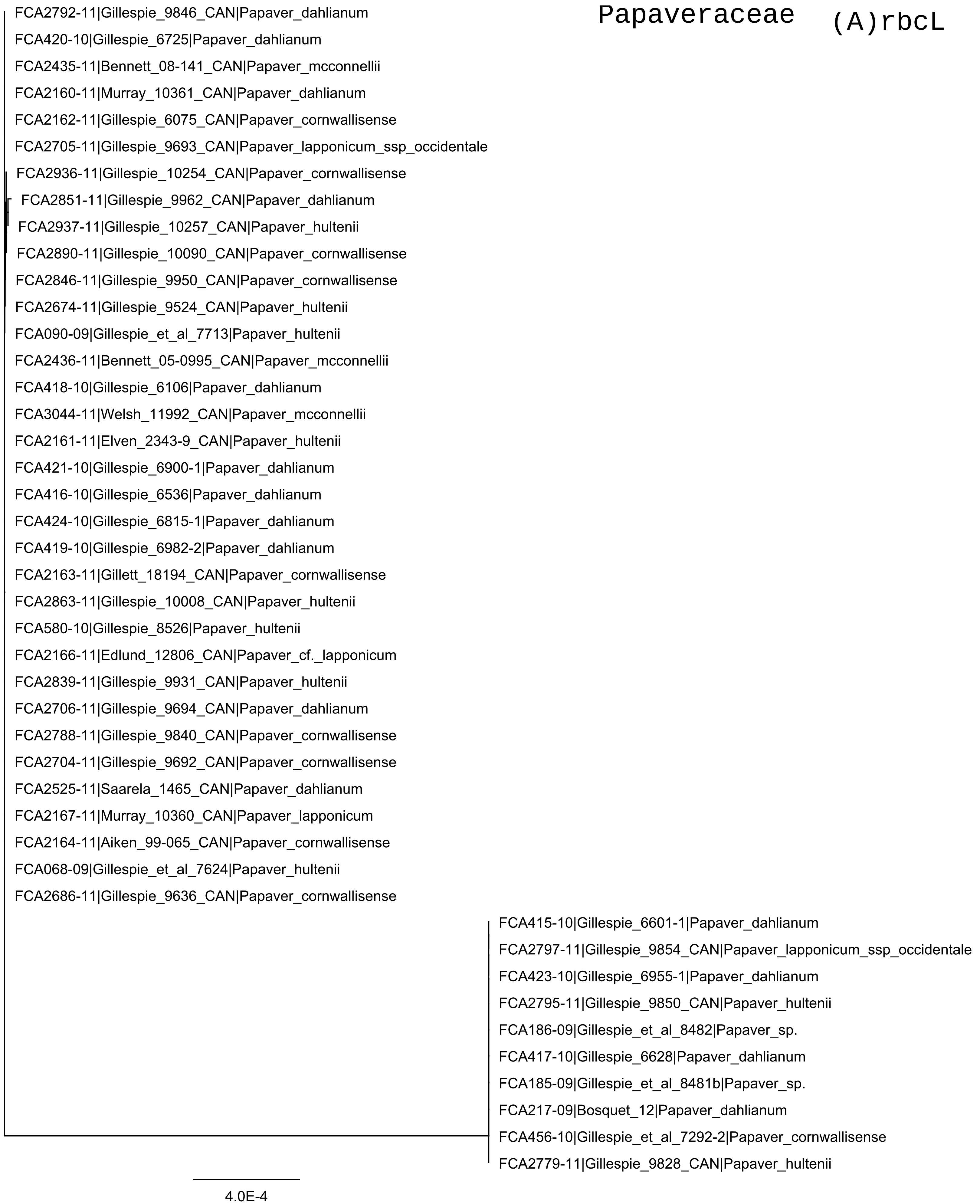
Papaveraceae
(A)rbcL
FCA2792-11|Gillespie_9846_CAN|Papaver_dahlianum
FCA420-10|Gillespie_6725|Papaver_dahlianum
FCA2435-11|Bennett_08-141_CAN|Papaver_mcconnellii
FCA2160-11|Murray_10361_CAN|Papaver_dahlianum
FCA2162-11|Gillespie_6075_CAN|Papaver_cornwallisense
FCA2705-11|Gillespie_9693_CAN|Papaver_lapponicum_ssp_occidentale
FCA2936-11|Gillespie_10254_CAN|Papaver_cornwallisense
FCA2851-11|Gillespie_9962_CAN|Papaver_dahlianum
FCA2937-11|Gillespie_10257_CAN|Papaver_hultenii
FCA2890-11|Gillespie_10090_CAN|Papaver_cornwallisense
FCA2846-11|Gillespie_9950_CAN|Papaver_cornwallisense
FCA2674-11|Gillespie_9524_CAN|Papaver_hultenii
FCA090-09|Gillespie_et_al_7713|Papaver_hultenii
FCA2436-11|Bennett_05-0995_CAN|Papaver_mcconnellii
FCA418-10|Gillespie_6106|Papaver_dahlianum
FCA3044-11|Welsh_11992_CAN|Papaver_mcconnellii
FCA2161-11|Elven_2343-9_CAN|Papaver_hultenii
FCA421-10|Gillespie_6900-1|Papaver_dahlianum
FCA416-10|Gillespie_6536|Papaver_dahlianum
FCA424-10|Gillespie_6815-1|Papaver_dahlianum
FCA419-10|Gillespie_6982-2|Papaver_dahlianum
FCA2163-11|Gillett_18194_CAN|Papaver_cornwallisense
FCA2863-11|Gillespie_10008_CAN|Papaver_hultenii
FCA580-10|Gillespie_8526|Papaver_hultenii
FCA2166-11|Edlund_12806_CAN|Papaver_cf._lapponicum
FCA2839-11|Gillespie_9931_CAN|Papaver_hultenii
FCA2706-11|Gillespie_9694_CAN|Papaver_dahlianum
FCA2788-11|Gillespie_9840_CAN|Papaver_cornwallisense
FCA2704-11|Gillespie_9692_CAN|Papaver_cornwallisense
FCA2525-11|Saarela_1465_CAN|Papaver_dahlianum
FCA2167-11|Murray_10360_CAN|Papaver_lapponicum
FCA2164-11|Aiken_99-065_CAN|Papaver_cornwallisense
FCA068-09|Gillespie_et_al_7624|Papaver_hultenii
FCA2686-11|Gillespie_9636_CAN|Papaver_cornwallisense
FCA415-10|Gillespie_6601-1|Papaver_dahlianum
FCA2797-11|Gillespie_9854_CAN|Papaver_lapponicum_ssp_occidentale
FCA423-10|Gillespie_6955-1|Papaver_dahlianum
FCA2795-11|Gillespie_9850_CAN|Papaver_hultenii
FCA186-09|Gillespie_et_al_8482|Papaver_sp.
FCA417-10|Gillespie_6628|Papaver_dahlianum
FCA185-09|Gillespie_et_al_8481b|Papaver_sp.
FCA217-09|Bosquet_12|Papaver_dahlianum
FCA456-10|Gillespie_et_al_7292-2|Papaver_cornwallisense
FCA2779-11|Gillespie_9828_CAN|Papaver_hultenii
4.0E-4

Papaveraceae (B)matK

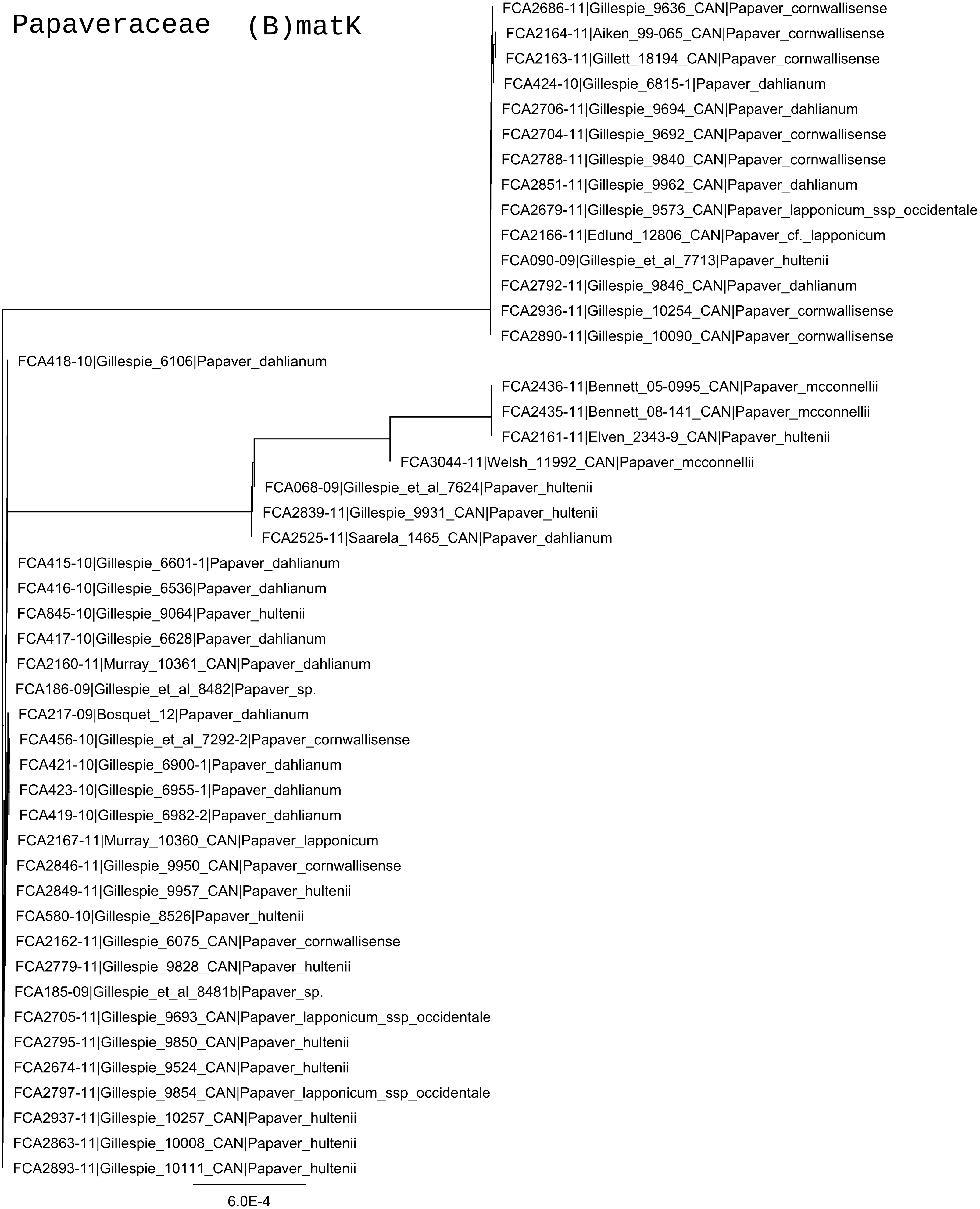

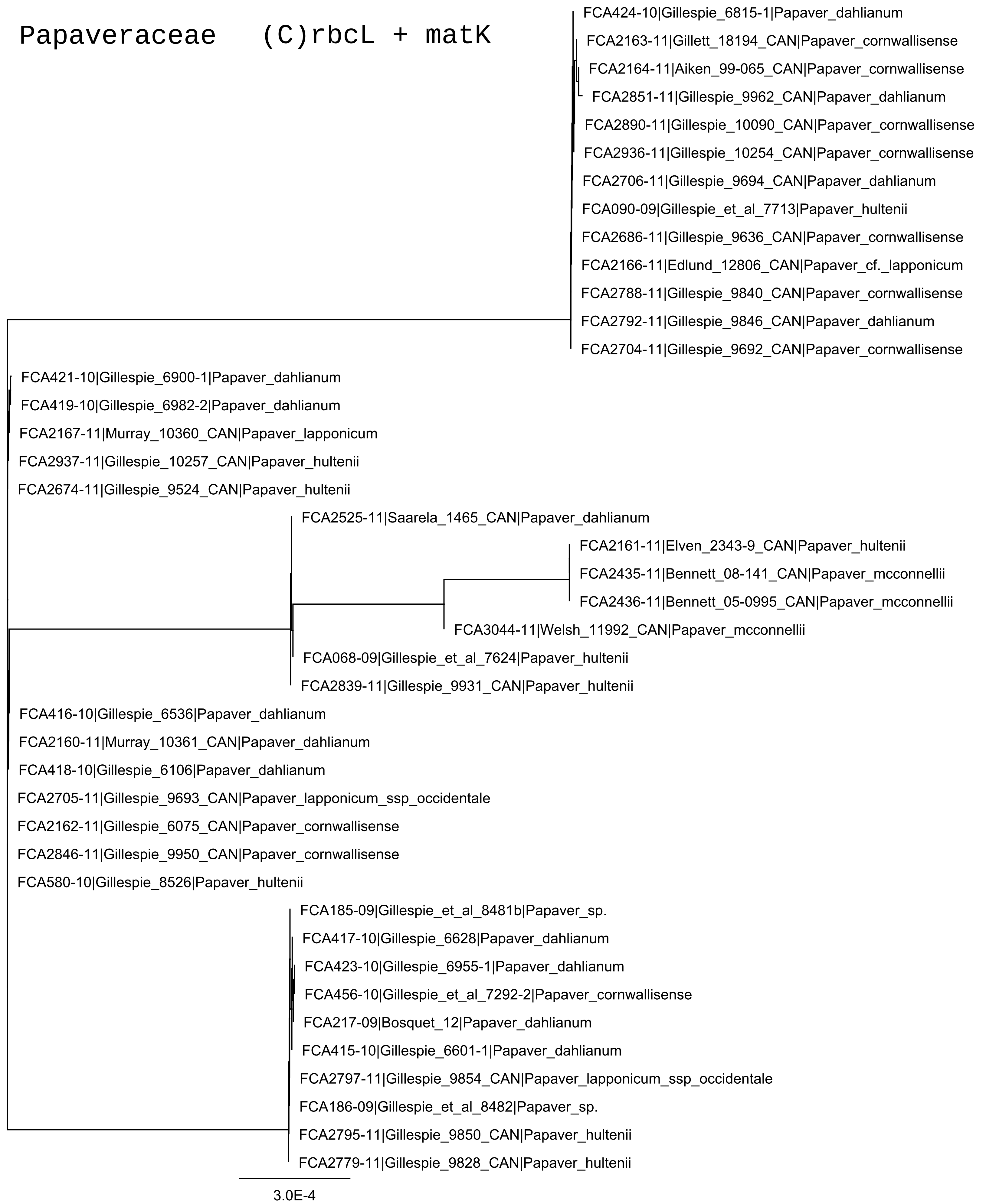

Papaveraceae (C)rbcL + matK
FCA424-10|Gillespie_6815-1|Papaver_dahlianum
FCA2163-11|Gillett_18194_CAN|Papaver_cornwallisense
FCA2164-11|Aiken_99-065_CAN|Papaver_cornwallisense
FCA2851-11|Gillespie_9962_CAN|Papaver_dahlianum
FCA2890-11|Gillespie_10090_CAN|Papaver_cornwallisense
FCA2936-11|Gillespie_10254_CAN|Papaver_cornwallisense
FCA2706-11|Gillespie_9694_CAN|Papaver_dahlianum
FCA090-09|Gillespie_et_al_7713|Papaver_hultenii
FCA2686-11|Gillespie_9636_CAN|Papaver_cornwallisense
FCA2166-11|Edlund_12806_CAN|Papaver_cf._lapponicum
FCA2788-11|Gillespie_9840_CAN|Papaver_cornwallisense
FCA2792-11|Gillespie_9846_CAN|Papaver_dahlianum
FCA2704-11|Gillespie_9692_CAN|Papaver_cornwallisense
FCA421-10|Gillespie_6900-1|Papaver_dahlianum
FCA419-10|Gillespie_6982-2|Papaver_dahlianum
FCA2167-11|Murray_10360_CAN|Papaver_lapponicum
FCA2937-11|Gillespie_10257_CAN|Papaver_hultenii
FCA2674-11|Gillespie_9524_CAN|Papaver_hultenii
FCA2525-11|Saarela_1465_CAN|Papaver_dahlianum
FCA2161-11|Elven_2343-9_CAN|Papaver_hultenii
FCA2435-11|Bennett_08-141_CAN|Papaver_mcconnellii
FCA2436-11|Bennett_05-0995_CAN|Papaver_mcconnellii
FCA3044-11|Welsh_11992_CAN|Papaver_mcconnellii
FCA068-09|Gillespie_et_al_7624|Papaver_hultenii
FCA2839-11|Gillespie_9931_CAN|Papaver_hultenii
FCA416-10|Gillespie_6536|Papaver_dahlianum
FCA2160-11|Murray_10361_CAN|Papaver_dahlianum
FCA418-10|Gillespie_6106|Papaver_dahlianum
FCA2705-11|Gillespie_9693_CAN|Papaver_lapponicum_ssp_occidentale
FCA2162-11|Gillespie_6075_CAN|Papaver_cornwallisense
FCA2846-11|Gillespie_9950_CAN|Papaver_cornwallisense
FCA580-10|Gillespie_8526|Papaver_hultenii
FCA185-09|Gillespie_et_al_8481b|Papaver_sp.
FCA417-10|Gillespie_6628|Papaver_dahlianum
FCA423-10|Gillespie_6955-1|Papaver_dahlianum
FCA456-10|Gillespie_et_al_7292-2|Papaver_cornwallisense
FCA217-09|Bosquet_12|Papaver_dahlianum
FCA415-10|Gillespie_6601-1|Papaver_dahlianum
FCA2797-11|Gillespie_9854_CAN|Papaver_lapponicum_ssp_occidentale
FCA186-09|Gillespie_et_al_8482|Papaver_sp.
FCA2795-11|Gillespie_9850_CAN|Papaver_hultenii
FCA2779-11|Gillespie_9828_CAN|Papaver_hultenii
3.0E-4
