# Supplementary material for: DNA Barcoding the Canadian Arctic Flora: Core Plastid Barcodes (rbcL + matK) for 490 Vascular Plant Species
Source: PLoS One. 2013 Oct 22;8(10):e77982. doi: 10.1371/journal.pone.0077982 (PMC3865322; doi:10.1371/journal.pone.0077982)
Supplement: Figure S42 — Neighbour joining analyses of uncorrected p-distances of rbcL and matK sequence data for Ranunculaceae. A. rbcL. B. matK. C. rbcL + matK. (PDF) [file pone.0077982.s047.pdf]

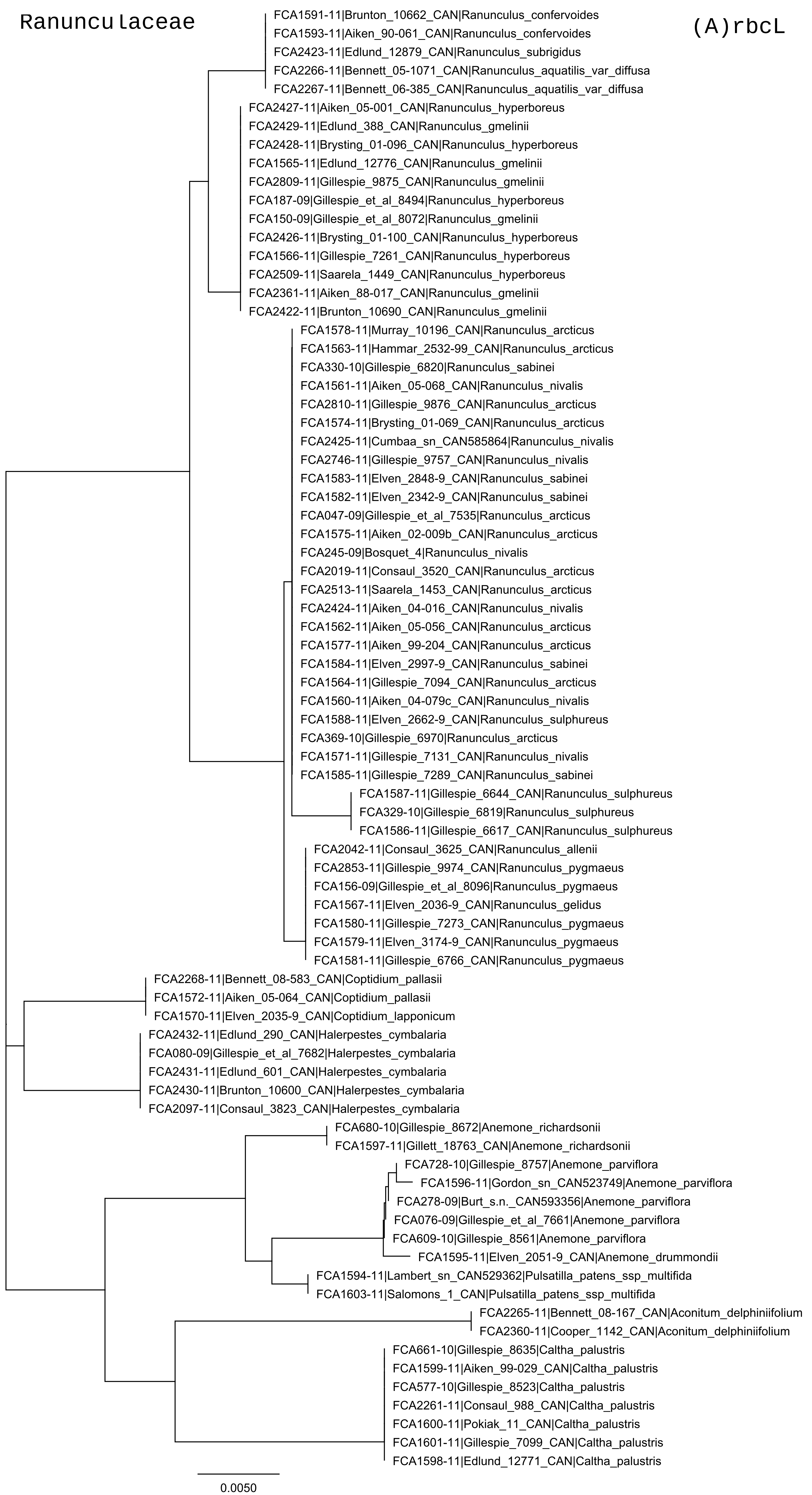
Ranunculaceae
(A) rbcL
FCA1591-11|Brunton_10662_CAN|Ranunculus_confervoides
FCA1593-11|Aiken_90-061_CAN|Ranunculus_confervoides
FCA2423-11|Edlund_12879_CAN|Ranunculus_subrigidus
FCA2266-11|Bennett_05-1071_CAN|Ranunculus_aquatilis_var_diffusa
FCA2267-11|Bennett_06-385_CAN|Ranunculus_aquatilis_var_diffusa
FCA2427-11|Aiken_05-001_CAN|Ranunculus_hyperboreus
FCA2429-11|Edlund_388_CAN|Ranunculus_gmelinii
FCA2428-11|Brysting_01-096_CAN|Ranunculus_hyperboreus
FCA1565-11|Edlund_12776_CAN|Ranunculus_gmelinii
FCA2809-11|Gillespie_9875_CAN|Ranunculus_gmelinii
FCA187-09|Gillespie_et_al_8494|Ranunculus_hyperboreus
FCA150-09|Gillespie_et_al_8072|Ranunculus_gmelinii
FCA2426-11|Brysting_01-100_CAN|Ranunculus_hyperboreus
FCA1566-11|Gillespie_7261_CAN|Ranunculus_hyperboreus
FCA2509-11|Saarela_1449_CAN|Ranunculus_hyperboreus
FCA2361-11|Aiken_88-017_CAN|Ranunculus_gmelinii
FCA2422-11|Brunton_10690_CAN|Ranunculus_gmelinii
FCA1578-11|Murray_10196_CAN|Ranunculus_arcticus
FCA1563-11|Hammar_2532-99_CAN|Ranunculus_arcticus
FCA330-10|Gillespie_6820|Ranunculus_sabinei
FCA1561-11|Aiken_05-068_CAN|Ranunculus_nivalis
FCA2810-11|Gillespie_9876_CAN|Ranunculus_arcticus
FCA1574-11|Brysting_01-069_CAN|Ranunculus_arcticus
FCA2425-11|Cumbaa_sn_CAN585864|Ranunculus_nivalis
FCA2746-11|Gillespie_9757_CAN|Ranunculus_nivalis
FCA1583-11|Elven_2848-9_CAN|Ranunculus_sabinei
FCA1582-11|Elven_2342-9_CAN|Ranunculus_sabinei
FCA047-09|Gillespie_et_al_7535|Ranunculus_arcticus
FCA1575-11|Aiken_02-009b_CAN|Ranunculus_arcticus
FCA245-09|Bosquet_4|Ranunculus_nivalis
FCA2019-11|Consaul_3520_CAN|Ranunculus_arcticus
FCA2513-11|Saarela_1453_CAN|Ranunculus_arcticus
FCA2424-11|Aiken_04-016_CAN|Ranunculus_nivalis
FCA1562-11|Aiken_05-056_CAN|Ranunculus_arcticus
FCA1577-11|Aiken_99-204_CAN|Ranunculus_arcticus
FCA1584-11|Elven_2997-9_CAN|Ranunculus_sabinei
FCA1564-11|Gillespie_7094_CAN|Ranunculus_arcticus
FCA1560-11|Aiken_04-079c_CAN|Ranunculus_nivalis
FCA1588-11|Elven_2662-9_CAN|Ranunculus_sulphureus
FCA369-10|Gillespie_6970|Ranunculus_arcticus
FCA1571-11|Gillespie_7131_CAN|Ranunculus_nivalis
FCA1585-11|Gillespie_7289_CAN|Ranunculus_sabinei
FCA1587-11|Gillespie_6644_CAN|Ranunculus_sulphureus
FCA329-10|Gillespie_6819|Ranunculus_sulphureus
FCA1586-11|Gillespie_6617_CAN|Ranunculus_sulphureus
FCA2042-11|Consaul_3625_CAN|Ranunculus_allenii
FCA2853-11|Gillespie_9974_CAN|Ranunculus_pygmaeus
FCA156-09|Gillespie_et_al_8096|Ranunculus_pygmaeus
FCA1567-11|Elven_2036-9_CAN|Ranunculus_gelidus
FCA1580-11|Gillespie_7273_CAN|Ranunculus_pygmaeus
FCA1579-11|Elven_3174-9_CAN|Ranunculus_pygmaeus
FCA1581-11|Gillespie_6766_CAN|Ranunculus_pygmaeus
FCA2268-11|Bennett_08-583_CAN|Coptidium_pallasii
FCA1572-11|Aiken_05-064_CAN|Coptidium_pallasii
FCA1570-11|Elven_2035-9_CAN|Coptidium_lapponicum
FCA2432-11|Edlund_290_CAN|Halerpestes_cymbalaria
FCA080-09|Gillespie_et_al_7682|Halerpestes_cymbalaria
FCA2431-11|Edlund_601_CAN|Halerpestes_cymbalaria
FCA2430-11|Brunton_10600_CAN|Halerpestes_cymbalaria
FCA2097-11|Consaul_3823_CAN|Halerpestes_cymbalaria
FCA680-10|Gillespie_8672|Anemone_richardsonii
FCA1597-11|Gillett_18763_CAN|Anemone_richardsonii
FCA728-10|Gillespie_8757|Anemone_parviflora
FCA1596-11|Gordon_sn_CAN523749|Anemone_parviflora
FCA278-09|Burt_s.n._CAN593356|Anemone_parviflora
FCA076-09|Gillespie_et_al_7661|Anemone_parviflora
FCA609-10|Gillespie_8561|Anemone_parviflora
FCA1595-11|Elven_2051-9_CAN|Anemone_drummondii
FCA1594-11|Lambert_sn_CAN529362|Pulsatilla_patens_ssp_multifida
FCA1603-11|Salomons_1_CAN|Pulsatilla_patens_ssp_multifida
FCA2265-11|Bennett_08-167_CAN|Aconitum_delphiniifolium
FCA2360-11|Cooper_1142_CAN|Aconitum_delphiniifolium
FCA661-10|Gillespie_8635|Caltha_palustris
FCA1599-11|Aiken_99-029_CAN|Caltha_palustris
FCA577-10|Gillespie_8523|Caltha_palustris
FCA2261-11|Consaul_988_CAN|Caltha_palustris
FCA1600-11|Pokiak_11_CAN|Caltha_palustris
FCA1601-11|Gillespie_7099_CAN|Caltha_palustris
FCA1598-11|Edlund_12771_CAN|Caltha_palustris
0.0050

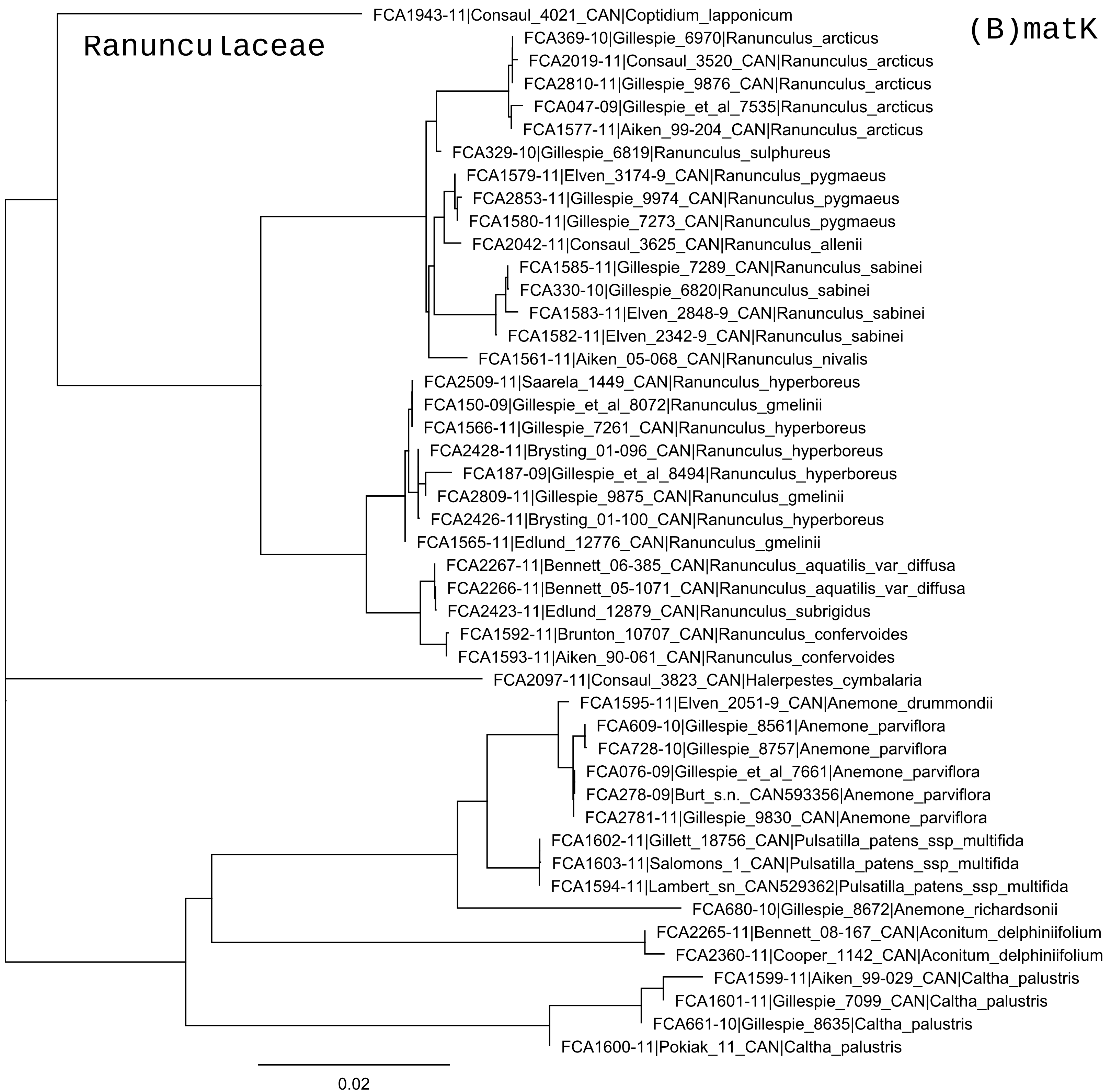
(B)matK
Ranunculaceae
FCA1943-11|Consaul_4021_CAN|Coptidium_lapponicum
FCA369-10|Gillespie_6970|Ranunculus_arcticus
FCA2019-11|Consaul_3520_CAN|Ranunculus_arcticus
FCA2810-11|Gillespie_9876_CAN|Ranunculus_arcticus
FCA047-09|Gillespie_et_al_7535|Ranunculus_arcticus
FCA1577-11|Aiken_99-204_CAN|Ranunculus_arcticus
FCA329-10|Gillespie_6819|Ranunculus_sulphureus
FCA1579-11|Elven_3174-9_CAN|Ranunculus_pygmaeus
FCA2853-11|Gillespie_9974_CAN|Ranunculus_pygmaeus
FCA1580-11|Gillespie_7273_CAN|Ranunculus_pygmaeus
FCA2042-11|Consaul_3625_CAN|Ranunculus_allenii
FCA1585-11|Gillespie_7289_CAN|Ranunculus_sabinei
FCA330-10|Gillespie_6820|Ranunculus_sabinei
FCA1583-11|Elven_2848-9_CAN|Ranunculus_sabinei
FCA1582-11|Elven_2342-9_CAN|Ranunculus_sabinei
FCA1561-11|Aiken_05-068_CAN|Ranunculus_nivalis
FCA2509-11|Saarela_1449_CAN|Ranunculus_hyperboreus
FCA150-09|Gillespie_et_al_8072|Ranunculus_gmelinii
FCA1566-11|Gillespie_7261_CAN|Ranunculus_hyperboreus
FCA2428-11|Brysting_01-096_CAN|Ranunculus_hyperboreus
FCA187-09|Gillespie_et_al_8494|Ranunculus_hyperboreus
FCA2809-11|Gillespie_9875_CAN|Ranunculus_gmelinii
FCA2426-11|Brysting_01-100_CAN|Ranunculus_hyperboreus
FCA1565-11|Edlund_12776_CAN|Ranunculus_gmelinii
FCA2267-11|Bennett_06-385_CAN|Ranunculus_aquatilis_var_diffusa
FCA2266-11|Bennett_05-1071_CAN|Ranunculus_aquatilis_var_diffusa
FCA2423-11|Edlund_12879_CAN|Ranunculus_subrigidus
FCA1592-11|Brunton_10707_CAN|Ranunculus_confervoides
FCA1593-11|Aiken_90-061_CAN|Ranunculus_confervoides
FCA2097-11|Consaul_3823_CAN|Halerpestes_cymbalaria
FCA1595-11|Elven_2051-9_CAN|Anemone_drummondii
FCA609-10|Gillespie_8561|Anemone_parviflora
FCA728-10|Gillespie_8757|Anemone_parviflora
FCA076-09|Gillespie_et_al_7661|Anemone_parviflora
FCA278-09|Burt_s.n._CAN593356|Anemone_parviflora
FCA2781-11|Gillespie_9830_CAN|Anemone_parviflora
FCA1602-11|Gillett_18756_CAN|Pulsatilla_patens_ssp_multifida
FCA1603-11|Salomons_1_CAN|Pulsatilla_patens_ssp_multifida
FCA1594-11|Lambert_sn_CAN529362|Pulsatilla_patens_ssp_multifida
FCA680-10|Gillespie_8672|Anemone_richardsonii
FCA2265-11|Bennett_08-167_CAN|Aconitum_delphiniifolium
FCA2360-11|Cooper_1142_CAN|Aconitum_delphiniifolium
FCA1599-11|Aiken_99-029_CAN|Caltha_palustris
FCA1601-11|Gillespie_7099_CAN|Caltha_palustris
FCA661-10|Gillespie_8635|Caltha_palustris
FCA1600-11|Pokiak_11_CAN|Caltha_palustris
0.02

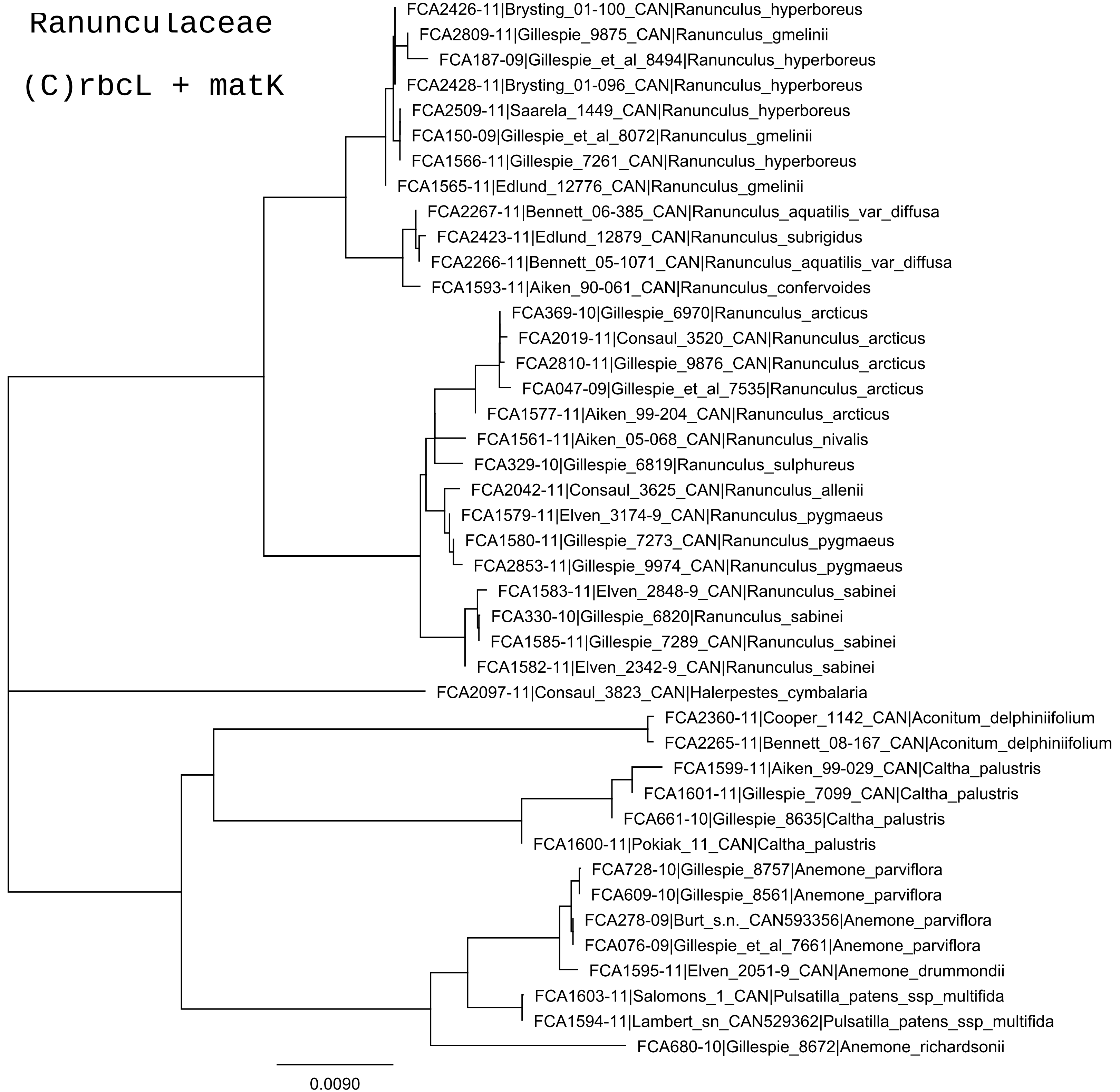
Ranunculaceae
(C)rbcL + matK
FCA2426-11|Brysting_01-100_CAN|Ranunculus_hyperboreus
FCA2809-11|Gillespie_9875_CAN|Ranunculus_gmelinii
FCA187-09|Gillespie_et_al_8494|Ranunculus_hyperboreus
FCA2428-11|Brysting_01-096_CAN|Ranunculus_hyperboreus
FCA2509-11|Saarela_1449_CAN|Ranunculus_hyperboreus
FCA150-09|Gillespie_et_al_8072|Ranunculus_gmelinii
FCA1566-11|Gillespie_7261_CAN|Ranunculus_hyperboreus
FCA1565-11|Edlund_12776_CAN|Ranunculus_gmelinii
FCA2267-11|Bennett_06-385_CAN|Ranunculus_aquatilis_var_diffusa
FCA2423-11|Edlund_12879_CAN|Ranunculus_subrigidus
FCA2266-11|Bennett_05-1071_CAN|Ranunculus_aquatilis_var_diffusa
FCA1593-11|Aiken_90-061_CAN|Ranunculus_confervoides
FCA369-10|Gillespie_6970|Ranunculus_arcticus
FCA2019-11|Consaul_3520_CAN|Ranunculus_arcticus
FCA2810-11|Gillespie_9876_CAN|Ranunculus_arcticus
FCA047-09|Gillespie_et_al_7535|Ranunculus_arcticus
FCA1577-11|Aiken_99-204_CAN|Ranunculus_arcticus
FCA1561-11|Aiken_05-068_CAN|Ranunculus_nivalis
FCA329-10|Gillespie_6819|Ranunculus_sulphureus
FCA2042-11|Consaul_3625_CAN|Ranunculus_allenii
FCA1579-11|Elven_3174-9_CAN|Ranunculus_pygmaeus
FCA1580-11|Gillespie_7273_CAN|Ranunculus_pygmaeus
FCA2853-11|Gillespie_9974_CAN|Ranunculus_pygmaeus
FCA1583-11|Elven_2848-9_CAN|Ranunculus_sabinei
FCA330-10|Gillespie_6820|Ranunculus_sabinei
FCA1585-11|Gillespie_7289_CAN|Ranunculus_sabinei
FCA1582-11|Elven_2342-9_CAN|Ranunculus_sabinei
FCA2097-11|Consaul_3823_CAN|Halerpestes_cymbalaria
FCA2360-11|Cooper_1142_CAN|Aconitum_delphiniifolium
FCA2265-11|Bennett_08-167_CAN|Aconitum_delphiniifolium
FCA1599-11|Aiken_99-029_CAN|Caltha_palustris
FCA1601-11|Gillespie_7099_CAN|Caltha_palustris
FCA661-10|Gillespie_8635|Caltha_palustris
FCA1600-11|Pokiak_11_CAN|Caltha_palustris
FCA728-10|Gillespie_8757|Anemone_parviflora
FCA609-10|Gillespie_8561|Anemone_parviflora
FCA278-09|Burt_s.n._CAN593356|Anemone_parviflora
FCA076-09|Gillespie_et_al_7661|Anemone_parviflora
FCA1595-11|Elven_2051-9_CAN|Anemone_drummondii
FCA1603-11|Salomons_1_CAN|Pulsatilla_patens_ssp_multifida
FCA1594-11|Lambert_sn_CAN529362|Pulsatilla_patens_ssp_multifida
FCA680-10|Gillespie_8672|Anemone_richardsonii
0.0090
