# Supplementary material for: DNA Barcoding the Canadian Arctic Flora: Core Plastid Barcodes (rbcL + matK) for 490 Vascular Plant Species
Source: PLoS One. 2013 Oct 22;8(10):e77982. doi: 10.1371/journal.pone.0077982 (PMC3865322; doi:10.1371/journal.pone.0077982)
Supplement: Figure S49 — Neighbour joining analysis of uncorrected p-distances of psbA–trnH sequence data for Puccinellia and Poa (Poaceae). (PDF) [file pone.0077982.s054.pdf]

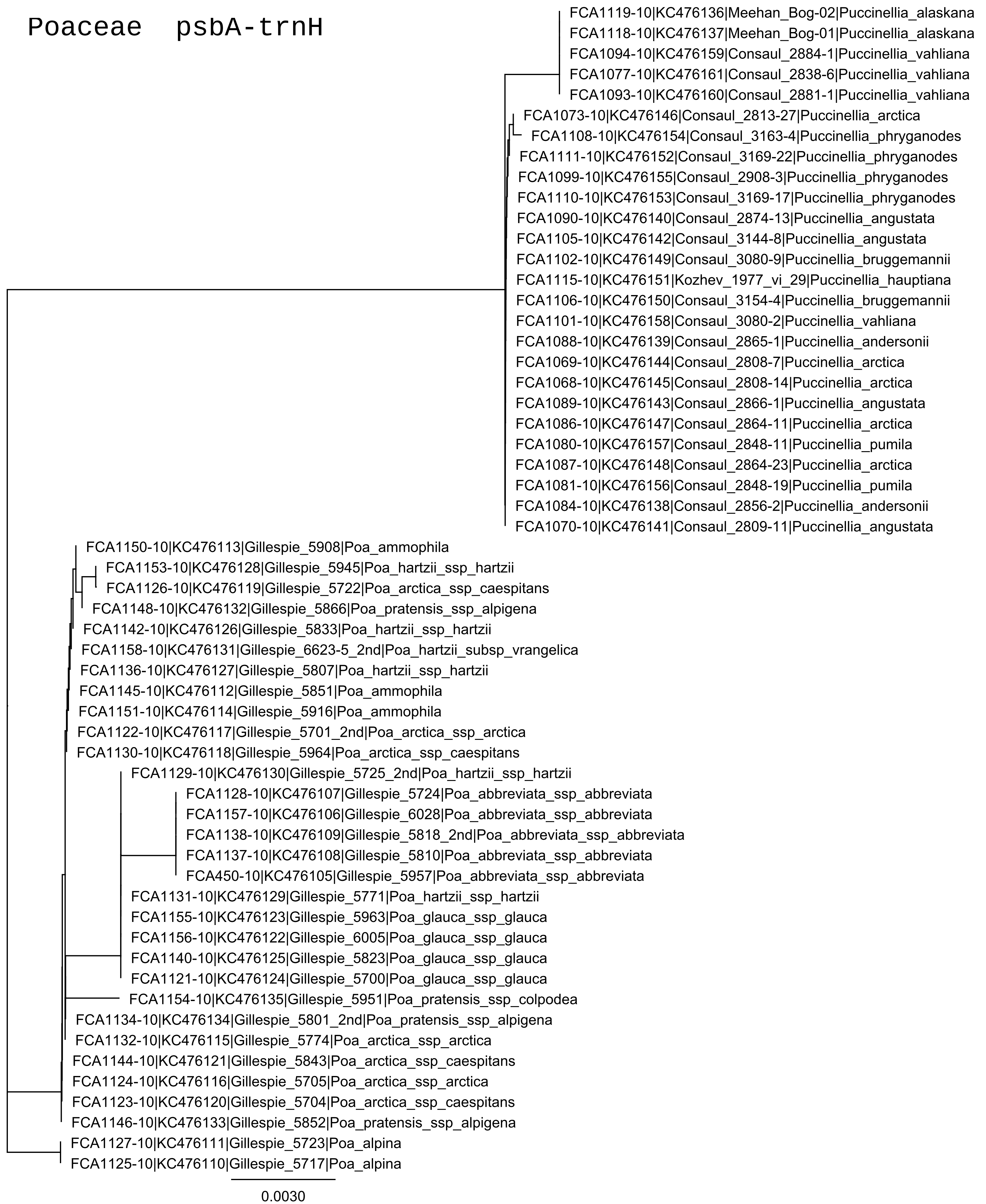
Poaceae psbA-trnH
FCA1119-10|KC476136|Meehan_Bog-02|Puccinellia_alaskana
FCA1118-10|KC476137|Meehan_Bog-01|Puccinellia_alaskana
FCA1094-10|KC476159|Consaul_2884-1|Puccinellia_vahliana
FCA1077-10|KC476161|Consaul_2838-6|Puccinellia_vahliana
FCA1093-10|KC476160|Consaul_2881-1|Puccinellia_vahliana
FCA1073-10|KC476146|Consaul_2813-27|Puccinellia_arctica
FCA1108-10|KC476154|Consaul_3163-4|Puccinellia_phryganodes
FCA1111-10|KC476152|Consaul_3169-22|Puccinellia_phryganodes
FCA1099-10|KC476155|Consaul_2908-3|Puccinellia_phryganodes
FCA1110-10|KC476153|Consaul_3169-17|Puccinellia_phryganodes
FCA1090-10|KC476140|Consaul_2874-13|Puccinellia_angustata
FCA1105-10|KC476142|Consaul_3144-8|Puccinellia_angustata
FCA1102-10|KC476149|Consaul_3080-9|Puccinellia_bruggemannii
FCA1115-10|KC476151|Kozhev_1977_vi_29|Puccinellia_hauptiana
FCA1106-10|KC476150|Consaul_3154-4|Puccinellia_bruggemannii
FCA1101-10|KC476158|Consaul_3080-2|Puccinellia_vahliana
FCA1088-10|KC476139|Consaul_2865-1|Puccinellia_andersonii
FCA1069-10|KC476144|Consaul_2808-7|Puccinellia_arctica
FCA1068-10|KC476145|Consaul_2808-14|Puccinellia_arctica
FCA1089-10|KC476143|Consaul_2866-1|Puccinellia_angustata
FCA1086-10|KC476147|Consaul_2864-11|Puccinellia_arctica
FCA1080-10|KC476157|Consaul_2848-11|Puccinellia_pumila
FCA1087-10|KC476148|Consaul_2864-23|Puccinellia_arctica
FCA1081-10|KC476156|Consaul_2848-19|Puccinellia_pumila
FCA1084-10|KC476138|Consaul_2856-2|Puccinellia_andersonii
FCA1070-10|KC476141|Consaul_2809-11|Puccinellia_angustata
FCA1150-10|KC476113|Gillespie_5908|Poa_ammophila
FCA1153-10|KC476128|Gillespie_5945|Poa_hartzii_ssp_hartzii
FCA1126-10|KC476119|Gillespie_5722|Poa_arctica_ssp_caespitans
FCA1148-10|KC476132|Gillespie_5866|Poa_pratensis_ssp_alpigena
FCA1142-10|KC476126|Gillespie_5833|Poa_hartzii_ssp_hartzii
FCA1158-10|KC476131|Gillespie_6623-5_2nd|Poa_hartzii_subsp_vrangelica
FCA1136-10|KC476127|Gillespie_5807|Poa_hartzii_ssp_hartzii
FCA1145-10|KC476112|Gillespie_5851|Poa_ammophila
FCA1151-10|KC476114|Gillespie_5916|Poa_ammophila
FCA1122-10|KC476117|Gillespie_5701_2nd|Poa_arctica_ssp_arctica
FCA1130-10|KC476118|Gillespie_5964|Poa_arctica_ssp_caespitans
FCA1129-10|KC476130|Gillespie_5725_2nd|Poa_hartzii_ssp_hartzii
FCA1128-10|KC476107|Gillespie_5724|Poa_abbreviata_ssp_abbreviata
FCA1157-10|KC476106|Gillespie_6028|Poa_abbreviata_ssp_abbreviata
FCA1138-10|KC476109|Gillespie_5818_2nd|Poa_abbreviata_ssp_abbreviata
FCA1137-10|KC476108|Gillespie_5810|Poa_abbreviata_ssp_abbreviata
FCA450-10|KC476105|Gillespie_5957|Poa_abbreviata_ssp_abbreviata
FCA1131-10|KC476129|Gillespie_5771|Poa_hartzii_ssp_hartzii
FCA1155-10|KC476123|Gillespie_5963|Poa_glauca_ssp_glauca
FCA1156-10|KC476122|Gillespie_6005|Poa_glauca_ssp_glauca
FCA1140-10|KC476125|Gillespie_5823|Poa_glauca_ssp_glauca
FCA1121-10|KC476124|Gillespie_5700|Poa_glauca_ssp_glauca
FCA1154-10|KC476135|Gillespie_5951|Poa_pratensis_ssp_colpodea
FCA1134-10|KC476134|Gillespie_5801_2nd|Poa_pratensis_ssp_alpigena
FCA1132-10|KC476115|Gillespie_5774|Poa_arctica_ssp_arctica
FCA1144-10|KC476121|Gillespie_5843|Poa_arctica_ssp_caespitans
FCA1124-10|KC476116|Gillespie_5705|Poa_arctica_ssp_arctica
FCA1123-10|KC476120|Gillespie_5704|Poa_arctica_ssp_caespitans
FCA1146-10|KC476133|Gillespie_5852|Poa_pratensis_ssp_alpigena
FCA1127-10|KC476111|Gillespie_5723|Poa_alpina
FCA1125-10|KC476110|Gillespie_5717|Poa_alpina
0.0030
